# Intramolecular *ortho* Photocycloaddition of 4-Substituted 7-(4′-Alkenyloxy)-1-indanones and Ensuing Reaction Cascades

**DOI:** 10.1021/acs.joc.5c00171

**Published:** 2025-03-11

**Authors:** Audrey Gilbert, Julian Zuber, Thorsten Bach

**Affiliations:** Department Chemie and Catalysis Research Center (CRC), Technische Universität München, Lichtenbergstr. 4, 85747 Garching, Germany

## Abstract

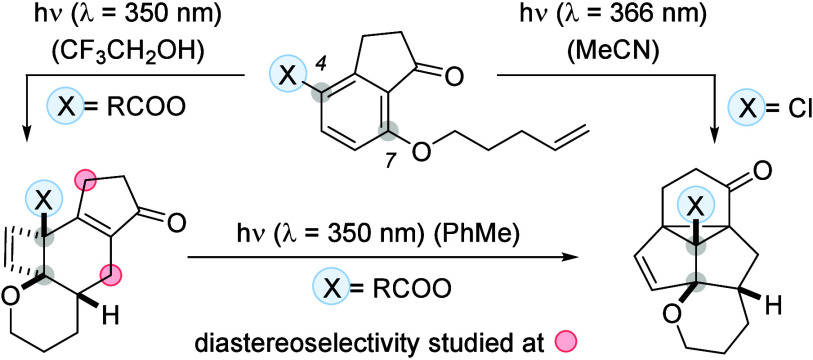

4-Substituted 7-(4′-alkenyloxy)-1-indanones were prepared from the respective substituted aryl propanoic acids and subjected to UV-A irradiation (λ = 350 or 366 nm). While the 4-chloro compound was directly converted at λ = 366 nm into a pentacyclic product (47% yield) by a three-photon cascade process, the oxygenated substrates reacted in trifluoroethanol at λ = 350 nm by a two-photon cascade, involving an *ortho* photocycloaddition, a thermal disrotatory ring opening, and a [4π] photocyclization (six examples, 67–82% yield). An ensuing photochemical di-π-methane rearrangement of the latter products was achieved by irradiation at λ = 350 nm in toluene (five examples, 36–70% yield). The diastereoselectivity of the reaction was probed employing a chiral 1-indanone with a stereogenic center at carbon atom C3. 1-Indanones with a 4-hexenyloxy side chain [(*E*)- or (*Z*)-configured] at carbon atom C7 served to interrogate the stereospecifity of the reaction.

## Introduction

**Scheme 1:**
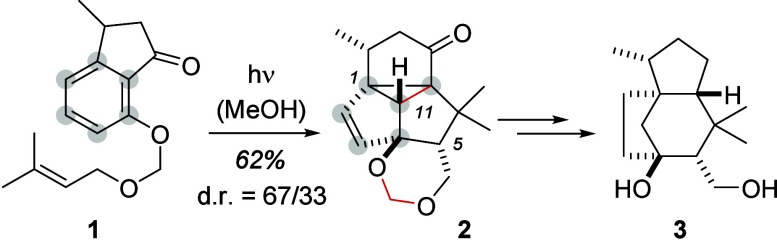
Total Synthesis of Agarozizanol B (**3**) from Indanone **1** via Pentacyclic Photocascade Product **2**

Upon absorption of a photon, a photochemical substrate reaches excited states which are energetically way above the ground state. Photons with a wavelength of λ = 300 nm correspond to an energy of approximately 400 kJ mol^–1^. Not surprisingly, subsequent reactions of photoexcited compounds enable the cleavage of strong bonds and the formation of highly strained molecules. Photochemical dearomatization reactions^1^ enforce an addition to double bonds of a stabilized benzene ring and allow one to individually address the six sp^2^-hybridized ring carbon atoms in further transformations. As an example, the benzene core of substrate **1** served to deliver the marked carbon atoms of the tetracyclo[5.3.1.0^1,7^0^4,11^]undec-2-ene unit in product **2**. The reaction represents the key step in the synthesis of the sesquiterpene natural product agarozizanol B (**3**), which has been accomplished by our group in 2021 (Scheme 1).^2^

**Scheme 2:**
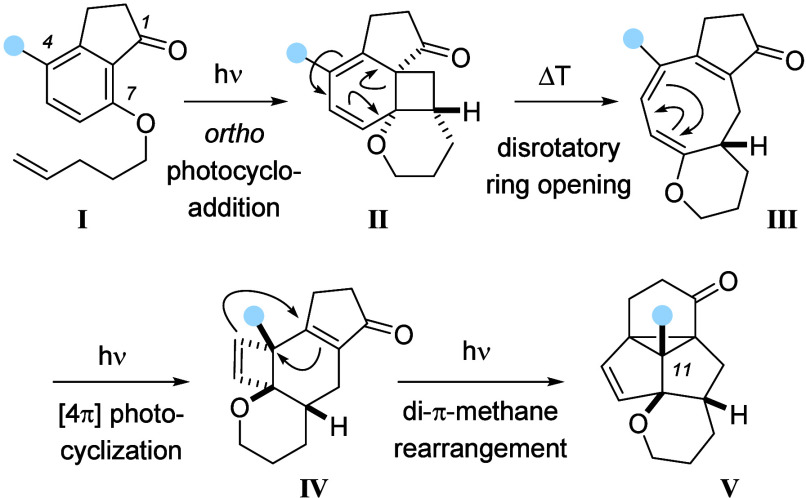
Proposed Mechanism for the Formation of Pentacyclic Products **V** from 7-(4′-Alkenyloxy)-1-indanones **I**

Although the facial diastereoselectivity (d.r. = diastereomeric ratio) induced by the existing stereogenic center within **1** was only moderate, the relative configuration at the newly formed five stereogenic centers was established in a defined fashion. Three stereogenic centers (at C1, C4, and C5) of **2** were retained in the natural product, which was synthesized in nine consecutive steps upon cleavage of the single bonds marked in red. Based on the isolation of intermediates and previous work in the field of arene photocycloaddition reactions,^3,4^ it is likely that the formation of product **2** is the result of a three-photon cascade reaction, converting 7-(4′-alkenyloxy)-substituted 1-indanones of general structure **I** into products **V** (Scheme 2).

The reaction is initiated by an *ortho* photocycloaddition providing tetracyclic intermediate **II**, which escapes the high ring strain of the cyclohexadiene-annulated cyclobutane by a rapid, thermally allowed disrotatory ring opening. However, the resulting cyclooctatriene **III** presents a suitable chromophore to capture another photon which in turn facilitates a disrotatory [4π] cyclization to cyclobutene **IV**. Remarkably, the formed enone chromophore in **IV** induces a third photochemical step, a di-π-methane rearrangement, completing the sequence with the formation of the pentacyclic product **V**. Although the transformation had shown notable scope in the choice of the alkenyl chain and in the substitution pattern at aliphatic carbon atoms,^5^ the important substituent at the C4 position of the substrate **I** (marked in blue) had not been varied beyond hydrogen and methyl. Several potential target molecules require a functionalization at position C11 of the tetracyclic core of product **V**, and it seemed worth attempting a cascade reaction that already included this very substituent. In this manuscript, we describe the synthesis of the respective 4-substituted 1-indanone precursors and the ensuing photochemical steps. A yet unknown solvent dependence on the reaction outcome was observed, and several functionalized products of type **V** could be isolated.

## Results and Discussion

### Initial Substrate Screening and Photochemical Studies

**Scheme 3:**
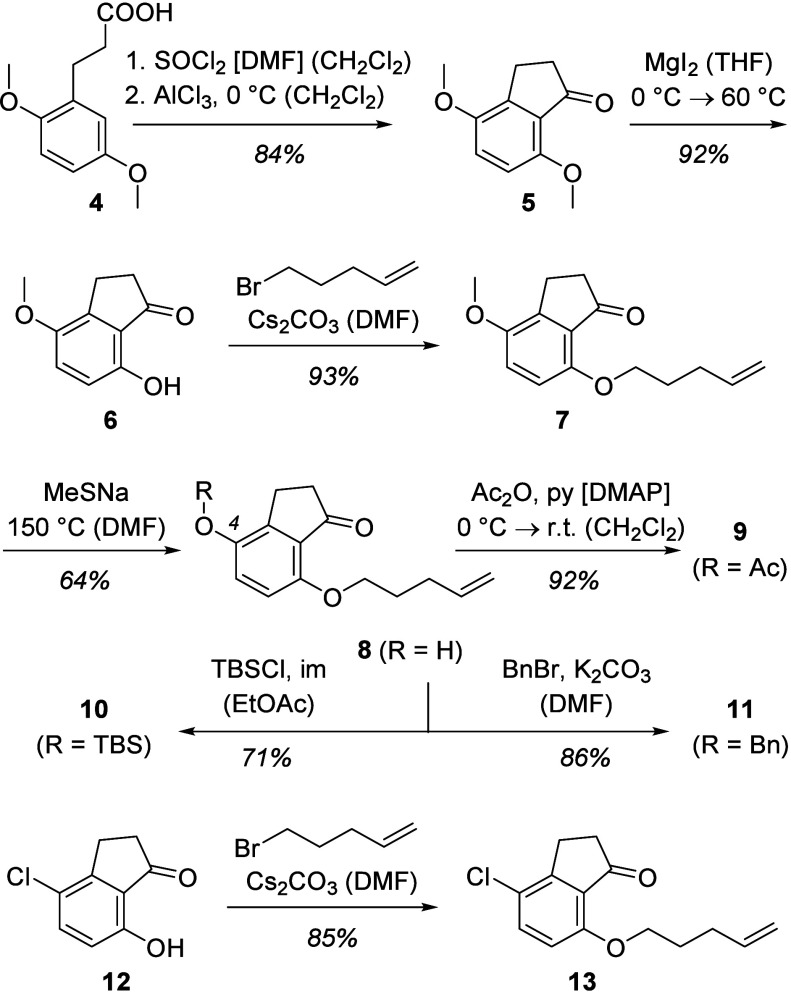
Synthesis of 1-Indanone Precursors for Initial Screening Studies

The initial selection of 1-indanone derivatives aimed at oxygen and chlorine substituents at position C4 with a 4-pentenyloxy tethering of the olefin to position C7 of the indanone skeleton. Oxygenated products with different protecting groups (Ac = acetyl, TBS = *tert*-butyldimethysilyl, Bn = benzyl) were obtained from the 4-hydroxy compound **8** which was accessible in five steps from known 3-(2,5-dimethoxyphenyl)propanoic acid (**4**)^6^ (Scheme 3).

The acid was converted with thionyl chloride into the respective acid chloride, which underwent an intramolecular Friedel–Crafts acylation when exposed to aluminum trichloride in dichloromethane. 4,7-Dimethoxy-1-indanone (**5**) was selectively demethylated with magnesium iodide^7^ to furnish 7-hydroxy-1-indanone **6**. Alkylation with 1-bromo-4-pentene delivered diether **7**, which was selectively demethylated by treatment with sodium methanethiolate at elevated temperature.^8^ Attachment of the mentioned protecting groups at the free hydroxy group of indanone **8** proceeded uneventfully and delivered desired products **9**–**11**. 4-Chloro-7-hydroxy-1-indanone (**12**) was accessible by a known procedure^9^ from commercially available 4-chlorophenol. Alkylation in analogy to the transformation **6** → **7** gave chlorinated indanone **13** in high yield.

**Scheme 4:**
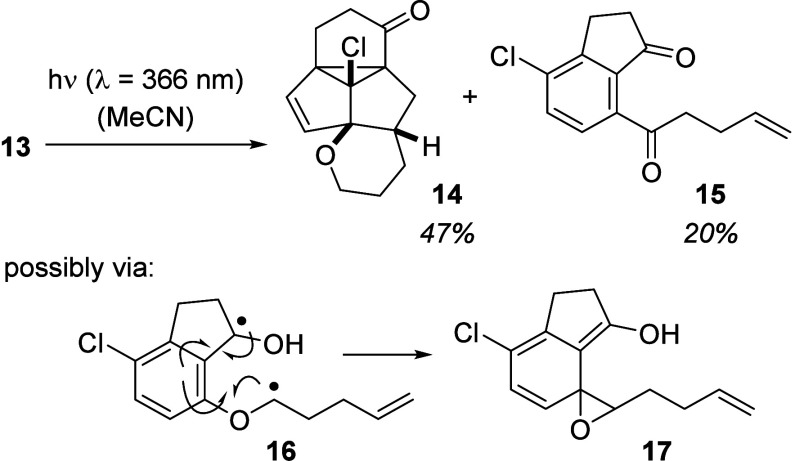
Photochemistry of 4-Chloro-1-indanone 13: Formation of the Desired Product **14** and of Ketone **15** by an Alternative Reaction Pathway

Irradiation experiments with 4-chloro-1-indanone **13** were initially performed under the conditions found optimal for nonchlorinated indanones like compound **1**.^2,5^ The compound was subjected in MeOH solution (*c* = 10 mM) to light from fluorescent lamps emitting at a maximum of λ = 350 nm. The desired product was not found, but there were indications for MeOH having ring-opened a putative cyclopropane intermediate (for details of the optimization, see the Supporting Information). In addition, a byproduct was isolated which appeared to be an oxidation product of the starting material. Other solvents were evaluated, and acetonitrile turned out to be a superior choice, avoiding any undesired cyclopropane ring opening. Variation of the light source and the reaction temperature led to the best result at λ = 366 nm and ambient temperature. In a preparative run (80 μmol scale), 47% of desired tricyclic product **14** was obtained. In addition, a side product was isolated, to which structure **15** (20% yield) was tentatively assigned (Scheme 4).

Compound **15** was clearly an aryl ketone, with a molecular mass varying by 2 Da from the molecular mass of the starting material. We suspect it to be formed by hydrogen atom abstraction at the carbon atom adjacent to the ether oxygen atom. This step has precedence in the reaction of *ortho*-substituted aromatic ketones. In very close analogy to our result, Wagner and co-workers observed in studies on the Norrish-Yang cyclization of *ortho*-alkoxy phenyl ketones the formation of a phenyl ketone from the respective benzyl ether by a similar rearrangement.^10^ Like Wagner *et al.*, we suspect that 1,5-diradical **16** might form labile epoxide **17** in which the ring opens to ketone **15**. The oxidation might be triggered by oxygen during the workup or–less likely–by residual oxygen in the solvent.

**Scheme 5:**
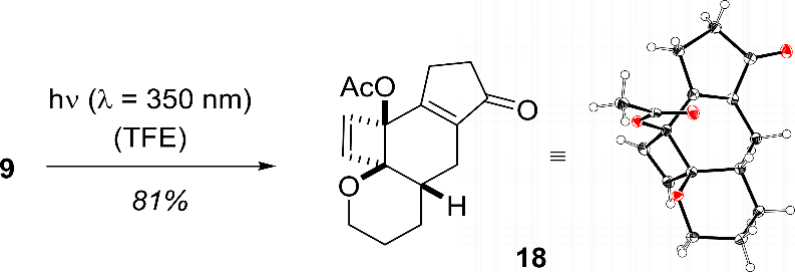
Isolation of Product **18** under Optimized Conditions and Its Molecular Structure in the Solid State Atomic displacement parameters given at the 50% probability level.

For oxygenated substrates **10** and **11**, preliminary irradiation results did not look promising. Upon excitation at λ = 350 nm in MeOH solution, complete degradation was recorded for 4-silyloxy-substituted indanone **10**. Under the same conditions, 4-methoxy- and 4-benzyloxy-substituted indanones **7** and **11** did not react and were recovered quantitatively. Only acetate **9** showed notable conversion, and a new product was identified. Further screening of possible solvents (see the Supporting Information for details) revealed that the reaction was cleanest in trifluoroethanol (TFE).^11^ Full conversion was achieved after 6 h, and the product was isolated in 81% yield (Scheme 5).

Closer inspection of the NMR spectra revealed that the compound lacked the expected tetracyclo[5.3.1.0^1,7^0^4,11^]undec-2-ene core (cf. structure **V**, Scheme 2). Rather, the reaction had stalled at the stage of the cyclobutene intermediate **18** (cf. structure **IV**, Scheme 2). The molecular structure of the latter compound and its relative configuration were corroborated by single-crystal X-ray diffraction (Note: The molecular structure shown in Scheme 5 corresponds to the inverted structure of the CIF file; see the Supporting Information). The outcome was remarkable because in previous work the ensuing di-π-methane rearrangement could only be suppressed when short-wavelength light below λ < 350 nm was excluded by applying a Fe_2_(SO_4_)_3_ filter solution.^12^ UV/vis spectra of product **18** in protic solvents (MeOH, TFE) showed the absence of any notable absorption above λ ≥ 300 nm. The substrate displayed an absorption at λ = 320 nm (ε = 3980 M^–1^ cm^–1^, MeOH) which is likely responsible for its reactivity. In fact, there is significant overlap between the emission spectrum of the lamps employed for irradiation and the UV/vis absorption of indanone **9**.

### Substrate Variation and Consecutive Reactions

**Scheme 6:**
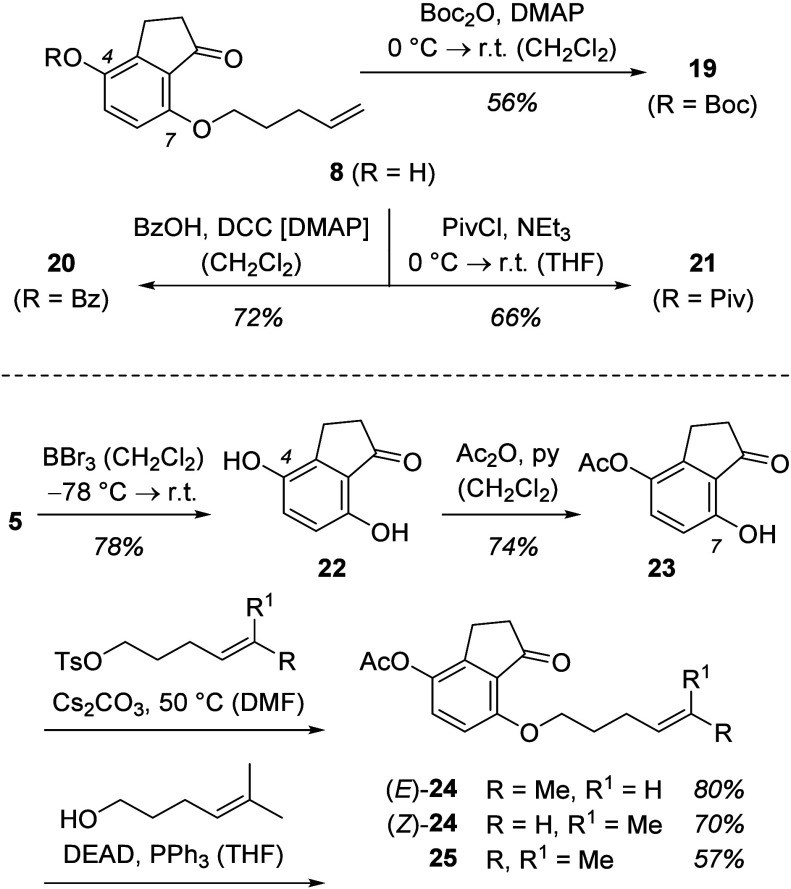
Synthesis of 1-Indanone Precursors for an Evaluation of the Substrate Scope

Given the unique access to a complex carbon skeleton with an intriguing dioxygenated cyclobutene core, we looked into the scope of this two-photon cascade reaction. In view of the failed attempts with substrates **10** and **11**, it seemed warranted to use 1-indanones as substrates, the C4 oxygen atom of which was substituted by an electron withdrawing group. Indanone **8** served as the precursor which was acylated (Scheme 6) to deliver products with the 4-hydroxy group being protected by a *tert*-butoxycarbonyl (Boc) group (product **19**), a benzoyl (Bz) group (product **20**), or a pivaloyl (Piv) group (product **21**).

Alternative 4-alkenyl tethers were attached to the oxygen atom at C7 by a Williamson ether synthesis or by a Mitsunobu reaction. Starting from dimethyl ether **5**, a removal of the methyl groups was accomplished with boron tribromide in dichloromethane.^13^ The resulting dihydroxy-1-indanone **22** was monoacetylated at carbon atom C4 before installing the tether at the hydroxy group of 7-hydroxy-1-indanone **23**. A 4-hexenyl chain was introduced via the respective tosylate (Ts = toluenesulfonyl) derived from either (*E*)-4-hexenol or (*Z*)-4-hexenol.^5a,14^ The configuration was retained in the alkylation reactions, and photosubstrates (*E*)-**24** and (*Z*)-**24** were obtained as pure diastereoisomers (d.r. ≥ 95/5). A trisubstituted olefin was part of the tether in indanone **25**, which was accessed by a Mitsunobu reaction (DEAD = diethyl azodicarboxylate) employing 5-methyl-4-hexenol as the reagent.^14^

**Scheme 7:**
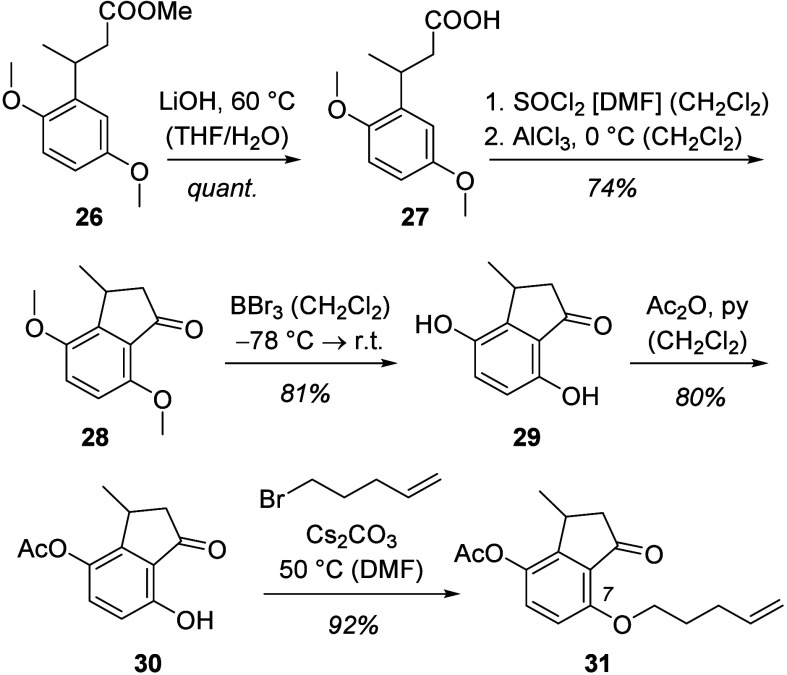
Synthesis of Chiral 1-Indanone **31** from Trisubstituted Benzene **26**

The synthesis of a chiral indanone with a stereogenic center at C3 was a bit more laborious and commenced with methyl 3-(2,5-dimethoxyphenyl)butanoate (**26**) (Scheme 7), which was synthesized from known^15^ methyl (*E*)-3-(2,5-dimethoxyphenyl)acrylate (see the Supporting Information for details). Saponification delivered carboxylic acid **27** which was converted to indanone **28** in full analogy to the transformation **4** → **5**. Comprehensive demethylation delivered dihydroxyindanone **29** which was selectively acetylated at the C4 oxygen atom to product **30**. Alkylation delivered desired indanone **31** with the terminal olefin tethered to carbon atom C7.

**Figure 1:**
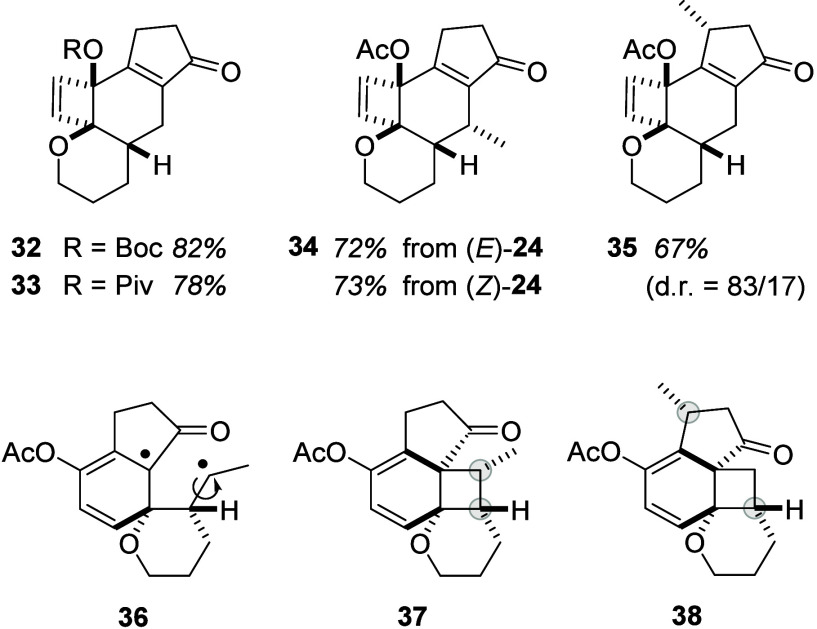
Products and yields achieved upon irradiation of the respective 1-indanone precursors **19**, **21**, **24**, and **31** at λ = 350 nm in TFE (top) and postulated intermediates **36**, **37**, and **38** involved in the formation of products **34** and **35** (bottom).

Irradiation of all substrates was performed under the same conditions previously applied to indanone **9** (λ = 350 nm, *c* = 10 mM in TFE). Among the three differently protected 4-hydroxy-1-indanones, Boc- and Piv-protected substrates **19** and **21** reacted smoothly and delivered products **32** and **33** in high yields (Figure 1).

The Bz-substituted compound **20** reacted to an array of products, with the desired compound detectable but not isolable. Likewise, the sterically encumbered, trisubstituted olefin **25** underwent decomposition upon irradiation in TFE, and no distinct product was isolated. The disubstituted olefins (*E*)-**24** and (*Z*)-**24** with the terminal methyl group reacted in a stereoconvergent fashion; i.e., both diastereoisomers furnished the same product **34** irrespective of the initial olefin configuration (Figure 1). Based on nuclear Overhauser spectroscopy (NOESY) studies, the methyl group is located *trans* to the adjacent hydrogen atom within the tetrahydropyran ring. The stereoconvergent reaction course suggests^16^ that initial *ortho* photocycloaddition (cf. **I** → **II**, Scheme 2) occurs from the first excited triplet state^17^ of the 1-indanone. The stereochemical information is erased at the stage of 1,4-diradical **36** before forming the cyclobutane intermediate **37**. The relative configuration at the two stereogenic centers marked in gray remains unchanged in the course of the further reaction. The chiral indanone **31** delivered the product as a mixture of diastereoisomers with a remarkably high facial diastereoselectivity (d.r. = 83/17) when compared to the reaction of substrate **1** (d.r. = 67/33). The relative configuration of major diastereoisomer **35** was assigned by NOESY studies, and it is in line with an initial approach of the olefin to the arene core from the face that is better accessible. The methyl group shields the bottom face of the indanone, and the *ortho* photocycloaddition delivers preferentially intermediate **38** with the two indicated carbon atoms retaining their relative configuration throughout the further reaction cascade.

**Scheme 8:**
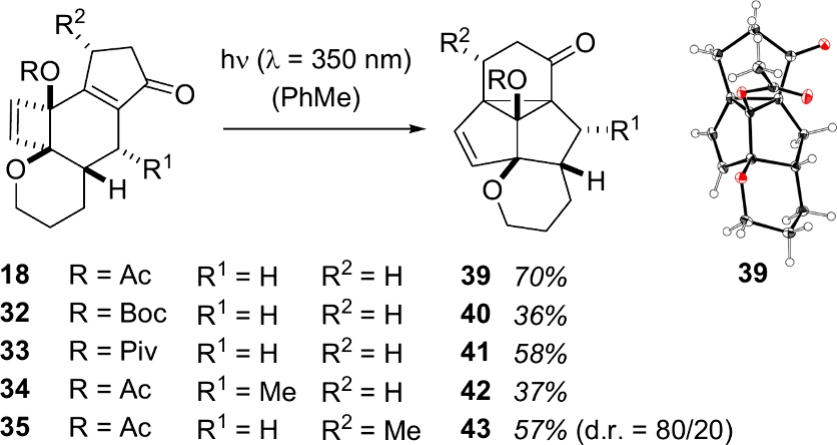
Photochemical di-π-Methane Rearrangement to Pentacyclic Products with an Oxygenated Cyclopropane Ring and Molecular Structure of Product **39** in the Solid State Atomic displacement parameters given at the 50% probability level.

Unlike in methanol, the UV/vis spectra of compound **18** in toluene revealed a weak absorption at λ ≥ 300 nm. The band was broad with three maxima to be seen at a high concentration (*c* = 10 mM). The longest wavelength maximum was at λ = 334 nm with an absorption coefficient of ε ≅ 80 M^–1^ cm^–1^. Although we have not substantiated the hypothesis, it is tempting to assume that hydrogen bonds to methanol or TFE weakens (or blue shifts) the nπ* transition of the carbonyl chromophore,^11^ and thus, an excitation in this solvent is avoided. The transition visible in toluene should consequently be suited to initiate the di-π-methane rearrangement. In fact, irradiation of compound **18** with the 350 nm fluorescent lamps led to product formation in toluene solution. The new product **39** displayed the expected pentacyclic structure (Scheme 8), and its relative configuration was established by single crystal X-ray analysis.

The crystal structure also confirmed the position of the acetoxy group at the cyclopropane ring, and the structural assignment was adapted to the other pentacyclic products **40**–**43** obtained from the photochemical di-π-methane rearrangement. For product **42**, it was assumed that the relative configuration of the stereogenic center carrying the methyl group (R^1^ = Me) and the adjacent stereogenic center within the tetrahydropyran ring remains intact. Similarly, the major diastereoisomer **43** (R^2^ = Me) obtained from the reaction of substrate **35** was assumed to retain its relative configuration, and the observed d.r. supports this notion. Attempts to directly access compounds **39**–**43** from indanone precursors by irradiation at various wavelengths and in different solvents remained unsuccessful. The initial reaction to cyclobutenes **18** and **32**–**35** requires TFE as protic solvent, and it remained sluggish in other solvents.

**Scheme 9:**
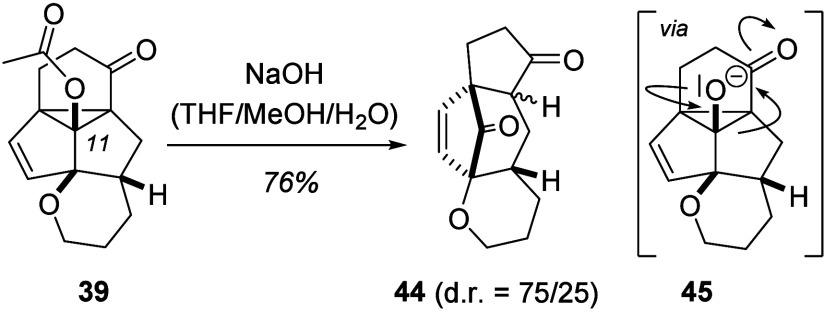
Base-Induced Fragmentation of Photocascade Product **39**

Several consecutive reactions of tetracyclo[5.3.1.0^1,7^0^4,11^]undec-2-ene had been looked at in previous work,^2,5a^ and they are expected to also be applicable to products **39**–**43**. In the course of the current study, we briefly investigated a retro-aldol cleavage that is only feasible with an oxygenated substituent at position C11. Upon saponification of acetate **39**, the cyclopropane ring was opened and product **44**, obtained (Scheme 9).

The reaction likely occurs via alkoxide **45** which undergoes fragmentation to provide the enolate of an annulated cyclopentanone. Subsequent protonation appears to be not completely diastereoselective but delivered a mixture of two stereoisomers (d.r. = 75/25) which were separable by preparative HPLC. An assignment of the relative configuration was not possible since crystallization results did not deliver suitable crystals and NOESY experiments remained inconclusive.

## Conclusion

In summary, the irradiation of 7-(4′-alkenyloxy)-1-indanones with a functional group at position C4 of the indanone core delivers, in a single operation complex, three-dimensional structures with several points for further functionalization (exit vectors). Acyloxy substituents at C4 offer the opportunity to access two different core structures in a photochemically triggered cascade. At λ = 350 nm in TFE, tetracyclic products **18** and **32**–**35** were accessed which invite a further photochemical step to provide pentacyclic products **39**–**43**. A chlorine atom at C4 enables a direct photochemical three-photon cascade to the pentacyclic product **14**. The study serves as an example of how the planarity of arenes^18^ can be readily overcome by photochemical methods.

## Experimental Section

### General Methods

Air and moisture sensitive reactions were carried out in oven-dried glassware sealed with rubber septa under a positive pressure of dry argon. Irradiation experiments were conducted in a photochemical reactor equipped with 16 fluorescence lamps (λ_max_ = 350 or 366 nm). Dry tetrahydrofuran (THF), dichloromethane (CH_2_Cl_2_), and diethyl ether (Et_2_O) were obtained from a solvent purification system. Other dry solvents, e.g., methanol (MeOH), were obtained in the highest purity available stored over molecular sieves. Analytical thin layer chromatography (TLC) was performed on silica gel 60 (F_254_) glass plates. The TLC plates were visualized by either ultraviolet (UV) light (λ = 254 nm) or treatment with KMnO_4_ stain, followed by gentle heating. Purification of products was accomplished by flash chromatography on silica gel 60 (230–400 mesh). All solvents for chromatography, e.g., hexane, ethyl acetate (EtOAc), were distilled prior to use. NMR spectra were measured on either a 400 or a 500 MHz nuclear magnetic resonance spectrometer. The ^1^H NMR spectra were calibrated against the residual solvent peak of either chloroform (7.26 ppm) or acetone-*d*_6_ (2.05 ppm), and the ^13^C{^1^H} NMR spectra were calibrated against either the central peak of CDCl_3_ (77.16 ppm) or the residual solvent peak of acetone-*d*_6_ (29.84 ppm). Data for ^1^H NMR spectra were reported as follows: chemical shift in parts per million (ppm), peak shape (s = singlet, d = doublet, t = triplet, q = quartet, quin = quintet, h = sextet, m = multiplet, and br = broad), coupling constant in Hertz (Hz), and integration. The assignment of signals to diastereoisomers are based on integration of the signals in the NMR of the mixture of isomers unless otherwise indicated. The relative configuration of products was determined by two-dimensional NMR spectra (COSY, HSQC, HMBC, NOESY). High resolution mass spectroscopy (HR-MS) was performed on a Thermo Scientific LTQ-FT Ultra (ESI) or on a double focusing magnetic sector instrument (EI, 70 eV). Infrared spectra were recorded by the attenuated total reflection (ATR) technique and are reported as wave numbers *ṽ* (cm^–1^).

#### General Procedure A for the Irradiation of 1-Indanone Precursors

To a flame-dried Duran phototube were successively added the precursor (1 equiv) and the solvent (0.01 M). The reaction mixture was purged with argon (ultrasound, 15 min). The phototube was equipped with an argon-filled balloon and subjected to irradiation (λ_max_ = 350 nm) for 6 h. The solvent was next removed *in vacuo*, and the crude mixture was purified over flash chromatography to give the respective photoproduct.

#### Characterization of Products

##### 3a^1^-Chloro-1,2,4a,5,6,7-hexahydro-3*H*,3a^1^*H*,4*H*-cyclopenta[1′,2′]cyclopropa[3,4]pentaleno[6a,1-b]pyran-3-one (**14**)

4-Chloro-7-(pent-4-en-1-yloxy)-2,3-dihydro-1*H*-inden-1-one (**13**) (20 mg, 0.08 mmol, 1 equiv) and MeCN (16 mL, 0.005 M) were submitted to the general procedure A for 24 h to generate the title compound as a colorless oil (9.4 mg, 0.04 mmol, 47%) after purification over flash chromatography on silica gel using hexane/EtOAc (9:1) as the eluent. TLC (hexane/EtOAc 9:1): *R*_f_ = 0.25 [UV, KMnO_4_]. ^1^H NMR (500 MHz, CDCl_3_) δ (ppm) = 6.18 (d, *J* = 6.2 Hz, 1H), 5.96 (d, *J* = 6.2 Hz, 1H), 4.05 (ddt, *J* = 12.0, 5.1, 1.4 Hz, 1H), 3.80 (td, *J* = 12.4, 3.1 Hz, 1H), 2.52–2.44 (m, 2H), 2.40–2.32 (m, 3H), 2.30–2.21 (m, 1H), 1.81–1.68 (m, 2H), 1.65–1.60 (m, 1H), 1.41–1.33 (m, 1H), 0.92 (dd, *J* = 13.3, 11.8 Hz, 1H). ^13^C{^1^H} NMR (126 MHz, CDCl_3_) δ (ppm) = 207.2, 130.6, 127.6, 93.5, 75.3, 65.0, 58.5, 57.7, 51.8, 36.6, 25.8, 24.2, 23.3, 21.1. HRMS (ESI): calcd for C_14_H_16_ClO_2_^+^ [M + H^+^] = 251.0833, found = 251.0834. IR (ATR): ν̃ (cm^–1^) = 2932, 1717, 1455, 1364, 1280, 1093, 937, 831.

##### 4-Chloro-7-(pent-4-enoyl)-2,3-dihydro-1*H*-inden-1-one (**15**)

The title compound was obtained from the experiment described above as a colorless oil (4.0 mg, 0.02 mmol, 20%) after purification over flash chromatography on silica gel using hexane/EtOAc (9:1) as the eluent. TLC (hexane/EtOAc 9:1): *R*_f_ = 0.39 [UV, KMnO_4_]. ^1^H NMR (500 MHz, CDCl_3_) δ (ppm) = 7.60 (d, *J* = 7.9 Hz, 1H), 7.22 (dt, *J* = 7.9, 0.8 Hz, 1H), 5.86 (ddt, *J* = 16.8, 10.2, 6.5 Hz, 1H), 5.06 (dq, *J* = 17.1, 1.7 Hz, 1H), 4.99 (dq, *J* = 10.2, 1.4 Hz, 1H), 3.20–3.14 (m, 2H), 2.98 (t, *J* = 7.6 Hz, 2H), 2.79–2.74 (m, 2H), 2.51–2.44 (m, 2H). ^13^C{^1^H} NMR (126 MHz, CDCl_3_) δ (ppm) = 205.0, 204.8, 153.1, 138.4, 137.1, 135.5, 134.8, 134.3, 127.1, 115.5, 42.7, 36.1, 28.2, 25.4. HRMS (ESI): calcd for C_14_H_14_ClO_2_^+^ [M + H^+^] = 249.0677, found = 249.0677. IR (ATR): ν̃ (cm^–1^) = 2927, 1720, 1584, 1468, 1327, 1259, 1128, 914.

##### 8-Oxo-4,5,6,6a,7,8,9,10-octahydro-10b*H*-cyclobuta[i]cyclopenta[g]chromen-10b-yl Acetate (**18**)

1-Oxo-7-(pent-4-en-1-yloxy)-2,3-dihydro-1*H*-inden-4-yl acetate (**9**) (100 mg, 0.36 mmol, 1 equiv) and trifluoroethanol (36 mL, 0.01 M) were submitted to the general procedure A to generate the title compound as a white solid (81.1 mg, 0.30 mmol, 81%) after purification over flash chromatography on silica gel using hexane/EtOAc (1:1) as the eluent. TLC (hexane/EtOAc 1:1): *R*_f_ = 0.3 [UV, KMnO_4_]. ^1^H NMR (500 MHz, CDCl_3_) δ (ppm) = δ 6.97 (d, *J* = 3.0 Hz, 1H), 6.39 (s, 1H), 3.97 (ddt, *J* = 11.6, 5.0, 1.5 Hz, 1H), 3.68–3.59 (m, 1H), 2.61–2.34 (m, 5H), 2.16 (s, 3H), 1.93–1.69 (m, 2H), 1.64–1.61 (m, 1H), 1.48–1.29 (m, 2H). ^13^C{^1^H} NMR (126 MHz, CDCl_3_) δ (ppm) = 207.3, 170.2, 170.1, 139.6, 139.2, 137.0, 88.4, 82.5, 65.4, 36.9, 34.7, 26.5, 26.1, 25.5, 24.0, 21.0. HRMS (ESI): calcd for C_16_H_19_O_4_^+^ [M + H^+^] = 275.1278, found = 275.1272. IR (ATR): ν̃ (cm^–1^) = 2931, 1744, 1693, 1538, 1369, 1231, 1051, 904.

##### *tert*-Butyl(8-oxo-4,5,6,6a,7,8,9,10-octahydro-10b*H*-cyclobuta[i]cyclopenta[g]chromen-10b-yl) Carbonate (**32**)

*tert*-Butyl (1-oxo-7-(pent-4-en-1-yloxy)-2,3-dihydro-1*H*-inden-4-yl) carbonate (**19**) (20 mg, 0.06 mmol, 1 equiv) and trifluoroethanol (6 mL, 0.01 M) were submitted to the general procedure A to generate the title compound as a white solid (16.5 mg, 0.05 mmol, 82%) after purification over flash chromatography on silica gel using hexane EtOAc (7:3) as the eluent. TLC (hexane/EtOAc 7:3): *R*_f_ = 0.33 [UV, KMnO_4_]. ^1^H NMR (500 MHz, CDCl_3_) δ (ppm) = 6.94 (d, *J* = 3.1 Hz, 1H), 6.35 (d, *J* = 3.0 Hz, 1H), 3.96 (dd, *J* = 11.7, 4.9 Hz, 1H), 3.64 (td, *J* = 12.2, 2.7 Hz, 1H), 2.67–2.33 (m, 5H), 1.85–1.67 (m, 3H), 1.63–1.57 (m, 1H), 1.47 (s, 9H), 1.43–1.28 (m, 2H). ^13^C{^1^H} NMR (126 MHz, CDCl_3_) δ (ppm) = 207.3, 170.4, 152.1, 139.7, 139.2, 137.4, 88.1, 83.0, 82.7, 65.4, 36.9, 34.8, 27.7, 26.5, 26.3, 25.6, 24.0. HRMS (ESI): calcd for C_19_H_25_O_5_^+^ [M + H^+^] = 333.1697, found = 333.1692. IR (ATR): ν̃ (cm^–1^) = 2931, 1746, 1698, 1639, 1336, 1283, 1160, 1084.

##### 8-Oxo-4,5,6,6a,7,8,9,10-octahydro-10b*H*-cyclobuta[i]cyclopenta[g]chromen-10b-yl Pivalate (**33**)

1-Oxo-7-(pent-4-en-1-yloxy)-2,3-dihydro-1H-inden-4-yl pivalate (**21**) (50 mg, 0.16 mmol, 1 equiv) and trifluoroethanol (16 mL, 0.01 M) were submitted to the general procedure A to generate the title compound as a white solid (38.4 mg, 0.12 mmol, 78%) after purification over flash chromatography on silica gel using hexane EtOAc (8:2) as the eluent. TLC (hexane/EtOAc 8:2): *R*_f_ = 0.22 [UV, KMnO_4_]. ^1^H NMR (500 MHz, CDCl_3_) δ (ppm) = 6.94 (d, *J* = 3.1 Hz, 1H), 6.35 (s, 1H), 3.88 (dd, *J* = 10.8, 4.4 Hz, 1H), 3.59 (td, *J* = 12.3, 2.8 Hz, 1H), 2.62–2.28 (m, 5H), 1.86–1.66 (m, 3H), 1.60–1.53 (m, 1H), 1.45–1.28 (m, 2H), 1.24 (s, 9H). ^13^C{^1^H} NMR (126 MHz, CDCl_3_) δ (ppm) = 207.3, 177.6, 170.9, 139.5, 139.1, 136.6, 88.4, 82.2, 65.2, 38.7, 36.8, 34.7, 27.3, 26.6, 26.2, 25.3, 24.1. HRMS (ESI): calcd for C_19_H_25_O_4_^+^ [M + H^+^] = 317.1747, found = 317.1744. IR (ATR): ν̃ (cm^–1^) = 2931, 1736, 1699, 1639, 1480, 1285, 1152, 1053.

##### 7-Methyl-8-oxo-4,5,6,6a,7,8,9,10-octahydro-10b*H*-cyclobuta[i]cyclopenta[g]chromen-10b-yl Acetate (**34**)

(*Z*)-7-(Hex-4-en-1-yloxy)-1-oxo-2,3-dihydro-1*H*-inden-4-yl acetate [(*Z*)-**24**] (36.1 mg, 0.13 mmol, 1 equiv) and trifluoroethanol (13 mL, 0.01 M) were submitted to the general procedure A to generate the title compound as a white solid (26.4 mg, 0.09 mmol, 73%, >20:1 d.r.) after purification over flash chromatography on silica gel using hexane EtOAc (6:4) as the eluent. (*E*)-7-(Hex-4-en-1-yloxy)-1-oxo-2,3-dihydro-1*H*-inden-4-yl acetate [(*E*)-**24**] (50 mg, 0.17 mmol, 1 equiv) and trifluoroethanol (17 mL, 0.01 M) were submitted to the general procedure A to generate the title compound as a white solid (36.4 mg, 0.13 mmol, 72%, >20:1 d.r.) after purification over flash chromatography on silica gel using hexane EtOAc (6:4) as the eluent. The relative configuration of the title compound was determined by NOESY experiments (see the Supporting Information). TLC (hexane/EtOAc 6:4): *R*_f_ = 0.31 [UV, KMnO_4_]. ^1^H NMR (500 MHz, CDCl_3_) δ (ppm) = 6.93 (d, *J* = 3.0 Hz, 1H), 6.55 (d, *J* = 3.0 Hz, 1H), 3.95 (dd, *J* = 11.6, 4.9 Hz, 1H), 3.63 (td, *J* = 12.0, 2.5 Hz, 1H), 2.71–2.62 (m, 1H), 2.60–2.48 (m, 1H), 2.43–2.37 (m, 3H), 2.17–2.10 (m, 4H), 1.82–1.72 (m, 1H), 1.70–1.59 (m, 3H), 0.83 (d, *J* = 7.5 Hz, 3H). ^13^C{^1^H} NMR (126 MHz, CDCl_3_) δ (ppm) = 207.6, 169.9, 169.4, 143.8, 141.7, 141.1, 87.9, 82.2, 65.4, 40.6, 34.7, 29.3, 26.6, 25.3, 23.8, 20.9, 10.5. HRMS (ESI): calcd for C_17_H_21_O_4_^+^ [M + H^+^] = 289.1434, found = 289.1430. IR (ATR): ν̃ (cm^–1^) = 2925, 2871, 1732, 1692, 1639, 1370, 1235, 1047.

##### 10-Methyl-8-oxo-4,5,6,6a,7,8,9,10-octahydro-10b*H*-cyclobuta[i]cyclopenta[g]chromen-10b-yl Acetate (**35**)

3-Methyl-1-oxo-7-(pent-4-en-1-yloxy)-2,3-dihydro-1*H*-inden-4-yl acetate (**31**) (50 mg, 0.17 mmol, 1 equiv) and trifluoroethanol (17 mL, 0.01 M) were submitted to the general procedure A to generate the title compound as a mixture of inseparable diastereoisomers (33.7 mg, 0.12 mmol, 67%, d.r. = dia 1/dia 2 = 83/17) after purification over flash chromatography on silica gel using hexane EtOAc (6:4) as the eluent. The relative configuration of the diastereoisomers was determined via NOESY experiments (see the Supporting Information). TLC (hexane/EtOAc 6:4): *R*_f_ = 0.34 [UV, KMnO_4_]. ^1^H NMR (500 MHz, CDCl_3_) δ (ppm) = 6.98–6.88 (dia 1 + 2, m, 1.20H), 6.48–6.40 (dia 2, m, 0.20H), 6.30 (dia 1, d, *J* = 3.0 Hz, 1H), 3.95 (dia 1 + 2, ddt, *J* = 11.6, 5.2, 1.5 Hz, 1.20H), 3.68–3.46 (dia 1 + 2, m, 1.20H), 3.10–2.95 (dia 2, m, 0.20H), 2.89–2.77 (dia 1, m, 1H), 2.67 (dia 1 + 2, dia 1 = dd, *J* = 18.6, 6.5 Hz, dia 2 = dd, *J* = 18.7, 6.5 Hz, 1.20H), 2.47 (dia 1 + 2, dia 1 = ddd, *J* = 16.3, 4.1, 1.7 Hz, dia 2 = dd, *J* = 17.0, 4.2 Hz, 1.20H), 2.15 (dia 1, s, 3H), 2.13 (dia 2, s, 0.60H), 2.02 (dia 2, dd, *J* = 18.6, 2.3 Hz, 0.20H), 1.96 (dia 1, dd, *J* = 18.6, 1.9 Hz, 1H), 1.85–1.66 (dia 1 + 2, m, 3.60H), 1.63–1.52 (dia 1 + 2, m, 1.20H), 1.45–1.29 (dia 1 + 2, m, 2.40H), 1.26–1.16 (dia 1 + 2, m, 3.60H). ^13^C{^1^H} NMR (126 MHz, CDCl_3_) δ (ppm) = 206.5 (dia 1), 206.3 (dia 2), 174.0 (dia 1), 171.8 (dia 2), 170.4 (dia 2), 170.0 (dia 1), 139.8 (dia 1 + 2), 139.3 (dia 1 + 2), 136.2 (dia 1 + 2), 88.9 (dia 1), 88.0 (dia 2), 82.4 (dia 1 + 2), 65.4 (dia 1), 65.3 (dia 2), 44.8 (dia 2), 43.7 (dia 1), 36.9 (dia 1 + 2), 34.1 (dia 1), 34.0 (dia 2), 26.7 (dia 2), 26.5 (dia 1), 26.14 (dia 1), 26.10 (dia 2), 24.3 (dia 2), 23.8 (dia 1), 21.4 (dia 2), 20.9 (dia 1), 19.8 (dia 1 + 2). HRMS (ESI): calcd for C_17_H_21_O_4_^+^ [M + H^+^] = 289.1434, found = 239.1431. IR (ATR): ν̃ (cm^–1^) = 2932, 1747, 1698, 1370, 1298, 1231, 1059, 968.

#### General Procedure B for the Photochemical di-π-Methane Rearrangement

To a flame-dried Duran phototube were successively added the precursor (1 equiv) and the solvent (0.01 M). The reaction mixture was purged with argon (ultrasound, 15 min). The phototube was equipped with an argon-filled balloon and subjected to irradiation (λ_max_ = 350 nm) for 24 h. The solvent was next removed *in vacuo*, and the crude mixture was purified over flash chromatography to give the desired photoproducts.

#### Characterization of the Products

##### 3-Oxo-2,3,4a,5,6,7-hexahydro-1*H*,3a^1^*H*,4*H*-cyclopenta [1′,2′]cyclopropa[3,4]pentaleno [6a,1-b]pyran-3a^1^-yl Acetate (**39**)

8-Oxo-4,5,6,6a,7,8,9,10-octahydro-10b*H*-cyclobuta[*i*] cyclopenta[*g*]chromen-10b-yl acetate (**18**) (100 mg, 0.49 mmol, 1 equiv) and toluene (48 mL, 0.01 M) were submitted to the general procedure B to generate the title compound as a white solid (70.2 mg, 0.34 mmol, 70%) after purification over flash chromatography on silica gel using hexane EtOAc (7:3) as the eluent. TLC (hexane/EtOAc 7:3): *R*_f_ = 0.32 [UV, KMnO_4_]. ^1^H NMR (500 MHz, CDCl_3_) δ (ppm) = 6.18 (d, *J* = 6.2 Hz, 1H), 5.97 (d, *J* = 6.2 Hz, 1H), 3.94–3.88 (m, 1H), 3.76 (td, *J* = 12.4, 3.2 Hz, 1H), 2.46 (dd, *J* = 13.0, 8.1 Hz, 1H), 2.42–2.28 (m, 3H), 2.27–2.21 (m, 1H), 2.20–2.08 (m, 4H), 1.76–1.62 (m, 3H), 1.42–1.29 (m, 1H), 0.85 (t, *J* = 12.5 Hz, 1H). ^13^C{^1^H} NMR (126 MHz, CDCl_3_) δ (ppm) = 206.7, 170.1, 130.1, 127.4, 91.8, 89.9, 64.7, 58.3, 56.0, 52.2, 36.2, 26.1, 24.2, 22.8, 21.2, 20.5. HRMS (ESI): calcd for C_16_H_19_O_4_^+^ [M + H^+^] = 275.1278, found = 275.1279. IR (ATR): ν̃ (cm^–1^) = 2935, 2865, 1766, 1713, 1368, 1225, 1195, 1047.

##### *tert*-Butyl (3-Oxo-2,3,4a,5,6,7-hexahydro-1*H*,3a^1^*H*,4*H*-cyclopenta[1′,2′]cyclopropa[3,4]penta-leno[6a,1-b]pyran-3a^1^-yl) Carbonate (**40**)

*tert*-Butyl(8-oxo-4,5,6,6a,7,8,9,10-octahydro-10b*H*-cyclobuta[*i*]cyclopenta[*g*]chromen-10b-yl) carbonate (**32**) (26.8 mg, 0.08 mmol, 1 equiv) and toluene (8.1 mL, 0.01 M) were submitted to the general procedure B to generate the title compound as a yellow oil (12.1 mg, 0.04 mmol, 36%) after purification over flash chromatography on silica gel using hexane EtOAc (8:2) as the eluent. The compound was isolated as a mixture with 20% of an unidentified side-product that could not be separated by flash chromatography. This has been subtracted from the yield. TLC (hexane/EtOAc 8:2): *R*_f_ = 0.32 [UV, KMnO_4_]. ^1^H NMR (500 MHz, CDCl_3_) δ (ppm) = 6.20 (d, *J* = 6.2 Hz, 1H), 5.97 (d, *J* = 6.3 Hz, 1H), 3.96–3.89 (m, 1H), 3.77 (td, *J* = 12.2, 3.0 Hz, 1H), 2.45 (dd, *J* = 12.9, 8.1 Hz, 1H), 2.37–2.27 (m, 4H), 1.76–1.65 (m, 2H), 1.62–1.56 (m, 2H), 1.49 (s, 9H), 0.82 (dd, *J* = 13.0, 11.4 Hz, 1H). ^13^C{^1^H} NMR (126 MHz, CDCl_3_) δ (ppm) = 211.1, 206.5, 130.1, 127.7, 91.7, 83.6, 64.7, 58.6, 56.2, 52.2, 36.2, 27.9, 27.7, 26.1, 24.2, 22.9, 21.1. HRMS (ESI): calcd for C_14_H_17_O_3_^+^ [M – Boc + H^+^] = 233.1172, found = 233.1173. IR (ATR): ν̃ (cm^–1^) = 2936, 2867, 1762, 1717, 1274, 1156, 1135, 1088.

##### 3-Oxo-2,3,4a,5,6,7-hexahydro-1*H*,3a^1^*H*,4*H*-cyclopenta[1′,2′]cyclopropa[3,4]pentaleno[6a,1-b]pyran-3a^1^-yl Pivalate (**41**)

8-Oxo-4,5,6,6a,7,8,9,10-octahydro-10b*H*-cyclobuta[*i*]cyclopenta[*g*]chromen-10b-yl pivalate (**33**) (74.9 mg, 0.24 mmol, 1 equiv) and toluene (24 mL, 0.01 M) were submitted to the general condition B to generate the title compound as a colorless oil (43.3 mg, 0.14 mmol, 58%) after purification over flash chromatography on silica gel using hexane EtOAc (85:15) as the eluent. TLC (hexane/EtOAc 85:15): *R*_f_ = 0.37 [UV, KMnO_4_]. ^1^H NMR (500 MHz, CDCl_3_) δ (ppm) = 6.17 (d, *J* = 6.2 Hz, 1H), 5.97 (d, *J* = 6.2 Hz, 1H), 3.90–3.82 (m, 1H), 3.74 (td, *J* = 12.1, 3.3 Hz, 1H), 2.46 (dd, *J* = 13.1, 8.1 Hz, 1H), 2.39–2.26 (m, 3H), 2.25–2.19 (m, 1H), 2.16–2.06 (m, 1H), 1.68–1.54 (m, 3H), 1.41–1.30 (m, 1H), 1.23 (s, 9H), 0.85 (t, *J* = 12.3 Hz, 1H). ^13^C{^1^H} NMR (126 MHz, CDCl_3_) δ (ppm) = 207.1, 177.8, 130.1, 127.5, 91.7, 89.9, 64.7, 58.6, 55.7, 52.2, 39.1, 36.2, 27.3, 26.2, 24.2, 22.8, 21.2. HRMS (ESI): calcd for C_19_H_25_O_4_^+^ [M + H^+^] = 317.1747, found = 317.1744. IR (ATR): ν̃ (cm^–1^) = 2935, 1756, 1716, 1480, 1369, 1280, 1116, 915.

##### 4-Methyl-3-oxo-2,3,4a,5,6,7-hexahydro-1*H*,3a^1^*H*,4*H*-cyclopenta[1′,2′]cyclopropa[3,4]pentaleno[6a,1-b]pyran-3a^1^-yl Acetate (**42**)

8-Oxo-4,5,6,6a,7,8,9,10-octahydro-10b*H*-cyclobuta[*i*] cyclopenta[*g*]chromen-10b-yl acetate (**34**) (49.2 mg, 0.17 mmol, 1 equiv) and toluene (17 mL, 0.01 M) were submitted to the general procedure B to generate the title compound as a colorless oil (18.2 mg, 0.06 mmol, 37%) after purification over flash chromatography on silica gel using hexane EtOAc (7:3) as the eluent. The final compound was not sufficiently stable to measure ^13^C NMR. TLC (hexane/EtOAc 7:3): *R*_f_ = 0.38 [UV, KMnO_4_]. ^1^H NMR (400 MHz, CDCl_3_) δ (ppm) = 6.29 (d, *J* = 6.2 Hz, 1H), 6.02 (d, *J* = 6.2 Hz, 1H), 3.94–3.86 (m, 1H), 3.79 (td, *J* = 12.1, 3.5 Hz, 1H), 3.15 (dqd, *J* = 10.6, 7.6, 1.0 Hz, 1H), 2.70 (ddd, *J* = 13.3, 10.5, 3.0 Hz, 1H), 2.40–2.29 (m, 2H), 2.26–2.19 (m, 1H), 2.15 (s, 3H), 2.14–2.06 (m, 1H), 1.72–1.62 (m, 3H), 1.54–1.36 (m, 1H), 0.82 (d, *J* = 7.5 Hz, 3H). HRMS (ESI): calcd for C_17_H_21_O_4_^+^ [M + H^+^] = 289.1434, found = 289.1434. IR (ATR): ν̃ (cm^–1^) = 2935, 1769, 1714, 1369, 1223, 1199, 1056, 917.

##### 1-Methyl-3-oxo-2,3,4a,5,6,7-hexahydro-1*H*,3a^1^*H*,4*H*-cyclopenta[1′,2′]cyclopropa[3,4]pentaleno[6a,1-b]pyran-3a^1^-yl Acetate (**43**)

The mixture of diastereoisomers of 10-methyl-8-oxo-4,5,6,6a,7,8,9,10-octahydro-10b*H*-cyclobuta[*i*]cyclopenta[*g*] chromen-10b-yl acetate (**35**) (40.5 mg, 0.14 mmol, 1 equiv) and toluene (14 mL, 0.01 M) was submitted to the general procedure B to generate the title compound as an inseparable mixture of diastereoisomers (23.1 mg, 0.08 mmol, 57%, dr = dia 1/dia 2 = 80/20) after purification over flash chromatography on silica gel using hexane EtOAc (7:3) as the eluent. TLC (hexane/EtOAc 7:3): *R*_f_ = 0.33 [UV, KMnO_4_]. ^1^H NMR (500 MHz, CDCl_3_) δ (ppm) = 6.20 (dia 1, d, *J* = 6.3 Hz, 1H), 6.19 (dia 2, d, *J* = 6.3 Hz, 0.25H), 6.10 (dia 1, d, *J* = 6.4 Hz, 1H), 5.92 (dia 2, d, *J* = 6.3 Hz, 0.25H), 3.95–3.85 (dia 1 + 2, m, 1.25H), 3.79–3.71 (dia 1 + 2, m, 1.25H), 2.82–2.70 (dia 2, m, 0.25H), 2.70–2.60 (dia 2, m, 0.25H), 2.55 (dia 1, p, *J* = 6.9 Hz, 1H), 2.50–2.31 (dia 1 + 2,m, 3.50H), 2.15 (dia 1, s, 3H), 2.14 (dia 2, s, 0.75H), 1.94–1.83 (dia 1 + 2, m, 1.25H), 1.75–1.57 (dia 1 + 2, m, 3.75H), 1.40–1.29 (dia 1 + 2,m, 1.25H), 1.21 (dia 2, d, *J* = 6.9 Hz, 0.75H), 1.16 (dia 1, d, *J* = 7.0 Hz, 3H), 0.85 (dia 1, t, *J* = 12.5 Hz, 1H), 0.81 (dia 2, t, *J* = 11.8 Hz, 0.25H). ^13^C{^1^H} NMR (126 MHz, CDCl_3_) δ (ppm) = 206.1 (dia 1), 205.1 (dia 2), 170.0 (dia 1), 169.8 (dia 2), 129.4 (dia 2), 128.3 (dia 2), 127.6 (dia 1), 127.5 (dia 1), 92.2 (dia 2), 91.7 (dia 1), 64.7 (dia 1), 63.5 (dia 2), 55.4 (dia 2), 52.4 (dia 1), 44.8 (dia 1), 43.7 (dia 2), 30.5 (dia 2), 27.8 (dia 1), 26.1 (dia 2), 26.0 (dia 1), 24.20 (dia 2), 24.18 (dia 2), 22.9 (dia 2), 22.7 (dia 1), 20.9 (dia 2), 20.5 (dia 1), 20.2 (dia 1 + 2), 17.1 (dia 1 + 2). HRMS (ESI): calcd for C_17_H_21_O_4_^+^ [M + H^+^] = 289.1434, found = 289.1430. IR (ATR): ν̃ (cm^–1^) = 2931, 1764, 1710, 1439, 1367, 1221, 1047, 921.

##### 2,3,4,4a,5,5a,7,8-Octahydro-6*H*-8a,10a-methanoazuleno[6,5-*b*]pyran-6,11-dione (**44**)

To a flask under argon were successively added 3-oxo-2,3,4a,5,6,7-hexahydro-1*H*,3a^1^*H*,4*H*-cyclopenta[1′,2′] cyclopropa[3,4]pentaleno [6a,1-*b*]pyran-3a^1^-yl acetate **39** (66.6 mg, 0.24 mmol, 1 equiv), THF (1.3 mL), and MeOH (0.44 mL). NaOH (19.4 mg, 0.49 mmol, 2 equiv) in H_2_O (0.66 mL) was added to the reaction, and the mixture was stirred at room temperature for 30 min before being diluted with water and acidified with 3 M HCl in water until a pH of ≤ 7. The mixture was extracted three times with EtOAc before the organic phases were combined, washed with brine, dried over Na_2_SO_4_, and concentrated *in vacuo*. The crude product was purified over flash chromatography on silica gel using hexane/EtOAc (6:4) as the eluent to yield the title compound as a mixture of diastereoisomers (43 mg, 0.19 mmol, 76%, dr = 75:25). TLC (hexane/EtOAc 6:4): *R*_f_ = 0.31 [KMnO_4_]. The diastereoisomers were separated by semipreparative HPLC (*i*PrOH:heptane 1:1) for characterization. Major diastereoisomer: ^1^H NMR (500 MHz, CDCl_3_) δ (ppm) = 6.63 (d, *J* = 7.4 Hz, 1H), 6.16 (d, *J* = 7.4 Hz, 1H), 4.05 (ddt, *J* = 11.7, 4.8, 1.6 Hz, 1H), 3.68 (td, *J* = 12.2, 2.5 Hz, 1H), 2.51 (ddt, *J* = 18.0, 9.5, 1.2 Hz, 1H), 2.46–2.39 (m, 1H), 2.36–2.28 (m, 1H), 2.26 (ddd, *J* = 12.0, 5.2, 1.2 Hz, 1H), 1.94 (dt, *J* = 13.9, 5.1 Hz, 1H), 1.91–1.86 (m, 1H), 1.84–1.67 (m, 3H), 1.63–1.58 (m, 1H), 1.28–1.22 (m, 1H), 1.12 (dt, *J* = 13.9, 11.7 Hz, 1H). ^13^C{^1^H} NMR (126 MHz, CDCl_3_) δ (ppm) = 213.3, 209.5, 130.8, 129.5, 85.9, 64.9, 58.1, 55.4, 39.1, 35.9, 28.2, 26.0, 25.5, 21.8. HRMS (ESI): calcd for C_14_H_17_O_3_^+^ [M + H^+^] = 233.1172, found = 233.1173. IR (ATR): ν̃ (cm^–1^) = 2930, 2859, 1763, 1740, 1409, 1367, 1224, 1144. Minor diastereoisomer: ^1^H NMR (500 MHz, CDCl_3_) δ (ppm) = 6.59 (d, *J* = 7.3 Hz, 1H), 6.47 (d, *J* = 7.3 Hz, 1H), 4.00 (ddt, *J* = 11.6, 4.7, 1.6 Hz, 1H), 3.65 (td, *J* = 12.0, 2.4 Hz, 1H), 2.57 (dd, *J* = 19.6, 9.7 Hz, 1H), 2.46–2.36 (m, 3H), 2.22 (dd, *J* = 14.7, 6.0 Hz, 1H), 1.87 (dt, *J* = 12.7, 9.9 Hz, 1H), 1.81–1.70 (m, 3H), 1.62–1.53 (m, 1H), 1.36–1.24 (m, 2H). ^13^C{^1^H} NMR (126 MHz, CDCl_3_) δ (ppm) = 212.6, 210.8, 135.0, 129.5, 83.0, 64.7, 58.2, 55.7, 40.5, 36.4, 29.0, 25.5, 24.0, 22.7. HRMS (ESI): calcd for C_14_H_17_O_3_^+^ [M + H^+^] = 233.1172, found = 233.1173. IR (ATR): ν̃ (cm^–1^) = 2926, 2855, 1764, 1742, 1450, 1289, 1126, 1098.

## Data Availability

The data underlying this study are available in the published article and its Supporting Information.

## Supporting Information Available

^1^H and ^13^C{^1^H} NMR spectra for all compounds, configuration assignment by NOESY studies, UV/vis spectra of compounds **9** and **18**, and crystallographic data for compounds **18** and **39** (PDF)
FAIR data, including the primary NMR FID files, for compounds **5**–**15**, **18**–**35**, and **39**–**44** (ZIP)
