# Supplementary material for: Intramolecular ortho Photocycloaddition of 4-Substituted 7-(4′-Alkenyloxy)-1-indanones and Ensuing Reaction Cascades
Source: J Org Chem. 2025 Mar 11;90(11):4099–107. doi: 10.1021/acs.joc.5c00171 (PMC11934143; doi:10.1021/acs.joc.5c00171)
Supplement: Supplementary file 1 — jo5c00171_si_001.pdf [file jo5c00171_si_001.pdf]

Supporting Information

Intramolecular *ortho* Photocycloaddition of 4-Substituted 7-(4'-Alkenyloxy)-1-indanones and Ensuing Reaction Cascades

Audrey Gilbert, Julian Zuber, Thorsten Bach*

Department Chemie and Catalysis Research Center (CRC), Technische Universität München, Lichtenbergstr. 4, 85747 Garching, Germany

*thorsten.bach@ch.tum.de

Table of contents

1. **Preparation of the starting materials and characterization data S2**

2. **Optimization studies S13**

3. **NOESY experiments S16**

4. **X-ray crystallographic details S19**

5. **UV-vis spectra of compounds 9 and 18 .... S35**

6. **Emission spectra of the light source S39**

7. **NMR Spectra S40**

1. Preparation of the starting materials and characterization data

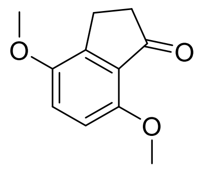

4,7-Dimethoxy-2,3-dihydro-1*H*-inden-1-one (5). Following the procedure described by Lee and coworkers,[1] to a flask under argon were successively added 3-(2,5-dimethoxyphenyl)propanoic acid (4.3 g, 20.64 mmol, 1 equiv.) and 2 drops of dry DMF at room temperature. Thionyl chloride (3.45 mL, 47.47 mmol, 2.3 equiv.) was added and the reaction was stirred for 2 hours at room temperature. The thionyl chloride was next removed under reduced pressure. The crude mixture was next added dropwise to a flask under argon containing a mixture of $AlCl_3$ (5.5 g, 41.28 mmol, 2 equiv.) and CH_2Cl_2 (26 mL, 0.8 M) at 0 °C. The reaction was stirred for 10 min at this temperature before it was poured in a 1M HCl solution at 0 °C. The crude was then extracted three times with dichloromethane. The organic phase was washed once with water, once with a saturated $NaHCO_3$ solution, dried over Na_2SO_4, filtered and concentrated *in vacuo*. The crude mixture was purified by trituration in Et_2O to yield the title compound as an orange solid (3.3 g, 17.32 mmol, 84%). Analytical data were identical to those previsouly reported.[1]

^{1}H NMR (500 MHz, $CDCl_3$) δ (ppm) = 6.98 (d, J = 8.7 Hz, 1H), 6.73 (d, J = 8.7 Hz, 1H), 3.90 (s, 3H), 3.85 (s, 3H), 3.03 – 2.91 (m, 2H), 2.70 – 2.63 (m, 2H).

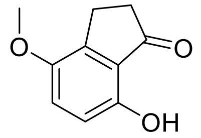

7-Hydroxy-4-methoxy-2,3-dihydro-1*H*-inden-1-one (6). To a flame-dried flask under argon were successively added magnesium powder (1.46 g, 60.07 mmol), Et_2O (120 mL) and I_2 (15.2 g, 60.07 mmol) at 0 °C. The solution was stirred for 2 hours at 0 °C and then 2 hours at room temperature. In another flame-dried flask under argon were added 4,7-dimethoxy-2,3-dihydro-1*H*-inden-1-one **5** (4.11 g, 21.40 mmol, 1 equiv.) and THF (107 mL, 0.5 M). The MgI_2 solution (107 mL, 53.50 mmol, 2.5 equiv.) was transferred to the reaction and the reaction was stirred at 60 °C in an oil-bath for 16 hours. The reaction was then cooled to room temperature and CH_2Cl_2 was added. The organic phase was washed with a 1 M HCl solution, dried over Na_2SO_4, filtered and concentrated *in vacuo*. The crude product was purified over flash chromatography on silica gel using hexane/EtOAc (8:2) as the eluent to afford the title compound as a yellowish solid (3.51 g, 19.7 mmol, 92%). Analytical data were identical as previously reported.[1]

^{1}H NMR (500 MHz, $CDCl_3$) δ (ppm) = 8.54 (s, 1H), 7.00 (d, J = 8.6 Hz, 1H), 6.73 (dt, J = 8.7, 0.9 Hz, 1H), 3.84 (s, 4H), 3.10 – 2.96 (m, 3H), 2.83 – 2.58 (m, 2H).

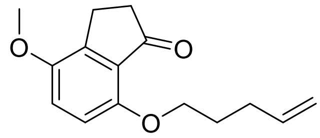

4-Methoxy-7-(pent-4-en-1-yloxy)-2,3-dihydro-1*H*-inden-1-one (7). To a flame-dried flask under argon were successively added 7-hydroxy-4-methoxy-2,3-dihydro-1*H*-inden-1-one (**6**) (629.4 mg, 3.53 mmol, 1 equiv.), dry DMF (3.5 mL, 1 M), 5-bromo-1-pentene (0.63 mL, 5.30 mmol, 1.5 equiv.) and Cs_2CO_3 (1.38 g, 4.24 mmol, 1.2 equiv.) at room temperature. The reaction was stirred for 16 hours at room temperature before it was diluted with $NH_4Cl_{(sat.)}$ and CH_2Cl_2. The mixture was extracted three times with CH_2Cl_2, and the organic phase was next washed once with water and once with brine. The organic phase was then dried over Na_2SO_4, filtered and concentrated *in vacuo*. The crude product was purified over flash chromatography on silica gel using

[1] Kim, Y. Y.; Pyun, Y. M.; Jeon, B. K.; Lee, D.-H. *Bull. Korean Chem. Soc.* **2018**, *39*, 597-598.

hexane/EtOAc (8:2) as the eluent to afford the title compound as a yellowish solid (812.3 mg, 3.30 mmol, 93%).

^{1}H NMR (500 MHz, $CDCl_3$) δ (ppm) = 6.95 (d, *J* = 8.7 Hz, 1H), 6.72 (d, *J* = 8.7 Hz, 1H), 5.86 (ddt, *J* = 16.9, 10.2, 6.6 Hz, 1H), 5.06 (dq, *J* = 17.1, 1.7 Hz, 1H), 4.98 (dd, *J* = 10.3, 1.7 Hz, 1H), 4.04 (t, *J* = 6.6 Hz, 2H), 3.84 (s, 3H), 2.99 – 2.93 (m, 2H), 2.67 – 2.60 (m, 2H), 2.27 (q, *J* = 7.3 Hz, 2H), 1.95 (p, *J* = 6.9 Hz, 2H). ^{13}C{^{1}H} NMR (126 MHz, $CDCl_3$) δ (ppm) = 204.9, 151.3, 150.5, 145.9, 138.0, 126.8, 116.6, 115.3, 111.1, 68.4, 55.9, 36.9, 30.1, 28.2, 22.3. HRMS (ESI): calcd for $C_{15}H_{19}O_3^+$ [M + H^+] = 247.1329, found = 247.1327. IR (ATR): $\tilde{\nu}$ (cm^{-1}) = 2954, 1698, 1593, 1494, 1262, 1101, 1007, 830.

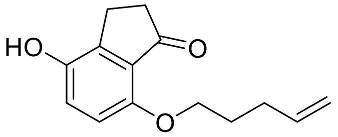

4-Hydroxy-7-(pent-4-en-1-yloxy)-2,3-dihydro-1*H*-inden-1-one (8). Sodium thiomethoxide (140 mg, 1.99 mmol, 2 equiv.) was added to a flame-dried flask under argon in the glove box. The flask was sealed and removed from the glove box. An argon flux was added to the flask before a solution of 4-methoxy-7-(pent-4-en-1-yloxy)-2,3-dihydro-1*H*-inden-1-one (**7**) (245 mg, 1.00 mmol, 1 equiv.) in dry DMF (3.3 mL, 0.3 M) was added to the mixture. The reaction was heated at 150 °C in an oil-bath and stirred at this temperature for one hour. The reaction was next cooled to room temperature and poured in $NH_4Cl_{(sat.)}$. The mixture was extracted three times with EtOAc, and the organic phase was next washed with brine. The organic phase was then dried over Na_2SO_4, filtered and concentrated *in vacuo*. The crude product was purified over flash chromatography on silica gel using hexane/EtOAc (6:4) as the eluent to afford the title compound as a yellowish solid (148.5 mg, 0.64 mmol, 64%).

^{1}H NMR (500 MHz, $CDCl_3$) δ (ppm) = 6.97 (d, *J* = 8.6 Hz, 1H), 6.67 (d, *J* = 8.6 Hz, 1H), 5.85 (ddt, *J* = 16.9, 10.2, 6.6 Hz, 1H), 5.05 (dq, *J* = 17.1, 1.7 Hz, 1H + OH), 5.01 – 4.95 (m, 2H), 4.04 (t, *J* = 6.6 Hz, 2H), 3.03 – 2.95 (m, 2H), 2.70 – 2.66 (m, 2H), 2.30 – 2.23 (m, 2H), 1.98 – 1.90 (m, 2H). ^{13}C{^{1}H} NMR (126 MHz, $CDCl_3$) δ (ppm) = 204.7, 151.8, 146.4, 143.1, 138.0, 126.5, 121.9, 115.3, 111.7, 68.5, 36.8, 30.1, 28.2, 22.0. HRMS (ESI): calcd for $C_{14}H_{17}O_3^+$ [M + H^+] = 233.1172, found = 233.1169. IR (ATR): $\tilde{\nu}$ (cm^{-1}) = 3191, 2669, 1670, 1594, 1503, 1459, 1275, 1052.

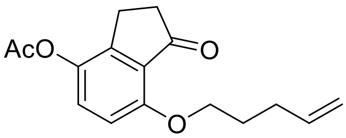

1-Oxo-7-(pent-4-en-1-yloxy)-2,3-dihydro-1*H*-inden-4-yl acetate (9). To a flame-dried flask under argon were successively added 4-hydroxy-7-(pent-4-en-1-yloxy)-2,3-dihydro-1*H*-inden-1-one (**8**) (50.0 mg, 0.22 mmol, 1 equiv.) and dry CH_2Cl_2 (2.2 mL, 0.1 M) at 0 °C. Pyridine (0.17 mL, 1.3 M) and 4-dimethylaminopyridine (DMAP) (3.90 mg, 0.03 mmol, 15 mol%) were added to the reaction before the dropwise addition of acetic anhydride (86 µL, 2.5 M). The reaction was warmed to room temperature and stirred for 24 hours. The mixture was next quenched with water and the organic phase was washed twice with a 1M HCl solution in water and then once with brine. The organic phase was dried over Na_2SO_4, filtered and concentrated *in vacuo*. The crude mixture was purified over flash chromatography on silica gel using hexane/EtOAc (8:2) as the eluent to afford the title compound as a colorless oil (54.1 mg, 0.20 mmol, 92%).

UV/Vis (MeOH): λ_{max} (ε) = 320 nm (3980 M^{-1} cm^{-1}). ^{1}H NMR (500 MHz, $CDCl_3$) δ (ppm) = 7.21 (d, *J* = 8.6 Hz, 1H), 6.77 (d, *J* = 8.7 Hz, 1H), 5.85 (ddt, *J* = 16.9, 10.0, 6.6 Hz, 1H), 5.06 (dq, *J* = 17.1, 1.7 Hz, 1H), 5.01 – 4.96 (m, 1H), 4.09 (t, *J* = 6.6 Hz, 2H), 2.93 – 2.89 (m, 2H), 2.67 – 2.62 (m, 2H), 2.33 (s, 3H), 2.31 – 2.24 (m, 2H), 1.97 (p, *J* = 6.8 Hz, 2H). ^{13}C{^{1}H} NMR (126 MHz, $CDCl_3$) δ (ppm) = 203.5, 169.3, 155.5, 148.8, 141.0, 137.8, 128.9, 126.5, 115.5, 111.1, 68.3, 36.6, 30.0, 28.0,

22.4, 20.9. HRMS (ESI): calcd for $C_{16}H_{19}O_4^+$ [M + H$^+$] = 275.1278, found = 275.1275. IR (ATR): $\tilde{\nu}$ (cm^{-1}) = 3344, 1753, 1690, 1596, 1487, 1210, 1144, 829.

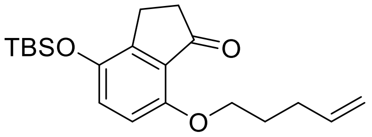

4-((*Tert*-butyldimethylsilyl)oxy)-7-(pent-4-en-1-yloxy)-2,3-dihydro-1*H*-inden-1-one (10). To a flame-dried flask under argon were successively added 4-hydroxy-7-(pent-4-en-1-yloxy)-2,3-dihydro-1*H*-inden-1-one (**8**) (185 mg, 0.80 mmol, 1 equiv.), dry EtOAc (4.3 mL, 0.19 M), imidazole (147 mg, 2.15 mmol, 2.7 equiv.) and TBSCl (143 mg, 0.95 mmol, 1.2 equiv.) at room temperature. The reaction was stirred for 16 hours before it was quenched with brine. The crude mixture was extracted three times with EtOAc and the organic phases were combined, dried over Na_2SO_4, filtered and concentrated *in vacuo*. The crude product was purified over flash chromatography on silica gel using hexane/CH_2Cl_2/Et_2O (7:2.5:0.5) as the eluent to afford the title compound as a white solid (195 mg, 0.56 mmol, 71%).

^{1}H NMR (500 MHz, $CDCl_3$) δ (ppm) = 6.91 (d, *J* = 8.6 Hz, 1H), 6.64 (d, *J* = 8.6 Hz, 1H), 5.85 (ddt, *J* = 16.9, 10.2, 6.6 Hz, 1H), 5.09 – 5.02 (m, 1H), 5.01 – 4.95 (m, 1H), 4.04 (t, *J* = 6.6 Hz, 2H), 2.98 – 2.91 (m, 2H), 2.66 – 2.58 (m, 2H), 2.27 (q, *J* = 7.1 Hz, 2H), 1.94 (p, *J* = 6.8 Hz, 2H), 1.01 (s, 9H), 0.20 (s, 6H). ^{13}C{^{1}H} NMR (126 MHz, $CDCl_3$) δ (ppm) = 204.7, 151.9, 147.7, 146.3, 138.0, 126.7, 125.4, 115.3, 111.2, 68.3, 36.9, 30.1, 28.2, 25.8, 22.8, 18.3, -4.1. HRMS (ESI): calcd for $C_{20}H_{31}O_3Si^+$ [M + H$^+$] = 347.2037, found = 347.2034. IR (ATR): $\tilde{\nu}$ (cm^{-1}) = 2930, 1711, 1589, 1492, 1278, 1231, 998, 731.

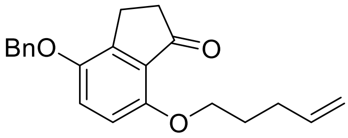

4-(Benzyloxy)-7-(pent-4-en-1-yloxy)-2,3-dihydro-1*H*-inden-1-one (11). To a flame-dried flask under argon were successively added 4-hydroxy-7-(pent-4-en-1-yloxy)-2,3-dihydro-1*H*-inden-1-one (**8**) (50.0 mg, 0.22 mmol, 1 equiv.), dry DMF (0.9 mL, 0.25 M), K_2CO_3 (44.6 mg, 0.32 mmol, 1.5 equiv.) and BnBr (38 μL, 0.32 mmol, 1.5 equiv.) at room temperature. The reaction was stirred for 16 hours before it was quenched with $NaHCO_{3(sat)}$. The crude mixture was extracted three times with EtOAc and the organic phases were combined and washed once with water and once with brine. The organic phase was dried over Na_2SO_4, filtered and concentrated *in vacuo*. The crude product was purified over flash chromatography on silica gel using hexane/EtOAc (9:1) as the eluent to afford the title compound as a yellowish solid (59.5 mg, 0.18 mmol, 86%).

^{1}H NMR (500 MHz, $CDCl_3$) δ (ppm) = 7.44 – 7.37 (m, 4H), 7.33 (tt, *J* = 6.3, 1.6 Hz, 1H), 7.00 (d, *J* = 8.7 Hz, 1H), 6.69 (d, *J* = 8.7 Hz, 1H), 5.86 (ddt, *J* = 16.9, 10.2, 6.6 Hz, 1H), 5.10 – 5.03 (m, 3H), 5.01 – 4.97 (m, 1H), 4.03 (t, *J* = 6.6 Hz, 2H), 3.04 – 2.97 (m, 2H), 2.67 – 2.58 (m, 2H), 2.31 – 2.24 (m, 2H), 1.95 (p, *J* = 6.8 Hz, 2H). ^{13}C{^{1}H} NMR (126 MHz, $CDCl_3$) δ (ppm) = 204.6, 151.5, 149.4, 146.4, 137.9, 137.0, 128.7, 128.1, 127.3, 126.7, 118.5, 115.2, 111.0, 70.7, 68.3, 36.8, 30.0, 28.1, 22.4. HRMS (ESI): calcd for $C_{21}H_{23}O_3^+$ [M + H$^+$] = 323.1642, found = 323.1639. IR (ATR): $\tilde{\nu}$ (cm^{-1}) = 2925, 1706, 1595, 1492, 1229, 1262, 1018, 911.

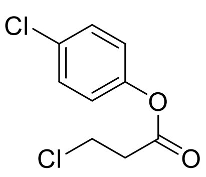

4-Chlorophenyl 3-chloropropanoate (S1). Following the procedure described by Nishi and coworkers[2], to a flamed-dried flask under argon were successively added 4-chlorophenol (500 mg, 3.89 mmol, 1 equiv.), CH_2Cl_2 (16 mL, 0.25 M), pyridine (0.63 mL, 7.78 mmol, 2 equiv.) and 3-chloropropanoyl chloride (0.45 mL, 4.66 mmol, 1.2 equiv.) at room temperature. The mixture was stirred for 2 hours before it was quench by the addition of water. The mixture was extracted three times with CH_2Cl_2 and the organic phases were combined, dried over Na_2SO_4, filtered and concentrated *in vacuo*. The crude product was purified over flash chromatography on silica gel using hexane/EtOAc (9:1) as the eluent to afford the title compound as a yellow oil (539 mg, 2.46 mmol, 63%). Analytical data were identical as previously reported.[2]

^{1}H NMR (500 MHz, $CDCl_3$) δ (ppm) = 7.38 – 7.31 (m, 1H), 7.08 – 7.04 (m, 1H), 3.86 (t, *J* = 6.6 Hz, 1H), 3.04 (t, *J* = 6.6 Hz, 1H).

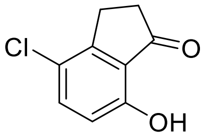

4-Chloro-7-hydroxy-2,3-dihydro-1*H*-inden-1-one (12). To a flame-dried flask under argon were successively added 4-chlorophenyl 3-chloropropanoate (**S1**) (953 mg, 4.35 mmol, 1 equiv.) and $AlCl_3$ (1.74 g, 13.1 mmol, 3 equiv.) at room temperature. The flask was equipped with a gas trap containing a 1M NaOH solution and the reaction was heated at 100 °C in an oil-bath for 3 hours. The reaction temperature was next increased to 190 °C for an additional 3 hours. The reaction mixture was next cooled to room temperature and was quenched with a saturated solution of Rochelle's salt until the gas release ceased. A saturated solution of $NaHCO_3$ and CH_2Cl_2 were added and the mixture was stirred overnight. The crude was next extracted three times with CH_2Cl_2 and the organic phases were combined, dried over Na_2SO_4, filtered and concentrated *in vacuo*. The crude mixture was purified over flash chromatography on silica gel using hexane/EtOAc (9:1) as the eluent to afford the title compound as a yellow solid (779 mg, 4.27 mmol, 52%). Analytical data were identical as previously reported.[2]

^{1}H NMR (500 MHz, $CDCl_3$) δ (ppm) = 8.96 (s, 1H), 7.42 (d, *J* = 8.6 Hz, 1H), 6.75 (dt, *J* = 8.6, 0.8 Hz, 1H), 3.14 – 3.04 (m, 2H), 2.78 – 2.72 (m, 2H).

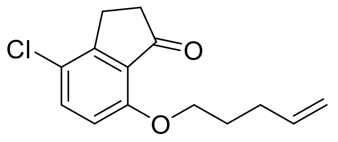

4-Chloro-7-(pent-4-en-1-yloxy)-2,3-dihydro-1*H*-inden-1-one (13). To a flame-dried flask under argon were successively added 4-chloro-7-hydroxy-2,3-dihydro-1*H*-inden-1-one (**12**) (173 mg, 0.94 mmol, 1 equiv.), dry DMF (1 mL, 1 M), 5-bromopent-1-ene (0.17 mL, 1.43 mmol, 1.5 equiv.) and Cs_2CO_3 (369 mg, 1.13 mmol, 1.2 equiv.) at room temperature. The reaction was stirred for 16 hours before it was diluted with $NH_4Cl_{(sat.)}$ and CH_2Cl_2. The reaction was extracted three times with CH_2Cl_2 and the organic phases were combined, dried over Na_2SO_4, filtered and evaporated. The crude product was purified over flash chromatography on silica gel using hexane/EtOAc (9:1) as the eluent to afford the title compound as a yellowish solid (202 mg, 0.81 mmol, 85%).

^{1}H NMR (500 MHz, $CDCl_3$) δ (ppm) = 7.45 (d, *J* = 8.7 Hz, 1H), 6.75 (d, *J* = 8.7 Hz, 1H), 5.85 (ddt, *J* = 16.9, 10.1, 6.6 Hz, 1H), 5.06 (dq, *J* = 17.1, 1.7 Hz, 1H), 5.00 (dq, *J* = 10.1, 1.4 Hz, 1H), 4.09 (t,

[2] Tsukada, T.; Takahashi, M.; Takemoto, T.; Kanno, O.; Yamane, T.; Kawamura, S.; Nishi, T. *Bioorg. Med. Chem. Lett.* **2010**, *20*, 1004-1007.

J = 6.6 Hz, 2H), 3.15 – 2.94 (m, 2H), 2.72 – 2.64 (m, 2H), 2.32 – 2.24 (m, 2H), 2.02 – 1.91 (m, 2H). $^{13}C\{^1H\}$ NMR (126 MHz, $CDCl_3$) δ (ppm) = δ 203.7, 156.4, 154.7, 137.7, 135.6, 127.0, 123.2, 115.6, 111.9, 68.3, 36.7, 30.0, 28.0, 24.9. HRMS (ESI): calcd for $C_{14}H_{16}{}^{35}ClO_2^+$ [M + H$^+$] = 251.0833, found = 251.0831. IR (ATR): $\tilde{\nu}$ (cm^{-1}) = 2929, 1713, 1588, 1463, 1290, 1222, 1191, 809.

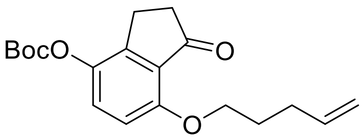

***Tert*-butyl (1-oxo-7-(pent-4-en-1-yloxy)-2,3-dihydro-1*H*-inden-4-yl) carbonate (19).** To a flame-dried flask under argon were successively added 4-hydroxy-7-(pent-4-en-1-yloxy)-2,3-dihydro-1*H*-inden-1-one (**8**) (200 mg, 0.86 mmol, 1 equiv.), dry CH_2Cl_2 (2.2 mL, 0.4 M) 4-dimethylaminopyridine (DMAP) (126 mg, 1.03 mmol, 1.2 equiv.) at 0 °C. Di-*tert*-butyl dicarbonate (Boc_2O) (207 mg, 0.95 mmol, 1.1 mmol) was dissolved in dry THF (0.8 mL) and this solution was added to the reaction mixture dropwise. The reaction was warmed to room temperature and stirred for 16 hours. The mixture was then washed once with a solution of HCl 1 M in water and once with brine. The organic phase was then dried over Na_2SO_4, filtered and concentrated *in vacuo*. The crude product was purified over flash chromatography on silica gel using toluene/EtOAc (95:05) as the eluent to afford the title compound as a yellowish oil (161 mg, 0.48 mmol, 56%).

^1H NMR (500 MHz, $CDCl_3$) δ (ppm) = 7.28 (d, J = 8.7 Hz, 1H), 6.77 (d, J = 8.8 Hz, 1H), 5.85 (ddt, J = 16.9, 10.1, 6.6 Hz, 1H), 5.06 (dq, J = 17.2, 1.7 Hz, 1H), 4.99 (dq, J = 10.2, 1.3 Hz, 1H), 4.09 (t, J = 6.6 Hz, 2H), 3.00 – 2.93 (m, 2H), 2.68 – 2.64 (m, 2H), 2.28 (q, J = 7.5 Hz, 2H), 1.96 (dt, J = 13.7, 6.6 Hz, 2H), 1.56 (s, 9H). $^{13}C\{^1H\}$ NMR (126 MHz, $CDCl_3$) δ (ppm) = 203.5, 155.5, 151.7, 148.8, 141.4, 137.8, 128.8, 126.5, 115.5, 111.1, 84.1, 68.3, 36.6, 30.0, 28.0, 27.8, 22.2. HRMS (ESI): calcd for $C_{19}H_{25}O_5^+$ [M + H$^+$] = 333.1697, found = 333.1691. IR (ATR): $\tilde{\nu}$ (cm^{-1}) = 2980, 1754, 1713, 1603, 1491, 1273, 1141, 1016.0.

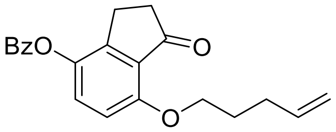

1-Oxo-7-(pent-4-en-1-yloxy)-2,3-dihydro-1*H*-inden-4-yl benzoate (20). To a flame-dried flask under argon were successively added 4-hydroxy-7-(pent-4-en-1-yloxy)-2,3-dihydro-1*H*-inden-1-one (**8**) (200 mg, 0.86 mmol, 1 equiv.), dry CH_2Cl_2 (5.4 mL, 0.16 M), benzoic acid (105 mg, 0.86 mmol, 1 equiv.), N,N'-dicyclohexylcarbodiimide (DCC) (267 mg, 1.29 mmol, 1.5 equiv.) and 4-dimethylaminopyridine (DMAP) (52.6 mg, 0.43 mmol, 0.5 equiv.) at room temperature. The reaction was stirred for 16 hours at room temperature before it was quenched with water. The mixture was extracted three times with CH_2Cl_2, and the organic phase was next washed once with water and once with brine. The organic phase was then dried over Na_2SO_4, filtered and concentrated *in vacuo*. The crude product was purified over flash chromatography on silica gel using hexane/EtOAc (8:2) as the eluent to afford the title compound as a yellowish solid (208 mg, 0.62 mmol, 72%).

^1H NMR (500 MHz, $CDCl_3$) δ (ppm) = 8.21 (dd, J = 8.2, 1.4 Hz, 2H), 7.69 – 7.64 (m, 1H), 7.54 (t, J = 7.7 Hz, 2H), 7.35 (d, J = 8.7 Hz, 1H), 6.83 (d, J = 8.7 Hz, 1H), 5.87 (ddt, J = 16.9, 10.2, 6.6 Hz, 1H), 5.08 (dq, J = 17.2, 1.7 Hz, 1H), 5.01 (dq, J = 10.2, 1.5 Hz, 1H), 4.13 (t, J = 6.6 Hz, 2H), 3.00 – 2.96 (m, 2H), 2.70 – 2.63 (m, 2H), 2.30 (q, J = 7.4 Hz, 2H), 1.99 (p, J = 6.9 Hz, 2H). $^{13}C\{^1H\}$ NMR (126 MHz, $CDCl_3$) δ (ppm) = 203.6, 164.9, 155.6, 149.0, 141.2, 137.8, 134.0, 130.4, 129.10, 129.07, 128.9, 126.6, 115.5, 111.2, 68.3, 36.6, 30.0, 28.1, 22.5. HRMS (ESI): calcd for $C_{21}H_{21}O_4^+$ [M + H$^+$] = 337.1434, found = 337.1429. IR (ATR): $\tilde{\nu}$ (cm^{-1}) = 2940, 1720, 1600, 1488, 1451, 1263, 1216, ,1062.

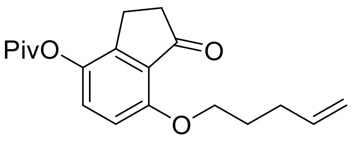

1-Oxo-7-(pent-4-en-1-yloxy)-2,3-dihydro-1*H*-inden-4-yl pivalate (21). To a flame-dried flask under argon were successively added 4-hydroxy-7-(pent-4-en-1-yloxy)-2,3-dihydro-1*H*-inden-1-one (**8**) (200 mg, 0.86 mmol, 1 equiv.), dry THF (2.9 mL, 0.3 M) and Et_3N (0.24 mL, 1.72 mmol, 2 equiv.) at 0 °C. The reaction was stirred for 10 minutes before pivaloyl chloride (PivCl) (0.12 mL, 0.95 mmol, 1.1 equiv.) was added dropwise over 5 minutes. The reaction was warmed to room temperature and stirred for 16 hours before it was quenched with water. The mixture was extracted three times with CH_2Cl_2, and the organic phase was dried over Na_2SO_4, filtered and concentrated *in vacuo*. The crude product was purified over flash chromatography on silica gel using toluene/EtOAc (95:05) as the eluent to afford the title compound as a yellowish oil (178.8 mg, 0.57 mmol, 66%).

^{1}H NMR (500 MHz, $CDCl_3$) δ (ppm) = 7.18 (d, *J* = 8.7 Hz, 1H), 6.77 (d, *J* = 8.7 Hz, 1H), 5.85 (ddt, *J* = 17.0, 10.1, 6.6 Hz, 1H), 5.06 (dq, *J* = 17.0 1.7 Hz, 1H), 5.01 – 4.96 (m, 1H), 4.09 (t, *J* = 6.6 Hz, 2H), 2.90 – 2.87 (m, 2H), 2.66 – 2.62 (m, 2H), 2.28 (q, *J* = 7.5 Hz, 2H), 1.97 (p, *J* = 7.4 Hz, 2H), 1.38 (s, 9H). ^{13}C{^{1}H} NMR (126 MHz, $CDCl_3$) δ (ppm) = 203.6, 176.9, 155.4, 148.8, 141.2, 137.8, 128.9, 126.4, 115.5, 111.1, 68.3, 39.4, 36.6, 30.0, 28.1, 27.3, 22.3. HRMS (ESI): calcd for $C_{19}H_{25}O_4^+$ [M + H$^+$] = 317.1747, found = 317.1745. IR (ATR): $\tilde{\nu}$(cm^{-1}) = 2932, 1749, 1713, 1602, 1490, 1220, 1111, 1020.

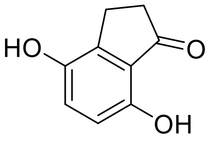

4,7-Dihydroxy-2,3-dihydro-1*H*-inden-1-one (22). To a flask under argon were successively added 4,7-dimethoxy-2,3-dihydro-1*H*-inden-1-one (**5**) (1.70 g, 8.85 mmol, 1 equiv.) and dry CH_2Cl_2 (22 mL, 0.4 M) at – 78 °C. A 1M solution of BBr_3 in CH_2Cl_2 (26.6 mL, 26.6 mmol, 3 equiv.) was next added and the reaction was warmed to room temperature and stirred at this temperature for 2 hours. The reaction was next quenched with water and extracted three times with CH_2Cl_2. The organic phase was dried over Na_2SO_4, filtered and concentrated *in vacuo*. The crude product was purified by flash chromatography over silica gel using hexane/EtOAc (7:3) as the eluent to afford the title compound as a brownish solid (1.13 g, 6.88 mmol, 78%). Analytical data were identical as previously reported.[3]

^{1}H NMR (500 MHz, acetone-*d*$_6$) δ (ppm) = δ 8.63 (s, 1H), 8.23 (s, 1H), 7.03 (d, *J* = 8.5 Hz, 1H), 6.61 (dt, *J* = 8.6, 0.9 Hz, 1H), 3.08 – 3.01 (m, 2H), 2.73 – 2.67 (m, 2H).

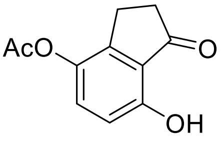

7-Hydroxy-1-oxo-2,3-dihydro-1*H*-inden-4-yl acetate (23). To a flask under argon were successively added 4,7-dihydroxy-2,3-dihydro-1*H*-inden-1-one (**22**) (1.12 g, 6.85 mmol, 1 equiv.) and CH_2Cl_2 (27.4 mL, 0.25 M) at room temperature. Pyridine (0.55 mL, 6.85 mmol, 1 equiv.) and Ac_2O (0.65 mL, 6.85 mmol, 1 equiv.) were next added to the mixture and the reaction was stirred at room temperature for 4 hours. The reaction was then diluted with water and extracted three times with CH_2Cl_2. The organic phase was dried over Na_2SO_4, filtered and concentrated *in vacuo*. The crude product was purified over flash chromatography on silica gel using hexane/EtOAc (8:2) as the eluent to obtain the title compound as a white solid (1.05 g, 5.10 mmol, 74%).

^{1}H NMR (500 MHz, $CDCl_3$) δ (ppm) = 9.00 (s, 1H), 7.20 (d, *J* = 8.7 Hz, 1H), 6.81 (d, *J* = 8.7 Hz, 1H), 3.05 – 2.96 (m, 2H), 2.78 – 2.71 (m, 3H), 2.35 (s, 4H). ^{13}C{^{1}H} NMR (126 MHz, $CDCl_3$) δ

[3] Tsukamoto, H.; Nomura, Y.; Doi, T. *Heterocycles* **2019**, *99*, 549-565.

(ppm) = 209.3, 169.1, 155.4, 145.9, 140.4, 130.7, 123.3, 115.0, 35.8, 23.3, 20.8. HRMS (TOF ESI+): calcd for $C_{11}H_{11}O_4^+$ [M + H$^+$] = 207.0657, found = 207.0684. IR (ATR): $\tilde{\nu}$ (cm^{-1}) = 3344, 1753, 1690, 1487, 1372, 1144, 960, 766.

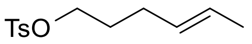

(*E*)-Hex-4-en-1-yl 4-methylbenzenesulfonate (S2). Following the procedure described by Auclair and coworkers[4], to a flame-dried flask under argon were successively added (*E*)-hex-4-en-1-ol (0,50 mL, 4.25 mmol, 1 equiv.), *p*-toluenesulfonyl chloride (TsCl) (891 mg, 4.67 mmol, 1.1 equiv.) and 4-dimethylaminopyridine (DMAP) (51.9 mg, 0.42 mmol, 0.1 equiv.) at room temperature. Dry CH_2Cl_2 (17 mL, 0.25 M) and Et_3N (0.71 mL, 5.10 mmol, 1.2 equiv.) were then added, and the mixture was stirred at room temperature for 3 hours. The mixture was next transferred in an extraction funnel and washed twice with water. The organic phase was dried over Na_2SO_4, filtered and concentrated *in vacuo*. The crude product was purified over flash chromatography on silica gel using hexane/EtOAc (95:5) as the eluent to obtain the title compound as a colorless oil (858 mg, 3.37 mmol, 79%). Analytical data were identical as previously reported.[4]

^{1}H NMR (500 MHz, $CDCl_3$) δ (ppm) = 7.79 (virt. d, *J* = 8.3 Hz, 2H), 7.34 (virt. d, *J* = 8.0 Hz, 2H), 5.39 – 5.30 (m, 1H), 5.30 – 5.21 (m, 1H), 4.02 (t, *J* = 6.4 Hz, 2H), 2.45 (s, 3H), 2.03 – 1.93 (m, 2H), 1.69 (dt, *J* = 7.8, 6.6 Hz, 2H), 1.58 (dd, *J* = 6.1, 1.4 Hz, 3H).

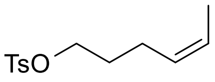

(*Z*)-Hex-4-en-1-yl 4-methylbenzenesulfonate (S3). Following the procedure previously described in the Bach group[5], (*Z*)-hex-4-en-1-ol (0.50 mL, 4.25 mmol, 1 equiv.) and pyridine (10.6 mL, 0.4 M) were added to a flame-dried flask under argon at – 20 °C. *p*-toluenesulfonyl chloride (TsCl) (6.10 g, 31.9 mmol, 7.5 equiv.) was added to the flask and the reaction mixture was stirred at – 20 °C for 16 hours. The reaction was next quenched with HCl 3M in water and extracted three times with Et_2O. The organic phases were combined, washed with HCl 3M in water and then with a saturated $NaHCO_3$ solution in water. The organic phase was then dried over Na_2SO_4, filtered and concentrated *in vacuo*. The crude product was purified over flash chromatography on silica gel using hexane/EtOAc (95:5) as the eluent to obtain the title compound as a colorless oil (344 mg, 1.35 mmol, 32%). Analytical data were identical as previously reported.[5]

^{1}H NMR (500 MHz, $CDCl_3$) δ (ppm) = 7.79 (virt. d, *J* = 8.3 Hz, 2H), 7.34 (virt. d, *J* = 8.0 Hz, 2H), 5.51 – 5.40 (m, 1H), 5.28 – 5.20 (m, 1H), 4.03 (t, *J* = 6.4 Hz, 2H), 2.45 (s, 3H), 2.10 – 2.04 (m, 2H), 1.73 – 1.66 (m, 2H), 1.55 (ddt, *J* = 6.9, 1.9, 0.9 Hz, 3H).

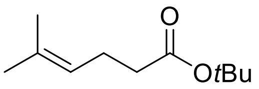

***Tert*-butyl 5-methylhex-4-enoate (S4).** Following the procedure described by Auclair and coworkers[4], to a flame-dried flask under argon were successively added diisopropylamine (1.60 mL, 11.7 mmol, 1.95 equiv.), dry THF (14 mL) and *n*BuLi (1.6 M in hexane, 6.60 mL, 10.5 mmol, 1.75 equiv.) at – 78 °C. The reaction was stirred for 1 hour before a solution of *t*BuOAc (1.50 mL, 11.1 mmol, 1.86 equiv.), dry THF (4 mL) and N,N′-dimethylpropyleneurea (DMPU) (2 mL) was added dropwise to the reaction over 5 minutes. The reaction was stirred for an additional 40 minutes before a solution of 1-bromo-3-methylbut-2-ene (0.70 mL, 6.00 mmol, 1 equiv.) in dry THF (11 mL) was added to the reaction dropwise. The reaction was then allowed to warm to room temperature and stirred for 16 hours. The reaction was next quenched with water and extracted three times with CH_2Cl_2. The organic phases were combined,

[4] Polic, V.; Cheong, K. J.; Hammerer, F.; Auclair, K. *Adv. Synth. Catal.* **2017**, *359*, 1983-3989.
[5] Næsbord, L.; Jandl, C.; Zech, A.; Bach, T. *Angew. Chem. Int. Ed.* **2020**, *59*, 5656-5659.

washed with brine, dried over Na_2SO_4, filtered and concentrated *in vacuo*. The crude product was purified over flash chromatography on silica gel to yield *tert*-butyl 5-methylhex-4-enoate as a colorless oil (580 mg, 3.15 mmol, 52%). Analytical data were identical as previously reported.[4]

^{1}H NMR (500 MHz, $CDCl_3$) δ (ppm) = 5.11 – 5.05 (m, 1H), 2.28 – 2.19 (m, 4H), 1.68 (s, 3H), 1.62 (s, 3H), 1.44 (s, 9H).

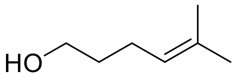

5-Methylhex-4-en-1-ol (S5). Following the procedure described by Auclair and coworkers[Error! Bookmark not defined.], to a flame-dried flask under argon were successively added $LiAlH_4$ (170 mg, 4.48 mmol, 1.5 equiv.), dry THF (14 mL) and *tert*-butyl 5-methylhex-4-enoate (**S4**) (550 mg, 2.99 mmol, 1 equiv.) at 0 °C. The reaction was then allowed to warm to room temperature and stirred for 16 hours. The reaction was next quenched with water (0.2 mL), NaOH 1M in water (0.2 mL) and then water again (0.5 mL). The mixture was stirred at 0 °C for 1 hour. The solid was filtered-off and the filtrate was dried over Na_2SO_4, filtered and concentrated *in vacuo*. The crude product was purified over flash chromatography on silica gel to yield the title compound as a colorless oil (65.3 mg, 0.57 mmol, 19%). Analytical data were identical as previously reported.[Error! Bookmark not defined.]

^{1}H NMR (500 MHz, $CDCl_3$) δ (ppm) = 5.13 (ddt, *J* = 7.3, 5.8, 1.5 Hz, 1H), 3.65 (t, *J* = 6.5 Hz, 2H), 2.07 (q, *J* = 7.2 Hz, 2H), 1.69 (d, *J* = 1.4 Hz, 3H), 1.65 – 1.57 (m, 5H).

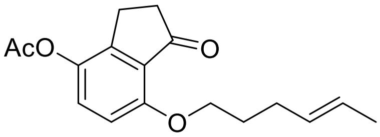

(*E*)-7-(Hex-4-en-1-yloxy)-1-oxo-2,3-dihydro-1*H*-inden-4-yl acetate ((*E*)-24). To a flame-dried flask under argon were successively added 7-hydroxy-1-oxo-2,3-dihydro-1*H*-inden-4-yl acetate (**23**) (200 mg, 0.97 mmol, 1 equiv.), dry DMF (3.9 mL, 0.25 M), (*E*)-hex-4-en-1-yl 4-methylbenzenesulfonate (370 mg, 1.45 mmol, 1.5 equiv.) and Cs_2CO_3 (379 mg, 1.16 mmol, 1.2 equiv.) at room temperature. The reaction was heated to 50 °C in an oil-bath and stirred for 16 hours, before it was cooled down to room temperature and diluted with $NH_4Cl_{(sat.)}$ and CH_2Cl_2. The mixture was extracted three times with CH_2Cl_2, and the organic phase was next washed once with water and once with brine. The organic phase was then dried over Na_2SO_4, filtered and concentrated *in vacuo*. The crude product was purified over flash chromatography on silica gel using hexane/EtOAc (75:25) as the eluent to afford the title compound as a yellowish oil (224 mg, 0.78 mmol, 80%).

^{1}H NMR (500 MHz, $CDCl_3$) δ (ppm) = 7.20 (d, *J* = 8.7 Hz, 1H), 6.77 (d, *J* = 8.7 Hz, 1H), 5.57 – 5.33 (m, 2H), 4.07 (t, *J* = 6.6 Hz, 2H), 2.93 – 2.86 (m, 2H), 2.68 – 2.61 (m, 2H), 2.32 (s, 3H), 2.21 – 2.14 (m, 2H), 1.91 (p, *J* = 6.7 Hz, 2H), 1.63 (dd, *J* = 4.9, 1.5 Hz, 3H). ^{13}C{^{1}H} NMR (126 MHz, $CDCl_3$) δ (ppm) = 203.5, 169.3, 155.6, 148.7, 140.9, 130.2, 128.9, 126.4, 126.0, 111.1, 68.4, 36.6, 28.8, 28.7, 22.4, 20.9, 18.1. HRMS (ESI): calcd for $C_{17}H_{21}O_4^+$ [M + H^+] = 289.1434, found = 289.1430. IR (ATR): $\tilde{\nu}$ (cm^{-1}) = 2935, 1760, 1709, 1600, 1489, 1293, 1192, 966.

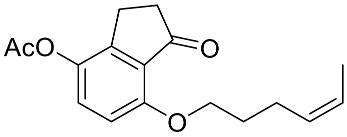

(*Z*)-7-(Hex-4-en-1-yloxy)-1-oxo-2,3-dihydro-1*H*-inden-4-yl acetate ((*Z*)-24). To a flame-dried flask under argon were successively added 7-hydroxy-1-oxo-2,3-dihydro-1*H*-inden-4-yl acetate (**23**) (100 mg, 0.49 mmol, 1 equiv.), dry DMF (1.9 mL, 0.25 M), (*Z*)-hex-4-en-1-yl 4-methylbenzenesulfonate **S3** (185 mg, 0.73 mmol, 1.5 equiv.) and Cs_2CO_3 (190 mg, 0.58 mmol, 1.2 equiv.) at room temperature. The reaction was heated to 50 °C in an oil-bath and stirred for 16 hours,

before it was cooled down to room temperature and diluted with $NH_4Cl_{(sat.)}$ and CH_2Cl_2. The mixture was extracted three times with CH_2Cl_2, and the organic phase was next washed once with water and once with brine. The organic phase was then dried over Na_2SO_4, filtered and concentrated *in vacuo*. The crude product was purified over flash chromatography on silica gel using hexane/EtOAc (75:25) as the eluent to afford the title compound as a yellowish oil (97.1 mg, 0.34 mmol, 70%).

^{1}H NMR (500 MHz, $CDCl_3$) δ (ppm) = 7.20 (d, *J* = 8.7 Hz, 1H), 6.77 (d, *J* = 8.8 Hz, 1H), 5.54 – 5.46 (m, 1H), 5.45 – 5.35 (m, 1H), 4.08 (t, *J* = 6.5 Hz, 2H), 2.96 – 2.86 (m, 2H), 2.66 – 2.63 (m, 2H), 2.33 (s, 3H), 2.28 (q, *J* = 7.5 Hz, 2H), 1.92 (p, *J* = 6.9 Hz, 2H), 1.62 – 1.59 (m, 3H). ^{13}C{^{1}H} NMR (126 MHz, $CDCl_3$) δ (ppm) = 203.5, 169.3, 155.6, 148.8, 140.9, 129.3, 128.9, 126.4, 125.3, 111.0, 68.3, 36.6, 28.7, 23.1, 22.4, 20.9, 12.9. HRMS (ESI): calcd for $C_{17}H_{21}O_4^+$ [M + H$^+$] = 289.1434, found = 289.1431. IR (ATR): $\tilde{\nu}$ (cm^{-1}) = 2935, 1760, 1709, 1601, 1489, 1293, 1192, 1029.

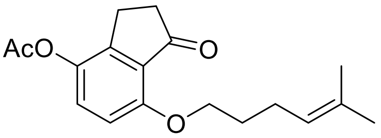

7-((5-Methylhex-4-en-1-yl)oxy)-1-oxo-2,3-dihydro-1*H*-inden-4-yl acetate (25). To a flame-dried flask under argon were successively added triphenylphosphine (335 mg, 1.28 mmol, 1.2 equiv.), dry THF (1.6 mL, 0.66 M), 7-hydroxy-1-oxo-2,3-dihydro-1*H*-inden-4-yl acetate (**23**) (263 mg, 1.28 mmol, 1.2 equiv.) and 5-methylhex-4-en-1-ol (**S5**) (122 mg, 1.06 mmol, 1 equiv.) at room temperature. Diethyl azodicarboxylate (DEAD) (2.2 M in toluene, 0.73 mL, 1.60 mmol, 1.5 equiv.) was then added to the mixture dropwise, and the reaction was stirred for 16 hours at room temperature. The mixture was next concentrated *in vacuo*. The crude product was purified over flash chromatography on silica gel using hexane/EtOAc (8:2) as the eluent to afford the title compound as a yellowish oil (182 mg, 0.60 mmol, 57%).

^{1}H NMR (500 MHz, $CDCl_3$) δ (ppm) = 7.20 (d, *J* = 8.7 Hz, 1H), 6.77 (d, *J* = 8.7 Hz, 1H), 5.14 (tt, *J* = 7.3, 1.5 Hz, 1H), 4.07 (t, *J* = 6.6 Hz, 2H), 2.93 – 2.89 (m, 2H), 2.67 – 2.61 (m, 2H), 2.32 (s, 3H), 2.20 (q, *J* = 7.3 Hz, 2H), 1.89 (p, *J* = 6.9 Hz, 2H), 1.68 (d, *J* = 1.5 Hz, 3H), 1.60 (d, *J* = 1.3 Hz, 3H). ^{13}C{^{1}H} NMR (126 MHz, $CDCl_3$) δ (ppm) = 203.3, 169.3, 155.6, 148.7, 140.9, 132.8, 128.9, 126.5, 123.4, 111.1, 68.4, 36.6, 29.1, 25.8, 24.3, 22.4, 20.9, 17.8. HRMS (ESI): calcd for $C_{18}H_{23}O_4^+$ [M + H$^+$] = 303.1591, found = 303.1589. IR (ATR): $\tilde{\nu}$ (cm^{-1}) = 2928, 1761, 1712, 1601, 1489, 1294, 1197, 1039.

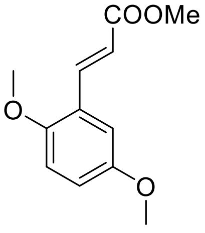

Methyl (*E*)-3-(2,5-dimethoxyphenyl)acrylate (S6). Following the procedure described by Zhang and coworkers,[6] to a flask under argon were successively added (*E*)-3-(2,5-dimethoxyphenyl)acrylic acid (5.00 g, 24.0 mmol, 1 equiv.), dry DMF (5 drops) and $SOCl_2$ (4.00 mL, 55.2 mmol, 2.3 equiv.) at room temperature. The reaction was stirred at this temperature for 2 hours. CH_2Cl_2 (ca. 5 mL) is added to the reaction if the magnetic stirrer gets stuck and stops stirring. After 2 hours, the $SOCl_2$ was removed *in vacuo*. The crude mixture was next put under argon and cooled to 0 °C. CH_2Cl_2 (48 mL, 0.5 M) and MeOH (48 mL, 0.5 M) were added and the reaction was stirred for 1 hour at 0 °C. The crude mixture was next concentrated *in vacuo* to yield the title compound as a yellow oil without further purification (5.34 g, 24.0 mmol, quant.). Analytical data were identical as previously reported.[7]

---

[6] Ge, W.; Hao, X.; Han, F.; Liu, Z.; Wang, T.; Wang, M.; Chen, N.; Ding, Y.; Chen, Y.; Zhang, Q. *Eur. J. Med. Chem.* **2019**, *166*, 445-469.
[7] Babu, B. P.; Meng, X.; Bäckvall, J.-E. *Chem. Eur. J.* **2013**, *19*, 4140-4145.

^{1}H NMR (500 MHz, $CDCl_3$) δ (ppm) = 7.97 (d, *J* = 16.1 Hz, 1H), 7.04 (d, *J* = 2.9 Hz, 1H), 6.91 (dd, *J* = 9.0, 2.9 Hz, 1H), 6.85 (d, *J* = 9.0 Hz, 1H), 6.50 (d, *J* = 16.2 Hz, 1H), 3.84 (s, 3H), 3.80 (s, 3H), 3.79 (s, 3H).

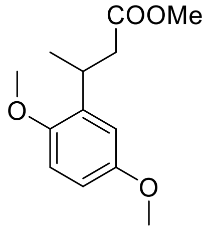

Methyl 3-(2,5-dimethoxyphenyl)butanoate (26). Following the procedure described by Aso and coworkers[8], CuI (4.60 g, 23.9 mmol, 1.2 equiv.) and dry Et_2O (40 mL, 0.5 M) were successively added to a flask under argon at – 40 °C. MeLi (1.6 M in Et_2O, 29.9 mL, 47.8 mmol, 2.4 equiv.) was next added dropwise to the solution and the mixture was stirred for 5 min at – 40 °C. The reaction was next cooled to – 78 °C before TMSCl (3.30 mL, 25.9 mmol, 1.3 equiv.) and a solution of (*E*)-3-(2,5-dimethoxyphenyl)acrylate (**S6**) (4.15 g, 19.9 mmol, 1 equiv.) in Et_2O (40 mL, 0.5 M) were successively added. The mixture was allowed to warm to room temperature and stirred for 5 hours. The reaction was next quenched with a saturated NH_4Cl solution and extracted three times with Et_2O. The organic phases were combined, washed with brine, dried over Na_2SO_4 and concentrated *in vacuo*. The crude product was purified over flash chromatography on silica gel using hexane/EtOAc (9:1) as the eluent to afford the title compound as a colorless oil (3.39 g, 14.2 mmol, 71%).

^{1}H NMR (500 MHz, $CDCl_3$) δ (ppm) = 6.78 (d, *J* = 8.8 Hz, 1H), 6.75 (d, *J* = 3.0 Hz, 1H), 6.70 (dd, *J* = 8.8, 3.1 Hz, 1H), 3.79 (s, 3H), 3.76 (s, 3H), 3.65 (s, 3H), 3.63 – 3.56 (m, 1H), 2.68 (dd, *J* = 15.1, 5.8 Hz, 1H), 2.49 (dd, *J* = 15.1, 9.0 Hz, 1H), 1.26 (d, *J* = 7.0 Hz, 3H). $^{13}C\{^{1}H\}$ NMR (126 MHz, $CDCl_3$) δ (ppm) = 173.4, 153.7, 151.3, 135.3, 113.9, 111.6, 110.9, 56.1, 55.8, 51.6, 41.2, 30.3, 20.1. HRMS (TOF ESI+): calcd for $C_{13}H_{19}O_4^+$ [M + H^+] = 239.1283, found = 239.1301. IR (ATR): $\tilde{\nu}$ (cm^{-1}) = 2952, 2835, 1734, 1498, 1357, 1216, 1050, 800.

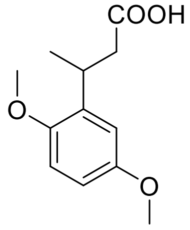

3-(2,5-Dimethoxyphenyl)butanoic acid (27). To a flask under argon were successively added 3-(2,5-dimethoxyphenyl)butanoate (**26**) (3.40 g, 14.2 mmol, 1 equiv.), THF (52 mL) and H_2O (18 ml) at room temperature. LiOH (713 mg, 29.8 mmol, 2.1 equiv.) was next added to the flask and the reaction was heated to 60 °C in an oil-bath and stirred at this temperature for 4 hours. The reaction was then allowed to cool to room temperature and neutralized with a 1 M HCl solution until pH < 7. The reaction was next extracted three times with EtOAc and the combined organic phases were dried over Na_2SO_4, filtered and concentrated *in vacuo*. The title compound was obtained as a yellow oil without further purification (3.15 g, 14.2 mmol, quant.).

^{1}H NMR (500 MHz, $CDCl_3$) δ (ppm) = 6.78 (d, *J* = 8.8 Hz, 1H), 6.76 (d, *J* = 3.0 Hz, 1H), 6.71 (dd, *J* = 8.8, 3.1 Hz, 1H), 3.78 (s, 3H), 3.76 (s, 3H), 3.66 – 3.56 (m, 1H), 2.73 (dd, *J* = 15.5, 5.8 Hz, 1H), 2.52 (dd, *J* = 15.5, 8.9 Hz, 1H), 1.29 (d, *J* = 6.9 Hz, 3H). $^{13}C\{^{1}H\}$ NMR (126 MHz, $CDCl_3$) δ (ppm) = 179.2, 153.7, 151.3, 134.9, 113.9, 111.6, 111.0, 56.0, 55.8, 41.1, 30.1, 20.1. HRMS (TOF ESI+): calcd for $C_{12}H_{17}O_4^+$ [M + H^+] = 225.1127, found = 225.1148. IR (ATR): $\tilde{\nu}$ (cm^{-1}) = 2981, 2921, 1692, 1503, 1441, 1373, 1220, 884.

[8] Aso, K.; Imai, Y.; Yukishige, K.; Ootsu, K.; Akimoto, H. *Chem. Pharm. Bull.* **2001**, *49*, 1280-1287.

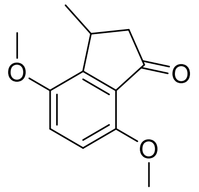

4,7-Dimethoxy-3-methyl-2,3-dihydro-1*H*-inden-1-one (28). To a flask under argon were successively added 3-(2,5-dimethoxyphenyl)butanoic acid (**27**) (100 mg, 0.44 mmol, 1 equiv.) and 2 drops of dry DMF at room temperature. Thionyl chloride (74.0 µL, 1.03 mmol, 2.3 equiv.) was added and the reaction was stirred for 2 hours at room temperature. The thionyl chloride was next removed under reduced pressure. The crude mixture was next added dropwise to a flask under argon containing a mixture of $AlCl_3$ (119 mg, 0.89 mmol, 2 equiv.) and CH_2Cl_2 (0.6 mL, 0.8 M) at 0 °C. The reaction was stirred for 10 min at this temperature before it was poured in a 1M HCl solution at 0 °C. The crude was then extracted three times with dichloromethane. The organic phase was then washed once with water, once with a saturated $NaHCO_3$ solution, dried over Na_2SO_4, filtered and concentrated *in vacuo*. The crude mixture was purified over flash chromatography on silica gel using hexane/EtOAc (6:4) as the eluent to obtain the title compound as an orange solid (67.6 mg, 0.33 mmol, 74%).

^{1}H NMR (500 MHz, $CDCl_3$) δ (ppm) = 6.98 (d, *J* = 8.7 Hz, 1H), 6.71 (d, *J* = 8.7 Hz, 1H), 3.87 (s, 3H), 3.83 (s, 3H), 3.43 (app. pd, *J* = 7.2, 2.3 Hz, 1H), 2.87 (dd, *J* = 18.8, 7.7 Hz, 1H), 2.25 (dd, *J* = 18.8, 2.3 Hz, 1H), 1.33 (d, *J* = 7.0 Hz, 3H). ^{13}C{^{1}H} NMR (126 MHz, $CDCl_3$) δ (ppm) = 204.8, 151.5, 150.7, 150.2, 125.6, 117.4, 109.7, 56.0, 55.8, 46.2, 30.5, 20.7. HRMS (TOF ESI+): calcd for $C_{12}H_{15}O_4^+$ [M + H$^+$] = 207.1021, found = 207.1053. IR (ATR): $\tilde{\nu}$ (cm^{-1}) = 3014, 1712, 1588, 1493, 1265, 1224, 1060, 808.

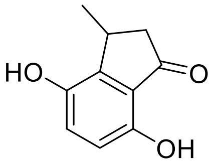

4,7-Dihydroxy-3-methyl-2,3-dihydro-1*H*-inden-1-one (29). To a flask under argon were successively added 4,7-dimethoxy-3-methyl-2,3-dihydro-1*H*-inden-1-one (**28**) (1.00 g, 5.20 mmol, 1 equiv.) and dry CH_2Cl_2 (13 mL, 0.4 M) at – 78 °C. A 1M solution of BBr_3 in CH_2Cl_2 (15.6 mL, 15.6 mmol, 3 equiv.) was next added and the reaction was warmed to room temperature and stirred at this temperature for 2 hours. The reaction was next quenched with water and extracted three times with CH_2Cl_2. The organic phase was dried over Na_2SO_4, filtered and concentrated *in vacuo*. The crude product was purified over flash chromatography on silica gel using hexane/EtOAc (7:3) as the eluent to afford the title compound as a yellow solid (751 mg, 4.22 mmol, 81%).

^{1}H NMR (500 MHz, acetone-*d*$_6$) δ (ppm) = 7.78 (s, 1H), 7.41 (s, 1H), 6.17 (d, *J* = 8.5 Hz, 1H), 5.74 (dd, *J* = 8.5, 0.8 Hz, 1H), 2.72 – 2.63 (m, 1H), 2.09 (dd, *J* = 19.2, 7.5 Hz, 1H), 1.39 (dd, *J* = 19.2, 2.5 Hz, 1H), 0.55 (d, *J* = 7.0 Hz, 3H). ^{13}C{^{1}H} NMR (126 MHz, acetone-*d*$_6$) δ (ppm) = 210.0, 150.9, 148.2, 145.0, 125.0, 123.4, 114.9, 45.6, 32.0, 20.2. HRMS (ESI): calcd for $C_{10}H_{11}O_3^+$ [M + H$^+$] = 179.0703, found = 179.0701. IR (ATR): $\tilde{\nu}$ (cm^{-1}) = 3238, 2969, 1648, 1599, 1470, 1199, 980, 763.

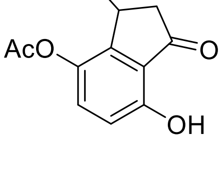

7-Hydroxy-3-methyl-1-oxo-2,3-dihydro-1*H*-inden-4-yl acetate (30). To a flame-dried flask under argon were successively added 4,7-dihydroxy-3-methyl-2,3-dihydro-1*H*-inden-1-one (**29**) (300 mg, 1.68 mmol, 1 equiv.) and CH_2Cl_2 (6.7 mL, 0.25 M) at room temperature. Pyridine (0.14 mL, 1.68 mmol, 1 equiv.) and Ac_2O (0.16 mL, 1.68 mmol, 1 equiv.) were next added to the mixture and the reaction was stirred at room temperature for 4 hours. The reaction was then diluted with water and extracted three times with CH_2Cl_2. The organic phase was dried over Na_2SO_4, filtered and concentrated *in vacuo*. The crude product was purified over flash chromatography on silica gel using hexane/EtOAc (8:2) as the eluent to obtain the title compound as a white solid (296 mg, 1.34 mmol, 80%).

^{1}H NMR (500 MHz, $CDCl_3$) δ (ppm) = 9.05 (s, 1H), 7.18 (d, *J* = 8.7 Hz, 1H), 6.79 (d, *J* = 8.7 Hz, 1H), 3.46 (pd, *J* = 7.2, 2.8 Hz, 1H), 2.98 (dd, *J* = 19.4, 7.6 Hz, 1H), 2.35 – 2.27 (m, 4H), 1.35 (d, *J* = 7.2 Hz, 3H). ^{13}C{^{1}H} NMR (126 MHz, $CDCl_3$) δ (ppm) = 208.8, 169.4, 155.3, 149.5, 140.3, 131.6, 122.7, 115.3, 45.2, 31.8, 21.0, 20.0. HRMS (ESI): calcd for $C_{12}H_{13}O_4^+$ [M + H$^+$] = 221.0808, found = 221.0807. IR (ATR): $\tilde{\nu}$ (cm^{-1}) = 3366, 2982, 1750, 1622, 1487, 1240, 1198, 830.

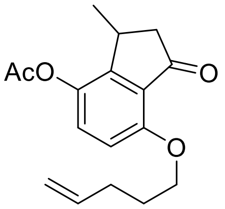

3-Methyl-1-oxo-7-(pent-4-en-1-yloxy)-2,3-dihydro-1*H*-inden-4-yl acetate (31). To a flame-dried flask under argon were successively added 7-hydroxy-3-methyl-1-oxo-2,3-dihydro-1*H*-inden-4-yl acetate (**30**) (247 mg, 1.12 mmol, 1 equiv.), dry DMF (4.5 mL, 1 M), 5-bromo-1-pentene (0.20 mL, 1.68 mmol, 1.5 equiv.) and Cs_2CO_3 (438 mg, 1.34 mmol, 1.2 equiv.) at room temperature. The reaction was heated to 50 °C in an oil-bath and stirred for 16 hours, before it was cooled down to room temperature and diluted with $NH_4Cl_{(sat.)}$ and CH_2Cl_2. The mixture was extracted three times with CH_2Cl_2, and the organic phase was next washed once with water and once with brine. The organic phase was then dried over Na_2SO_4, filtered and concentrated *in vacuo*. The crude product was purified over flash chromatography on silica gel using hexane/EtOAc (8:2) as the eluent to afford the title compound as a yellowish oil (298 mg, 1.03 mmol, 92%).

^{1}H NMR (500 MHz, $CDCl_3$) δ (ppm) = 7.21 (d, *J* = 8.7 Hz, 1H), 6.78 (d, *J* = 8.7 Hz, 1H), 5.85 (ddt, *J* = 16.9, 10.2, 6.6 Hz, 1H), 5.06 (dq, *J* = 17.1, 1.6 Hz, 1H), 4.99 (dq, *J* = 10.1, 1.4 Hz, 1H), 4.09 (tt, *J* = 6.6, 3.2 Hz, 2H), 3.37 (pd, *J* = 7.2, 2.6 Hz, 1H), 2.90 (dd, *J* = 18.8, 7.8 Hz, 1H), 2.34 (s, 3H), 2.33 – 2.13 (m, 3H), 1.97 (p, *J* = 6.9 Hz, 2H), 1.31 (d, *J* = 7.0 Hz, 3H). ^{13}C{^{1}H} NMR (126 MHz, $CDCl_3$) δ (ppm) = 203.0, 169.5, 155.3, 152.5, 140.9, 137.8, 129.8, 125.8, 115.5, 111.4, 68.3, 46.1, 30.5, 30.0, 28.1, 21.1, 20.7. HRMS (ESI): calcd for $C_{17}H_{21}O_4^+$ [M + H$^+$] = 289.1434, found = 289.1432. IR (ATR): $\tilde{\nu}$ (cm^{-1}) = 2933, 1761, 1710, 1488, 1467, 1289, 1193, 1024.

2. Optimization studies

TABLE S1. Optimization of the photoreaction with 4-chloro-1-indanone **13**

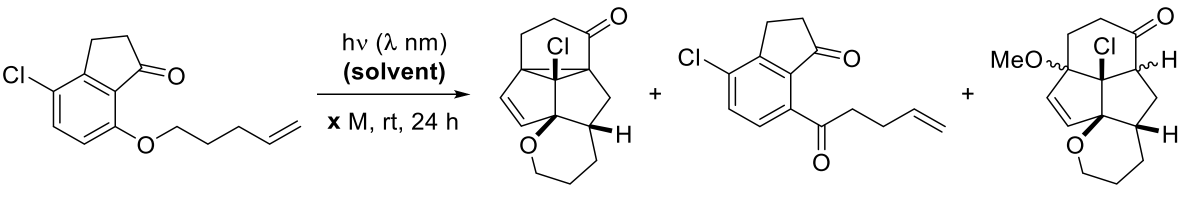

13 **14** **15** **S7**

Entry	λ (nm)	solvent	x M	Conversion (%)[a]	Yield (%)[a]
1	350	MeOH	0.01	100	14%[b] of **S7**
2	300	MeOH	0.01	100	21% of **S7**
3	366	MeOH	0.01	100	23% of **S7**
4	350	MeCN	0.01	100	15%[b] of **14**
5	300	MeCN	0.01	100	Complete degradation
6	366	MeCN	0.01	100	12% of **14**
7	366	MeCN	0.005	98	47%[b] of **14**, 20%[b] of **15**

8	350	DCM	0.01	incomplete	Mixture of **14** and **15**
9	300	DCM	0.01	100	5% of **14**, 20% of **15**
10	366	DCM	0.01	90	20% of **14**, 15% of **15**
11	350	THF	0.01	Incomplete	Complete degradation
12	300	THF	0.01	incomplete	Complete degradation
13	350	toluene	0.01	complete	Complete degradation
14	300	toluene	0.01	complete	Complete degradation
15	350	DMF	0.01	Complete	Complete degradation
16	300	DMF	0.01	Complete	Complete degradation
17	350	DCE	0.01	100	9% of **14**, 21% of **15**
18	350	acetone	0.01	86	4% of **14**, 11% of **15**
19	350	EtOAc	0.01	70	10% of **14**, 35% of **15**

[a]NMR yield evaluated using acetophenone as the internal standard. [b]Yield of isolated product

TABLE S2. Optimization of the photoreaction with 1-oxo-7-(pent-4-en-1-yloxy)-2,3-dihydro-1*H*-inden-4-yl acetate **9**

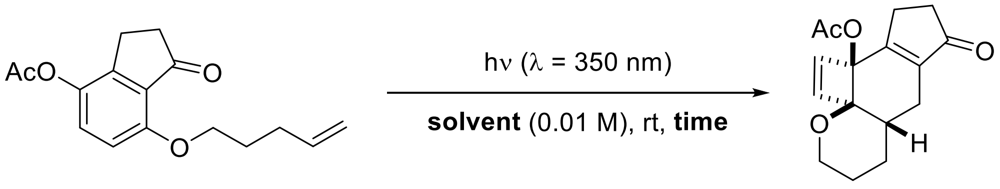

9 **18**

Entry	Solvent	time (h)	Conversion (%)[a]	Yield (%)[a]
1	MeOH	6	100	61%[b]
2	TFE	2	100	60%
3	CH_2Cl_2	6	88	21%
4	MeCN	6	81	9%
5	EtOAc	6	100	0
6	Acetone	6	100	0
7	TFE	6	100	81%[b]
8	EtOH	6	n.d.	12%

[a]NMR yield evaluated using 1,3,5-trimethoxybenzene as the internal standard. [b]Yield of isolated product

TABLE S3. Optimization of the photochemical di-π-methane rearrangement

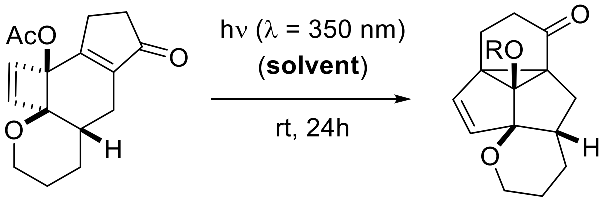

18 **39**, R = Ac

Entry	Solvent	Conversion (%)[a]	Yield (%)[a]
1	DCM	100	0
2	MeCN	100	34%[b]
3	Acetone	100	30%[b]
4	EtOAc	100	63%
5	Toluene	100	84%, 70%[b]
6	THF	100	71%

[a]NMR yield evaluated using 1,3,5-trimethoxybenzene as the internal standard. [b]Yield of isolated product

3. NOESY experiments

In the NOESY-spectrum of compound **34** there are NOE interactions between the proton at the cyclobutene ring **H** and the methyl **H** which supports the relative configuration at the carbon bearing the methyl substituent. Additionally, NOE interactions can be found between **H** and H, also supporting this relative configuration. The key interactions are marked with a circle in the NOESY-spectrum shown below and highlighted with double sided arrows on the depicted compound structure with respective colors.

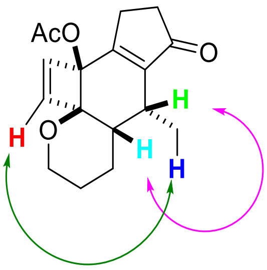

34

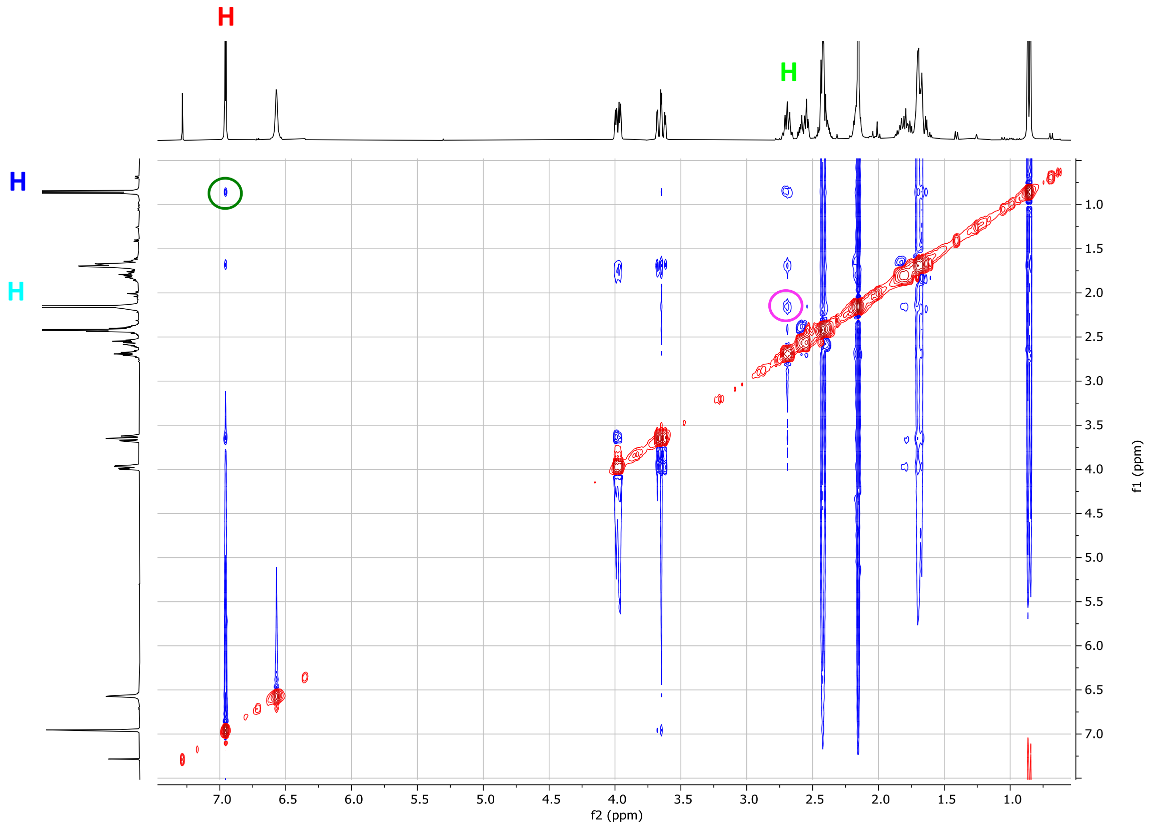

In the NOESY-spectrum of compound **35** there are NOE interactions between the proton at the cyclobutene ring **H** and the methyl **H** which supports the relative configuration of the major diastereoisomer at the carbon bearing the methyl substituent. Additionally, NOE interactions can be found between the acetate protons **H** and H, also supporting this relative configuration of the major diastereoisomer. The key interactions are marked with a circle in the NOESY-spectrum shown below and highlighted with double sided arrows on the depicted compound structure with respective colors.

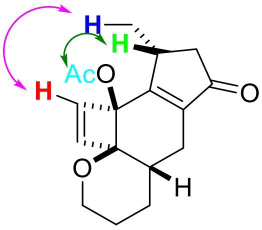

35

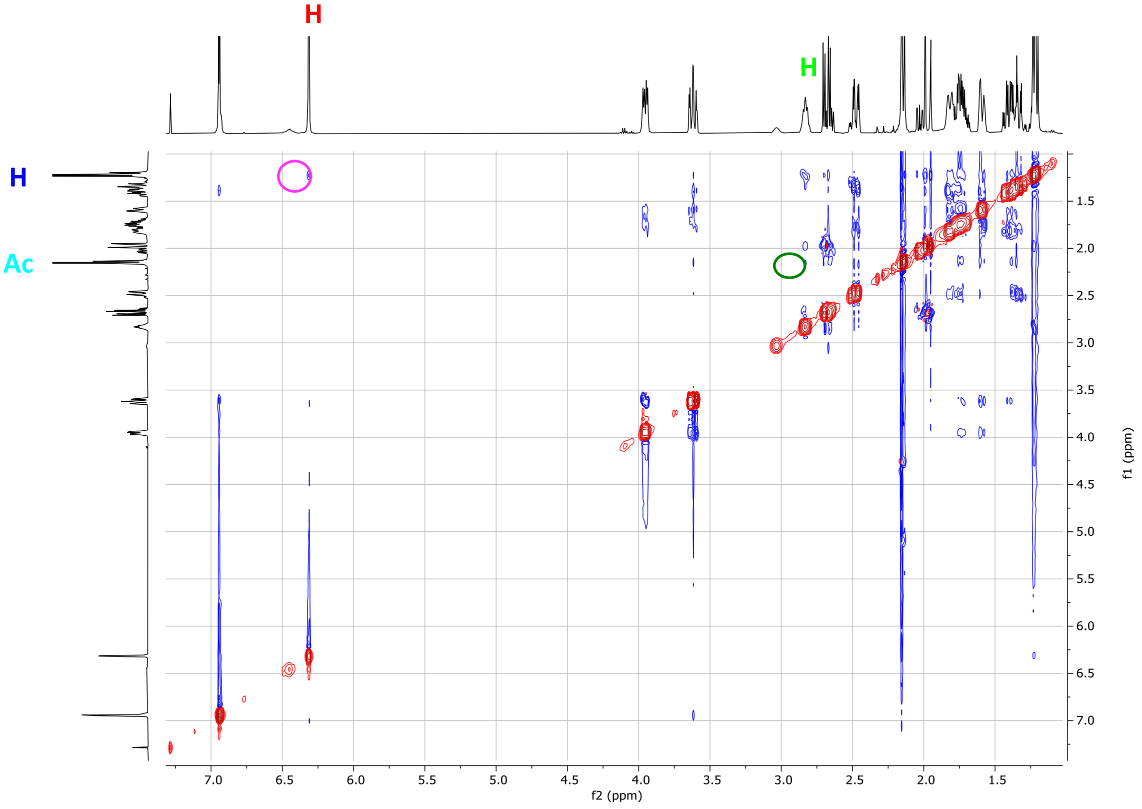

In the NOESY-spectrum of compound **39** there are NOE interactions between the proton at the double bond **H** and the methyl **H** which supports the relative configuration of the major diastereoisomer at the carbon bearing the methyl substituent. The key interaction is marked with a circle in the NOESY-spectrum shown below and highlighted with double sided arrows on the depicted compound structure with respective color.

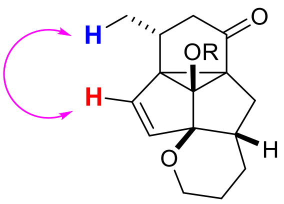

39, R^4 = Ac

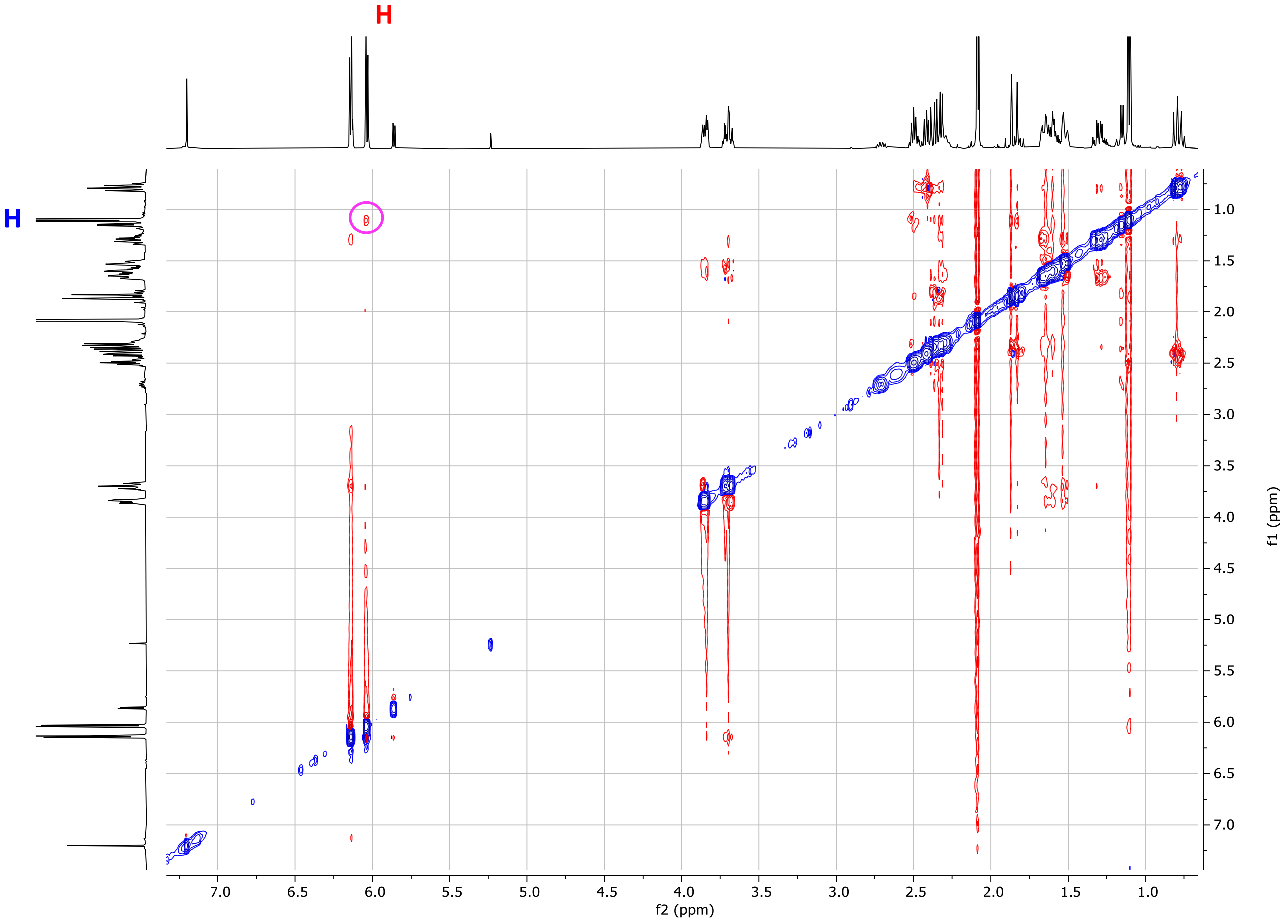

4. X-ray crystallographic details

4.1. SC-XRD structure report for compound 18 (CCDC 2418665)

FIGURE S1: Molecular structure of **18** in the solid-state. ADPs are given at the 50% probability level. Flack parameter of the refinement is arbitrary and therefore, both enantiomers are possible.

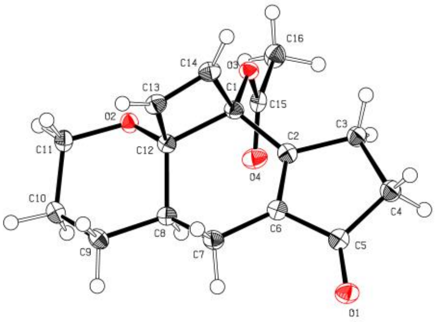

Compound **18** was dissolved in a minimum EtOAc in a 10 mL round-bottom flask. The neck of the flask was blocked with paper towel and the solvent slowly evaporated until formation of the crystal. A colorless, block shaped crystal of $C_{16}H_{18}O_4$ coated with perfluorinated ether and fixed on top of a Kapton micro sampler was used for X-ray crystallographic analysis. The X-ray intensity data were collected at 100(2) K on a Bruker D8 VENTURE three-angle diffractometer with a TXS rotating anode with MoK_α radiation (λ=0.71073 Å) using APEX4.[9] The diffractometer was equipped with a Helios optic monochromator, a Bruker PHOTON III detector, and an Oxford Cryostream low temperature device.

A matrix scan was used to determine the initial lattice parameters. All data were integrated with the Bruker SAINT V8.40B software package using a narrow-frame algorithm and the reflections were corrected for Lorentz and polarisation effects, scan speed, and background.[10] The integration of the data using a orthorhombic unit cell yielded a total of 59384 reflections within a 2θ range [°] of 5.05 to 52.79 (0.80 Å), of which 2714 were independent. Data were corrected for absorption effects including odd and even ordered spherical harmonics by the multi-scan method (SADABS 2016/2).[11] Space group assignment was based upon systematic absences, E statistics, and successful refinement of the structure.

The structure was solved by direct methods using SHELXT and refined by full-matrix least-squares methods against F^2 by minimizing $\Sigma w(F_o^2 - F_c^2)^2$ using SHELXL in conjunction with SHELXLE.[12] All non-hydrogen atoms were refined with anisotropic displacement parameters. Hydrogen atoms were refined isotropically on calculated positions using a riding model with their U_{iso} values constrained to 1.5 times the U_{eq} of their pivot atoms for terminal sp[3] carbon atoms and a C–H distance of 0.98 Å. Non-methyl hydrogen atoms were refined using a riding model with methylene, aromatic,

---

[9] *APEX4 Suite of Crystallographic Software, Version 2021-10.0,* Bruker AXS Inc., Madison, Wisconsin, USA, **2021**.
[10] Bruker, *SAINT, V8.40B*, Bruker AXS Inc., Madison, Wisconsin, USA.
[11] L. Krause, R. Herbst-Irmer, G. M. Sheldrick, D. Stalke, *J. Appl. Cryst.* **2015**, *48*, 3–10, doi:10.1107/S1600576714022985.
[12] a) G. M. Sheldrick, *Acta Cryst.* **2015**, *A71*, 3–8. b) G. M. Sheldrick, *Acta Cryst.* **2015**, *C71*, 3–8. c) C. B. Huebschle, G. M. Sheldrick, B. Dittrich, *J. Appl. Cryst.* **2011**, *44*, 1281–1284.

and other C–H distances of 0.99 Å, 0.95 Å, and 1.00 Å, respectively, and U_{iso} values constrained to 1.2 times the U_{eq} of their pivot atoms.
Neutral atom scattering factors for all atoms and anomalous dispersion corrections for the non-hydrogen atoms were taken from International Tables for Crystallography.[13] Crystallographic data for the structures reported in this paper have been deposited with the Cambridge Crystallographic Data Centre.[14] Supplementary crystallographic data reported in this paper have been deposited with the Cambridge Crystallographic Data Centre (CCDC 2418669) and can be obtained free of charge from The Cambridge Crystallographic Data Centre via www.ccdc.cam.ac.uk/structures.[8] This report and the CIF file were generated using FinalCif.[15]

TABLE S4. Crystal data and structure refinement for compound **18**.

CCDC number	2418665
Empirical formula	$C_{16}H_{18}O_4$
Formula weight	274.30
Temperature [K]	100(2)
Crystal system	orthorhombic
Space group (number)	$Pna2_1$ (33)
a [Å]	13.2284(11)
b [Å]	10.1893(9)
c [Å]	9.8266(7)
α [°]	90
β [°]	90
γ [°]	90
Volume [Å^3]	1324.51(19)
Z	4
ρ_{calc} [gcm^{-3}]	1.376
μ [mm^{-1}]	0.098
F(000)	584
Crystal size [mm^3]	0.047×0.090×0.134
Crystal colour	colorless
Crystal shape	block
Radiation	MoK_α (λ=0.71073 Å)
2θ range [°]	5.05 to 52.79 (0.80 Å)
Index ranges	$-16 \le h \le 16$<br>$-12 \le k \le 12$<br>$-12 \le l \le 12$
Reflections collected	59384
Independent reflections	2714<br>R_{int} = 0.0443<br>R_{sigma} = 0.0142

[13] Ed. E. Prince, *International Tables for Crystallography Volume C, Mathematical, Physical and Chemical Tables*, International Union of Crystallography, Chester, England, **2006**, 500–502; 219–222; 193–199.
[14] C. R. Groom, I. J. Bruno, M. P. Lightfoot, S. C. Ward, *Acta Cryst.* **2016**, *B72*, 171–179.
[15] D. Kratzert, *FinalCif*, *V123*, https://dkratzert.de/finalcif.html.

Completeness to θ = 25.242°	99.8 %
Data / Restraints / Parameters	2714 / 1 / 182
Goodness-of-fit on F^2	1.051
Final R indexes [I≥2σ(I)]	R_1 = 0.0266<br>wR_2 = 0.0688
Final R indexes [all data]	R_1 = 0.0282<br>wR_2 = 0.0703
Largest peak/hole [eÅ^{-3}]	0.22/−0.17
Flack X parameter	-0.2(2)

TABLE S5. Atomic coordinates and Ueq [Å^2] for compound **18**.

**Atom**	***x***	***y***	***z***	U_{eq}
O2	0.54248(9)	0.60669(12)	0.60344(13)	0.0168(3)
O3	0.69398(9)	0.43200(12)	0.66281(13)	0.0163(3)
O4	0.76941(10)	0.63020(13)	0.65564(16)	0.0233(3)
O1	0.88173(11)	0.54496(13)	0.13616(14)	0.0226(3)
C1	0.66703(14)	0.44492(18)	0.52171(18)	0.0159(4)
C2	0.75721(14)	0.44804(17)	0.42963(19)	0.0164(4)
C3	0.85314(14)	0.37184(18)	0.45438(19)	0.0194(4)
H3A	0.838446	0.278059	0.471786	0.023
H3B	0.891106	0.408167	0.532603	0.023
C4	0.91228(16)	0.38936(19)	0.3213(2)	0.0219(4)
H4A	0.981757	0.420657	0.339956	0.026
H4B	0.916243	0.305386	0.270888	0.026
C5	0.85351(14)	0.49085(18)	0.2402(2)	0.0178(4)
C6	0.75684(14)	0.51302(18)	0.31076(19)	0.0168(4)
C7	0.67352(14)	0.60053(19)	0.26238(18)	0.0183(4)
H7A	0.626479	0.549881	0.204230	0.022
H7B	0.702135	0.672790	0.207136	0.022
C8	0.61606(14)	0.65747(18)	0.38395(18)	0.0164(4)
H8	0.663002	0.718009	0.433166	0.020
C9	0.52253(14)	0.73670(19)	0.34327(19)	0.0189(4)
H9A	0.475092	0.680222	0.291870	0.023
H9B	0.542612	0.810815	0.284002	0.023
C10	0.47072(14)	0.78905(19)	0.4717(2)	0.0205(4)
H10A	0.514019	0.856709	0.514475	0.025
H10B	0.405687	0.830642	0.446698	0.025
C11	0.45157(14)	0.67893(19)	0.5727(2)	0.0192(4)
H11A	0.423825	0.716390	0.657818	0.023
H11B	0.400495	0.618142	0.534629	0.023
C12	0.58414(13)	0.55055(17)	0.48340(19)	0.0156(4)
C13	0.52405(15)	0.43238(19)	0.4315(2)	0.0188(4)
H13	0.461953	0.424824	0.383565	0.023
C14	0.58974(14)	0.34404(18)	0.4774(2)	0.0188(4)

H14	0.588578	0.250881	0.481027	0.023
C15	0.74596(14)	0.5336(2)	0.71798(19)	0.0175(4)
C16	0.77103(15)	0.5067(2)	0.8638(2)	0.0231(4)
H16A	0.744913	0.577983	0.920826	0.035
H16B	0.844555	0.500930	0.874499	0.035
H16C	0.740044	0.423582	0.891712	0.035

U_{eq} is defined as 1/3 of the trace of the orthogonalized U_{ij} tensor.

TABLE S6. Anisotropic displacement parameters (Å^2) for compound **18**. The anisotropic displacement factor exponent takes the form: $-2\pi^2[h^2(a^*)^2U_{11} + k^2(b^*)^2U_{22} + \ldots + 2hka^*b^*U_{12}]$

Atom	U_{11}	U_{22}	U_{33}	U_{23}	U_{13}	U_{12}
O2	0.0162(6)	0.0191(6)	0.0152(6)	0.0003(5)	−0.0007(5)	0.0025(5)
O3	0.0167(6)	0.0175(6)	0.0148(6)	0.0017(5)	−0.0011(5)	−0.0009(5)
O4	0.0246(7)	0.0230(7)	0.0223(7)	0.0008(6)	−0.0010(6)	−0.0068(5)
O1	0.0271(7)	0.0249(7)	0.0158(7)	0.0017(6)	0.0024(6)	−0.0005(5)
C1	0.0169(8)	0.0156(9)	0.0153(9)	0.0009(7)	−0.0021(7)	−0.0010(6)
C2	0.0176(8)	0.0145(8)	0.0170(9)	−0.0027(7)	−0.0022(7)	−0.0010(7)
C3	0.0196(9)	0.0207(9)	0.0180(9)	0.0009(7)	0.0021(7)	0.0027(7)
C4	0.0223(9)	0.0231(10)	0.0201(10)	0.0009(8)	0.0028(8)	0.0033(8)
C5	0.0197(9)	0.0177(9)	0.0161(9)	−0.0040(7)	−0.0003(7)	−0.0008(7)
C6	0.0185(9)	0.0171(9)	0.0147(9)	−0.0023(7)	−0.0015(7)	−0.0009(7)
C7	0.0205(9)	0.0206(9)	0.0138(9)	0.0021(7)	−0.0002(7)	0.0010(7)
C8	0.0176(8)	0.0161(8)	0.0155(9)	0.0001(7)	−0.0004(7)	−0.0010(7)
C9	0.0201(9)	0.0195(9)	0.0171(9)	0.0021(7)	−0.0038(7)	0.0020(7)
C10	0.0195(8)	0.0196(9)	0.0224(10)	0.0010(8)	−0.0014(7)	0.0033(7)
C11	0.0159(8)	0.0214(9)	0.0204(9)	−0.0003(7)	−0.0003(7)	0.0022(7)
C12	0.0162(8)	0.0159(8)	0.0146(8)	−0.0006(7)	−0.0014(7)	−0.0004(6)
C13	0.0184(8)	0.0207(9)	0.0173(9)	−0.0005(7)	−0.0011(7)	−0.0040(7)
C14	0.0212(9)	0.0167(8)	0.0185(9)	−0.0017(7)	−0.0001(7)	−0.0035(7)
C15	0.0127(8)	0.0208(10)	0.0190(9)	−0.0014(7)	0.0009(7)	0.0018(7)
C16	0.0195(9)	0.0303(11)	0.0194(10)	0.0002(8)	−0.0023(7)	0.0009(8)

TABLE S7. Bond lengths and angles for compound **18**.

Atom–Atom	Length [Å]
O2–C12	1.422(2)
O2–C11	1.442(2)
O3–C15	1.356(2)
O3–C1	1.438(2)
O4–C15	1.200(2)
O1–C5	1.220(2)
C1–C2	1.498(3)
C1–C14	1.514(2)
C1–C12	1.582(2)
C2–C6	1.343(3)
C2–C3	1.507(2)
C3–C4	1.534(3)
C3–H3A	0.9900
C3–H3B	0.9900

C4–C5	1.520(3)
C4–H4A	0.9900
C4–H4B	0.9900
C5–C6	1.472(3)
C6–C7	1.495(3)
C7–C8	1.530(2)
C7–H7A	0.9900
C7–H7B	0.9900
C8–C12	1.523(3)
C8–C9	1.530(2)
C8–H8	1.0000
C9–C10	1.532(3)
C9–H9A	0.9900
C9–H9B	0.9900
C10–C11	1.519(3)
C10–H10A	0.9900
C10–H10B	0.9900
C11–H11A	0.9900
C11–H11B	0.9900
C12–C13	1.530(2)
C13–C14	1.330(3)
C13–H13	0.9500
C14–H14	0.9500
C15–C16	1.497(3)
C16–H16A	0.9800
C16–H16B	0.9800
C16–H16C	0.9800
**Atom–Atom–Atom**	**Angle [°]**
C12–O2–C11	110.78(13)
C15–O3–C1	116.19(14)
O3–C1–C2	112.77(14)
O3–C1–C14	112.51(15)
C2–C1–C14	112.24(15)
O3–C1–C12	117.63(15)
C2–C1–C12	113.20(15)
C14–C1–C12	85.72(13)
C6–C2–C1	122.22(17)
C6–C2–C3	113.42(17)
C1–C2–C3	124.20(16)
C2–C3–C4	103.40(15)
C2–C3–H3A	111.1
C4–C3–H3A	111.1
C2–C3–H3B	111.1
C4–C3–H3B	111.1
H3A–C3–H3B	109.0
C5–C4–C3	105.39(16)
C5–C4–H4A	110.7
C3–C4–H4A	110.7
C5–C4–H4B	110.7
C3–C4–H4B	110.7
H4A–C4–H4B	108.8
O1–C5–C6	126.24(17)
O1–C5–C4	126.20(17)

C6–C5–C4	107.56(16)
C2–C6–C5	109.33(17)
C2–C6–C7	124.98(18)
C5–C6–C7	125.59(17)
C6–C7–C8	110.12(15)
C6–C7–H7A	109.6
C8–C7–H7A	109.6
C6–C7–H7B	109.6
C8–C7–H7B	109.6
H7A–C7–H7B	108.1
C12–C8–C7	111.55(15)
C12–C8–C9	108.71(14)
C7–C8–C9	113.44(15)
C12–C8–H8	107.6
C7–C8–H8	107.6
C9–C8–H8	107.6
C8–C9–C10	109.28(15)
C8–C9–H9A	109.8
C10–C9–H9A	109.8
C8–C9–H9B	109.8
C10–C9–H9B	109.8
H9A–C9–H9B	108.3
C11–C10–C9	110.83(15)
C11–C10–H10A	109.5
C9–C10–H10A	109.5
C11–C10–H10B	109.5
C9–C10–H10B	109.5
H10A–C10–H10B	108.1
O2–C11–C10	112.01(15)
O2–C11–H11A	109.2
C10–C11–H11A	109.2
O2–C11–H11B	109.2
C10–C11–H11B	109.2
H11A–C11–H11B	107.9
O2–C12–C8	110.60(14)
O2–C12–C13	113.06(15)
C8–C12–C13	119.53(16)
O2–C12–C1	110.17(14)
C8–C12–C1	116.57(15)
C13–C12–C1	84.49(13)
C14–C13–C12	94.60(15)
C14–C13–H13	132.7
C12–C13–H13	132.7
C13–C14–C1	94.55(15)
C13–C14–H14	132.7
C1–C14–H14	132.7
O4–C15–O3	123.59(17)
O4–C15–C16	125.58(19)
O3–C15–C16	110.81(17)
C15–C16–H16A	109.5
C15–C16–H16B	109.5
H16A–C16–H16B	109.5
C15–C16–H16C	109.5

H16A–C16–H16C	109.5
H16B–C16–H16C	109.5

TABLE S8. Torsion angles for compound **18**.

**Atom–Atom–Atom–Atom**	**Torsion Angle [°]**
C15–O3–C1–C2	65.01(19)
C15–O3–C1–C14	−166.79(15)
C15–O3–C1–C12	−69.6(2)
O3–C1–C2–C6	−150.46(17)
C14–C1–C2–C6	81.2(2)
C12–C1–C2–C6	−13.8(2)
O3–C1–C2–C3	34.4(2)
C14–C1–C2–C3	−93.9(2)
C12–C1–C2–C3	171.05(15)
C6–C2–C3–C4	−5.1(2)
C1–C2–C3–C4	170.42(16)
C2–C3–C4–C5	8.60(19)
C3–C4–C5–O1	169.92(18)
C3–C4–C5–C6	−9.5(2)
C1–C2–C6–C5	−176.48(15)
C3–C2–C6–C5	−0.9(2)
C1–C2–C6–C7	6.9(3)
C3–C2–C6–C7	−177.46(17)
O1–C5–C6–C2	−172.77(18)
C4–C5–C6–C2	6.6(2)
O1–C5–C6–C7	3.8(3)
C4–C5–C6–C7	−176.80(18)
C2–C6–C7–C8	26.6(3)
C5–C6–C7–C8	−149.40(17)
C6–C7–C8–C12	−50.7(2)
C6–C7–C8–C9	−173.91(15)
C12–C8–C9–C10	55.27(19)
C7–C8–C9–C10	179.98(15)
C8–C9–C10–C11	−52.0(2)
C12–O2–C11–C10	−59.31(19)
C9–C10–C11–O2	53.8(2)
C11–O2–C12–C8	63.30(18)
C11–O2–C12–C13	−73.78(18)
C11–O2–C12–C1	−166.37(14)
C7–C8–C12–O2	172.58(14)
C9–C8–C12–O2	−61.60(18)
C7–C8–C12–C13	−53.5(2)
C9–C8–C12–C13	72.3(2)
C7–C8–C12–C1	45.7(2)
C9–C8–C12–C1	171.54(15)
O3–C1–C12–O2	−6.1(2)
C2–C1–C12–O2	−140.47(15)
C14–C1–C12–O2	107.14(15)
O3–C1–C12–C8	121.00(17)
C2–C1–C12–C8	−13.4(2)
C14–C1–C12–C8	−125.79(16)
O3–C1–C12–C13	−118.65(16)
C2–C1–C12–C13	106.96(15)
14–C1–C12–C13	−5.43(13)
O2–C12–C13–C14	−103.42(17)
C8–C12–C13–C14	123.69(17)
C1–C12–C13–C14	6.18(15)
C12–C13–C14–C1	−6.45(16)
O3–C1–C14–C13	124.43(16)
C2–C1–C14–C13	−107.10(17)
C12–C1–C14–C13	6.24(15)
C1–O3–C15–O4	0.0(3)
C1–O3–C15–C16	−178.84(14)

4.2. SC-XRD structure report for compound 39 (CCDC 2418666).

FIGURE S2: Molecular structure of **39** in the solid-state. ADPs are given at the 50% probability level.

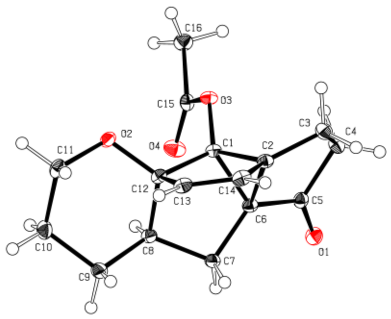

Compound **39** was dissolved in a minimum EtOAc in a 10 mL round-bottom flask. The neck of the flask was blocked with paper towel and the solvent slowly evaporated until formation of the crystal. A colourless, plate-shaped crystal of $C_{16}H_{18}O_4$ coated with perfluorinated ether and fixed on top of a Kapton micro sampler was used for X-ray crystallographic analysis. The X-ray intensity data were collected at 100(2) K on a Bruker D8 VENTURE three-angle diffractometer with a TXS rotating anode with MoK_{α} radiation (λ=0.71073 Å) using APEX4.[9] The diffractometer was equipped with a Helios optic monochromator, a Bruker PHOTON III detector, and an Oxford Cryostreamlow temperature device.

A matrix scan was used to determine the initial lattice parameters. All data were integrated with the Bruker SAINT V8.40B software package using a narrow-frame algorithm and the reflections were corrected for Lorentz and polarisation effects, scan speed, and background.[10] The integration of the data using a monoclinic unit cell yielded a total of 86099 reflections within a 2θ range [°] of 4.23 to 52.95 (0.80 Å), of which 2720 were independent. Data were corrected for absorption effects including odd and even ordered spherical harmonics by the multi-scan method (SADABS 2016/2).[11] Space group assignment was based upon systematic absences, E statistics, and successful refinement of the structure.

The structure was solved by direct methods using SHELXT and refined by full-matrix least-squares methods against F^2 by minimizing $\Sigma w(F_o^2 - F_c^2)^2$ using SHELXL in conjunction with SHELXLE.[12] All non-hydrogen atoms were refined with anisotropic displacement parameters. Hydrogen atoms were refined isotropically on calculated positions using a riding model with their U_{iso} values constrained to 1.5 times the U_{eq} of their pivot atoms for terminal sp^3 carbon atoms and a C–H distance of 0.98 Å. Non-methyl hydrogen atoms were refined using a riding model with methylene, aromatic, and other C–H distances of 0.99 Å, 0.95 Å, and 1.00 Å, respectively, and U_{iso} values constrained to 1.2 times the U_{eq} of their pivot atoms.

Neutral atom scattering factors for all atoms and anomalous dispersion corrections for the non-hydrogen atoms were taken from International Tables for Crystallography.[13] Supplementary crystallographic data reported in this paper have been deposited with the Cambridge Crystallographic Data Centre (CCDC 2418666) and can be obtained free of charge from The Cambridge Crystallographic Data Centre via www.ccdc.cam.ac.uk/structures.[14] This report and the CIF file were generated using FinalCif.[15]

TABLE S9. Crystal data and structure refinement for compound **39.**

CCDC number	2418666
Empirical formula	$C_{16}H_{18}O_4$
Formula weight	274.30
Temperature [K]	100(2)
Crystal system	monoclinic
Space group (number)	$P2_1/n$ (14)
a [Å]	7.5677(5)
b [Å]	9.0030(6)
c [Å]	19.4928(11)
α [°]	90
β [°]	98.736(2)
γ [°]	90
Volume [Å^3]	1312.68(14)
Z	4
ρ_{calc} [gcm^{-3}]	1.388
μ [mm^{-1}]	0.099
F(000)	584
Crystal size [mm^3]	0.072×0.105×0.147
Crystal colour	colourless
Crystal shape	plate
Radiation	MoK_α (λ=0.71073 Å)
2θ range [°]	4.23 to 52.95 (0.80 Å)
Index ranges	−9 ≤ h ≤ 9<br>−11 ≤ k ≤ 11<br>−24 ≤ l ≤ 24
Reflections collected	86099
Independent reflections	2720<br>R_{int} = 0.0881<br>R_{sigma} = 0.0220
Completeness to θ = 25.242°	100.0
Data / Restraints / Parameters	2720 / 0 / 182
Goodness-of-fit on F^2	1.053
Final R indexes [I≥2σ(I)]	R_1 = 0.0341<br>wR_2 = 0.0894
Final R indexes [all data]	R_1 = 0.0427<br>wR_2 = 0.0940
Largest peak/hole [eÅ^{-3}]	0.36/−0.23

Atom	*x*	*y*	*z*	U_{eq}
C2	0.92837(16)	0.82365(14)	0.39800(6)	0.0155(3)
C3	1.06143(16)	0.74203(15)	0.45075(6)	0.0189(3)
H3A	1.168300	0.804267	0.465507	0.023
H3B	1.099728	0.648286	0.430898	0.023
C4	0.96236(17)	0.70992(15)	0.51259(6)	0.0191(3)
H4A	0.913496	0.607722	0.509665	0.023
H4B	1.044273	0.720758	0.557002	0.023
C5	0.81201(15)	0.82340(13)	0.50774(6)	0.0154(3)
C1	0.74848(15)	0.76171(13)	0.37676(6)	0.0137(2)
O1	0.74663(12)	0.86994(10)	0.55675(4)	0.0201(2)
O2	0.56699(11)	0.76313(10)	0.25965(4)	0.0173(2)
O3	0.71742(11)	0.60790(9)	0.38127(4)	0.0154(2)
O4	0.45798(12)	0.64798(10)	0.42229(5)	0.0236(2)
C6	0.76538(16)	0.87472(13)	0.43521(6)	0.0146(3)
C8	0.53766(16)	0.95860(13)	0.34429(6)	0.0150(3)
H8	0.439570	0.896530	0.358332	0.018
C9	0.45028(17)	1.06659(14)	0.28951(6)	0.0188(3)
H9A	0.541610	1.131085	0.273360	0.023
H9B	0.363008	1.130229	0.308741	0.023
C10	0.35540(17)	0.97220(15)	0.22919(6)	0.0210(3)
H10A	0.251726	0.921515	0.244271	0.025
H10B	0.309543	1.038340	0.189932	0.025
C11	0.47840(17)	0.85603(15)	0.20445(6)	0.0197(3)
H11A	0.407275	0.792263	0.169156	0.024
H11B	0.570034	0.907219	0.181853	0.024
C12	0.66287(16)	0.85201(13)	0.31281(6)	0.0147(3)
C13	0.82374(17)	0.93391(14)	0.29223(6)	0.0175(3)
H13	0.821084	0.988609	0.250413	0.021
C14	0.96778(17)	0.91921(14)	0.34002(6)	0.0178(3)
H14	1.080455	0.963656	0.337474	0.021
C15	0.56283(16)	0.56339(14)	0.40340(6)	0.0160(3)
C16	0.54949(18)	0.39790(14)	0.40237(7)	0.0208(3)
H16A	0.423928	0.368487	0.399587	0.031
H16B	0.618417	0.357429	0.444884	0.031
H16C	0.597385	0.359173	0.361946	0.031
C7	0.65363(16)	1.01091(13)	0.41169(6)	0.0157(2)
H7A	0.730384	1.095654	0.402801	0.019
H7B	0.578600	1.040541	0.446801	0.019

U_{eq} is defined as 1/3 of the trace of the orthogonalized U_{ij} tensor.

TABLE S10. Anisotropic displacement parameters (Å^2) for compound **39**. The anisotropic displacement factor exponent takes the form: $-2\pi^2[h^2(a^*)^2U_{11} + k^2(b^*)^2U_{22} + \ldots + 2hka^*b^*U_{12}]$

Atom	U_{11}	U_{22}	U_{33}	U_{23}	U_{13}	U_{12}

C2	0.0155(6)	0.0147(6)	0.0173(5)	−0.0021(5)	0.0054(4)	0.0005(5)
C3	0.0160(6)	0.0205(6)	0.0207(6)	0.0006(5)	0.0041(5)	0.0024(5)
C4	0.0177(6)	0.0209(6)	0.0185(6)	0.0029(5)	0.0019(5)	0.0025(5)
C5	0.0142(6)	0.0149(6)	0.0168(6)	0.0000(5)	0.0016(4)	−0.0029(5)
C1	0.0156(6)	0.0113(6)	0.0148(5)	−0.0008(4)	0.0046(4)	−0.0004(4)
O1	0.0220(5)	0.0239(5)	0.0146(4)	−0.0006(3)	0.0040(3)	0.0015(4)
O2	0.0214(5)	0.0156(4)	0.0144(4)	−0.0028(3)	0.0008(3)	−0.0015(3)
O3	0.0167(4)	0.0109(4)	0.0195(4)	−0.0002(3)	0.0058(3)	−0.0004(3)
O4	0.0194(5)	0.0196(5)	0.0338(5)	0.0008(4)	0.0105(4)	0.0009(4)
C6	0.0148(6)	0.0140(6)	0.0155(6)	−0.0017(4)	0.0040(4)	0.0001(4)
C8	0.0173(6)	0.0136(6)	0.0144(5)	−0.0006(4)	0.0032(4)	0.0003(5)
C9	0.0225(6)	0.0167(6)	0.0166(6)	0.0012(5)	0.0012(5)	0.0028(5)
C10	0.0221(6)	0.0240(7)	0.0156(6)	0.0015(5)	−0.0009(5)	0.0007(5)
C11	0.0236(6)	0.0226(6)	0.0126(5)	0.0000(5)	0.0010(5)	−0.0034(5)
C12	0.0176(6)	0.0138(6)	0.0131(5)	−0.0015(4)	0.0030(4)	−0.0018(5)
C13	0.0227(6)	0.0156(6)	0.0156(5)	−0.0010(5)	0.0078(5)	−0.0021(5)
C14	0.0191(6)	0.0167(6)	0.0196(6)	−0.0024(5)	0.0087(5)	−0.0033(5)
C15	0.0151(6)	0.0178(6)	0.0147(5)	0.0019(4)	0.0010(4)	−0.0013(5)
C16	0.0227(6)	0.0155(6)	0.0245(6)	0.0017(5)	0.0042(5)	−0.0028(5)
C7	0.0179(6)	0.0140(6)	0.0152(5)	−0.0010(4)	0.0025(4)	0.0024(5)

TABLE S11. Bond lengths and angles for compound **39**.

**Atom–Atom**	**Length [Å]**
C2–C1	1.4713(17)
C2–C14	1.4863(17)
C2–C3	1.5155(17)
C2–C6	1.5908(16)
C3–C4	1.5406(17)
C3–H3A	0.9900
C3–H3B	0.9900
C4–C5	1.5214(17)
C4–H4A	0.9900
C4–H4B	0.9900
C5–O1	1.2152(15)
C5–C6	1.4783(16)
C1–O3	1.4095(14)
C1–C6	1.5183(16)
C1–C12	1.5458(16)
O2–C12	1.4184(14)
O2–C11	1.4449(15)
O3–C15	1.3670(14)
O4–C15	1.1974(16)
C6–C7	1.5201(16)
C8–C9	1.5192(16)
C8–C7	1.5388(16)

C8–C12	1.5401(16)
C8–H8	1.0000
C9–C10	1.5375(17)
C9–H9A	0.9900
C9–H9B	0.9900
C10–C11	1.5262(18)
C10–H10A	0.9900
C10–H10B	0.9900
C11–H11A	0.9900
C11–H11B	0.9900
C12–C13	1.5281(16)
C13–C14	1.3285(18)
C13–H13	0.9500
C14–H14	0.9500
C15–C16	1.4933(18)
C16–H16A	0.9800
C16–H16B	0.9800
C16–H16C	0.9800
C7–H7A	0.9900
C7–H7B	0.9900
**Atom–Atom–Atom**	**Angle [°]**
C1–C2–C14	106.82(10)
C1–C2–C3	119.88(11)
C14–C2–C3	127.39(11)
C1–C2–C6	59.30(7)
C14–C2–C6	116.01(10)
C3–C2–C6	108.19(9)
C2–C3–C4	105.65(10)
C2–C3–H3A	110.6
C4–C3–H3A	110.6
C2–C3–H3B	110.6
C4–C3–H3B	110.6
H3A–C3–H3B	108.7
C5–C4–C3	105.87(10)
C5–C4–H4A	110.6
C3–C4–H4A	110.6
C5–C4–H4B	110.6
C3–C4–H4B	110.6
H4A–C4–H4B	108.7
O1–C5–C6	125.28(11)
O1–C5–C4	124.93(11)
C6–C5–C4	109.70(10)
O3–C1–C2	120.72(10)
O3–C1–C6	127.40(10)

C2–C1–C6	64.27(8)
O3–C1–C12	120.90(10)
C2–C1–C12	106.51(9)
C6–C1–C12	103.29(9)
C12–O2–C11	110.21(9)
C15–O3–C1	117.80(9)
C5–C6–C1	119.26(10)
C5–C6–C7	125.56(10)
C1–C6–C7	109.55(10)
C5–C6–C2	104.87(9)
C1–C6–C2	56.43(7)
C7–C6–C2	122.00(10)
C9–C8–C7	122.35(10)
C9–C8–C12	110.32(9)
C7–C8–C12	103.02(9)
C9–C8–H8	106.7
C7–C8–H8	106.7
C12–C8–H8	106.7
C8–C9–C10	106.65(10)
C8–C9–H9A	110.4
C10–C9–H9A	110.4
C8–C9–H9B	110.4
C10–C9–H9B	110.4
H9A–C9–H9B	108.6
C11–C10–C9	112.58(10)
C11–C10–H10A	109.1
C9–C10–H10A	109.1
C11–C10–H10B	109.1
C9–C10–H10B	109.1
H10A–C10–H10B	107.8
O2–C11–C10	113.43(9)
O2–C11–H11A	108.9
C10–C11–H11A	108.9
O2–C11–H11B	108.9
C10–C11–H11B	108.9
H11A–C11–H11B	107.7
O2–C12–C13	114.84(9)
O2–C12–C8	111.42(9)
C13–C12–C8	111.52(10)
O2–C12–C1	113.43(10)
C13–C12–C1	102.26(9)
C8–C12–C1	102.37(9)
C14–C13–C12	111.68(10)
C14–C13–H13	124.2
C12–C13–H13	124.2
C13–C14–C2	110.66(11)

C13–C14–H14	124.7
C2–C14–H14	124.7
O4–C15–O3	123.33(11)
O4–C15–C16	126.31(12)
O3–C15–C16	110.34(10)
C15–C16–H16A	109.5
C15–C16–H16B	109.5
H16A–C16–H16B	109.5
C15–C16–H16C	109.5
H16A–C16–H16C	109.5
H16B–C16–H16C	109.5
C6–C7–C8	103.28(9)
C6–C7–H7A	111.1
C8–C7–H7A	111.1
C6–C7–H7B	111.1
C8–C7–H7B	111.1
H7A–C7–H7B	109.1

TABLE S12. Torsion angles for compound **39**.

**Atom–Atom–Atom–Atom**	**Torsion Angle [°]**
C1–C2–C3–C4	−51.06(14)
C14–C2–C3–C4	159.71(12)
C6–C2–C3–C4	13.41(13)
C2–C3–C4–C5	−22.58(13)
C3–C4–C5–O1	−152.35(12)
C3–C4–C5–C6	24.30(13)
C14–C2–C1–O3	129.45(11)
C3–C2–C1–O3	−25.42(16)
C6–C2–C1–O3	−119.87(12)
C14–C2–C1–C6	−110.68(11)
C3–C2–C1–C6	94.45(11)
C14–C2–C1–C12	−13.60(12)
C3–C2–C1–C12	−168.47(10)
C6–C2–C1–C12	97.09(10)
C2–C1–O3–C15	140.14(11)
C6–C1–O3–C15	60.58(15)
C12–C1–O3–C15	−82.06(13)
O1–C5–C6–C1	−139.40(12)
C4–C5–C6–C1	43.97(14)
O1–C5–C6–C7	11.31(19)
C4–C5–C6–C7	−165.32(11)
O1–C5–C6–C2	161.07(12)
C4–C5–C6–C2	−15.57(13)

O3–C1–C6–C5	21.28(17)
C2–C1–C6–C5	−88.92(11)
C12–C1–C6–C5	168.94(10)
O3–C1–C6–C7	−133.74(12)
C2–C1–C6–C7	116.06(11)
C12–C1–C6–C7	13.92(12)
O3–C1–C6–C2	110.20(13)
C12–C1–C6–C2	−102.14(10)
C1–C2–C6–C5	115.52(11)
C14–C2–C6–C5	−149.62(10)
C3–C2–C6–C5	1.02(13)
C14–C2–C6–C1	94.86(11)
C3–C2–C6–C1	−114.50(11)
C1–C2–C6–C7	−93.38(12)
C14–C2–C6–C7	1.49(16)
C3–C2–C6–C7	152.12(11)
C7–C8–C9–C10	177.10(10)
C12–C8–C9–C10	55.84(13)
C8–C9–C10–C11	−51.74(13)
C12–O2–C11–C10	−55.65(13)
C9–C10–C11–O2	52.80(14)
C11–O2–C12–C13	−67.80(12)
C11–O2–C12–C8	60.21(12)
C11–O2–C12–C1	175.10(9)
C9–C8–C12–O2	−62.81(12)
C7–C8–C12–O2	165.02(9)
C9–C8–C12–C13	66.95(12)
C7–C8–C12–C13	−65.21(11)
C9–C8–C12–C1	175.63(10)
C7–C8–C12–C1	43.46(11)
O3–C1–C12–O2	−4.82(15)
C2–C1–C12–O2	138.14(10)
C6–C1–C12–O2	−155.13(9)
O3–C1–C12–C13	−129.06(10)
C2–C1–C12–C13	13.90(11)
C6–C1–C12–C13	80.63(10)
O3–C1–C12–C8	115.35(11)
C2–C1–C12–C8	−101.69(10)
C6–C1–C12–C8	−34.97(11)
O2–C12–C13–C14	−132.99(11)
C8–C12–C13–C14	99.06(12)
C1–C12–C13–C14	−9.69(13)
C12–C13–C14–C2	1.56(14)
C1–C2–C14–C13	7.85(14)
C3–C2–C14–C13	160.24(12)
C6–C2–C14–C13	−55.66(14)

C1–O3–C15–O4	−3.88(17)
C1–O3–C15–C16	177.87(10)
C5–C6–C7–C8	−140.36(11)
C1–C6–C7–C8	12.71(12)
C2–C6–C7–C8	74.67(13)
C9–C8–C7–C6	−159.09(11)
C12–C8–C7–C6	−34.46(11)

5. UV-vis spectra of compounds 9 and 18

5.1. UV-vis spectra of compound 9

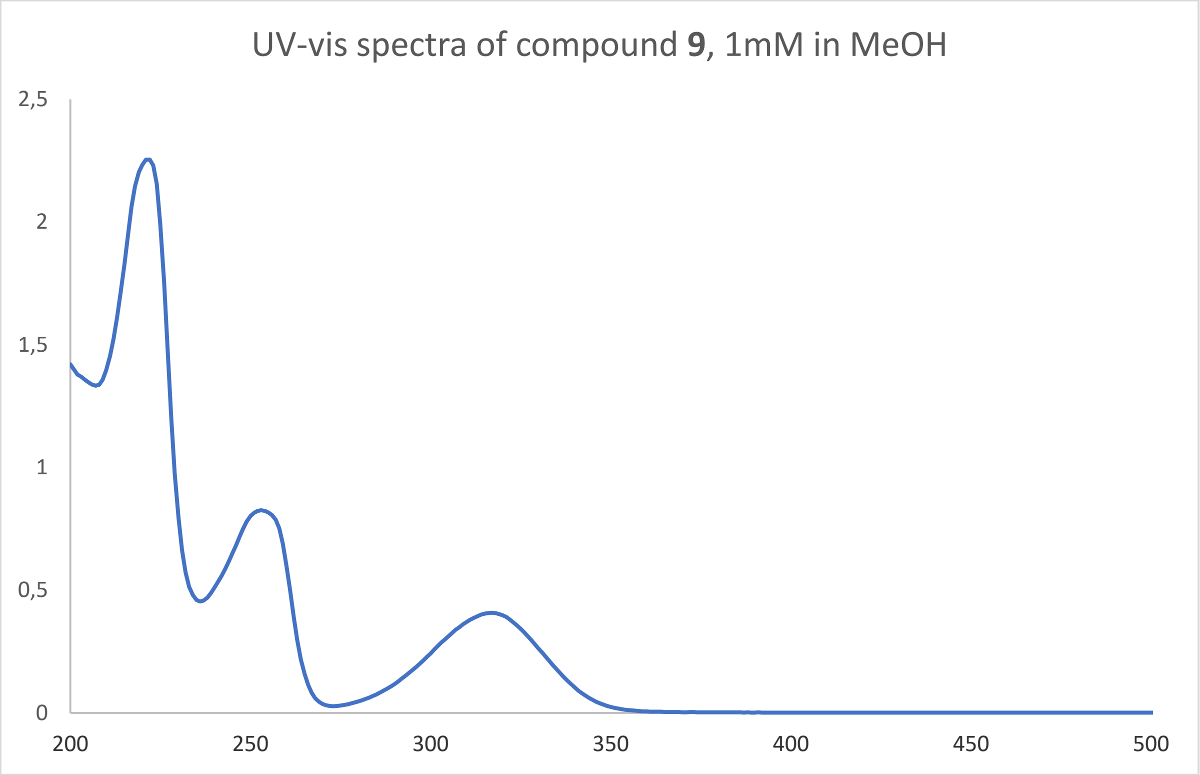

5.2.UV-vis spectra of compound 18

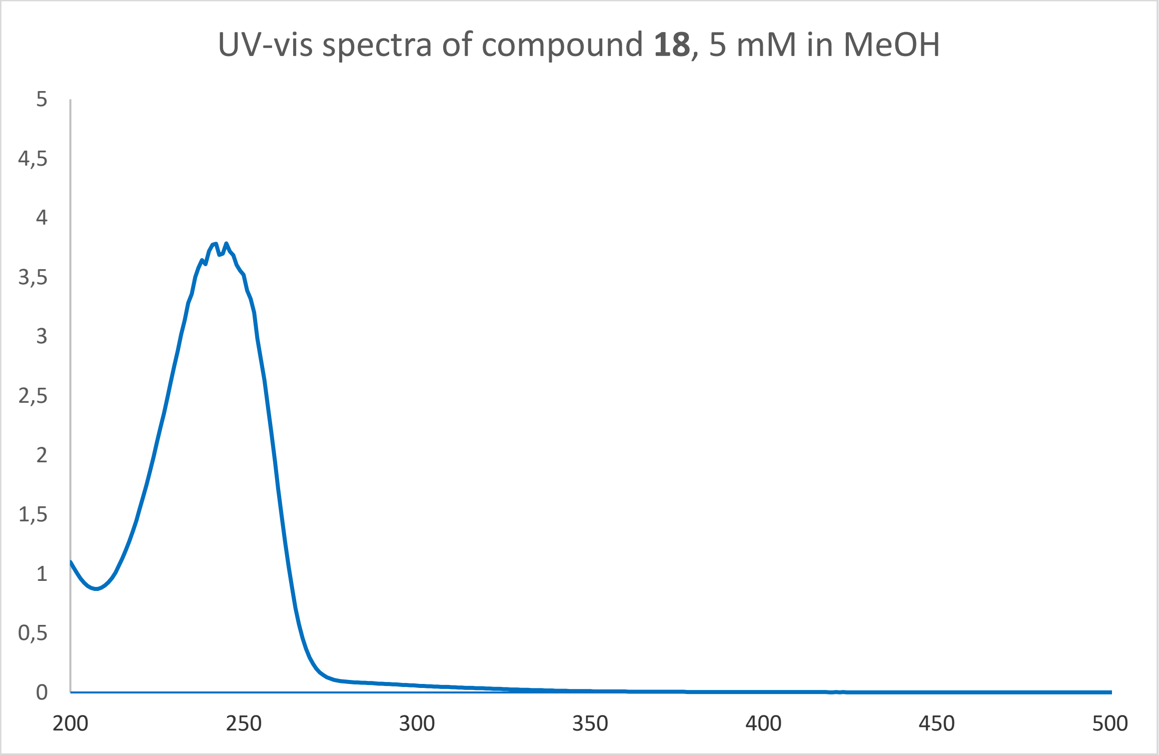

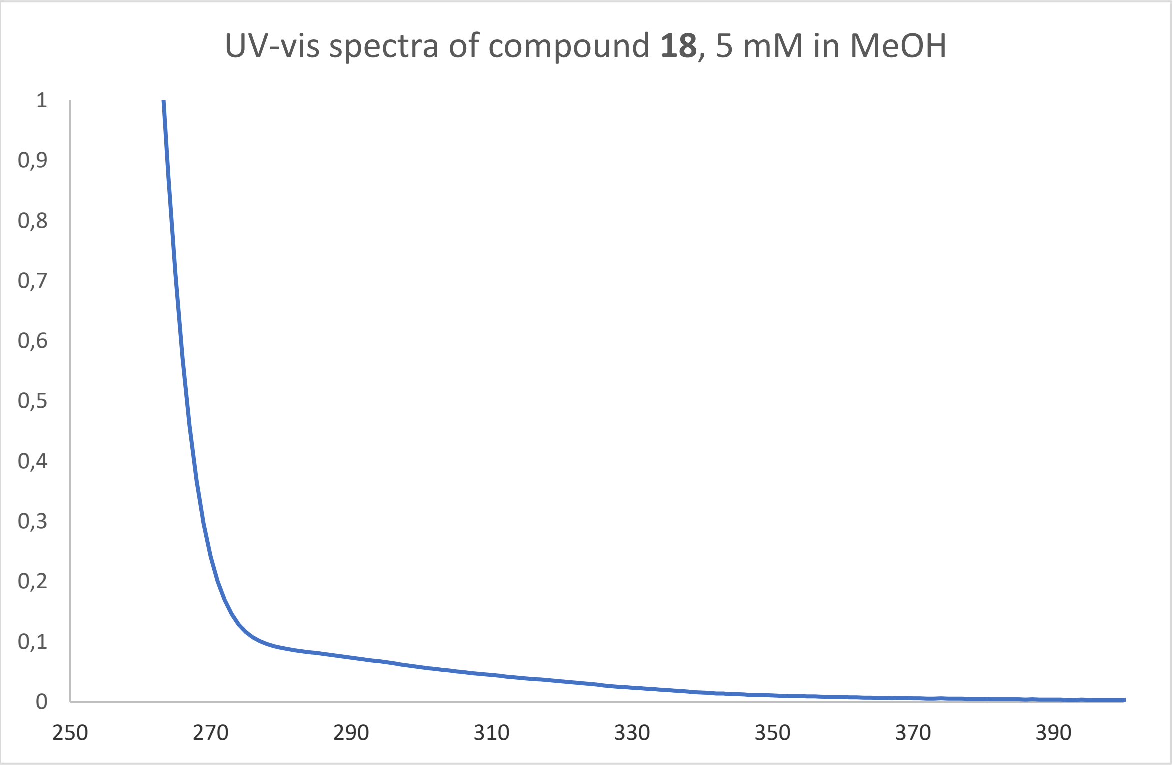

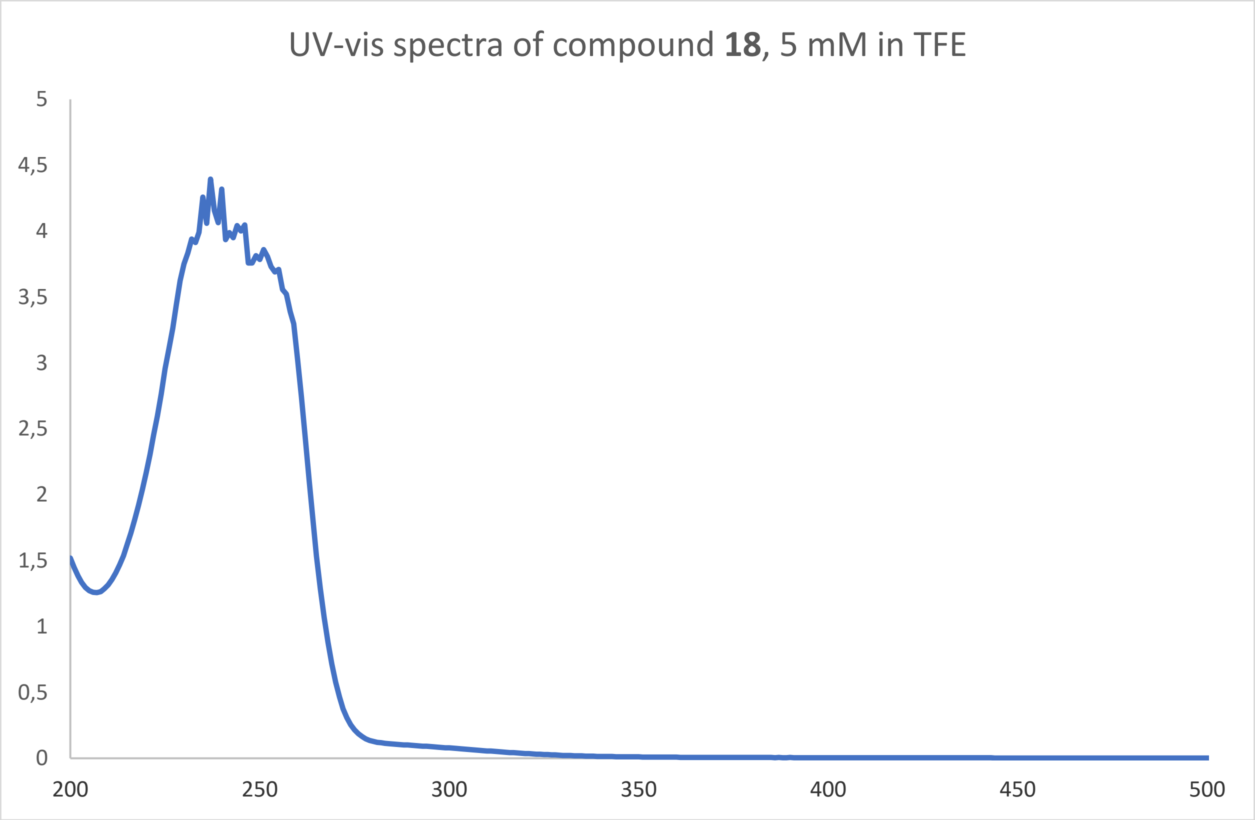
UV-vis spectra of compound **18**, 5 mM in TFE
5
4,5
4
3,5
3
2,5
2
1,5
1
0,5
0
200
250
300
350
400
450
500

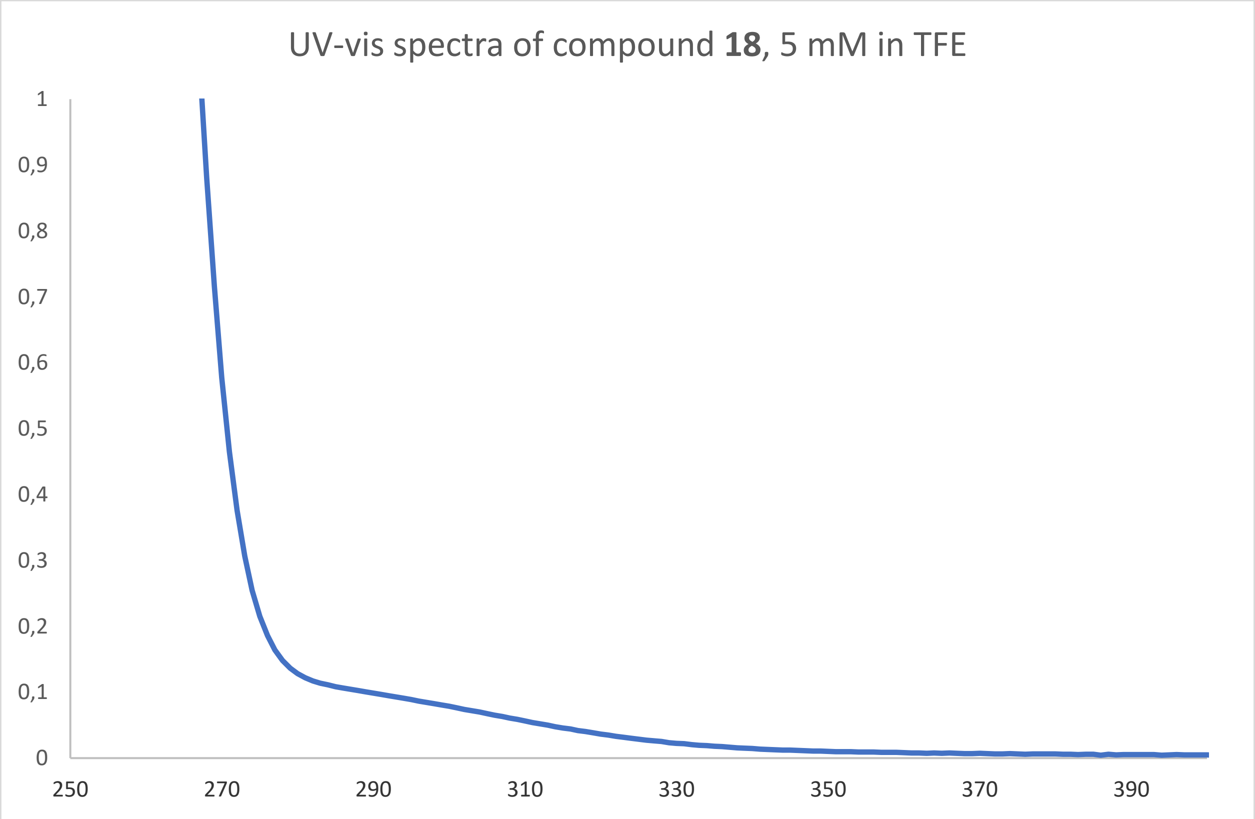
UV-vis spectra of compound **18**, 5 mM in TFE
1
0,9
0,8
0,7
0,6
0,5
0,4
0,3
0,2
0,1
0
250
270
290
310
330
350
370
390

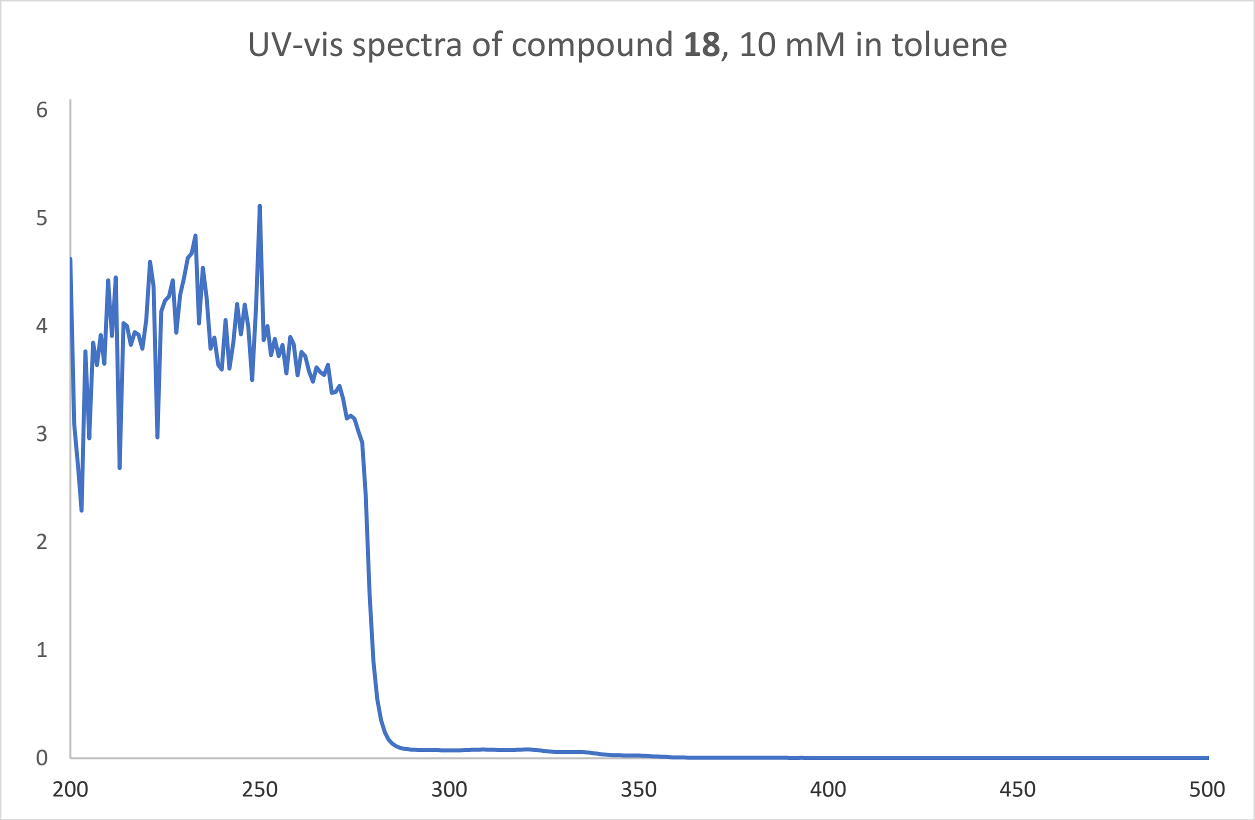
UV-vis spectra of compound **18**, 10 mM in toluene
6
5
4
3
2
1
0
200
250
300
350
400
450
500

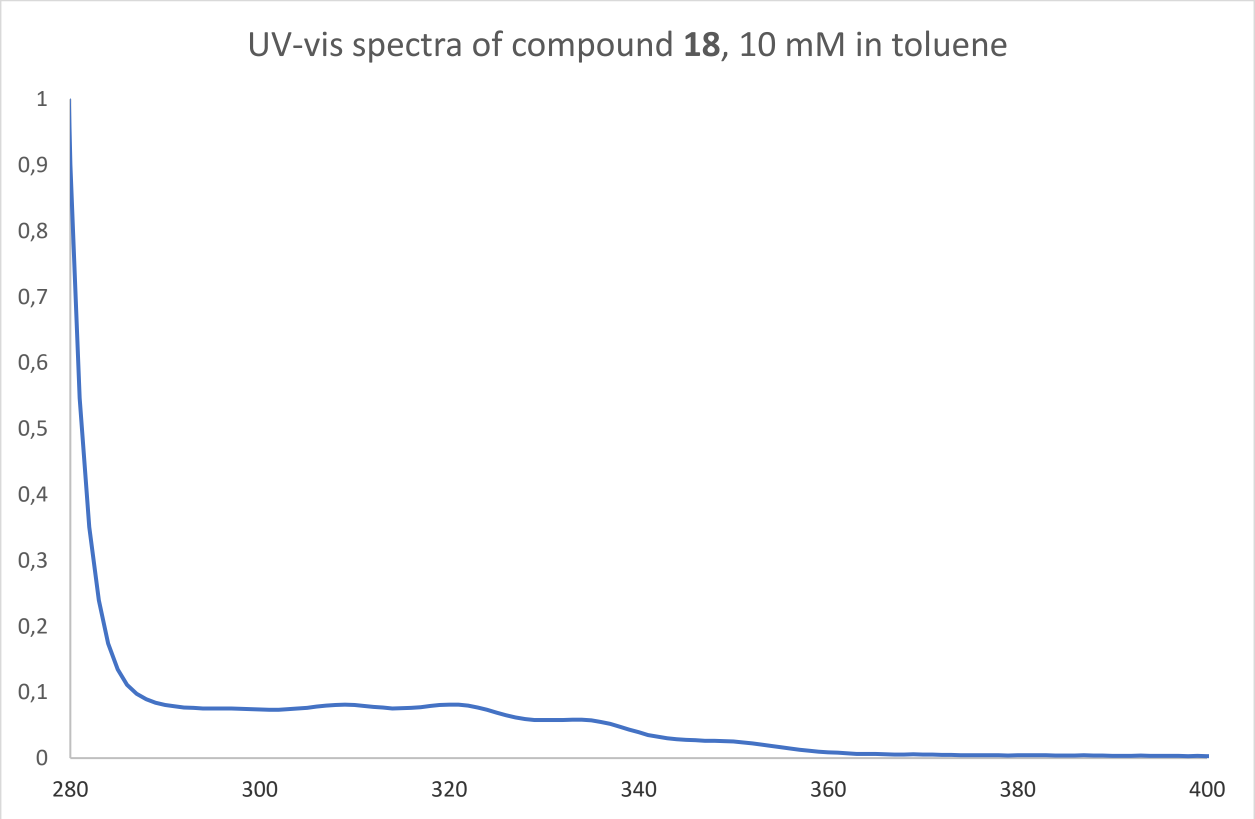
UV-vis spectra of compound **18**, 10 mM in toluene
1
0,9
0,8
0,7
0,6
0,5
0,4
0,3
0,2
0,1
0
280
300
320
340
360
380
400

6. Emission spectra of the light source

Lehrstuhl OC 1 - TUM

200 nm | 250 nm | 300 nm | 350 nm | 400 nm | 450 nm | 500 nm | 550 nm | 600 nm | 850 nm

Datasheet FLT021 LZC-UVA

Basic Information

Type	Fluorescent light tube
Description	Luzchem LZC-UVA
Manufacturer / Supplier	Hitachi / Luzchem
Order number / Date of purch.	LZC-UVA / 09/2015
Internal lot / serial number	2015-09 / FLT021

Specification Manufacturer

Type / size	T5 tube, G5 socket
Mechanical specification	16 mm diameter, 288 mm length
Electrical specification	8 W
Wavelength (range, typ.)	300 - 400 nm, 350m nm, UV-A
Spectral width (FWHM)	~ 40 nm
Datasheet	

Characterization

Description of measurement	Measured with Ocean-optics USB4000 spectrometer using a calibrated setup (cosine corrector/fibre). The cosine corrector was placed at 20 mm distance from a single fluorescent tube at half height.	
Measured dominant wavelength / Int.	350 nm	115 μW/mm²nm
Measured spectral width (FWHM)	40 nm	
Integral Reference intensity / range	5017 μW/cm²	300-425 nm

Spectrum

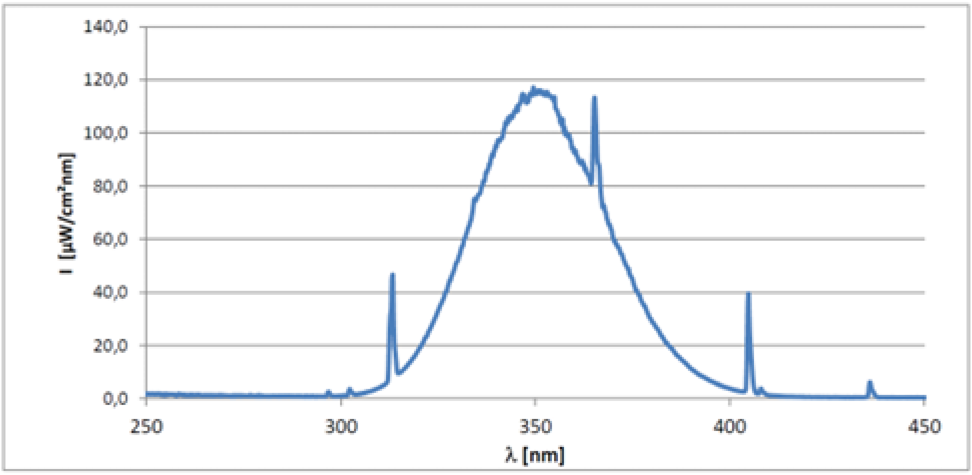

7. NMR Spectra

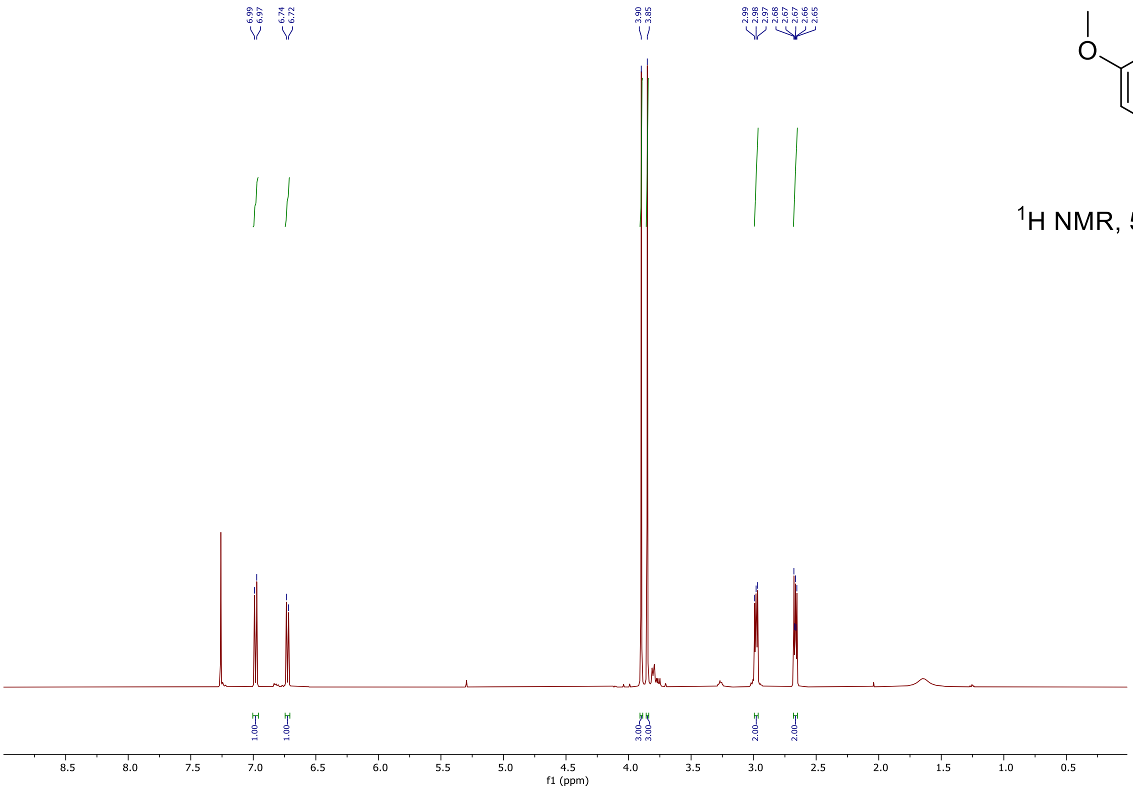

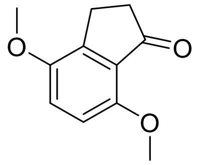

5

^{1}H NMR, 500 MHz, $CDCl_3$

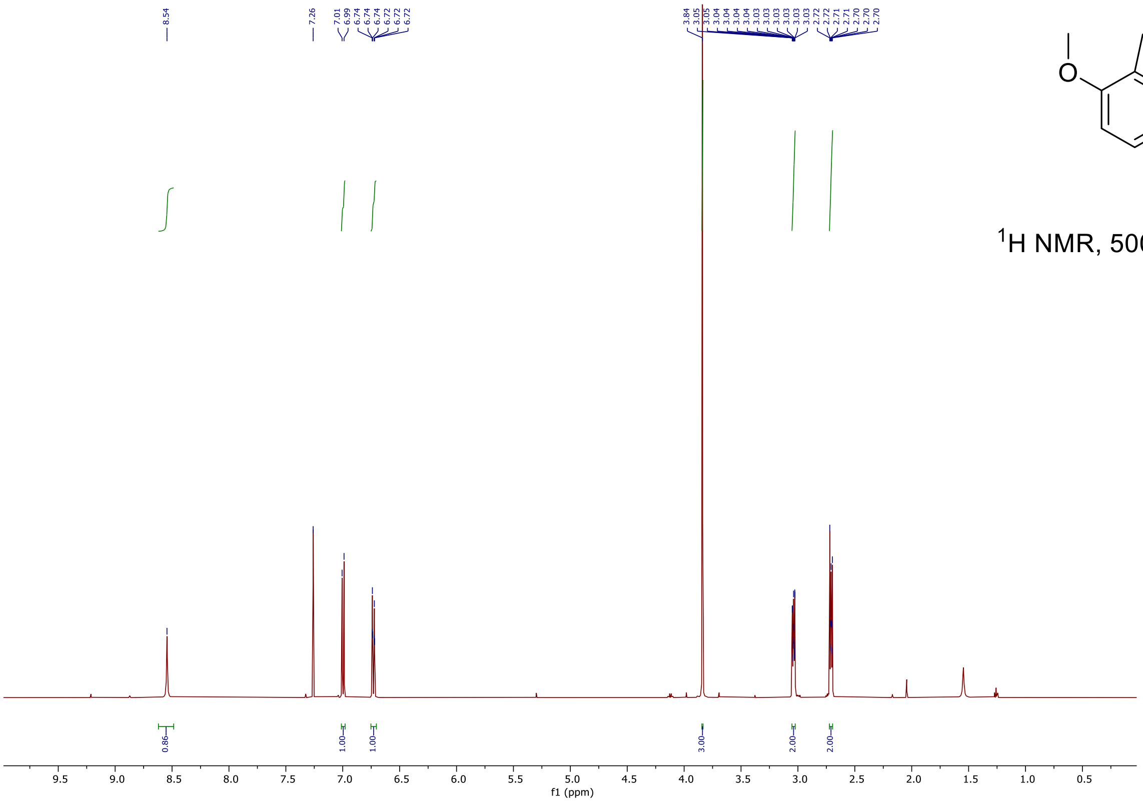

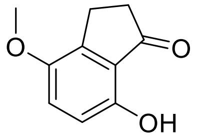

6

^{1}H NMR, 500 MHz, $CDCl_3$

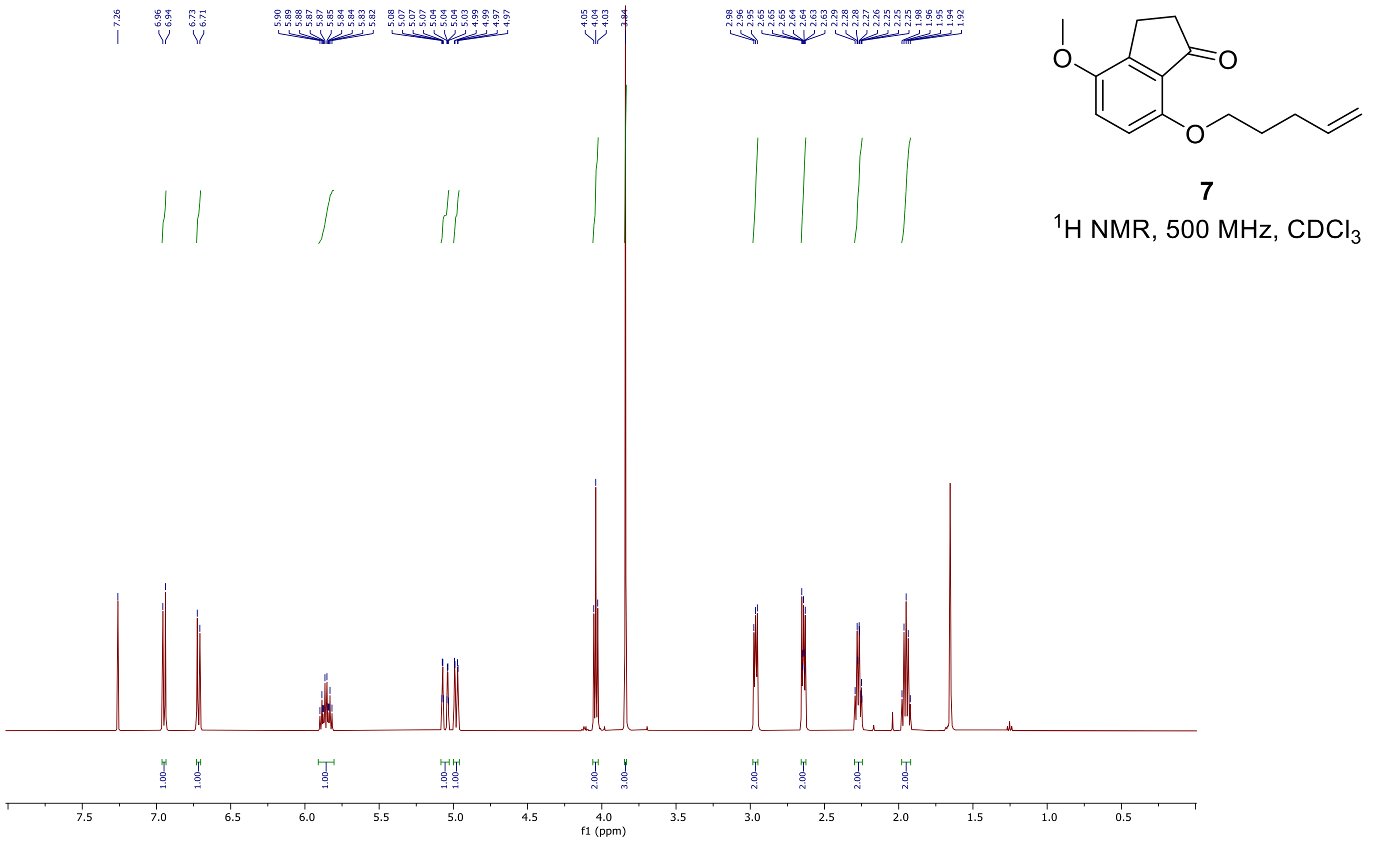

7

^{1}H NMR, 500 MHz, $CDCl_3$

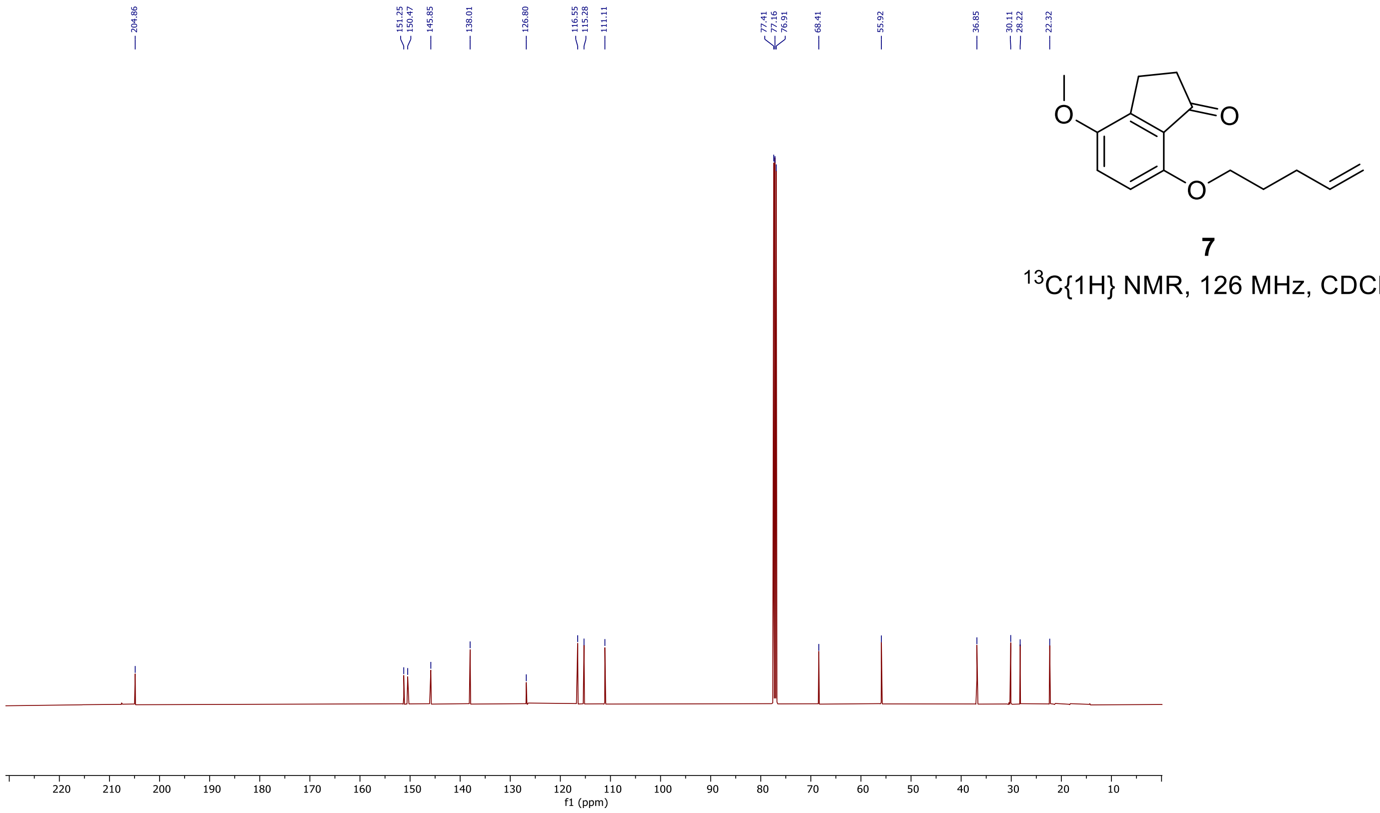

7

$^{13}C\{1H\}$ NMR, 126 MHz, $CDCl_3$

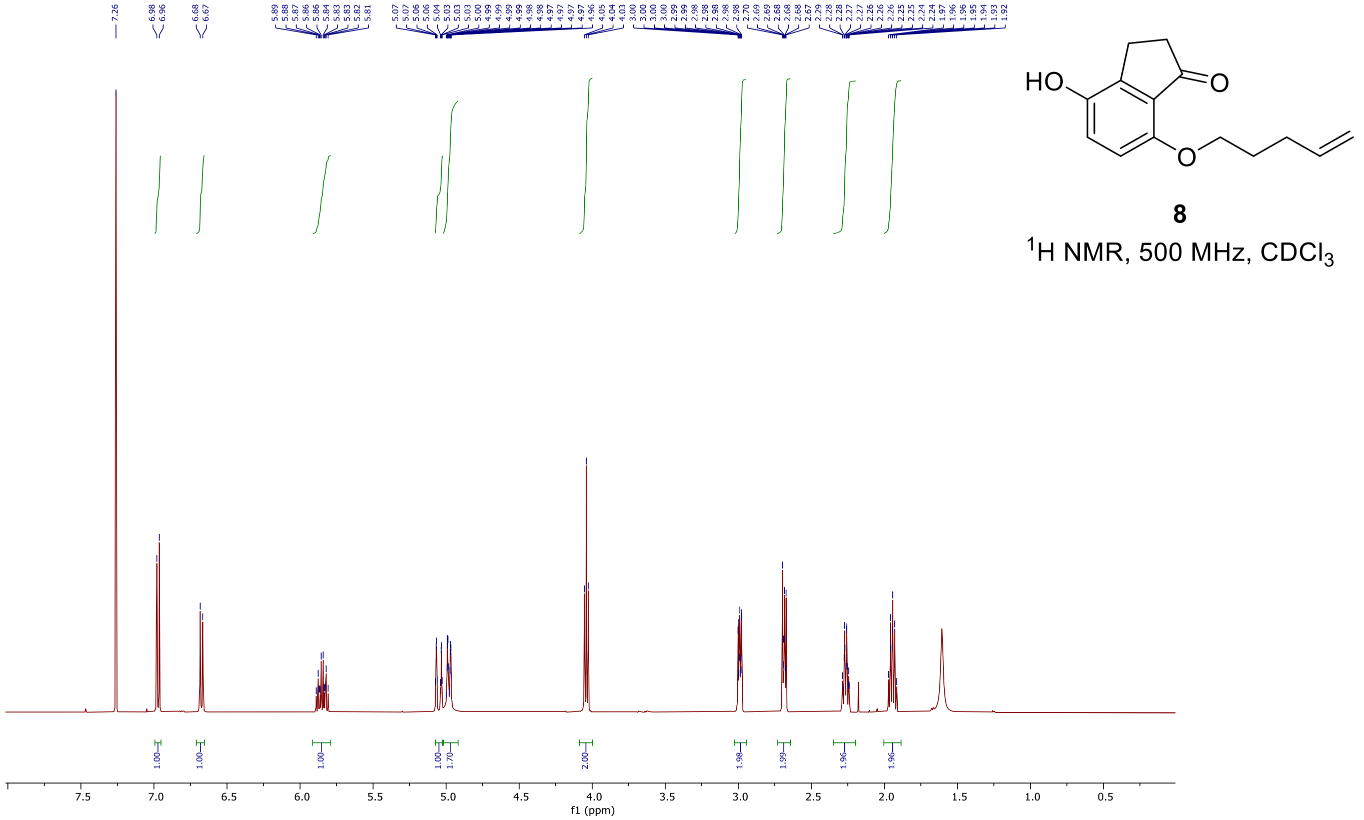
HO
O
O
8
^{1}H NMR, 500 MHz, $CDCl_3$
f1 (ppm)

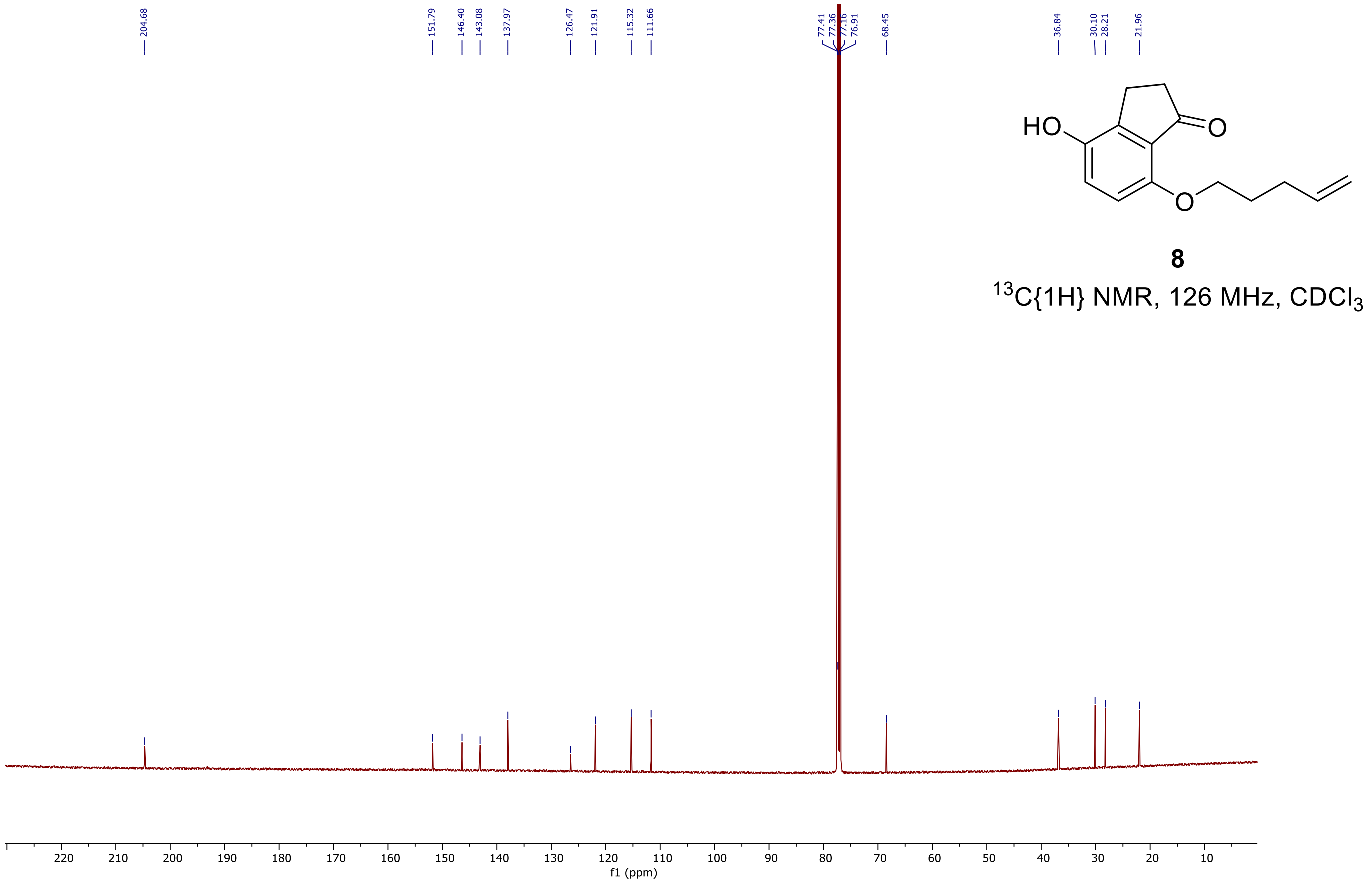
HO
O
O
8
^{13}C{1H} NMR, 126 MHz, $CDCl_3$
f1 (ppm)

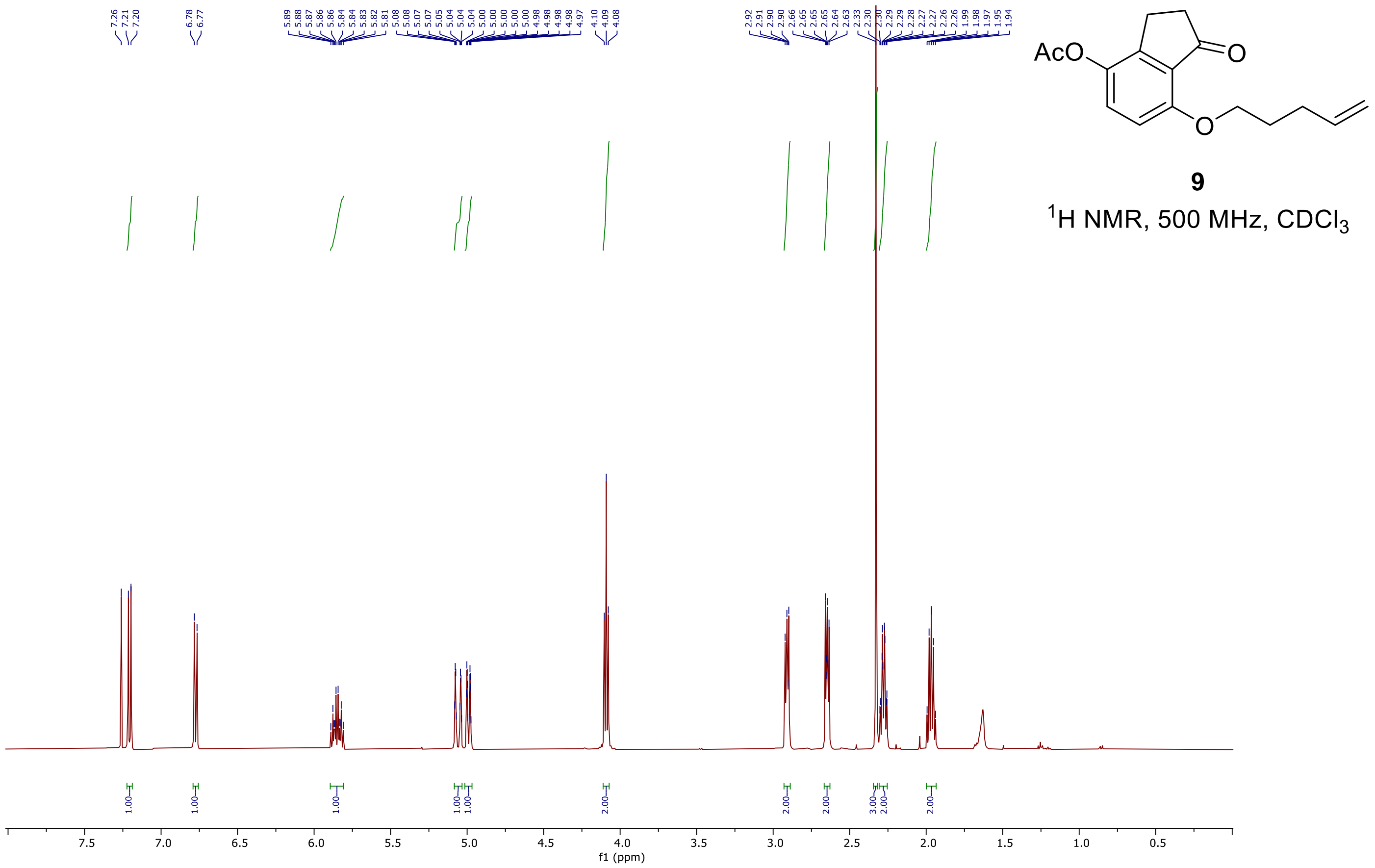
AcO
O
O
9
1H NMR, 500 MHz, CDCl3
f1 (ppm)

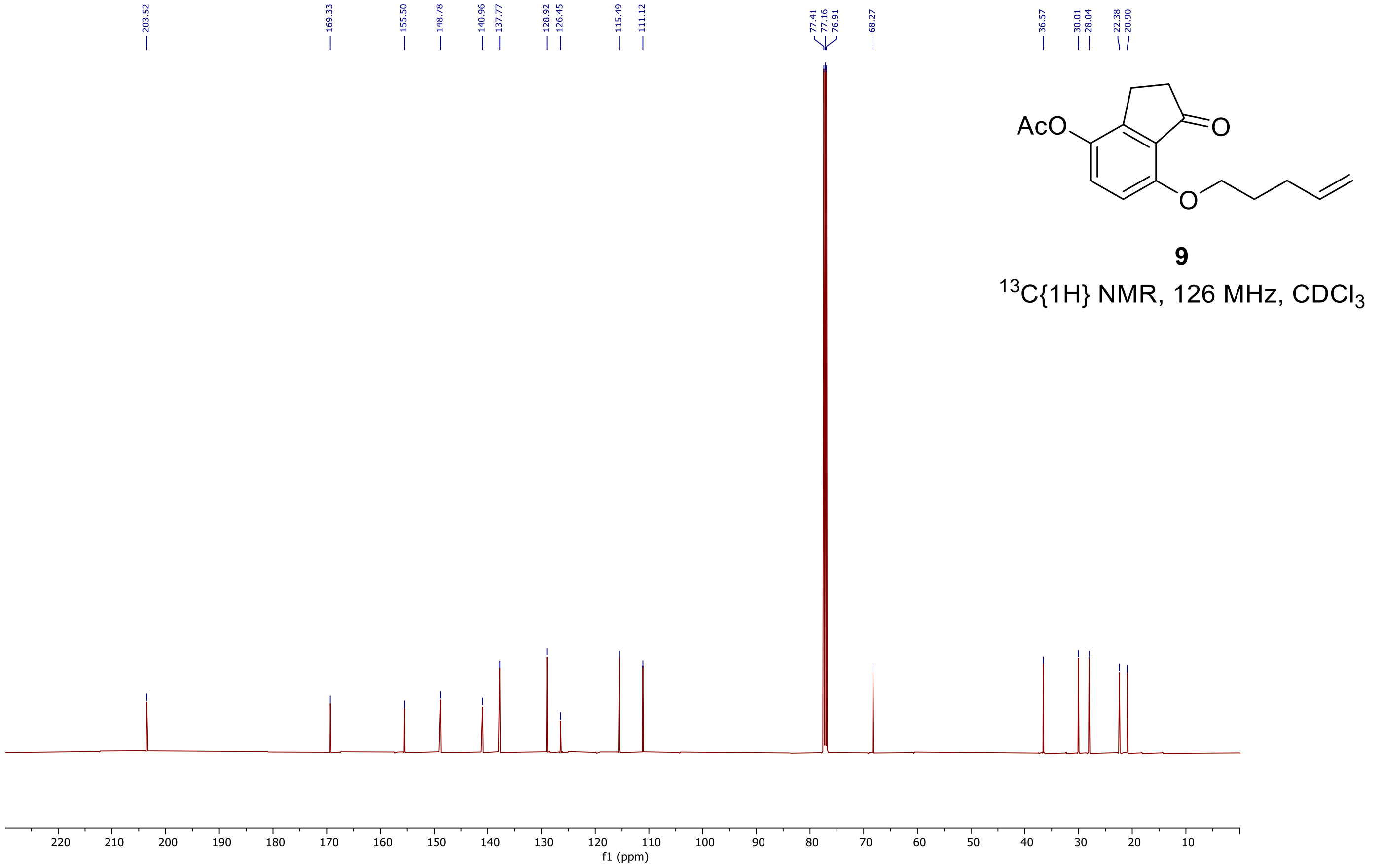
AcO
O
O
9
13C{1H} NMR, 126 MHz, CDCl3
f1 (ppm)

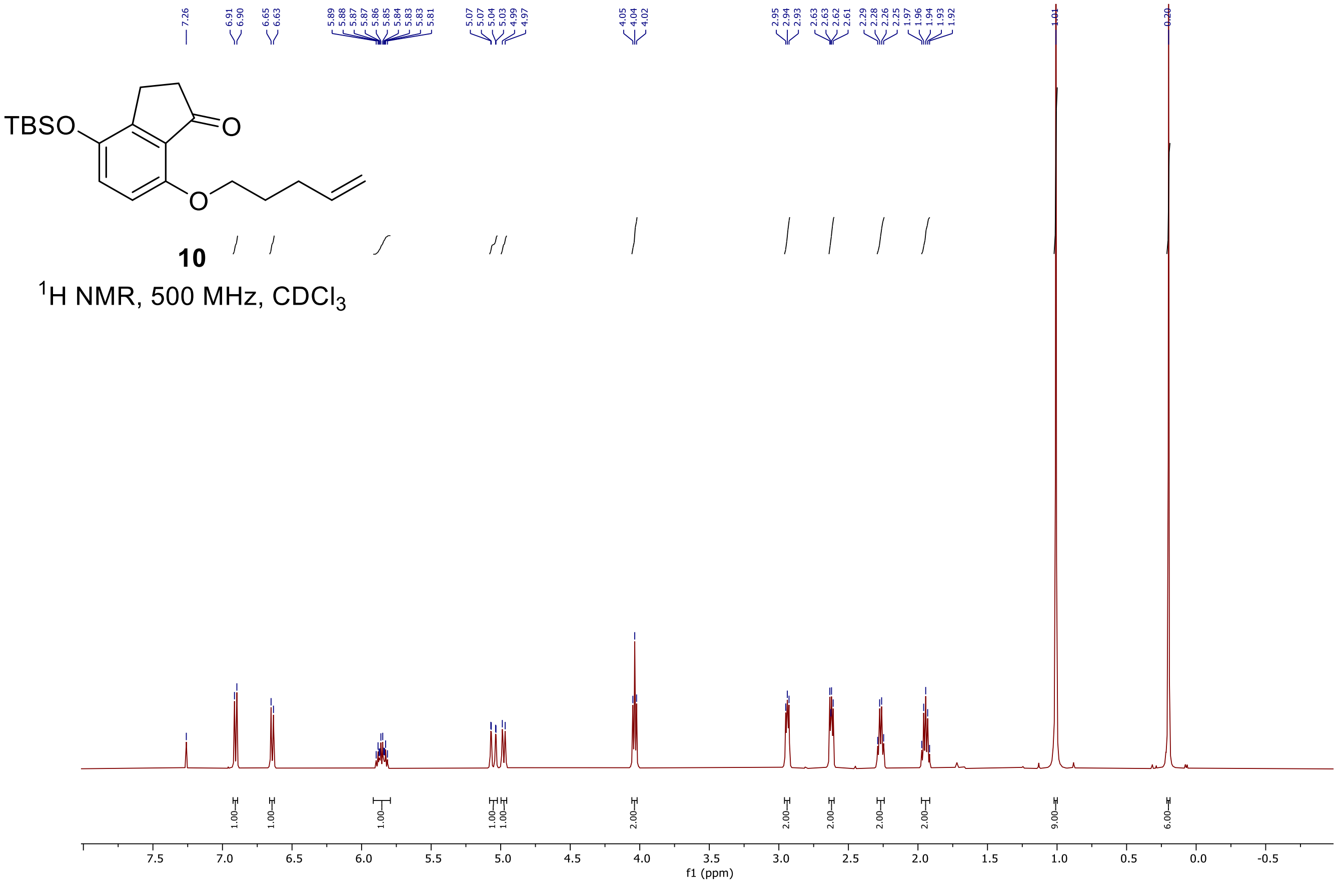

10

^{1}H NMR, 500 MHz, $CDCl_3$

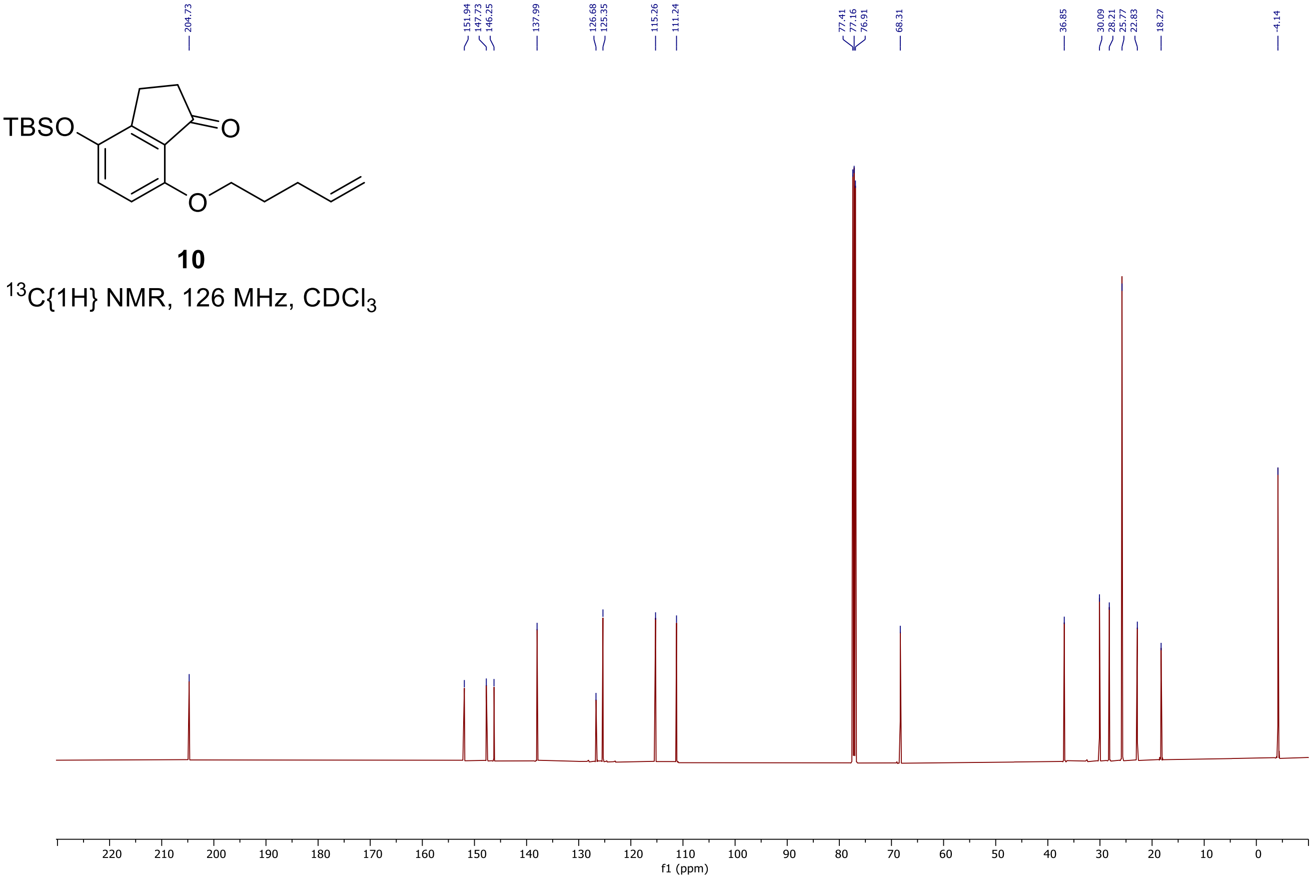

10

^{13}C{1H} NMR, 126 MHz, $CDCl_3$

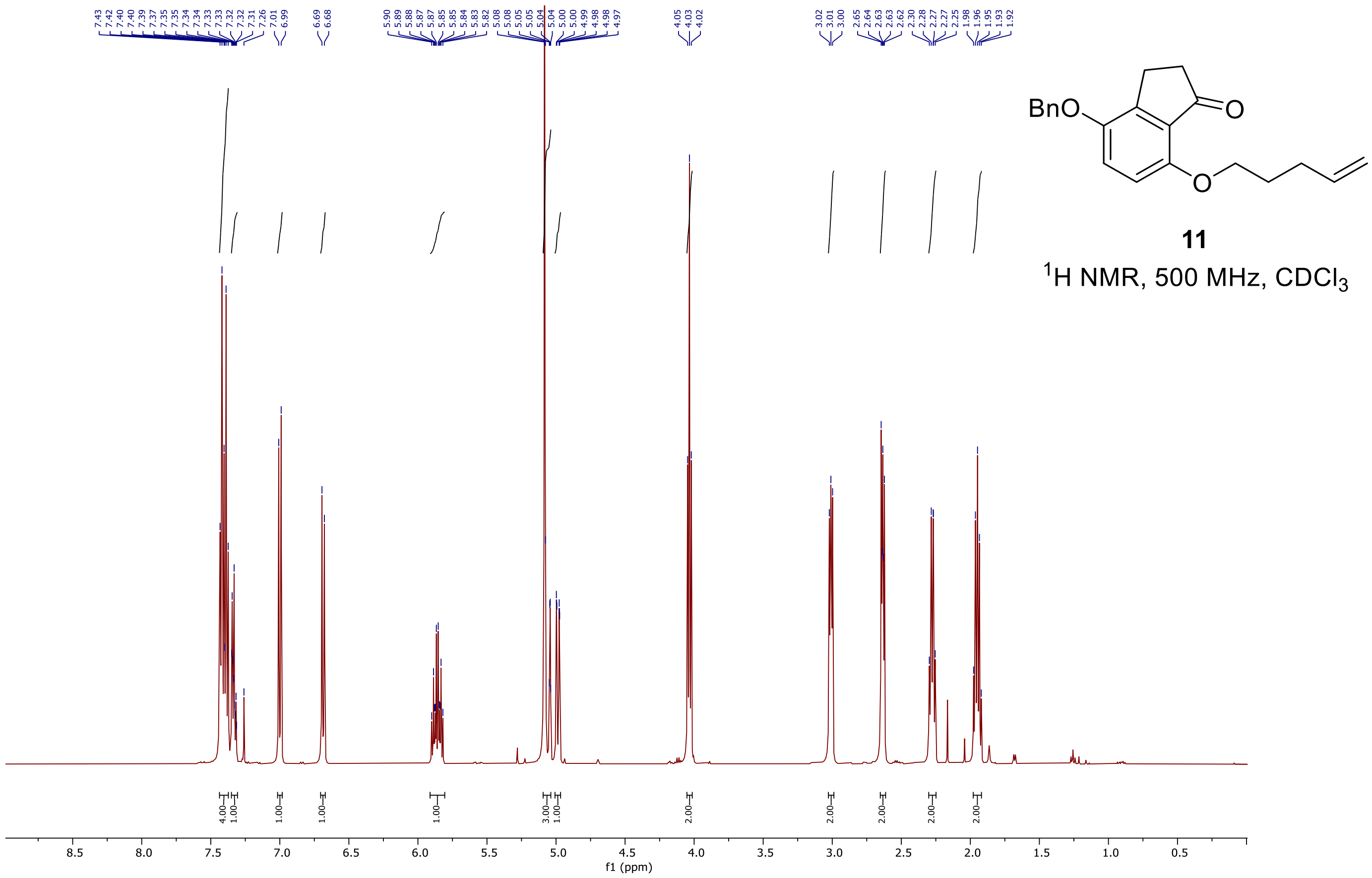

11

^{1}H NMR, 500 MHz, $CDCl_3$

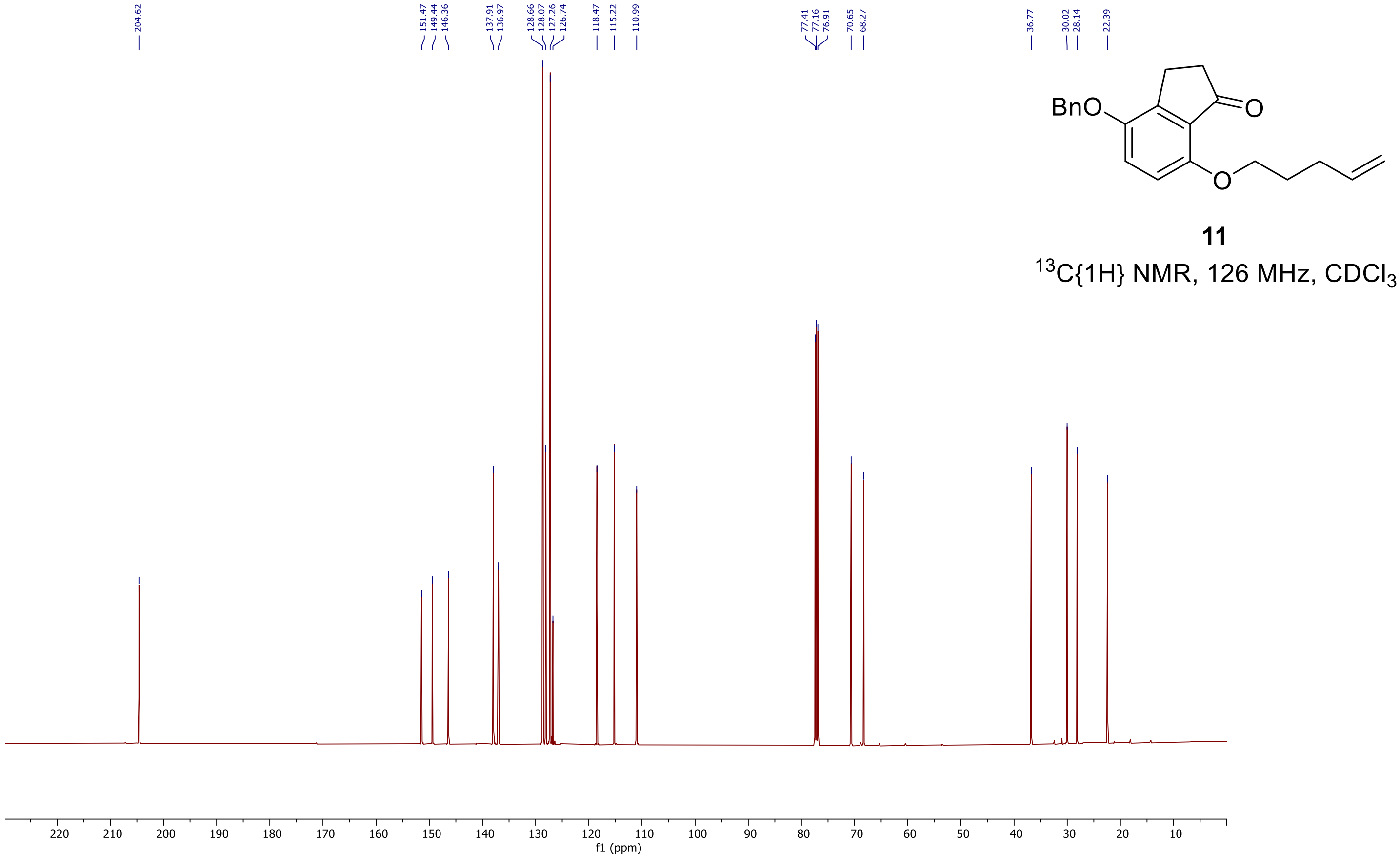

11

^{13}C{1H} NMR, 126 MHz, $CDCl_3$

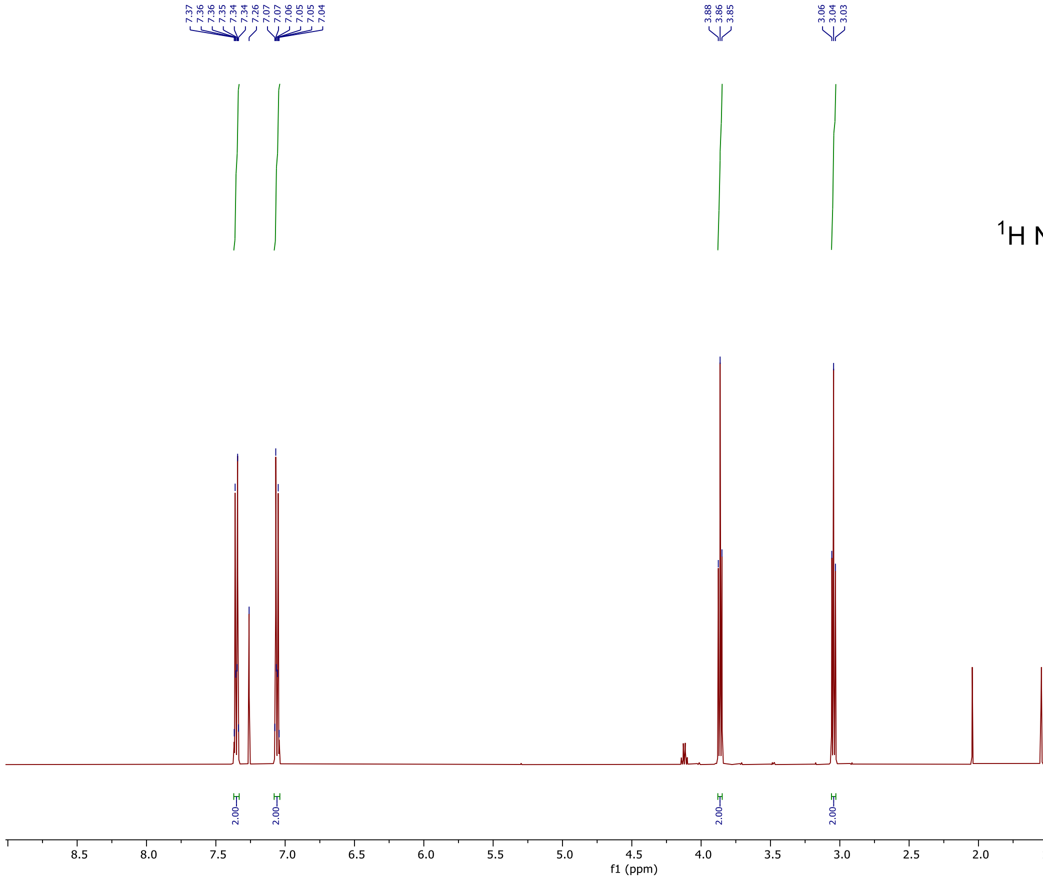

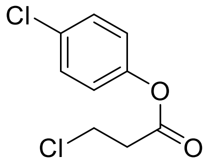

S1

^{1}H NMR, 500 MHz, $CDCl_3$

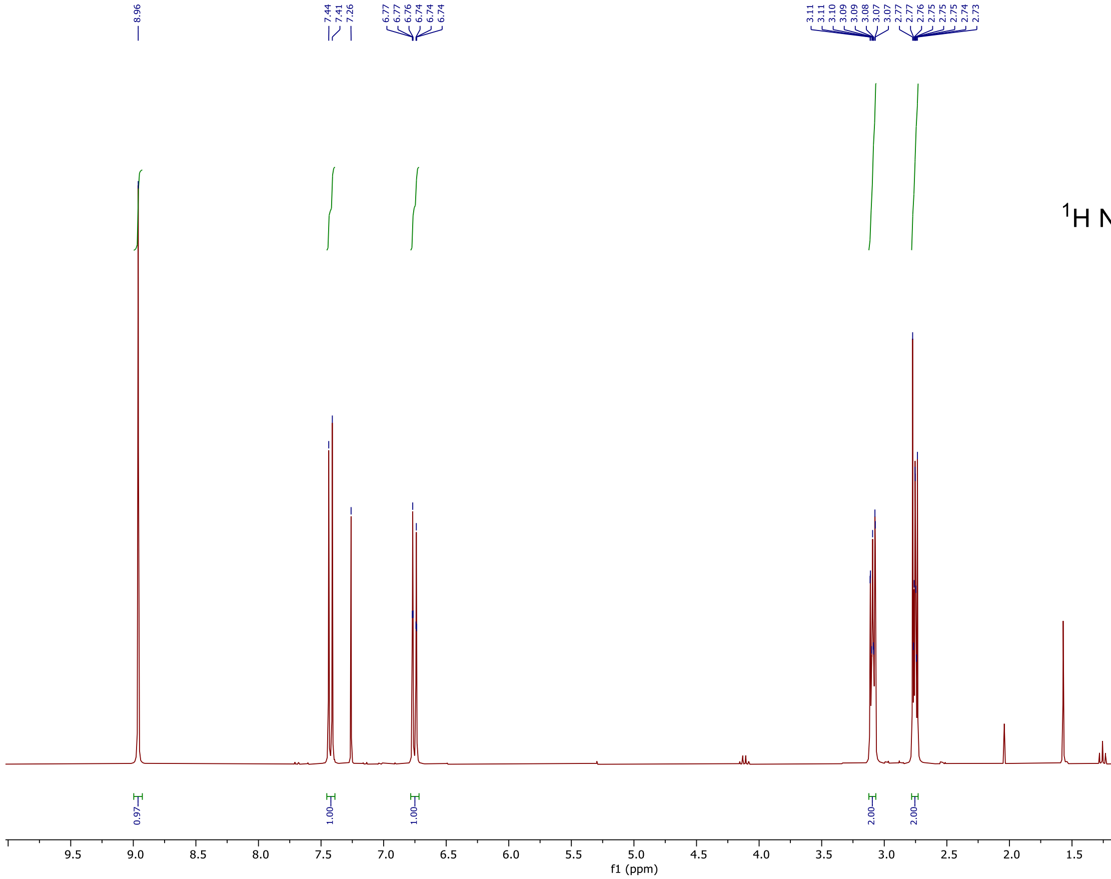

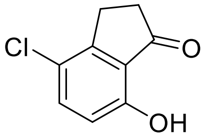

12

^{1}H NMR, 500 MHz, $CDCl_3$

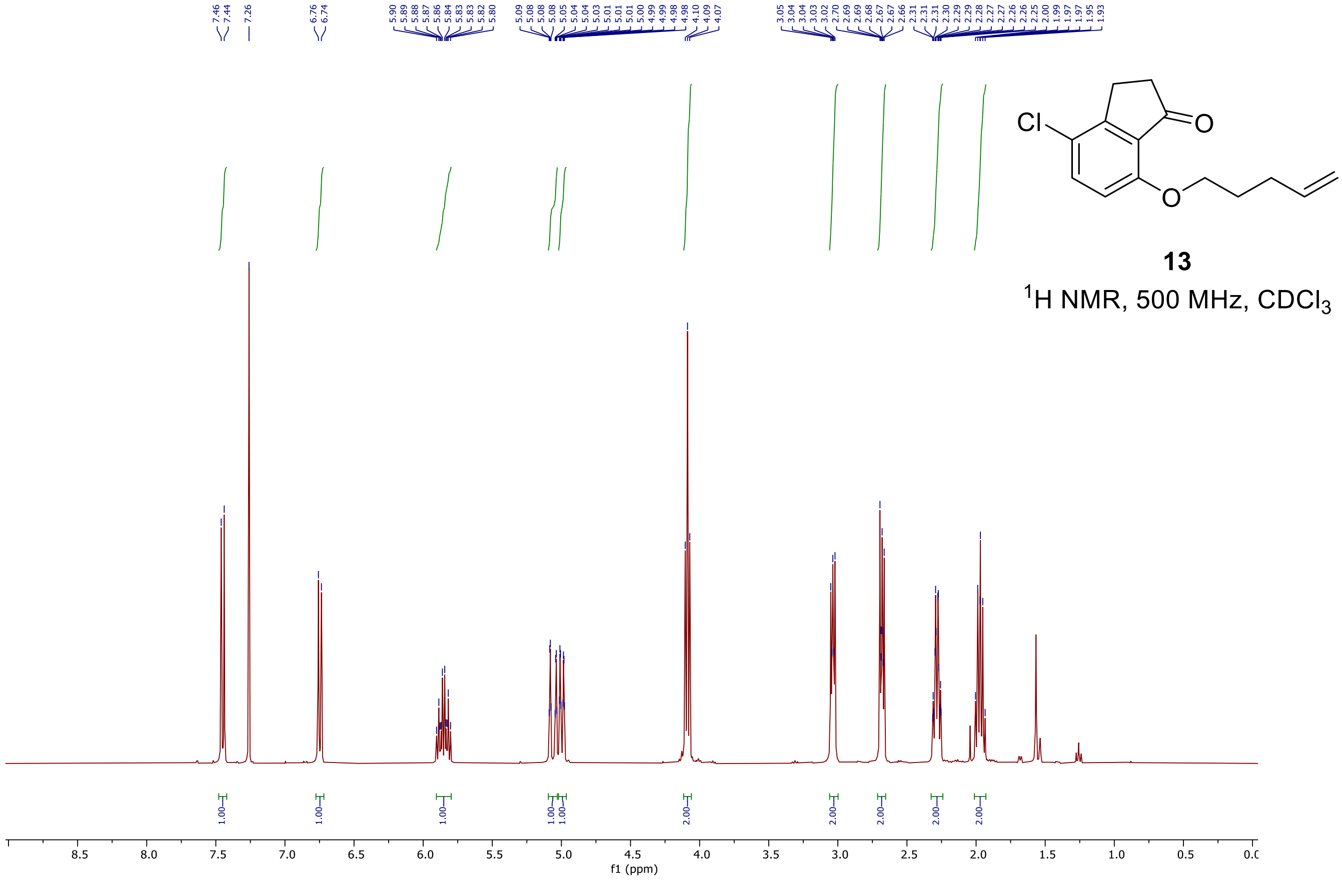

Cl
O
O
13
¹H NMR, 500 MHz, CDCl₃
f1 (ppm)

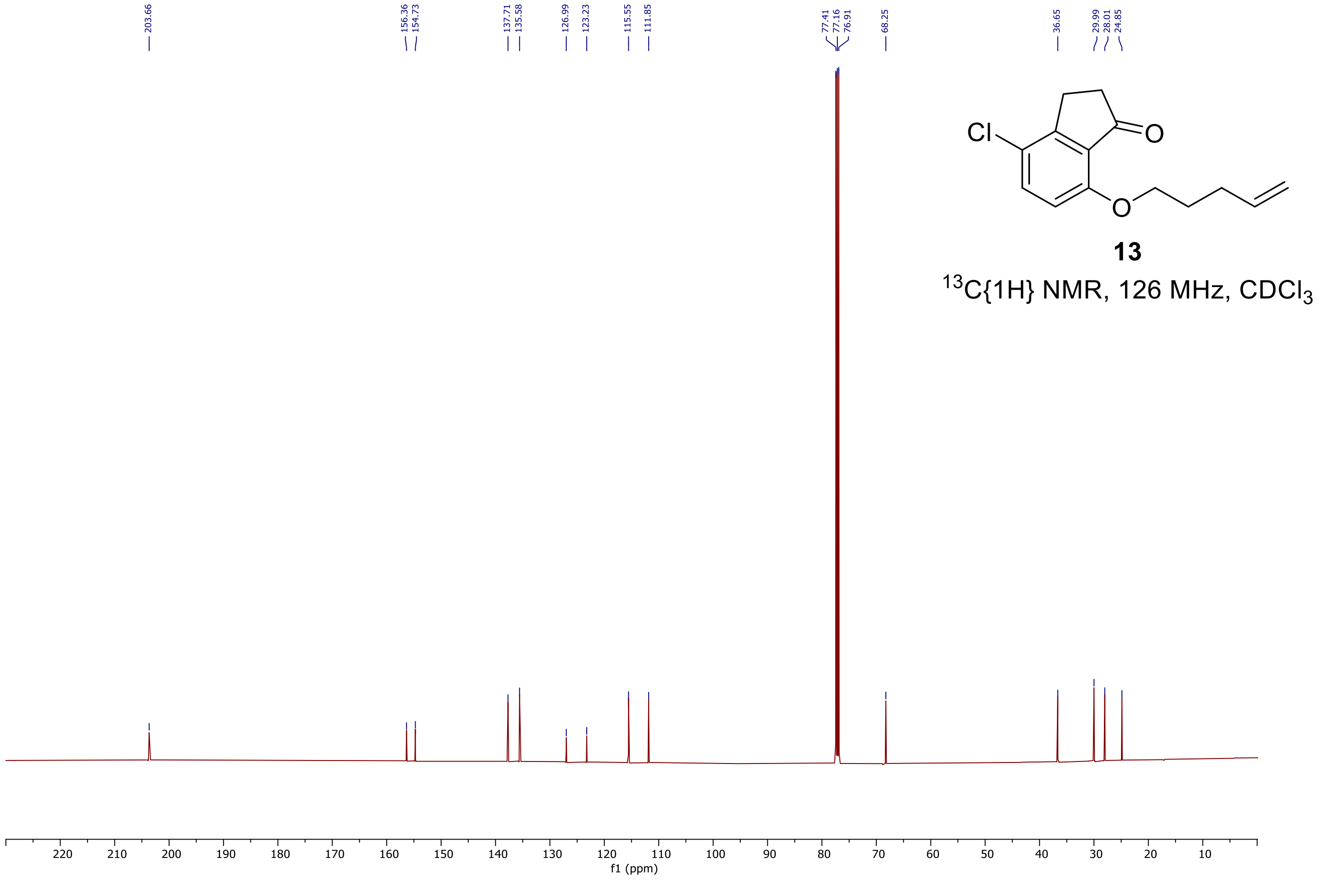

Cl
O
O
13
¹³C{1H} NMR, 126 MHz, CDCl₃
f1 (ppm)

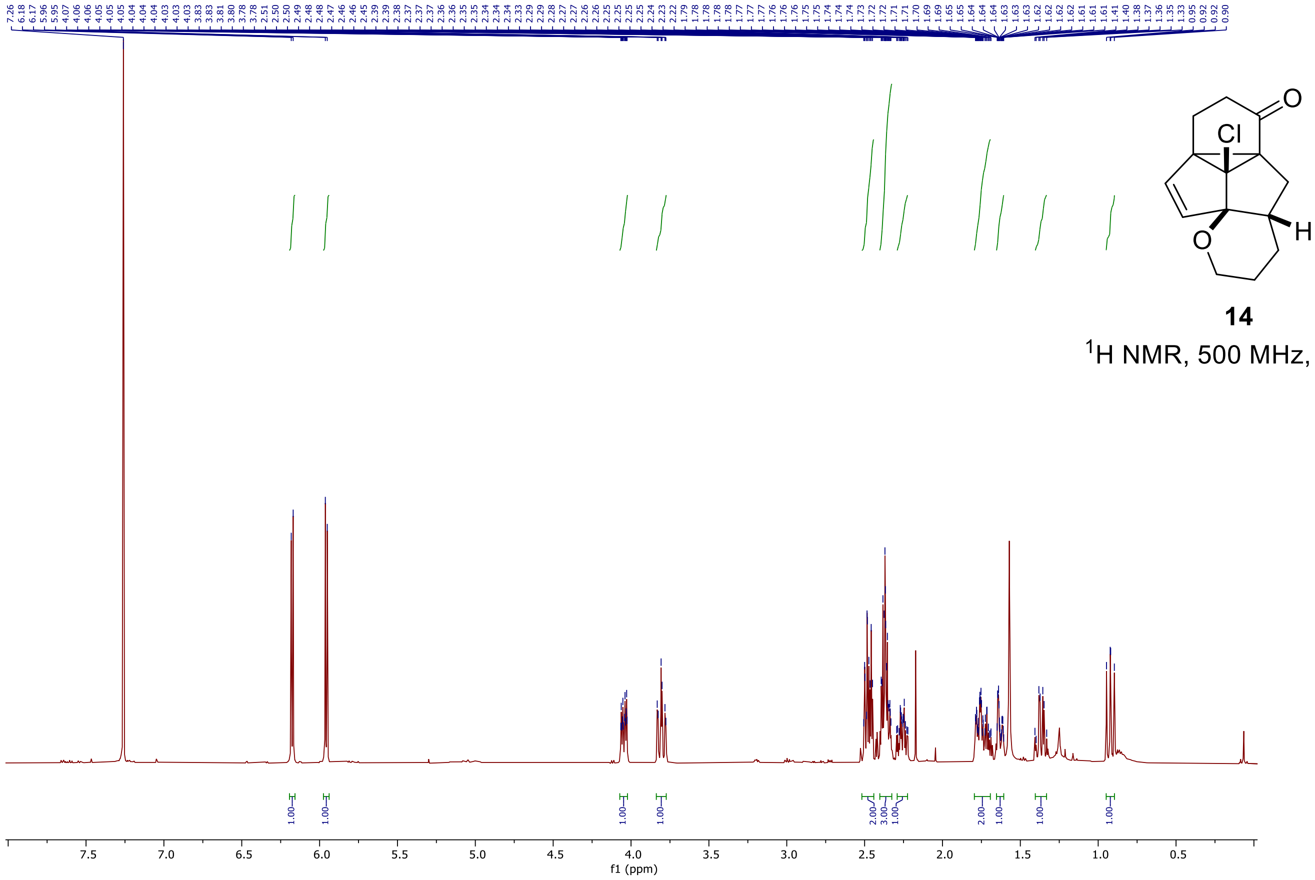

14

^{1}H NMR, 500 MHz, $CDCl_3$

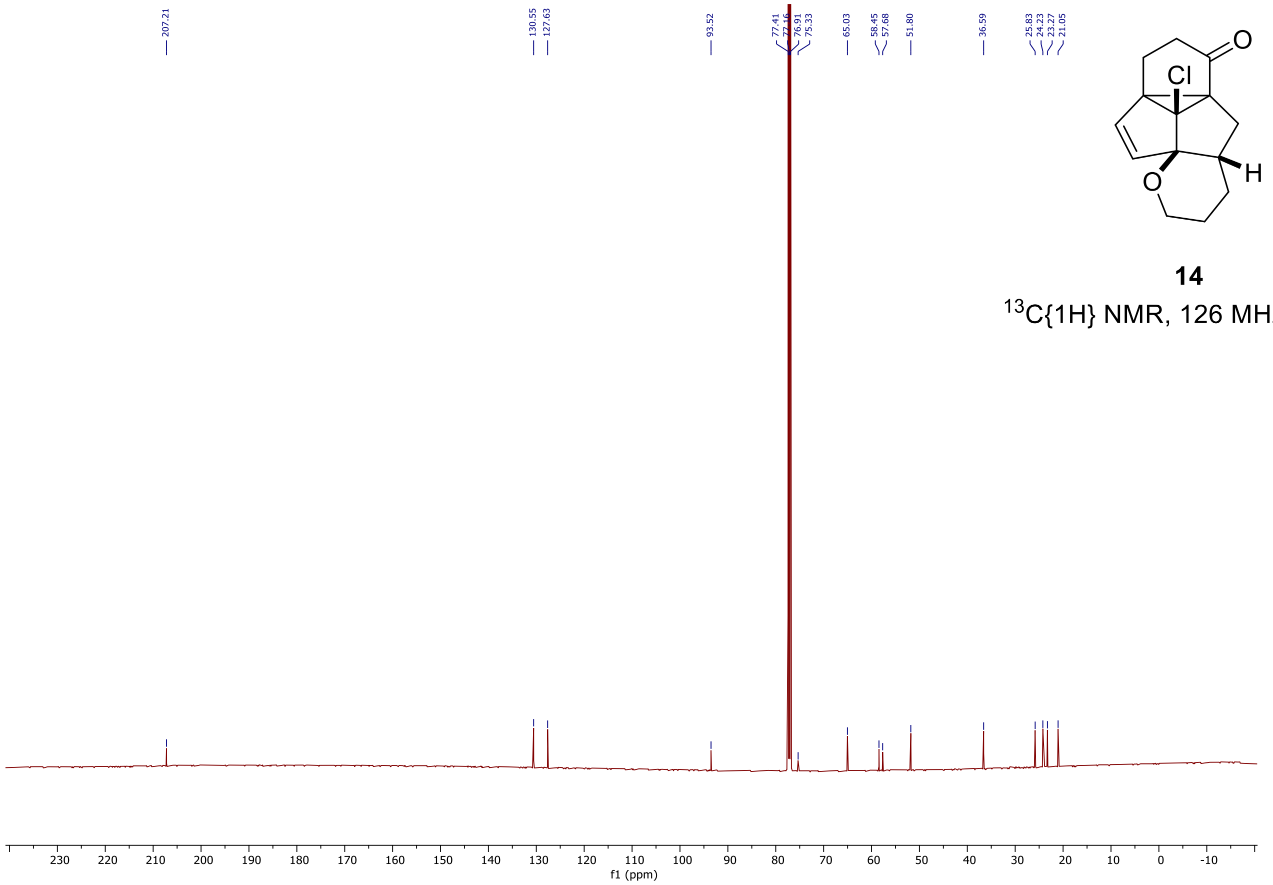

14

^{13}C{1H} NMR, 126 MHz, $CDCl_3$

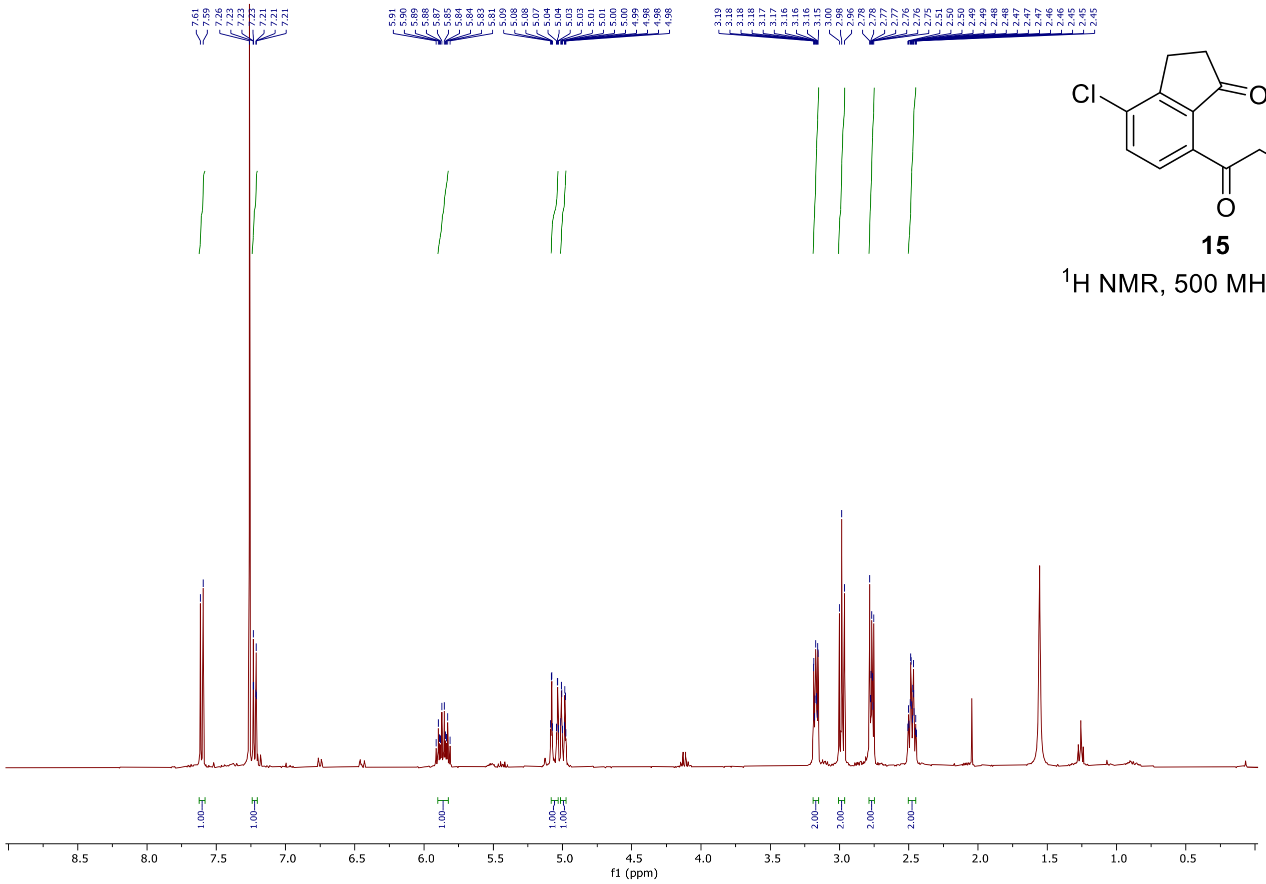

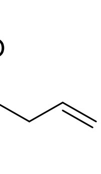

15

^{1}H NMR, 500 MHz, $CDCl_3$

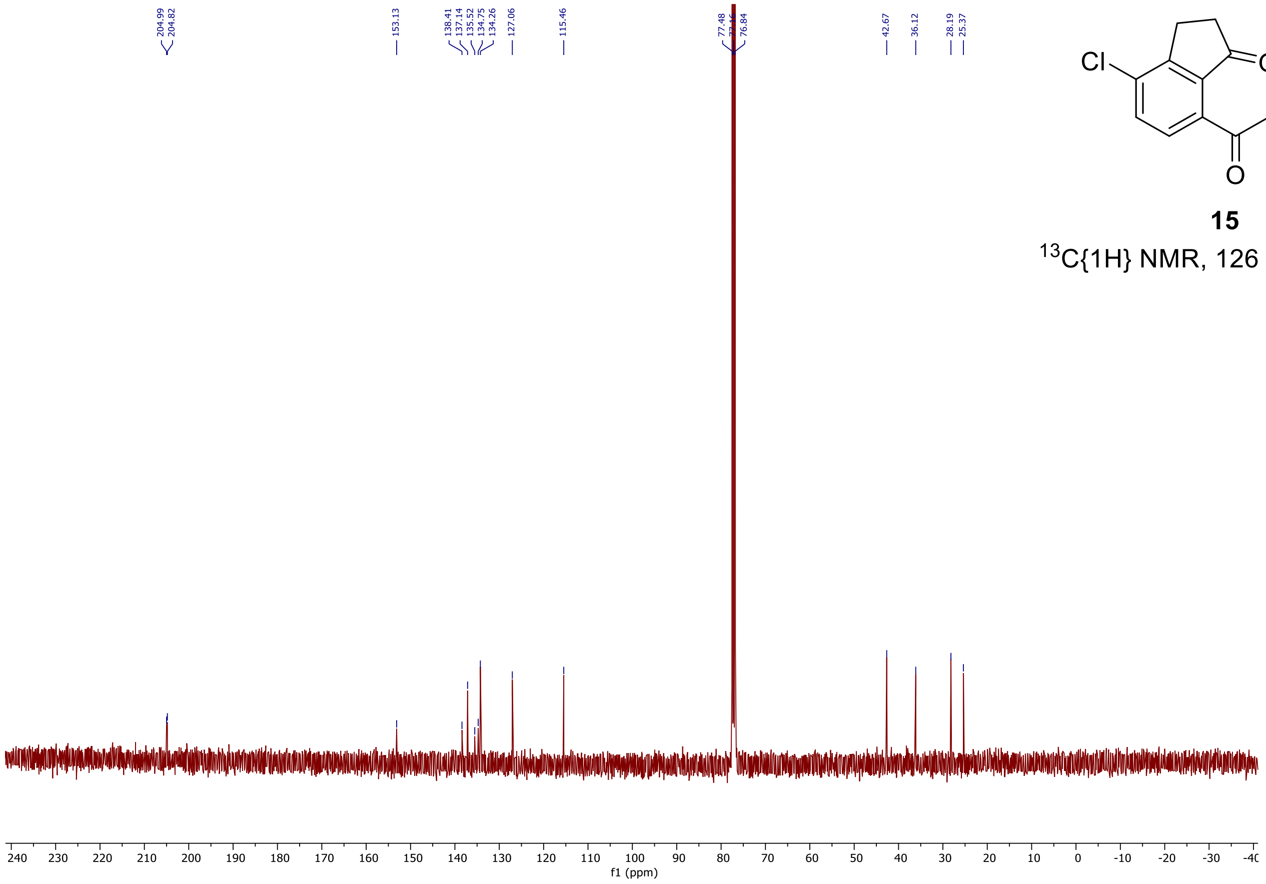

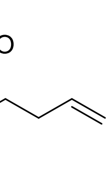

15

$^{13}C\{1H\}$ NMR, 126 MHz, $CDCl_3$

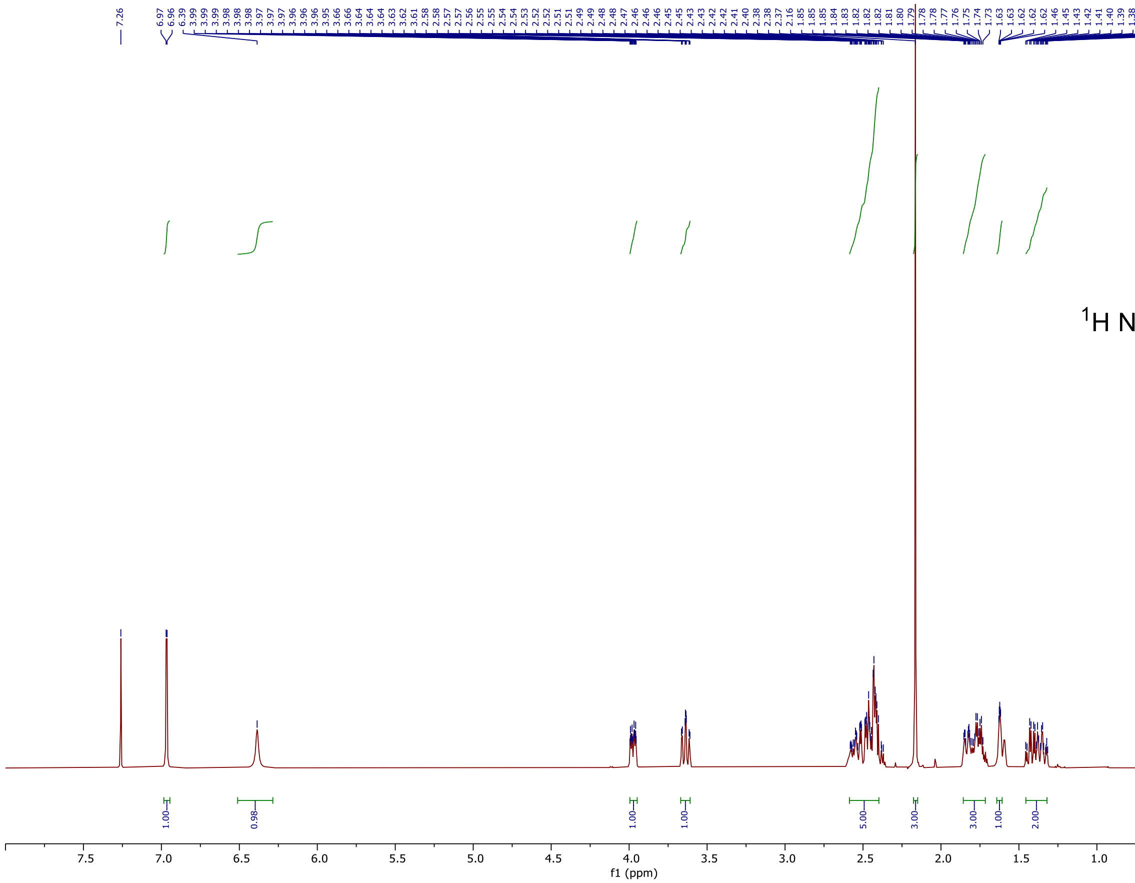

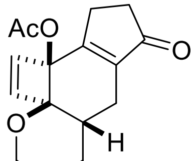

18

^{1}H NMR, 500 MHz, $CDCl_3$

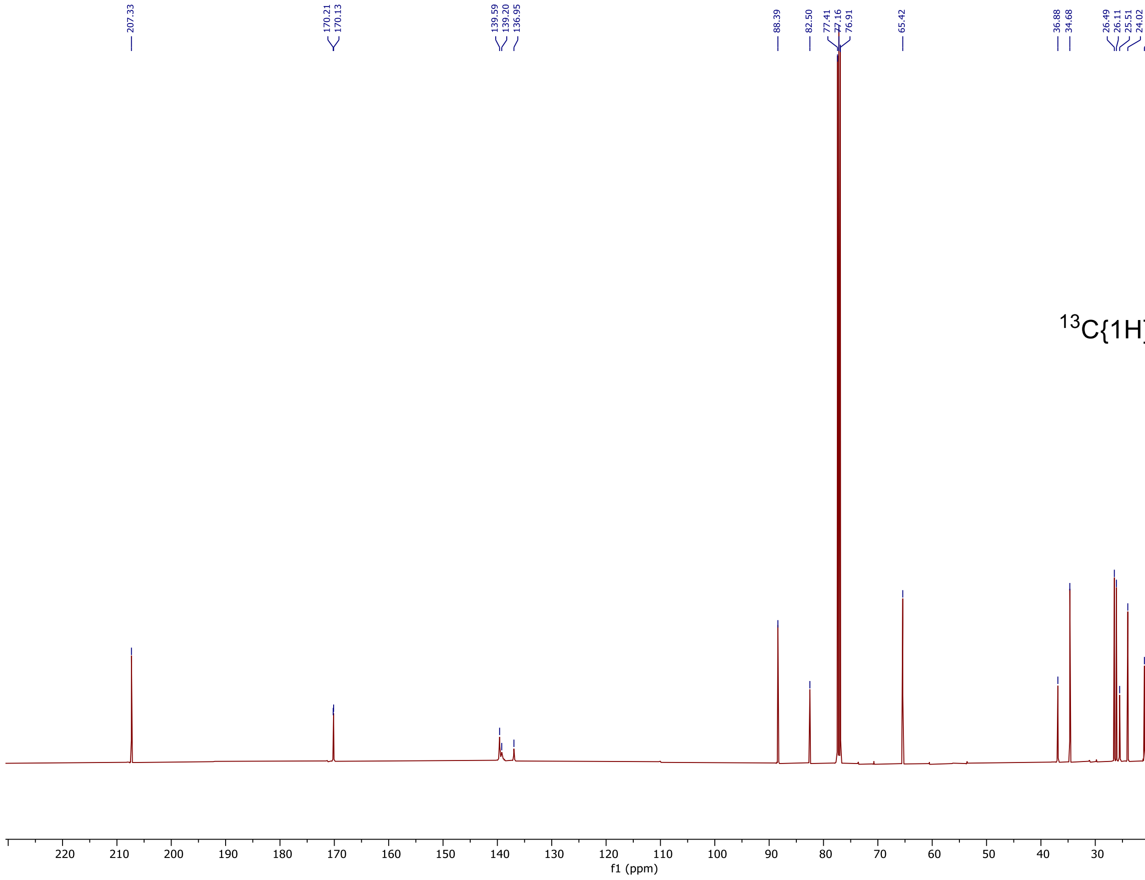

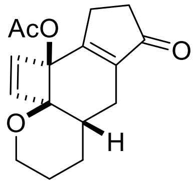

18

^{13}C{1H} NMR, 126 MHz, $CDCl_3$

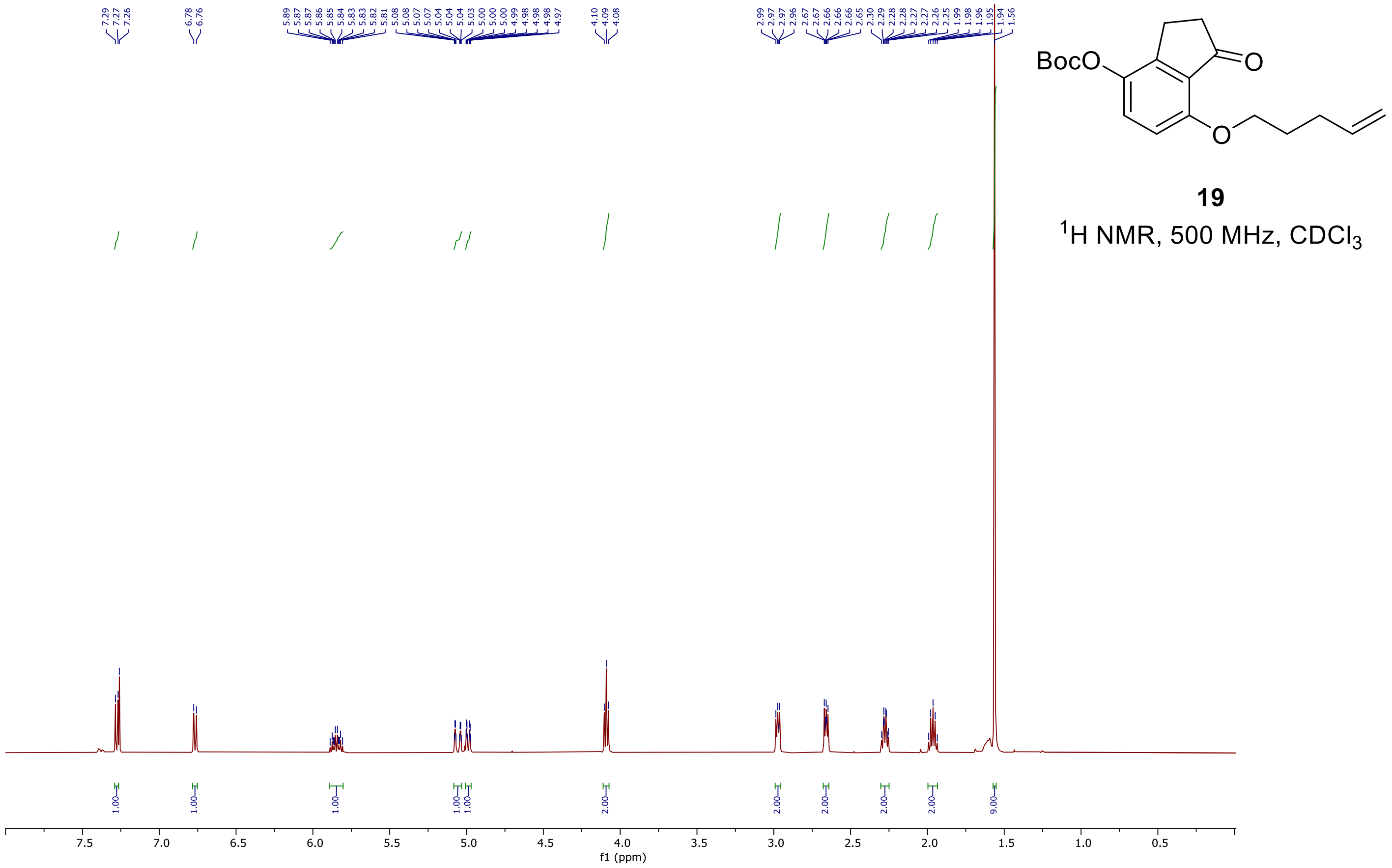

19

^{1}H NMR, 500 MHz, $CDCl_3$

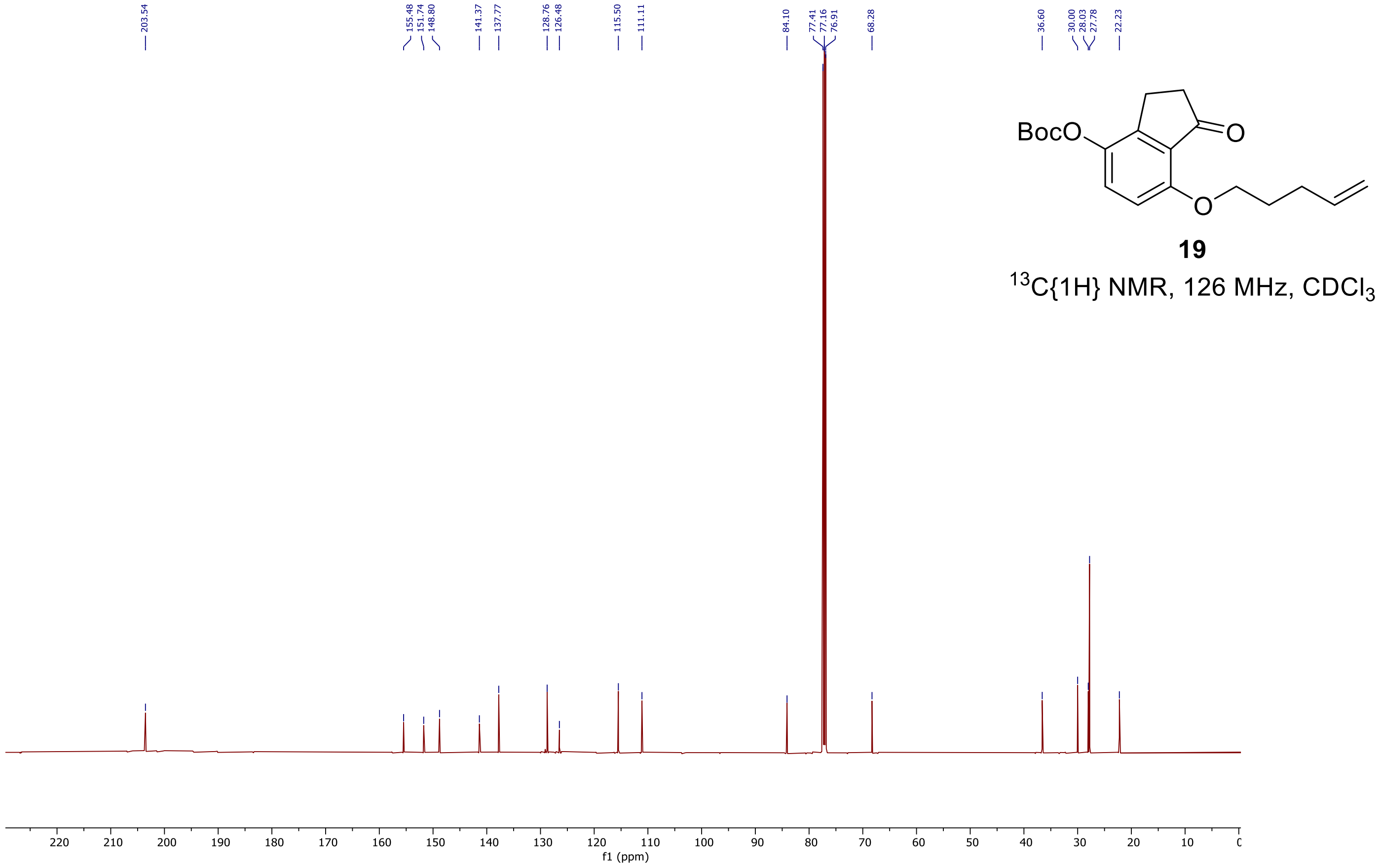

19

^{13}C{1H} NMR, 126 MHz, $CDCl_3$

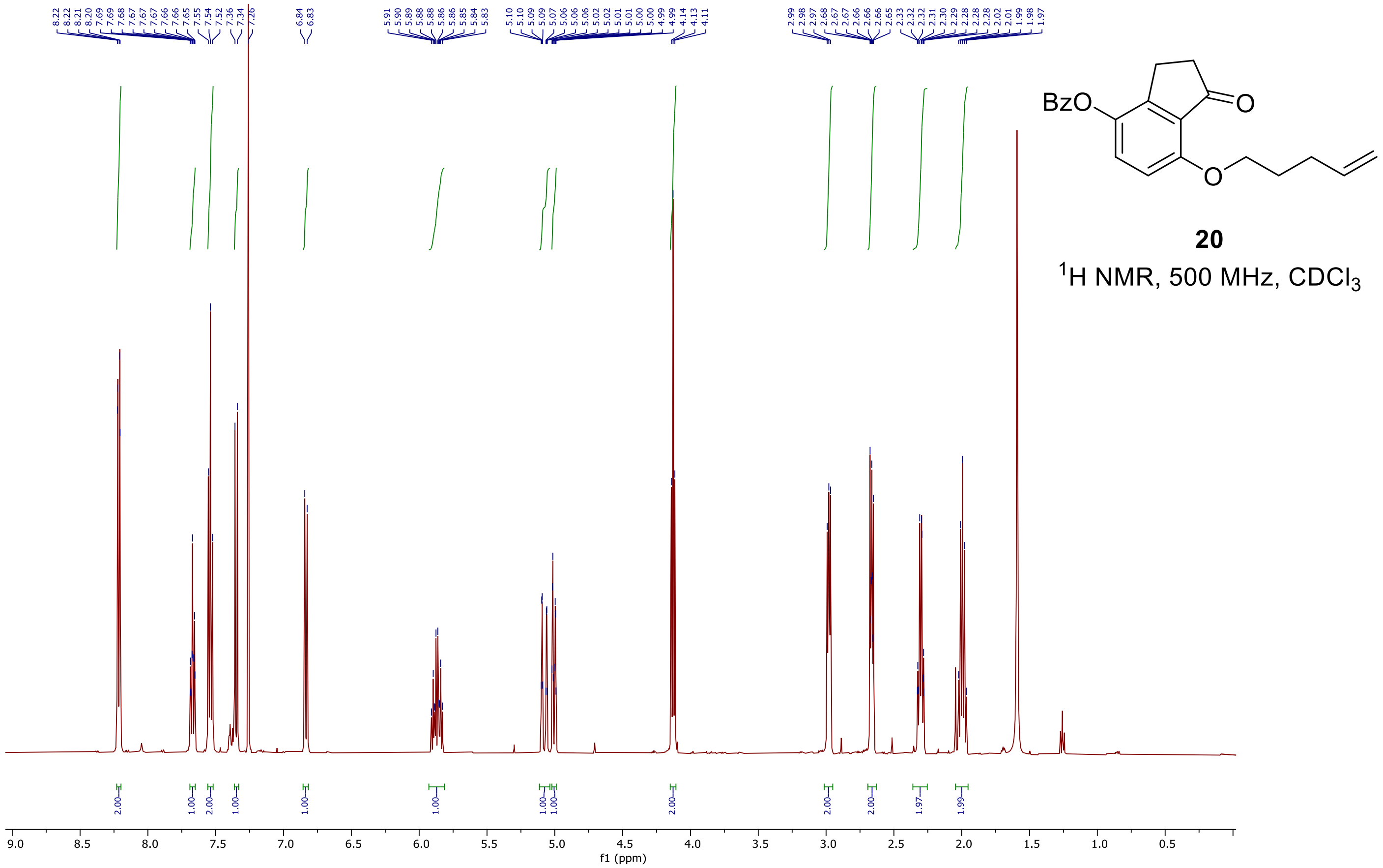

20

^{1}H NMR, 500 MHz, $CDCl_3$

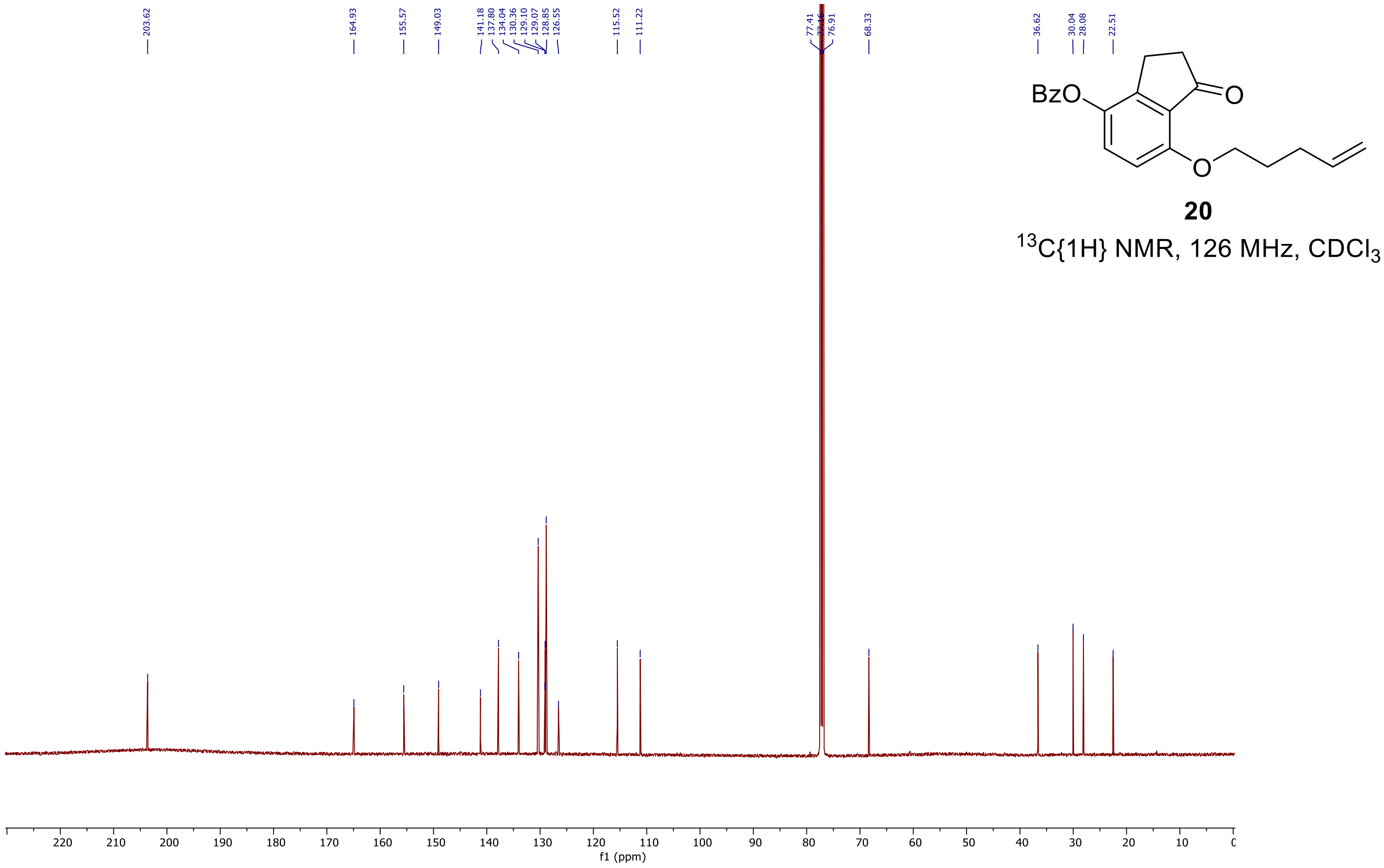

20

$^{13}C\{1H\}$ NMR, 126 MHz, $CDCl_3$

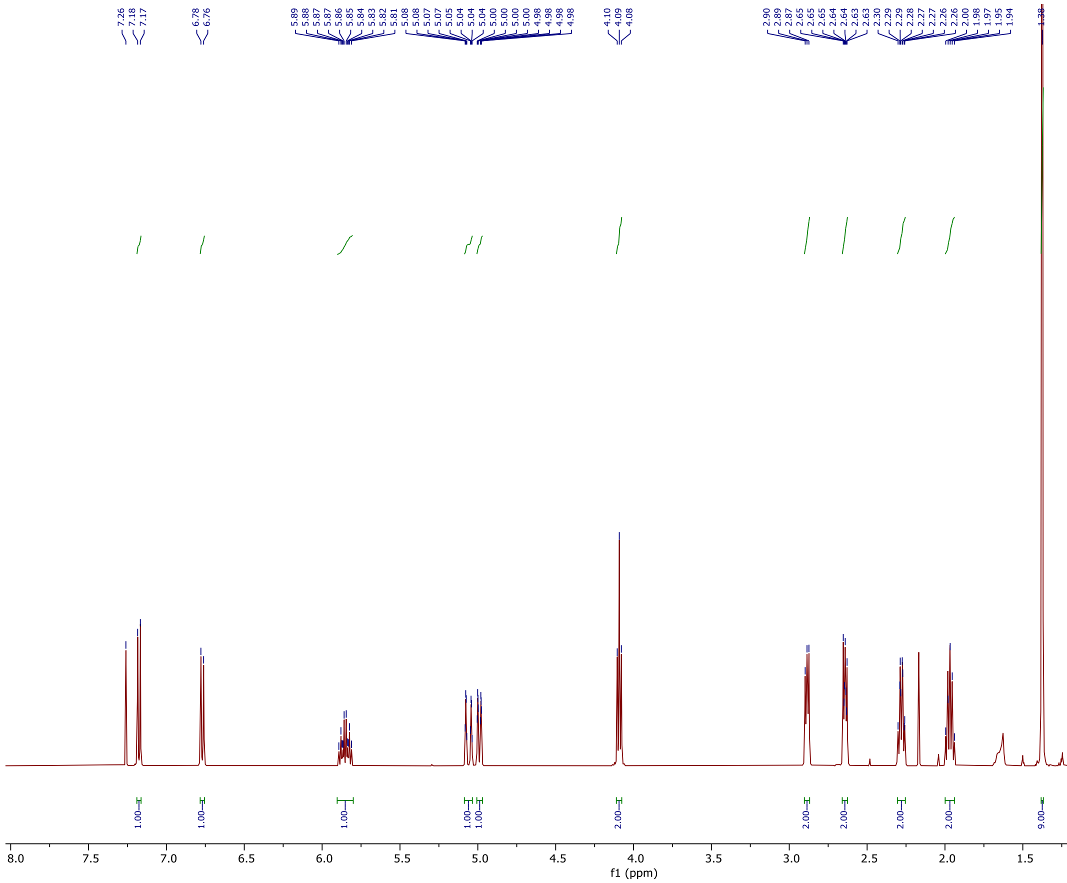

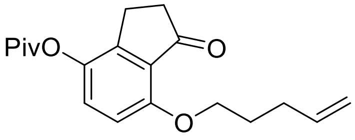

21

^{1}H NMR, 500 MHz, $CDCl_3$

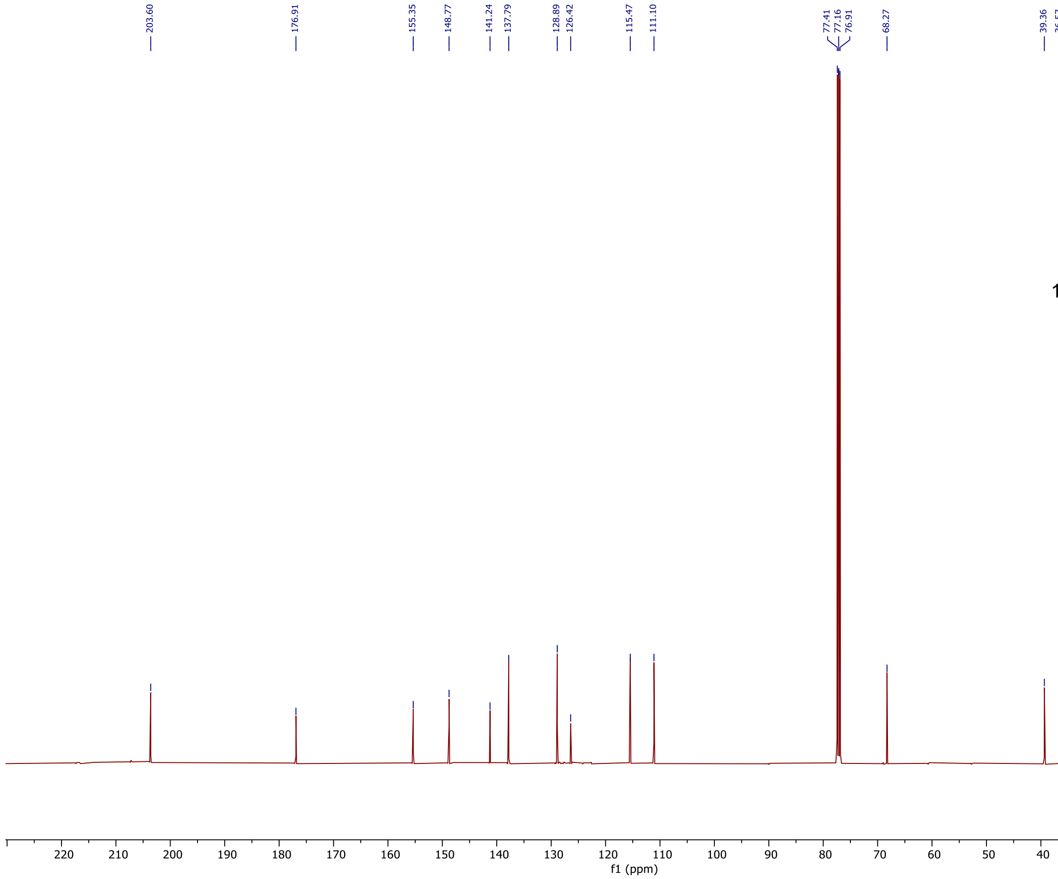

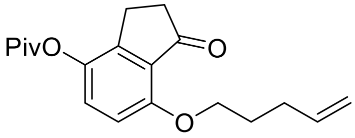

21

^{13}C{1H} NMR, 126 MHz, $CDCl_3$

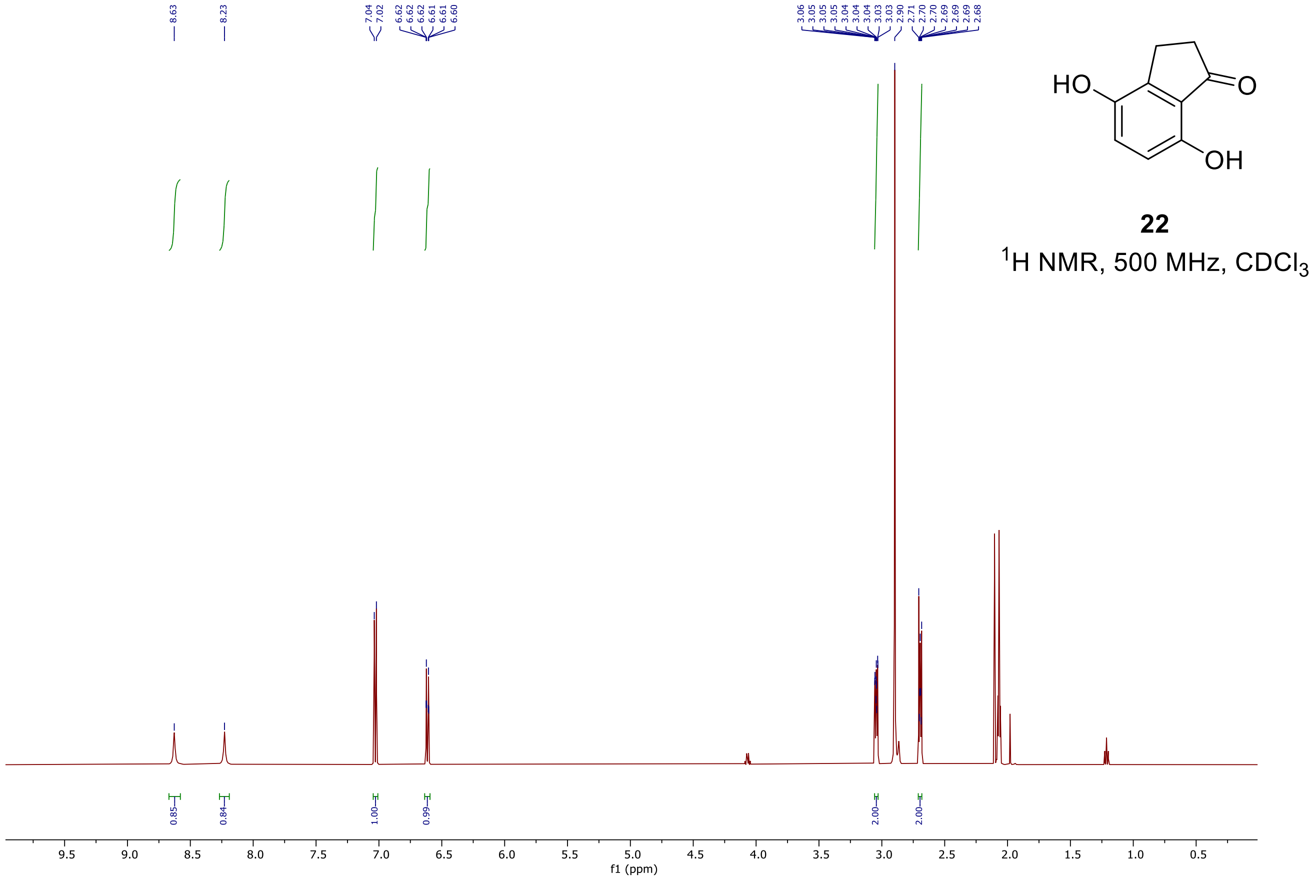

22

^{1}H NMR, 500 MHz, $CDCl_3$

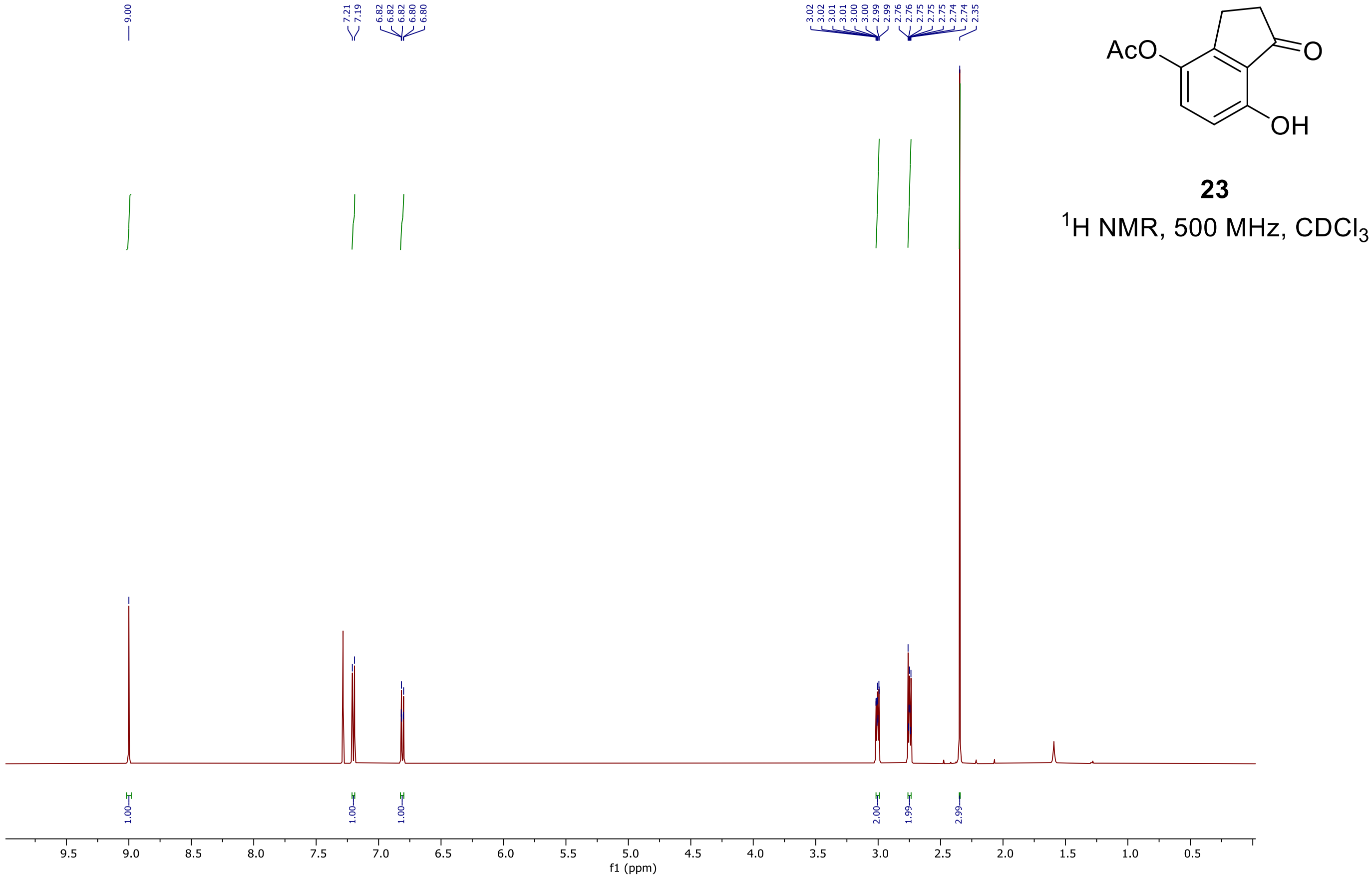

23

^{1}H NMR, 500 MHz, $CDCl_3$

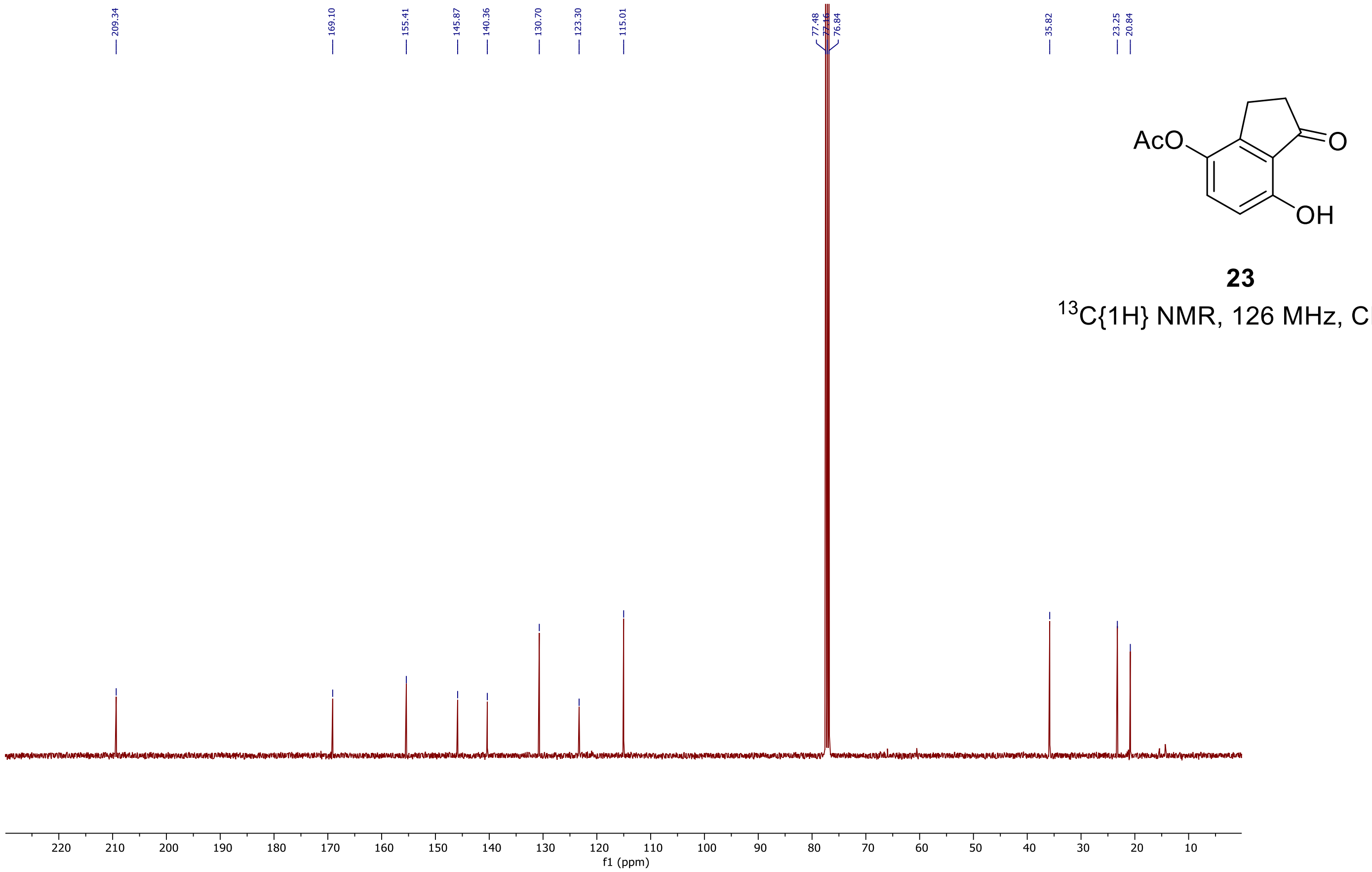

23
$^{13}C\{1H\}$ NMR, 126 MHz, $CDCl_3$

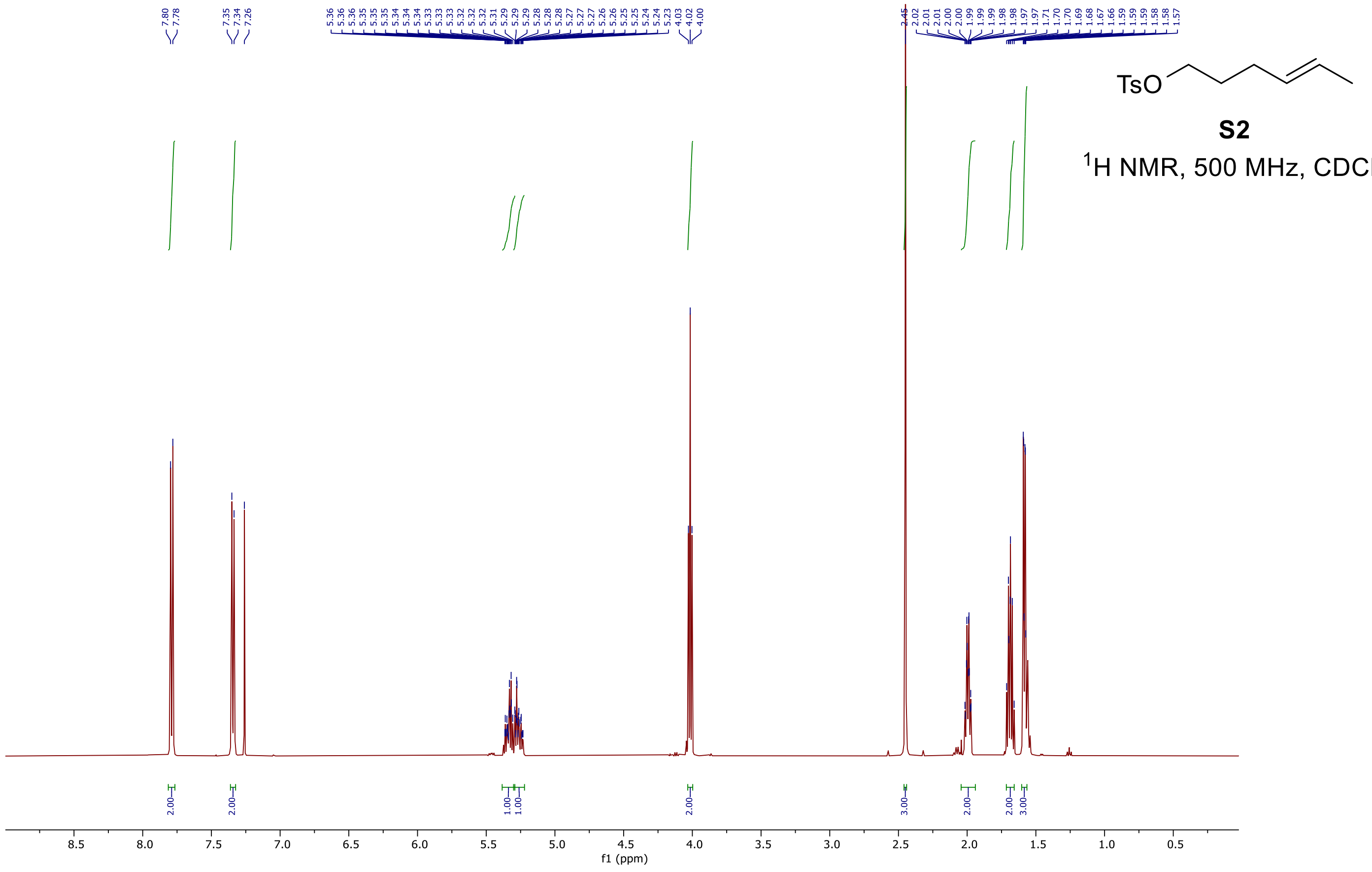

S2
^{1}H NMR, 500 MHz, $CDCl_3$

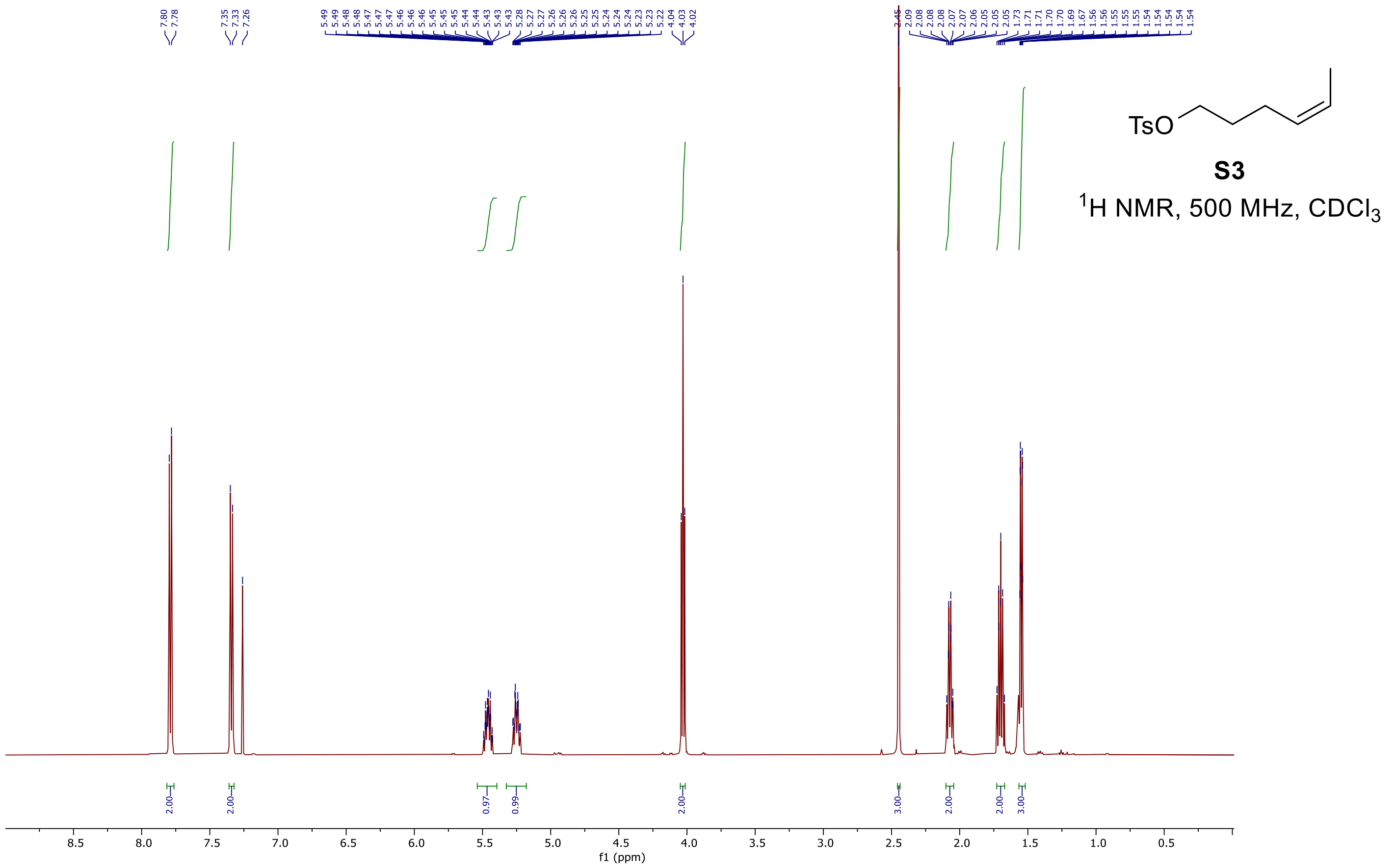

S3

^{1}H NMR, 500 MHz, $CDCl_3$

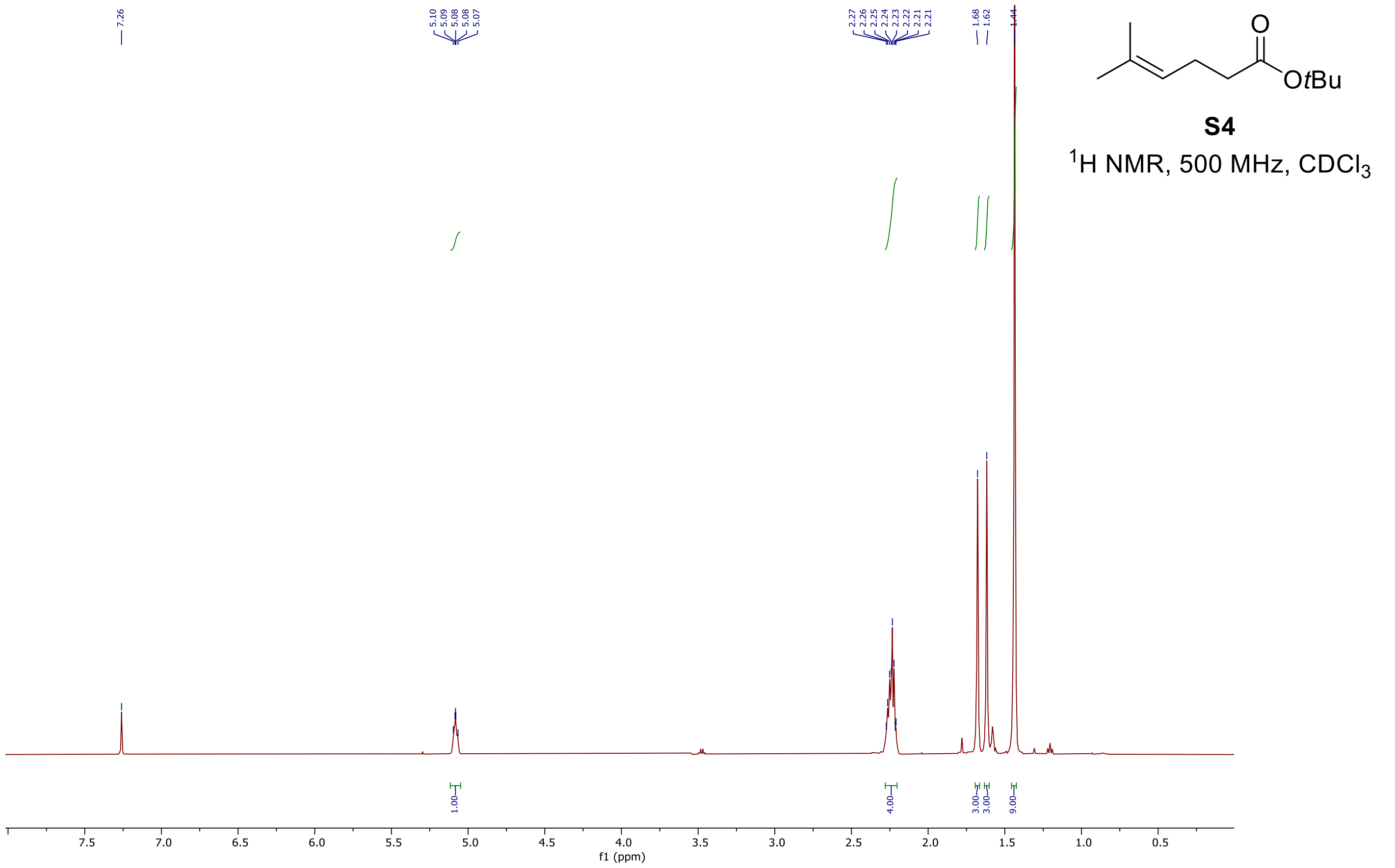

S4

^{1}H NMR, 500 MHz, $CDCl_3$

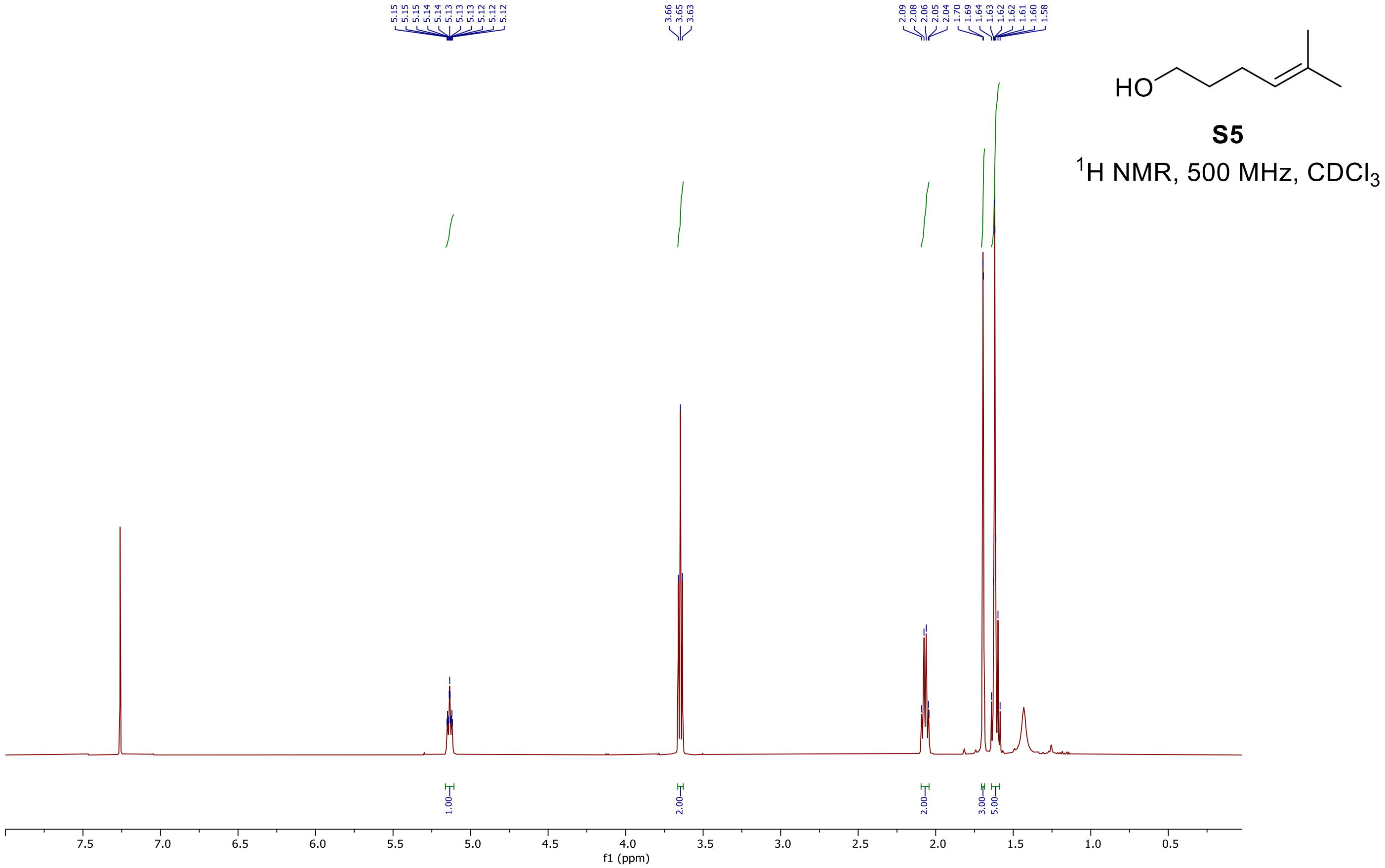

S5

^{1}H NMR, 500 MHz, $CDCl_3$

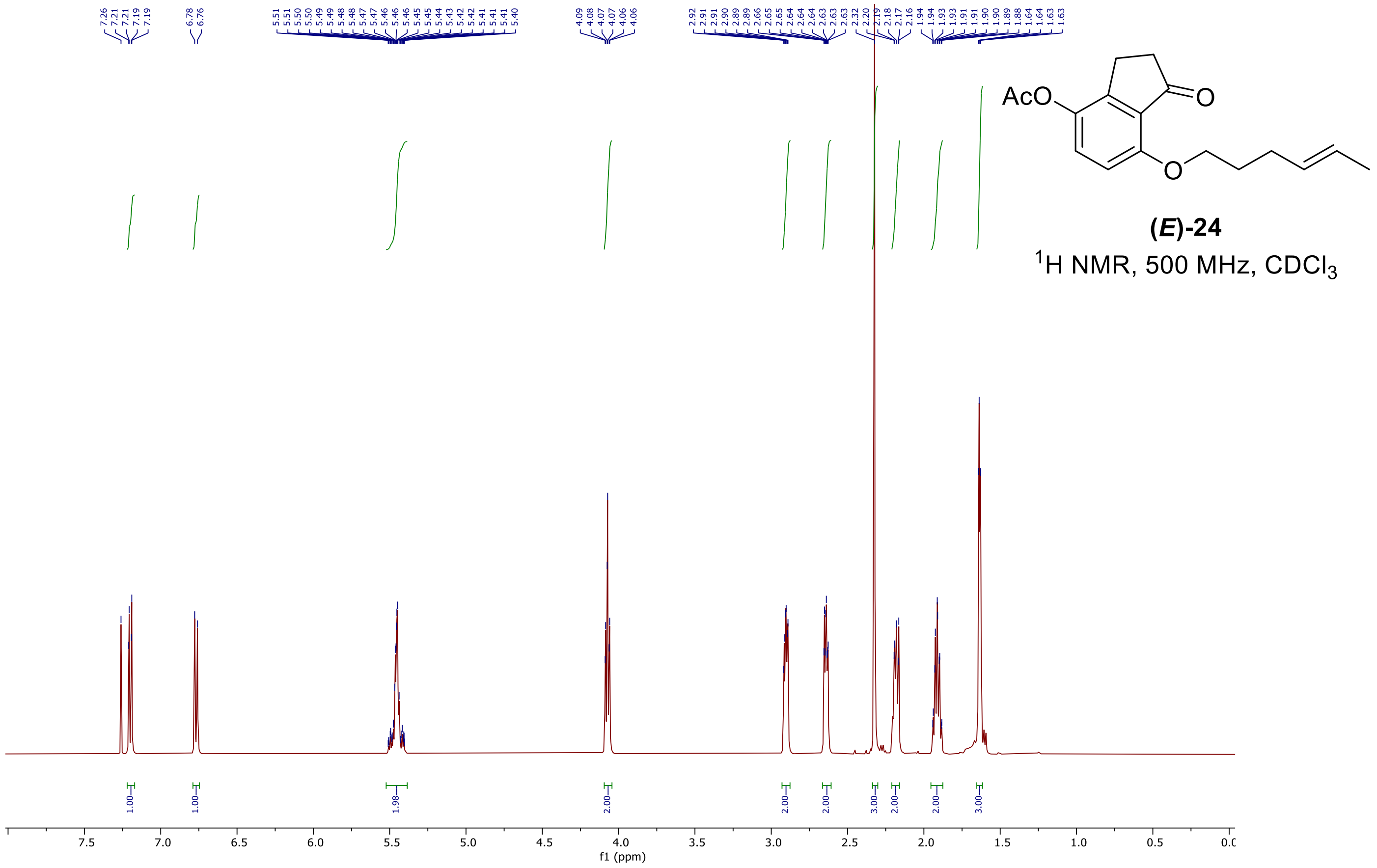

(*E*)-24

^{1}H NMR, 500 MHz, $CDCl_3$

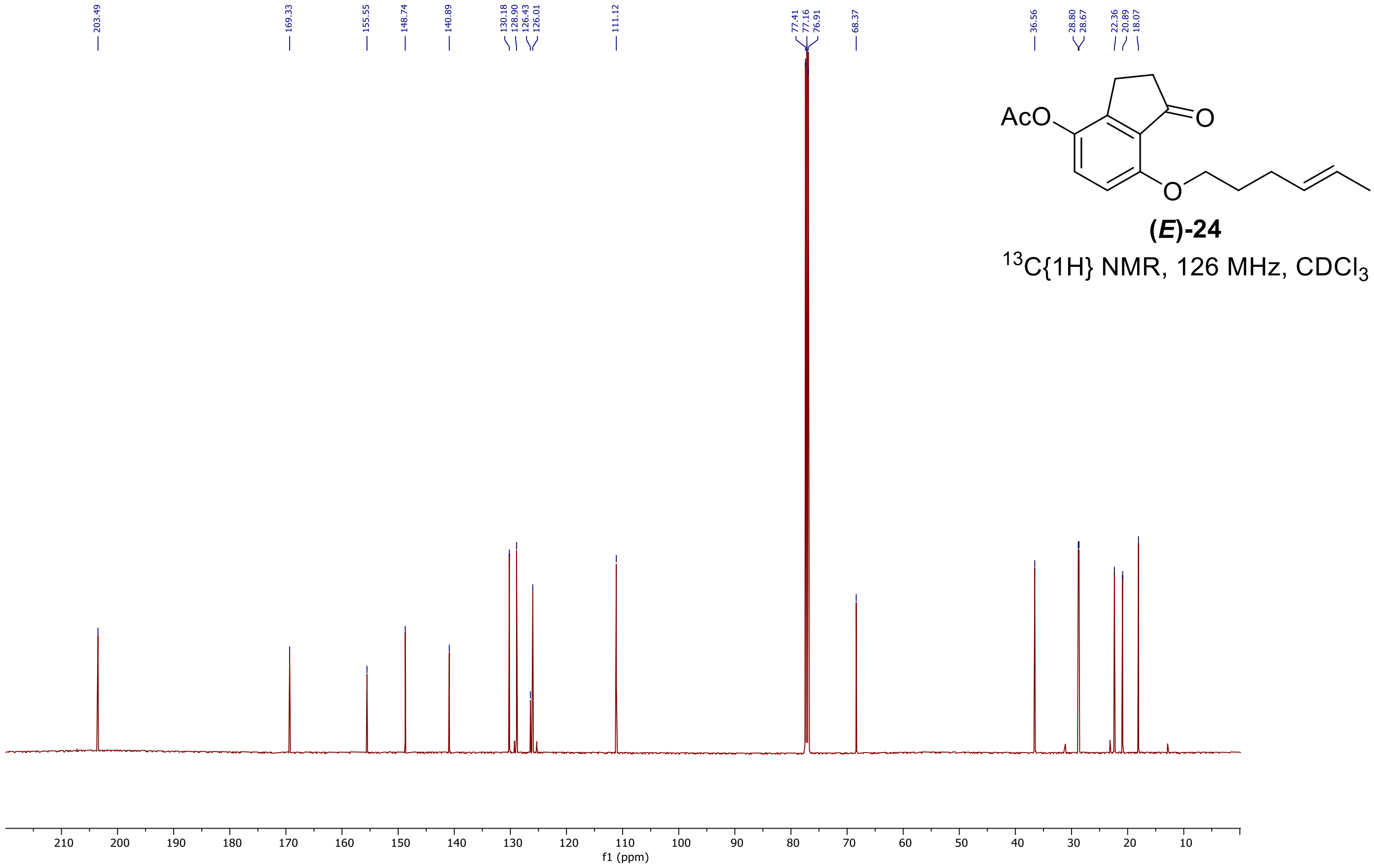

AcO
O
O
(E)-24
^{13}C{1H} NMR, 126 MHz, CDCl$_3$
f1 (ppm)

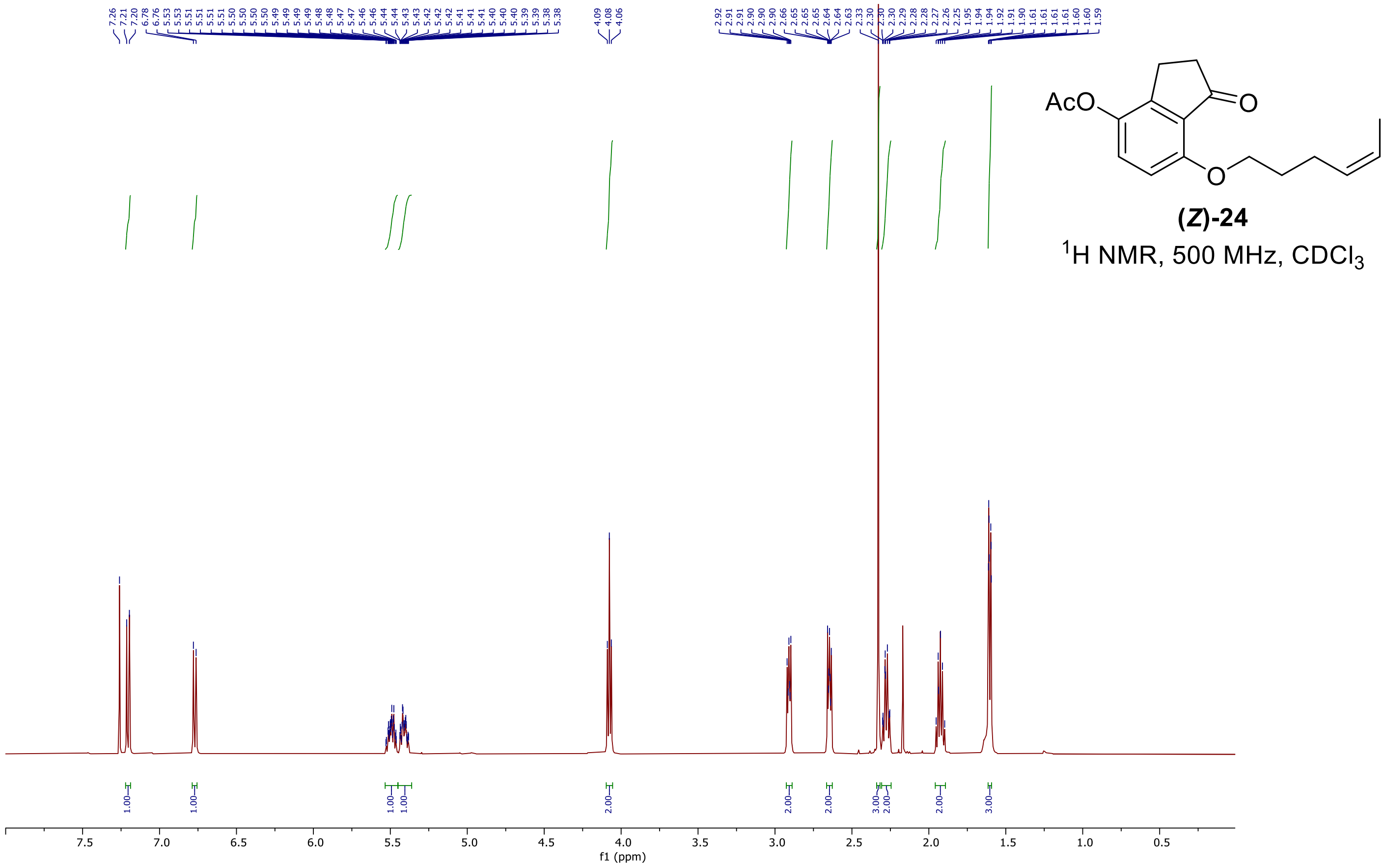

AcO
O
O
(Z)-24
^{1}H NMR, 500 MHz, CDCl$_3$
f1 (ppm)

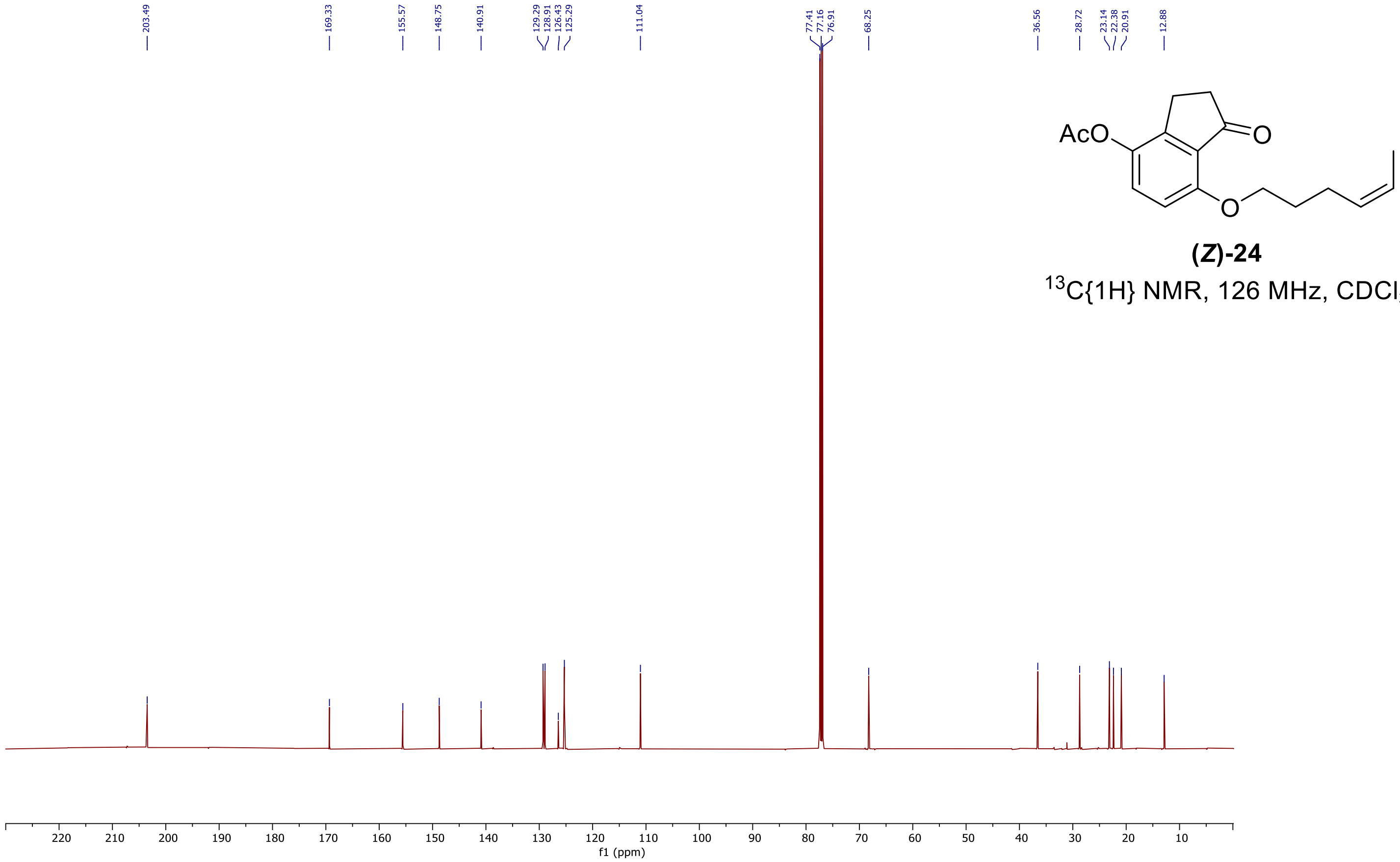

(*Z*)-24

^{13}C{1H} NMR, 126 MHz, $CDCl_3$

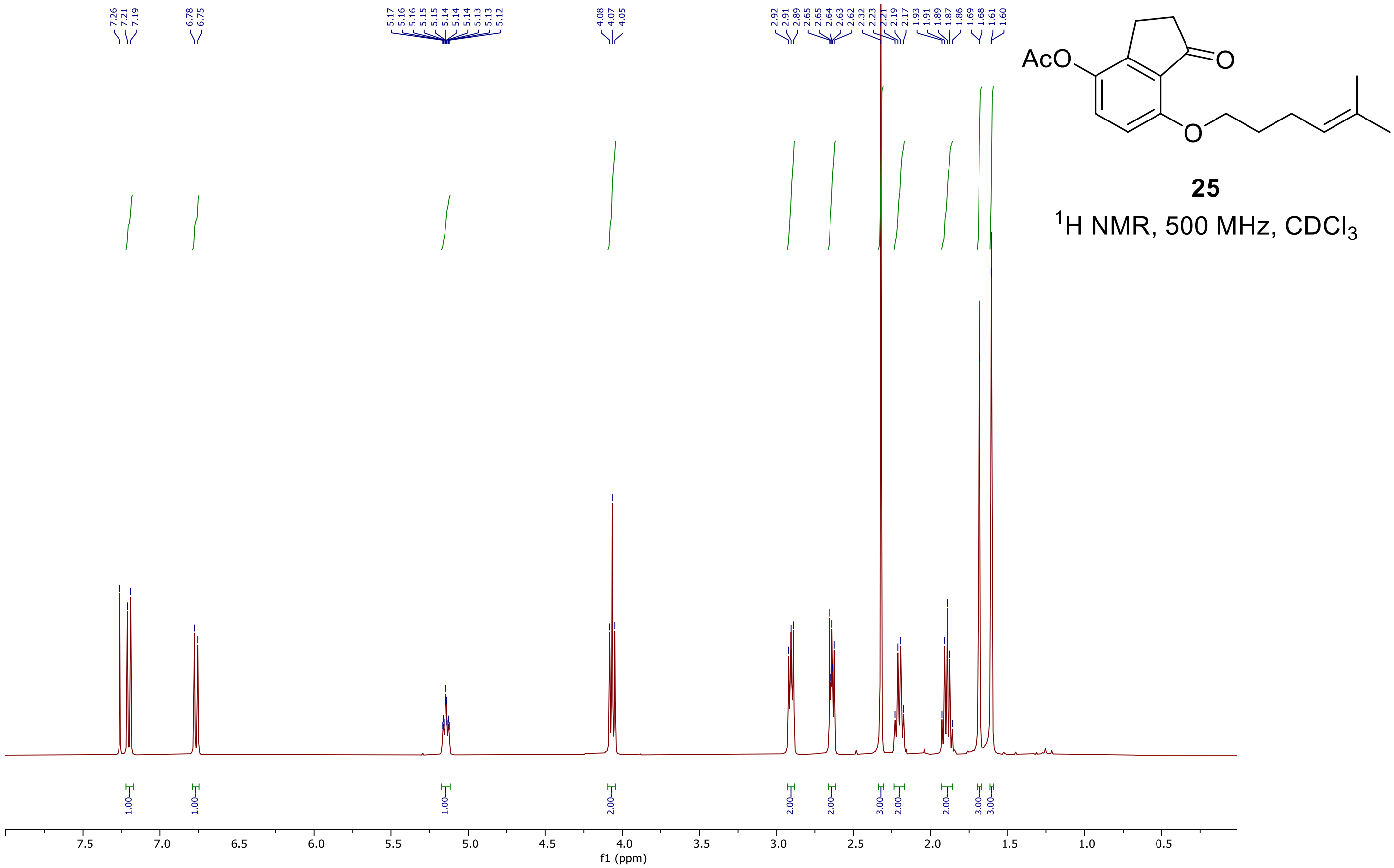

25

^{1}H NMR, 500 MHz, $CDCl_3$

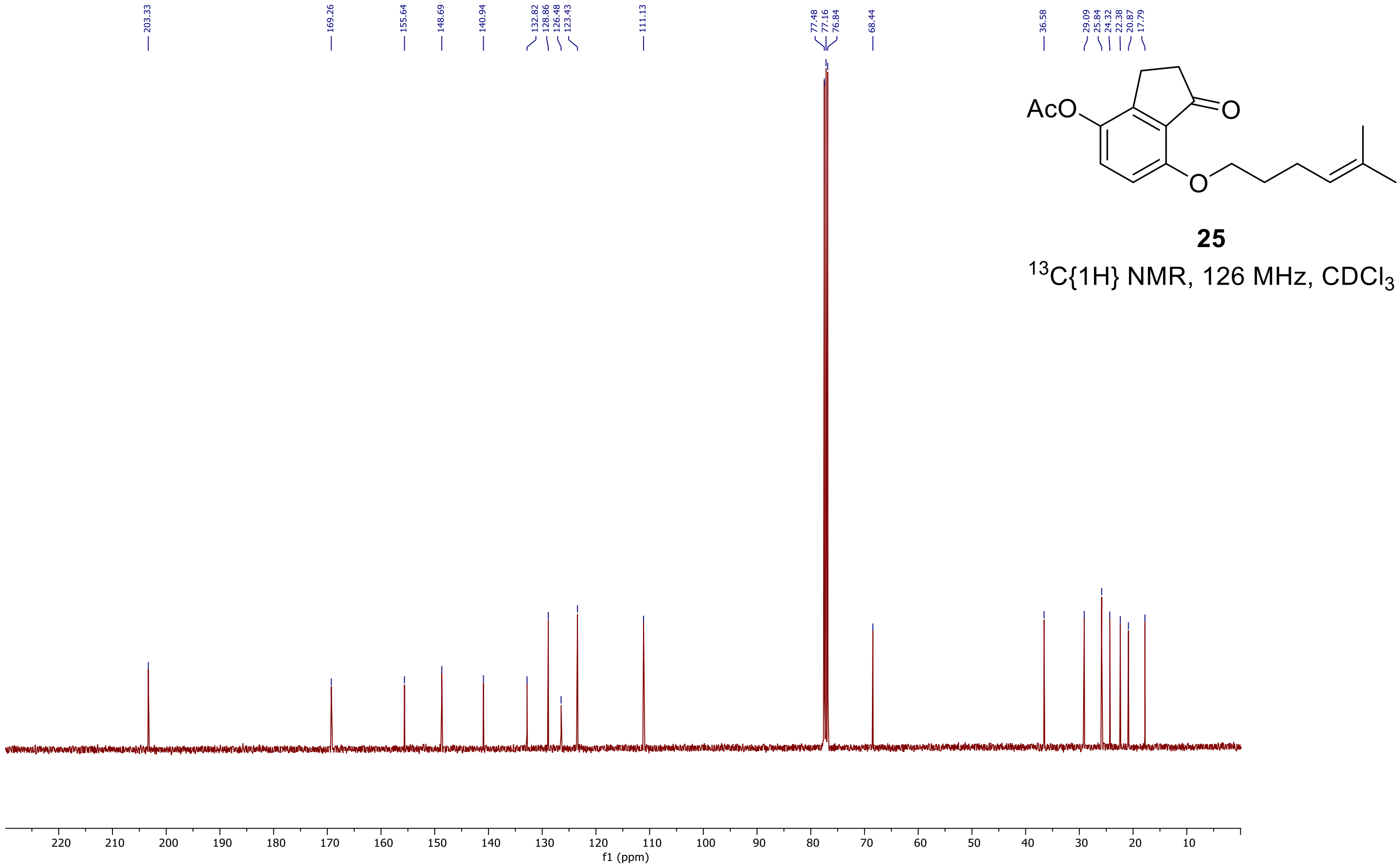

25

$^{13}C\{1H\}$ NMR, 126 MHz, $CDCl_3$

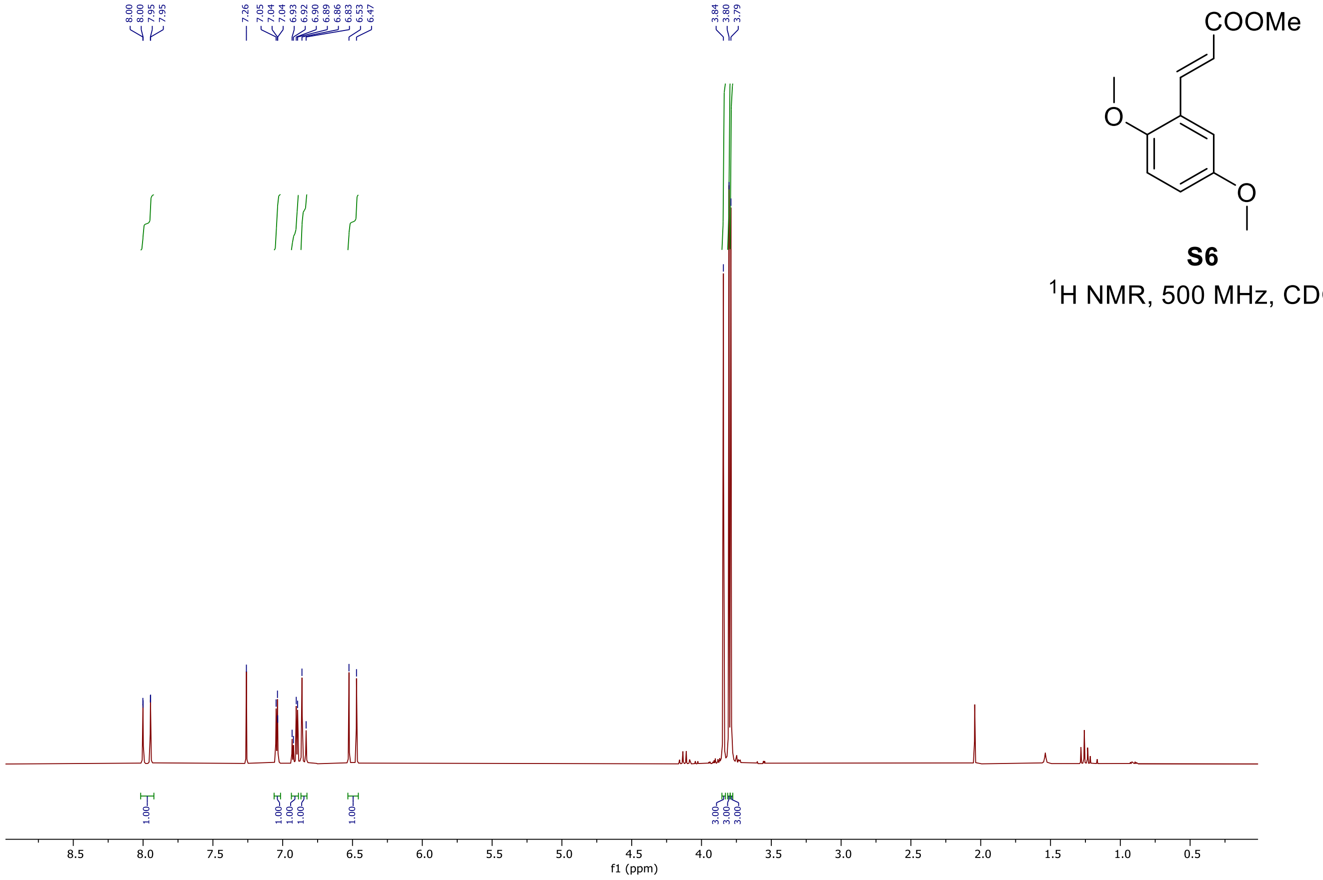

S6

^{1}H NMR, 500 MHz, $CDCl_3$

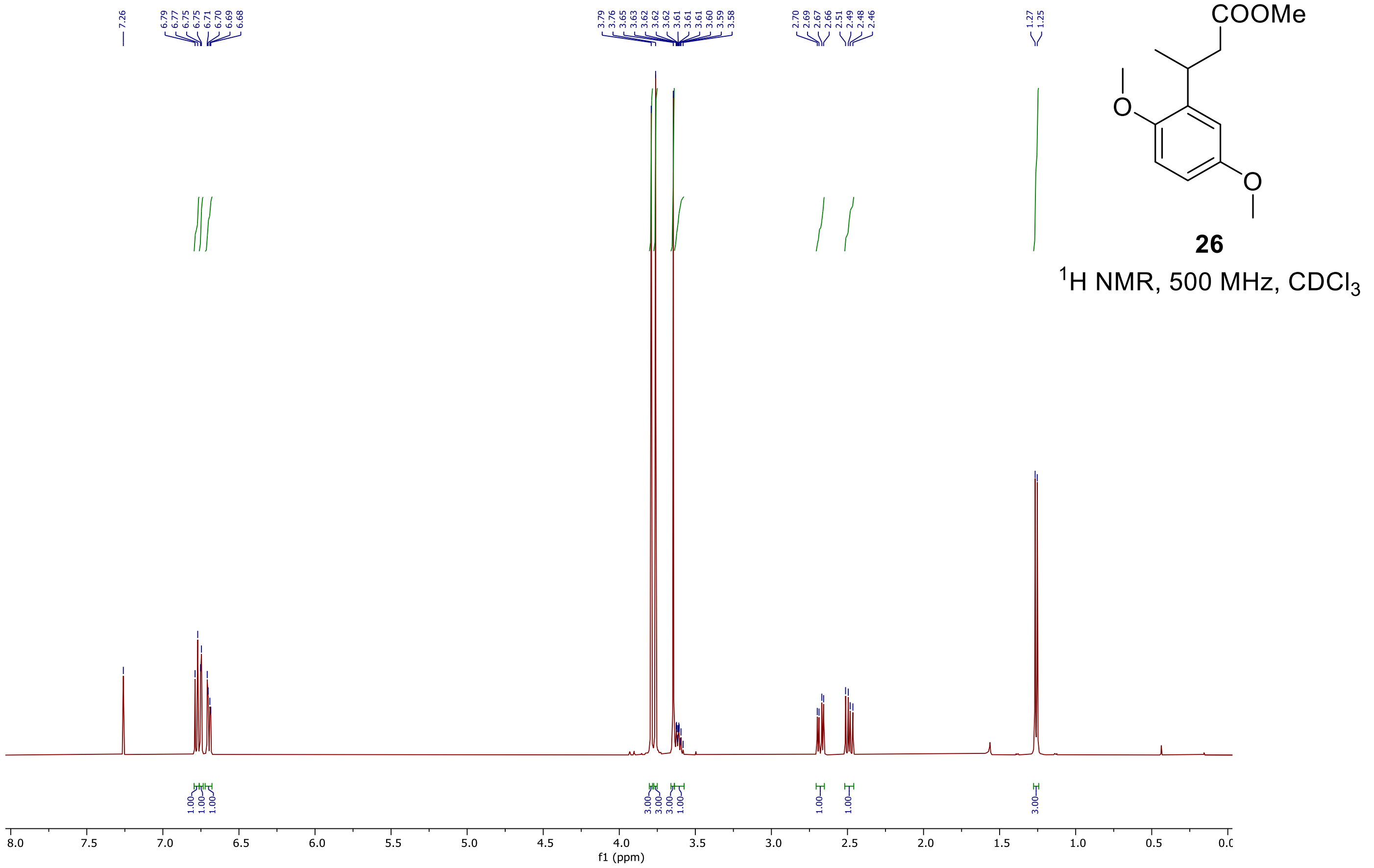

26

^{1}H NMR, 500 MHz, $CDCl_3$

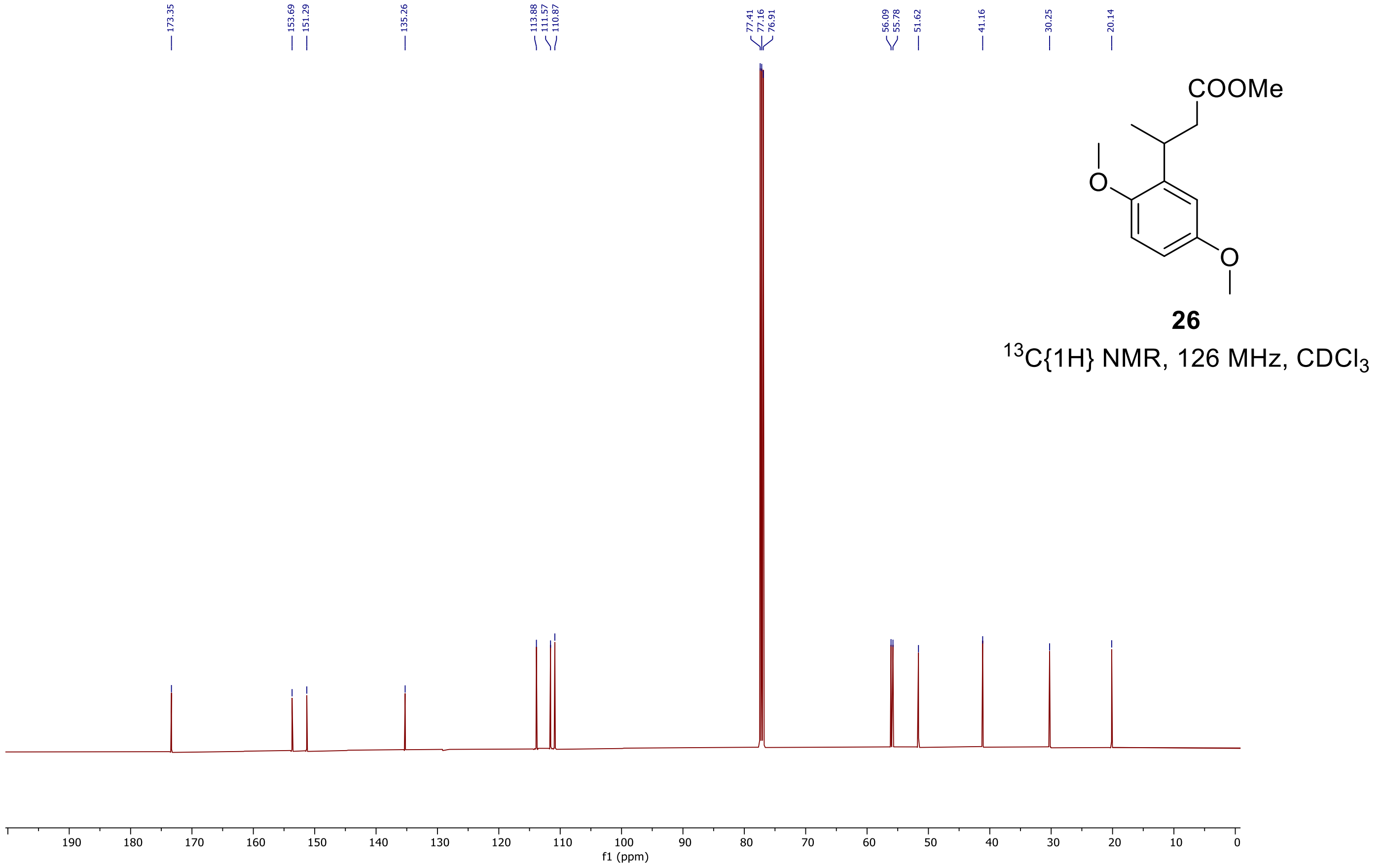

26

^{13}C{1H} NMR, 126 MHz, $CDCl_3$

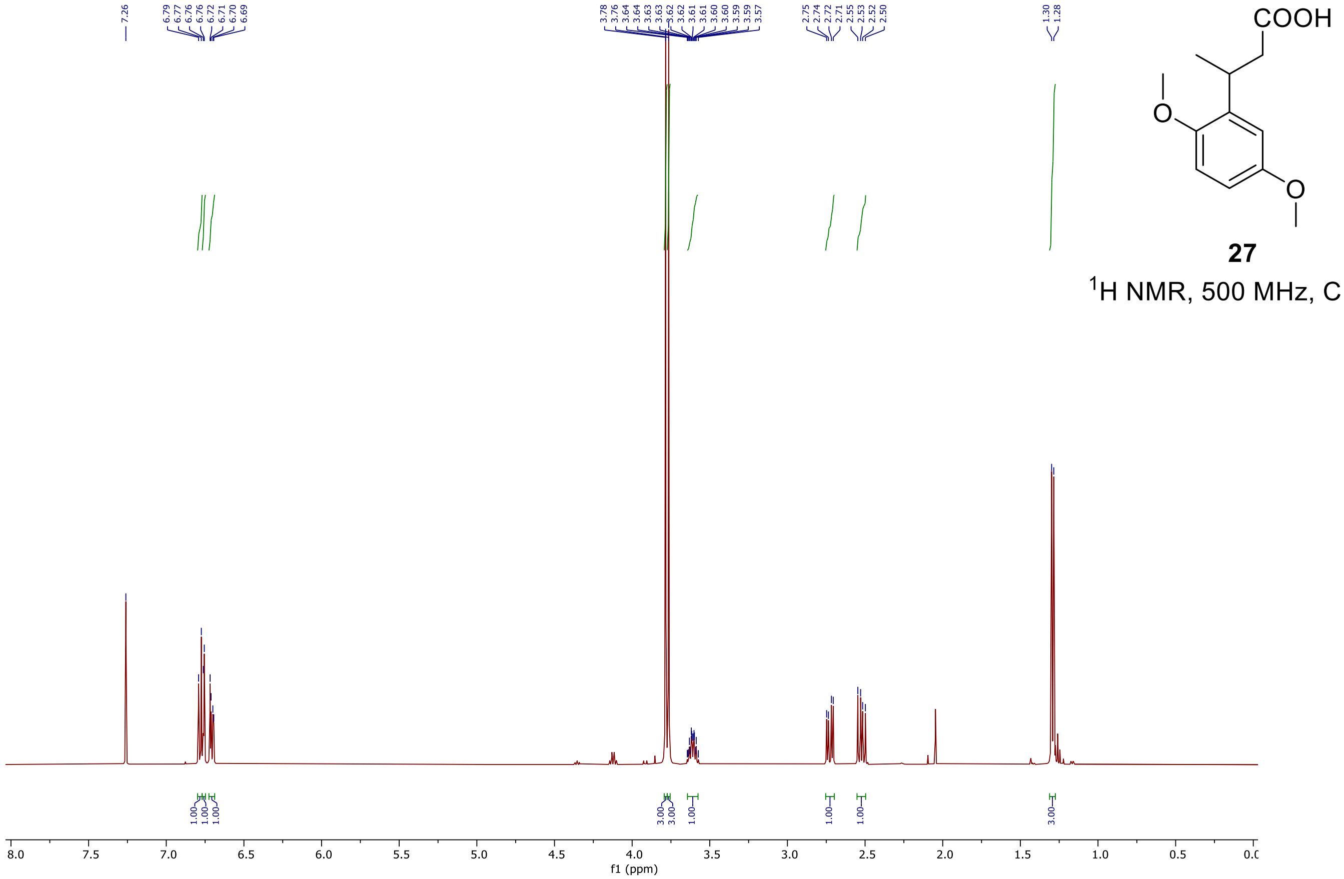

27

^{1}H NMR, 500 MHz, $CDCl_3$

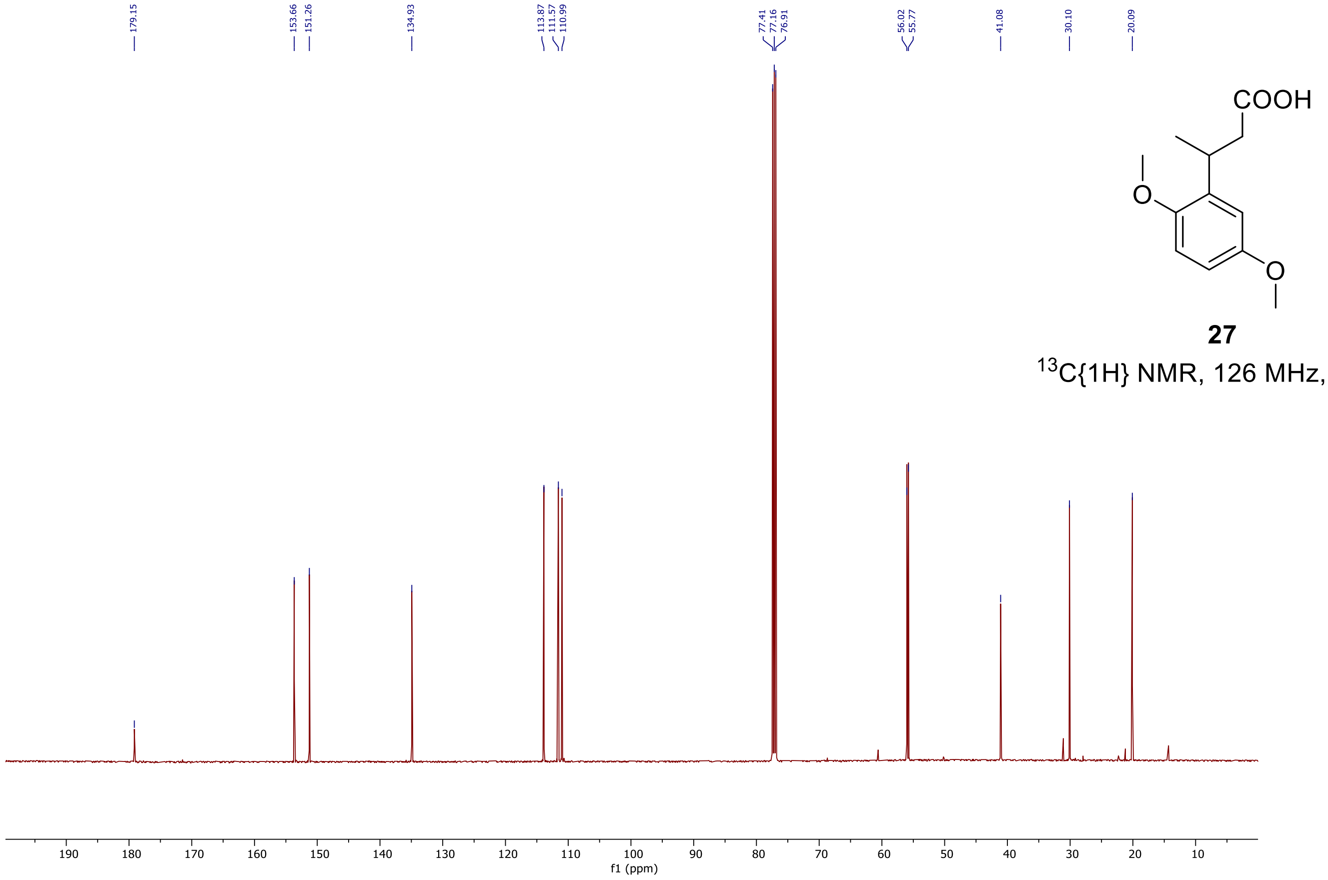

27

$^{13}C\{1H\}$ NMR, 126 MHz, $CDCl_3$

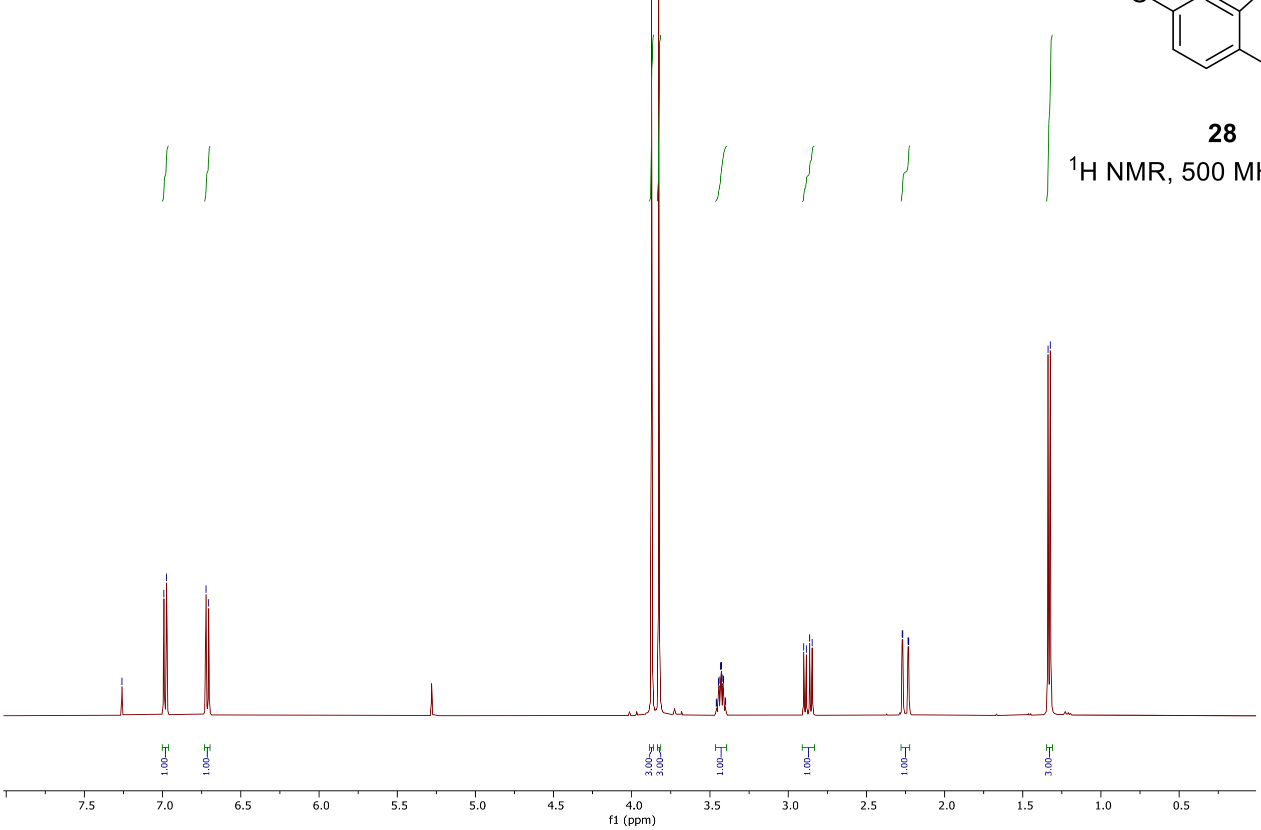

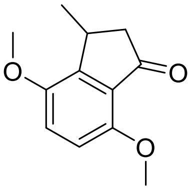

28

^{1}H NMR, 500 MHz, $CDCl_3$

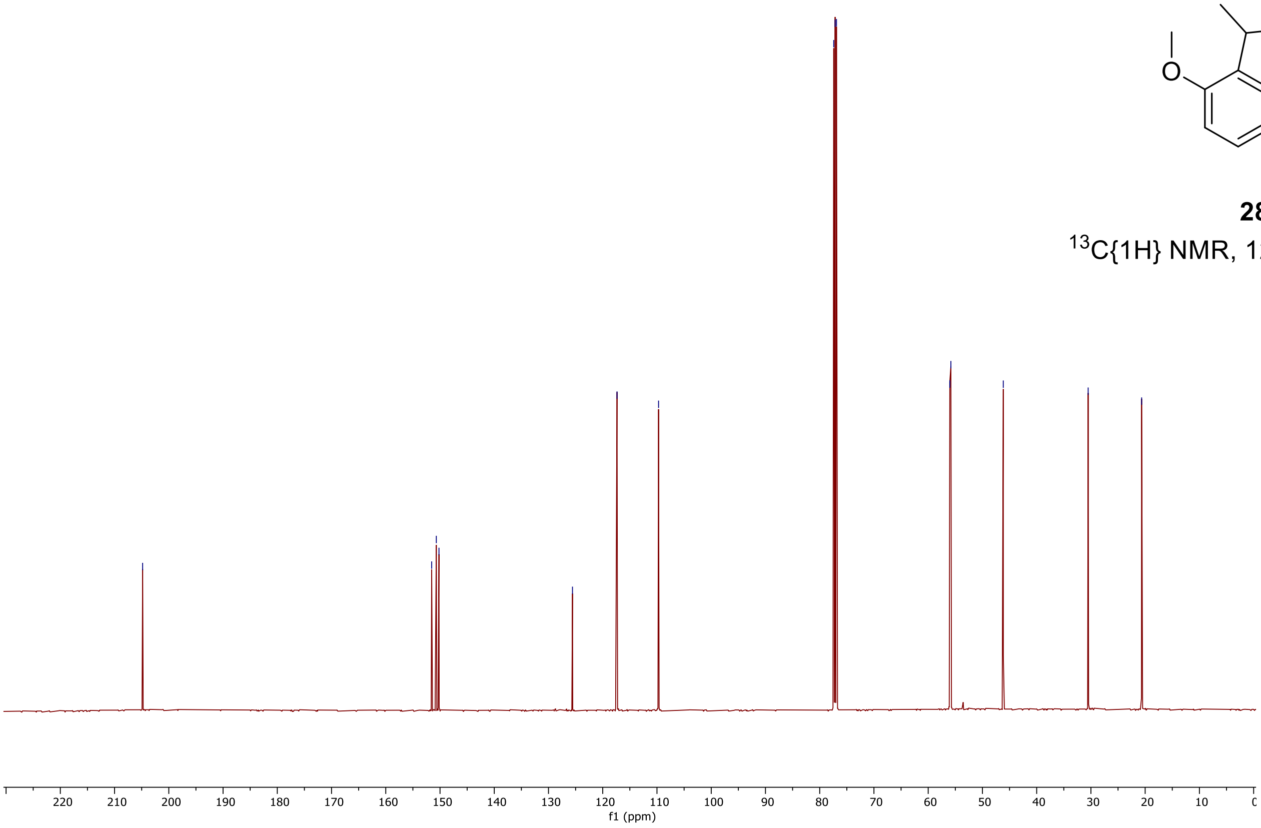

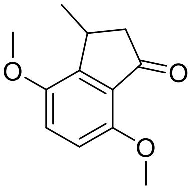

28

^{13}C{1H} NMR, 126 MHz, $CDCl_3$

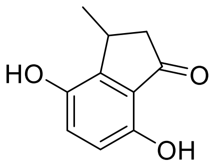

29

^{1}H NMR, 500 MHz, $CDCl_3$

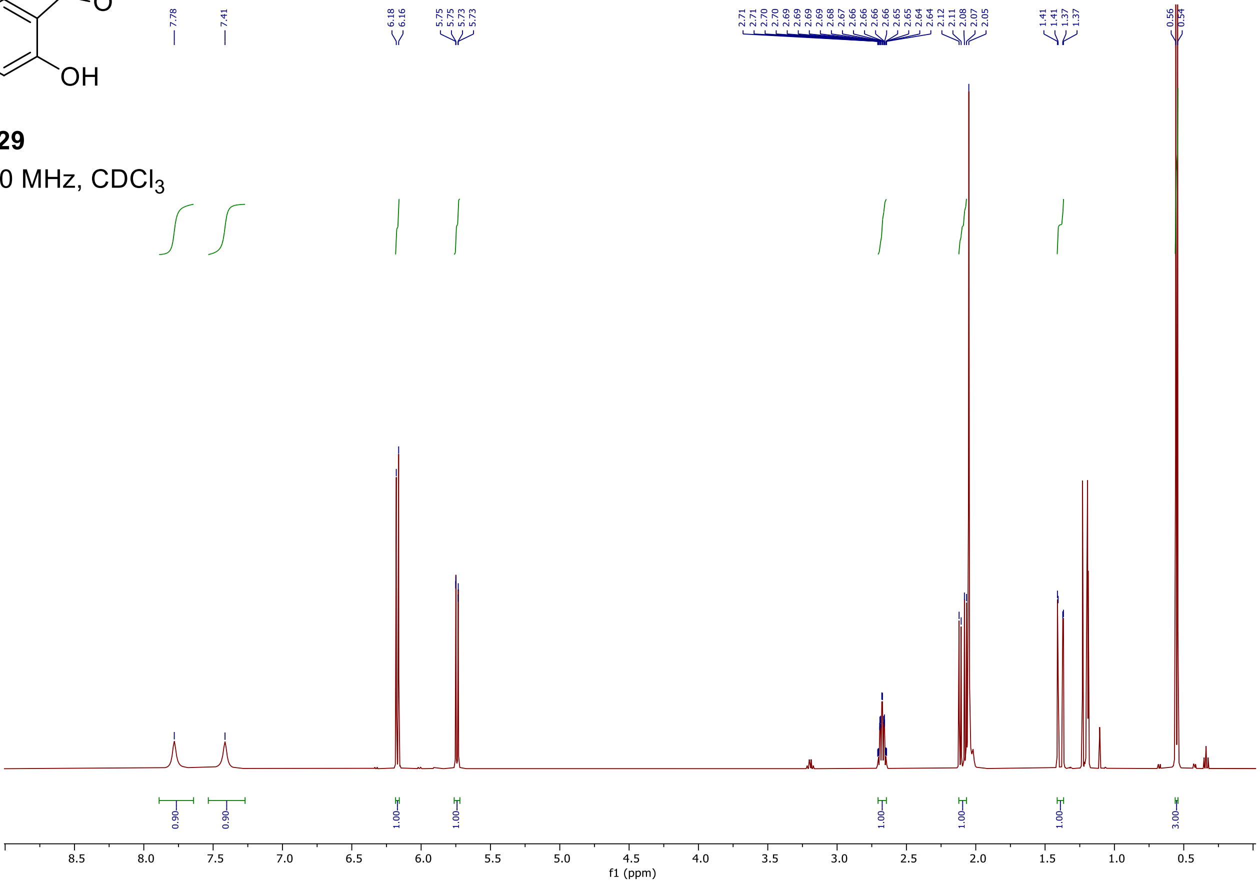

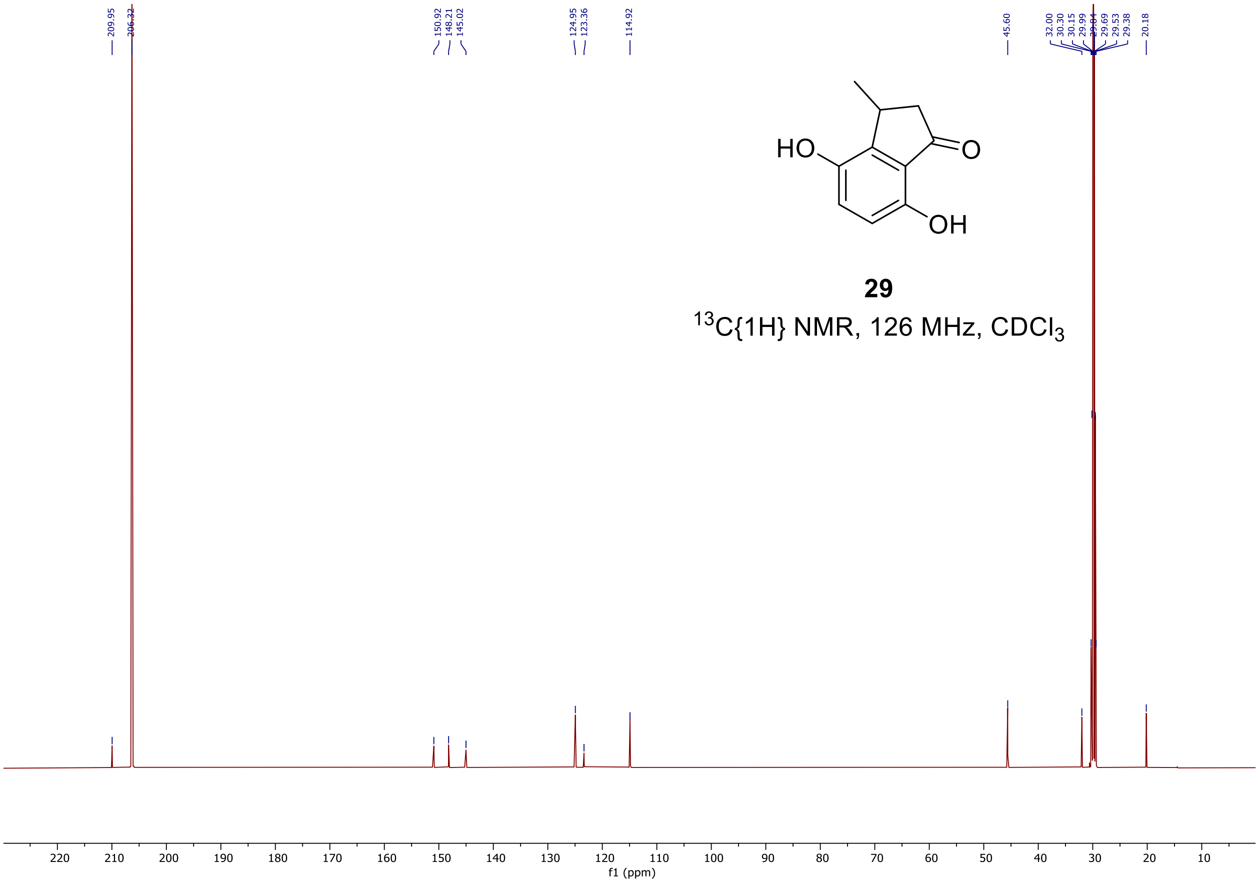

29

$^{13}C\{1H\}$ NMR, 126 MHz, $CDCl_3$

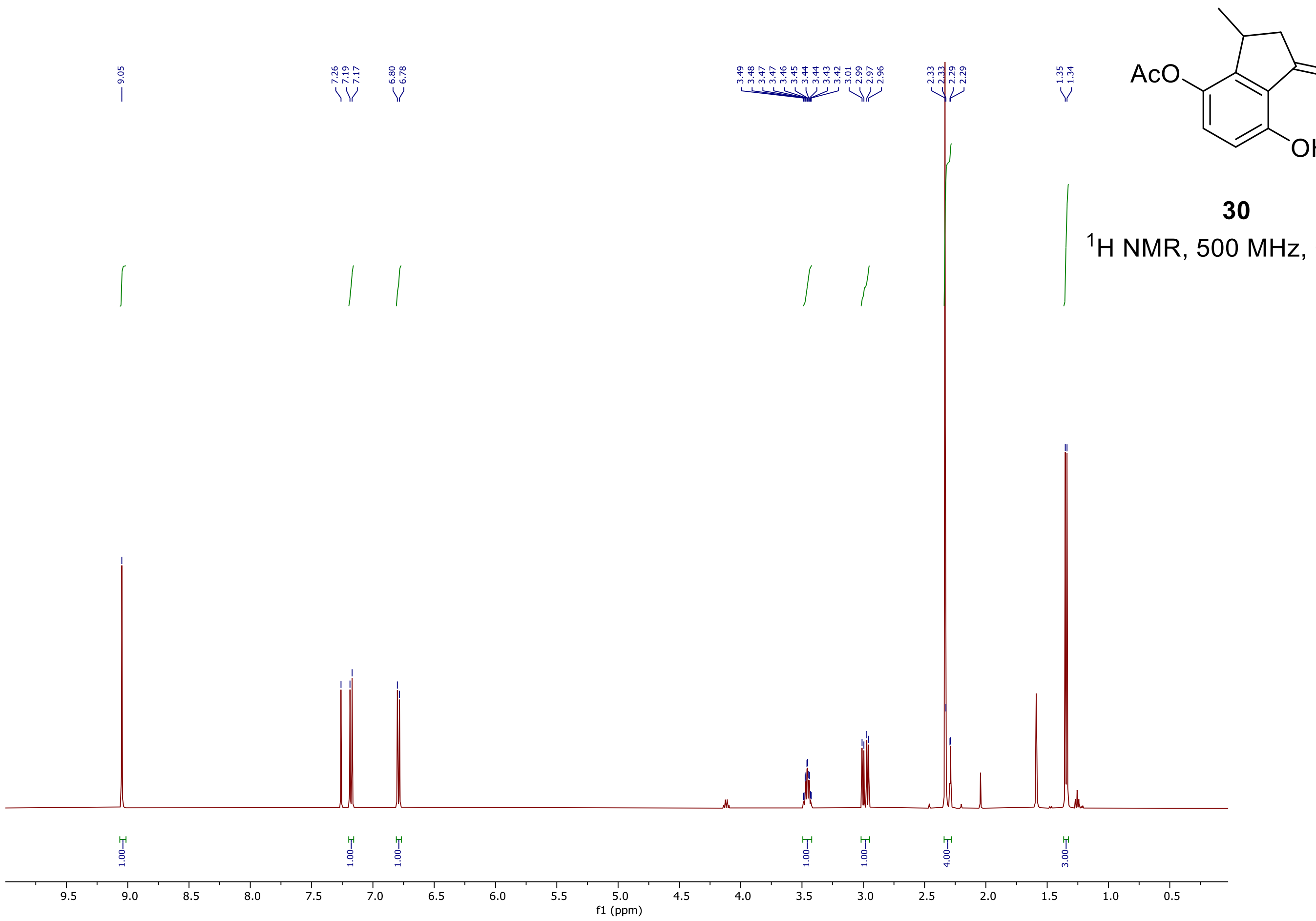

30

^{1}H NMR, 500 MHz, $CDCl_3$

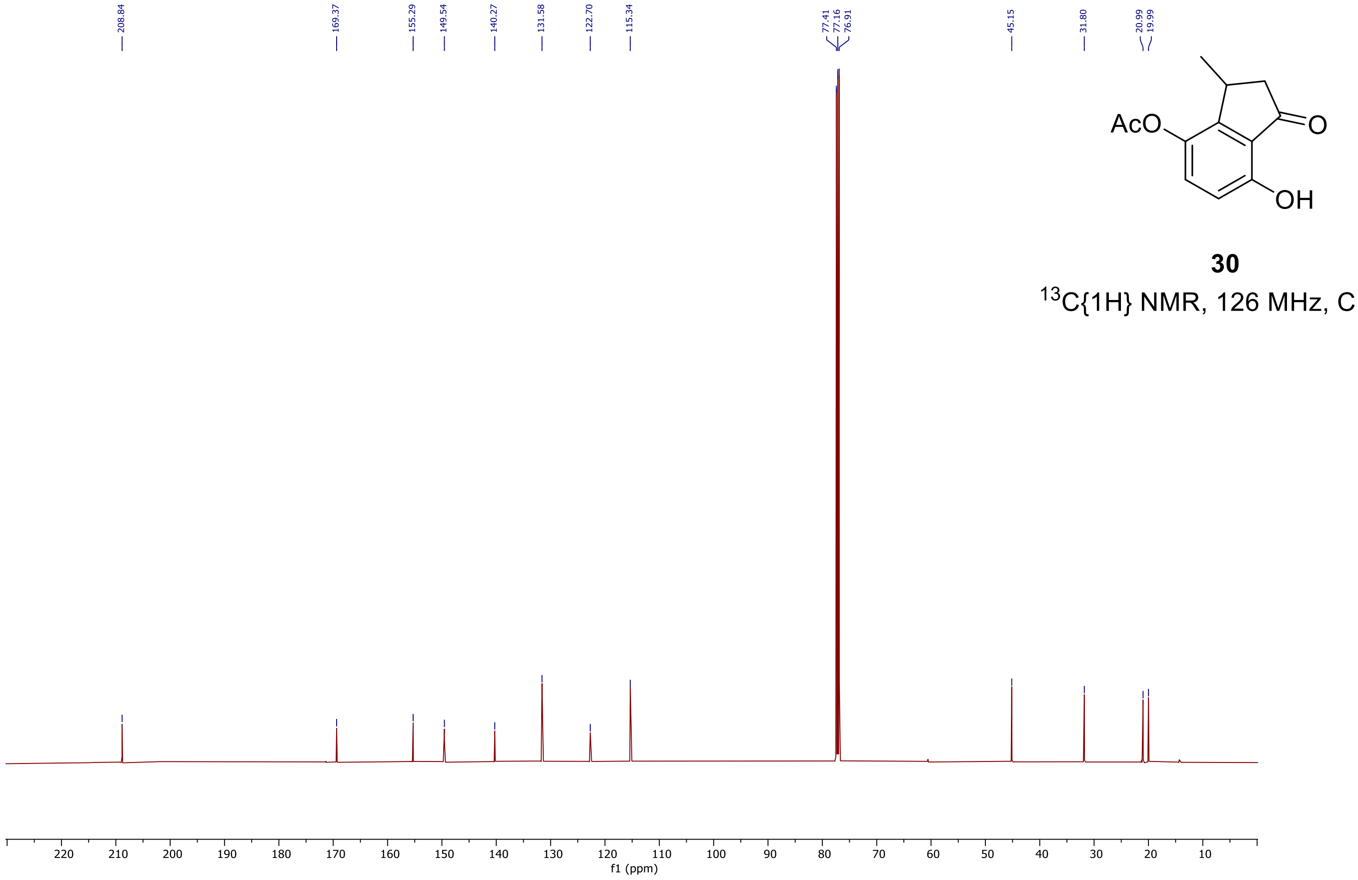

30

$^{13}C\{1H\}$ NMR, 126 MHz, $CDCl_3$

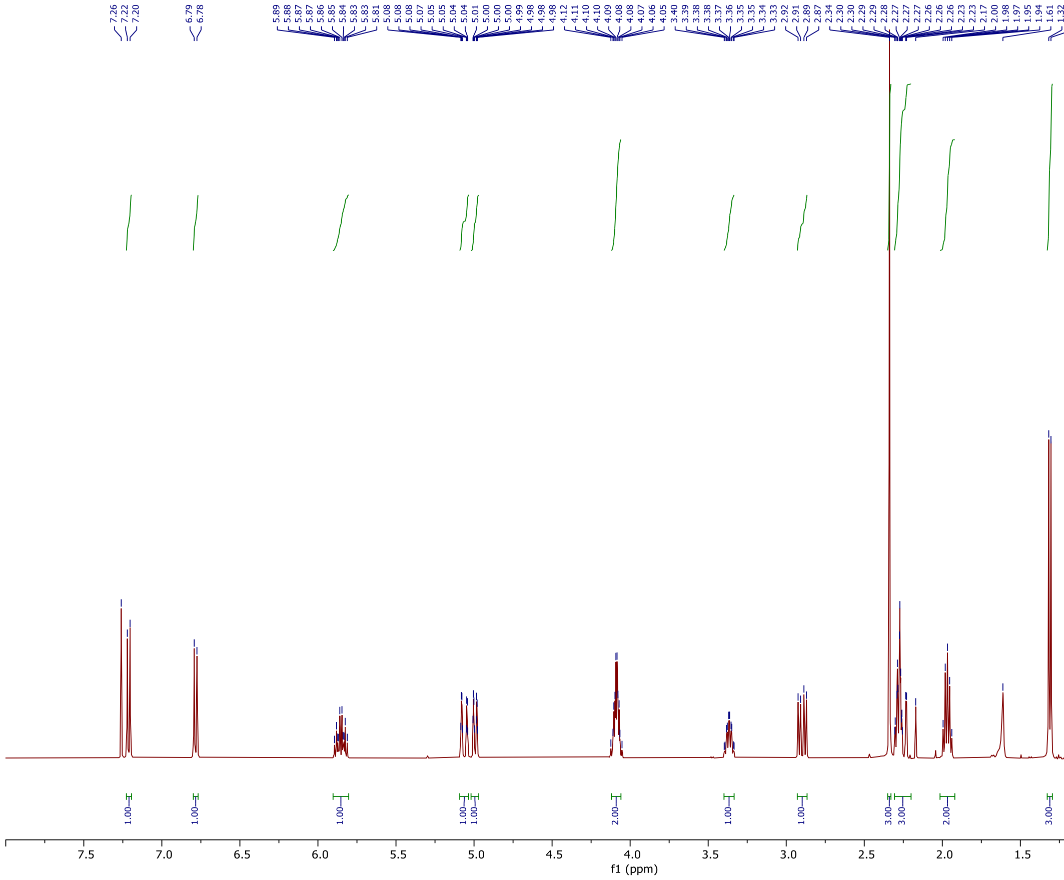

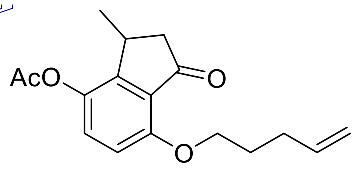

31

^{1}H NMR, 500 MHz, $CDCl_3$

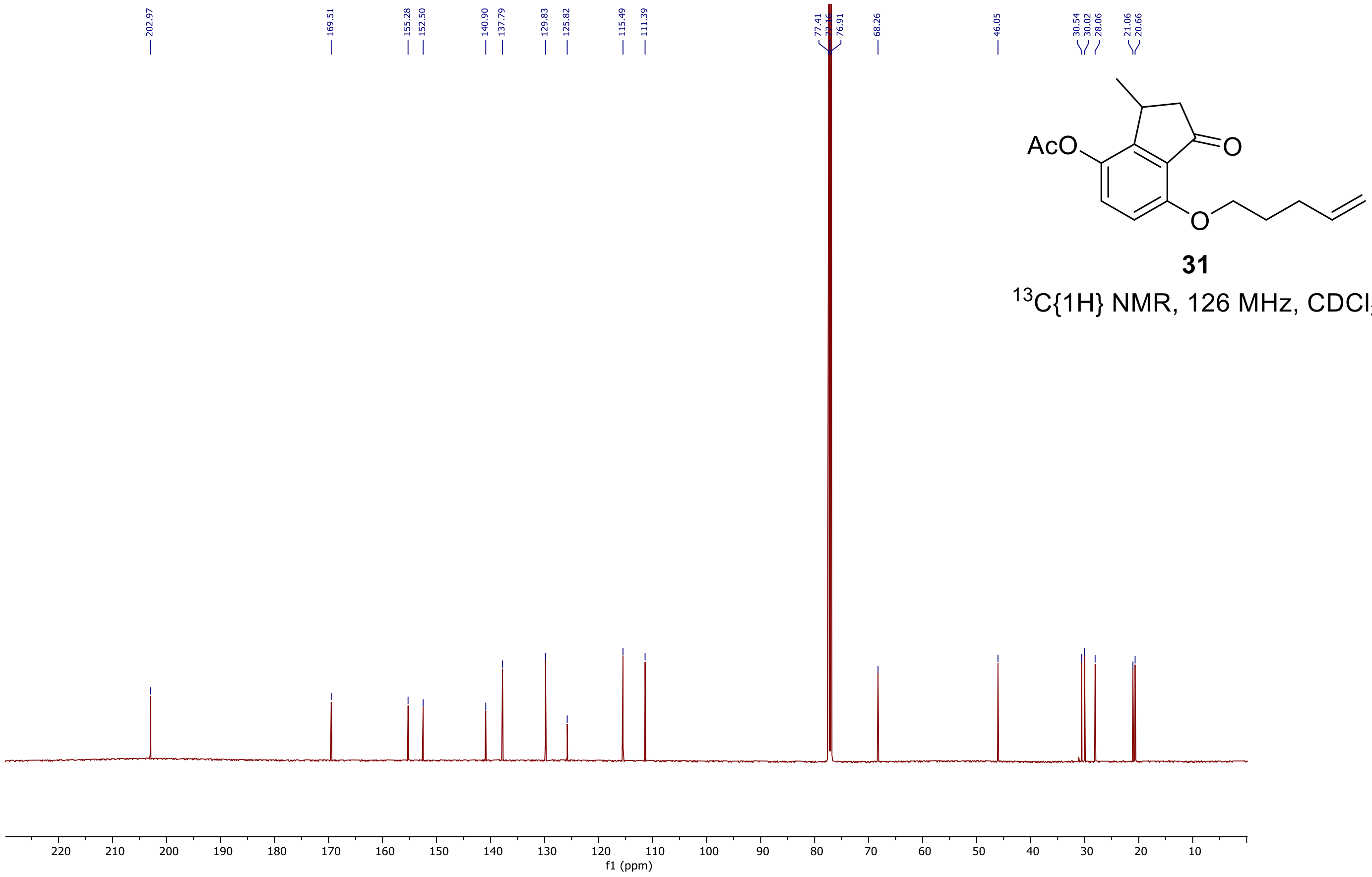

31

^{13}C{1H} NMR, 126 MHz, $CDCl_3$

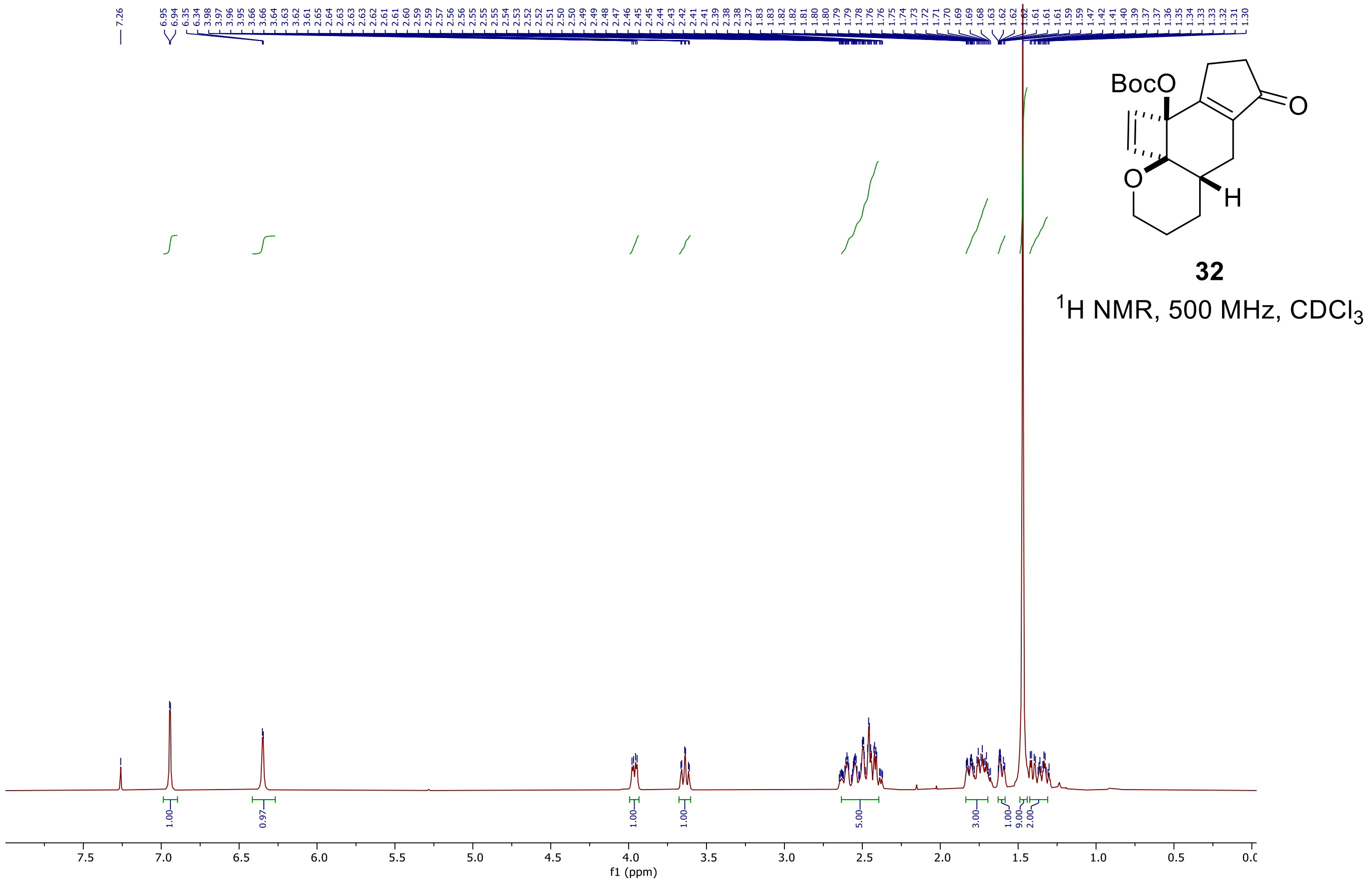

32
^{1}H NMR, 500 MHz, $CDCl_3$

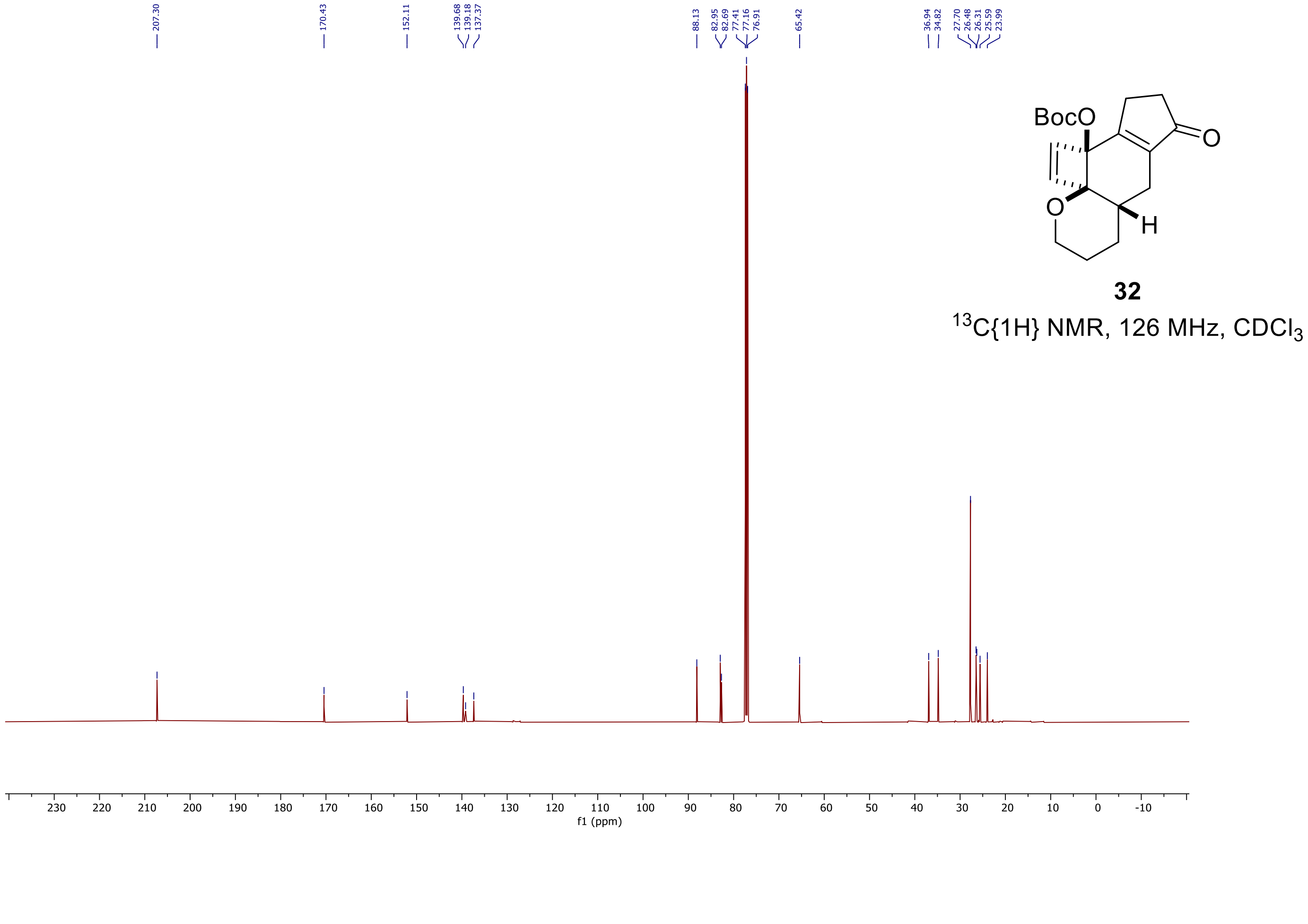

32
^{13}C{1H} NMR, 126 MHz, $CDCl_3$

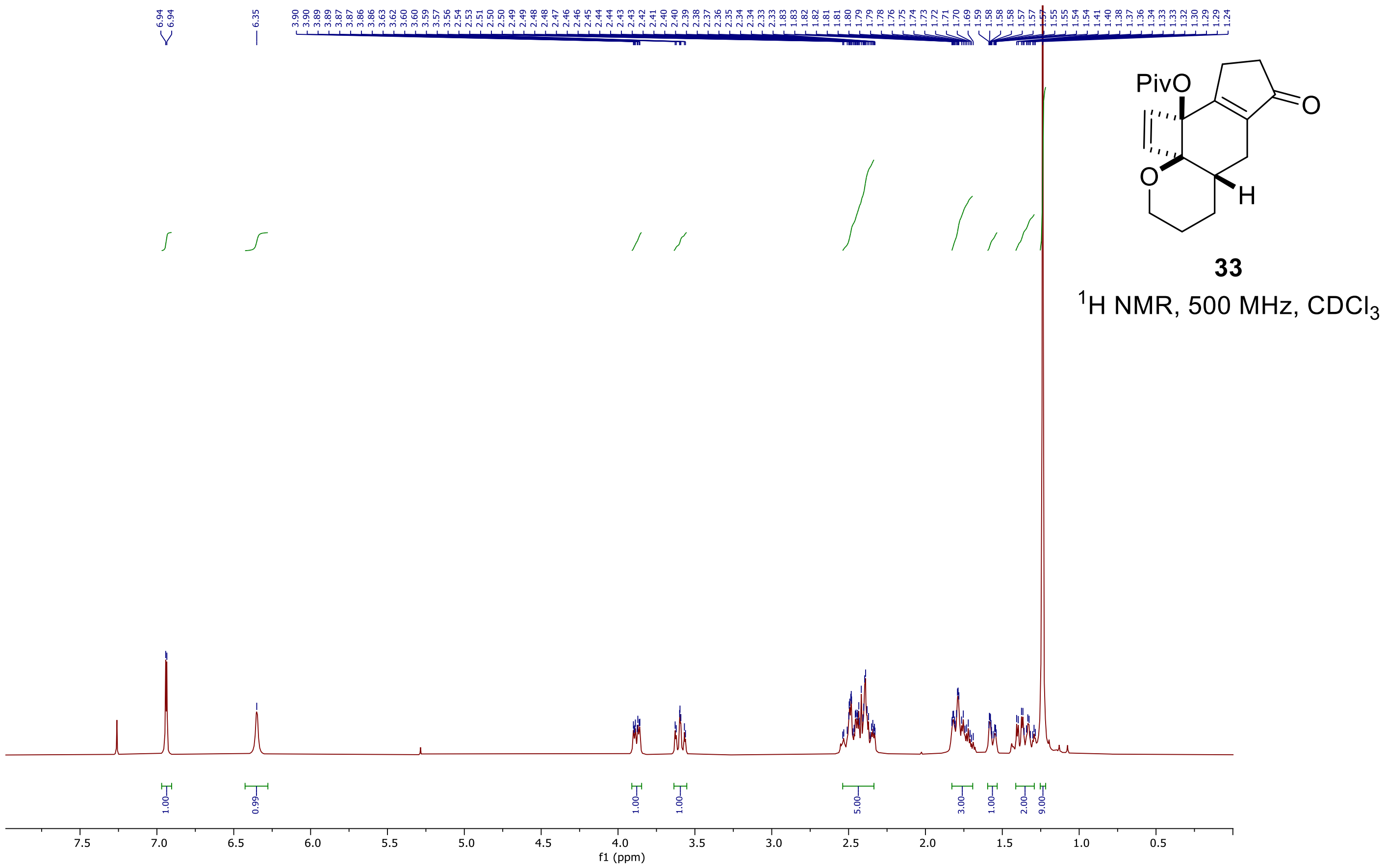

33

^{1}H NMR, 500 MHz, $CDCl_3$

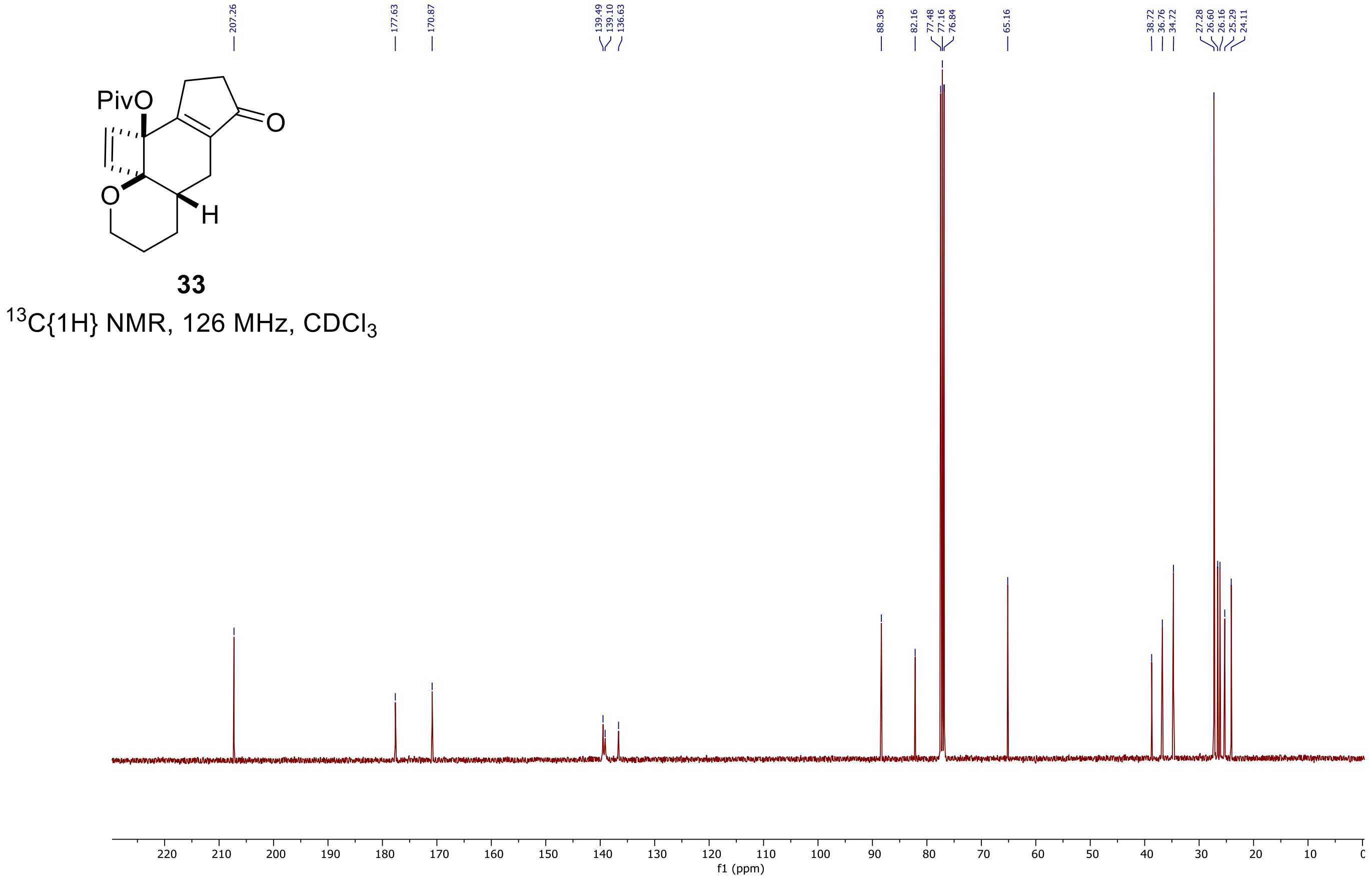

33

^{13}C{1H} NMR, 126 MHz, $CDCl_3$

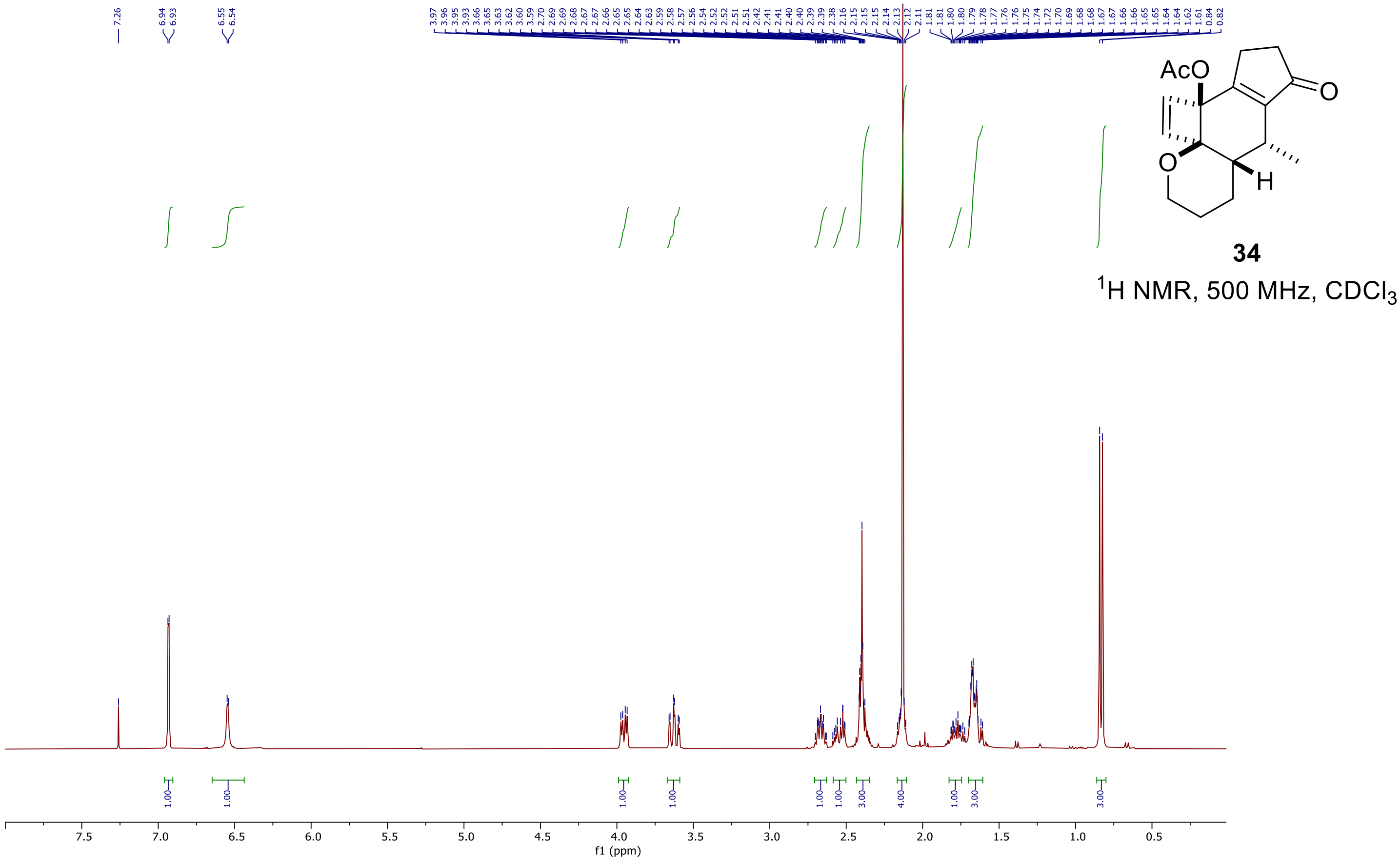

34

^{1}H NMR, 500 MHz, $CDCl_3$

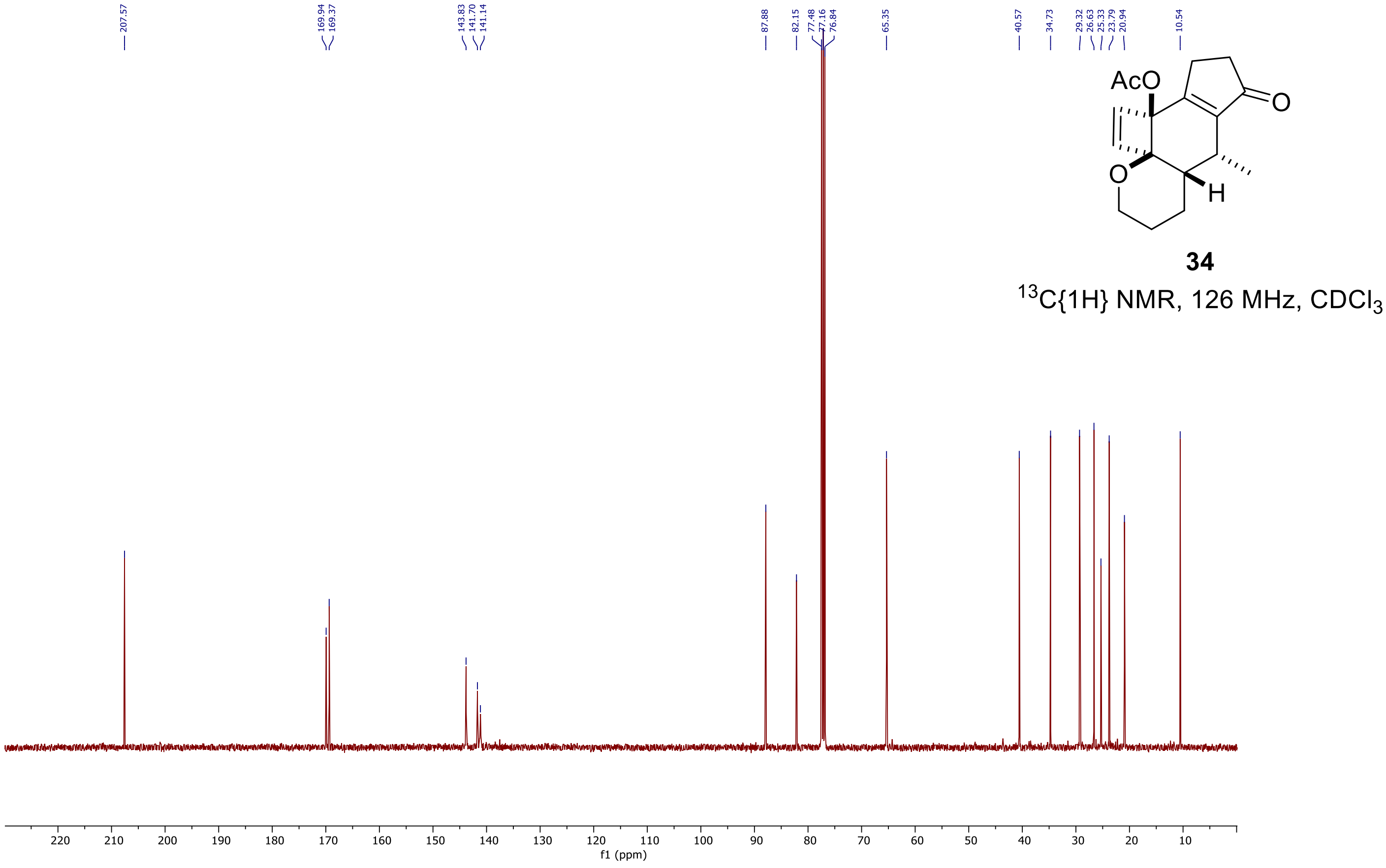

34

^{13}C{1H} NMR, 126 MHz, $CDCl_3$

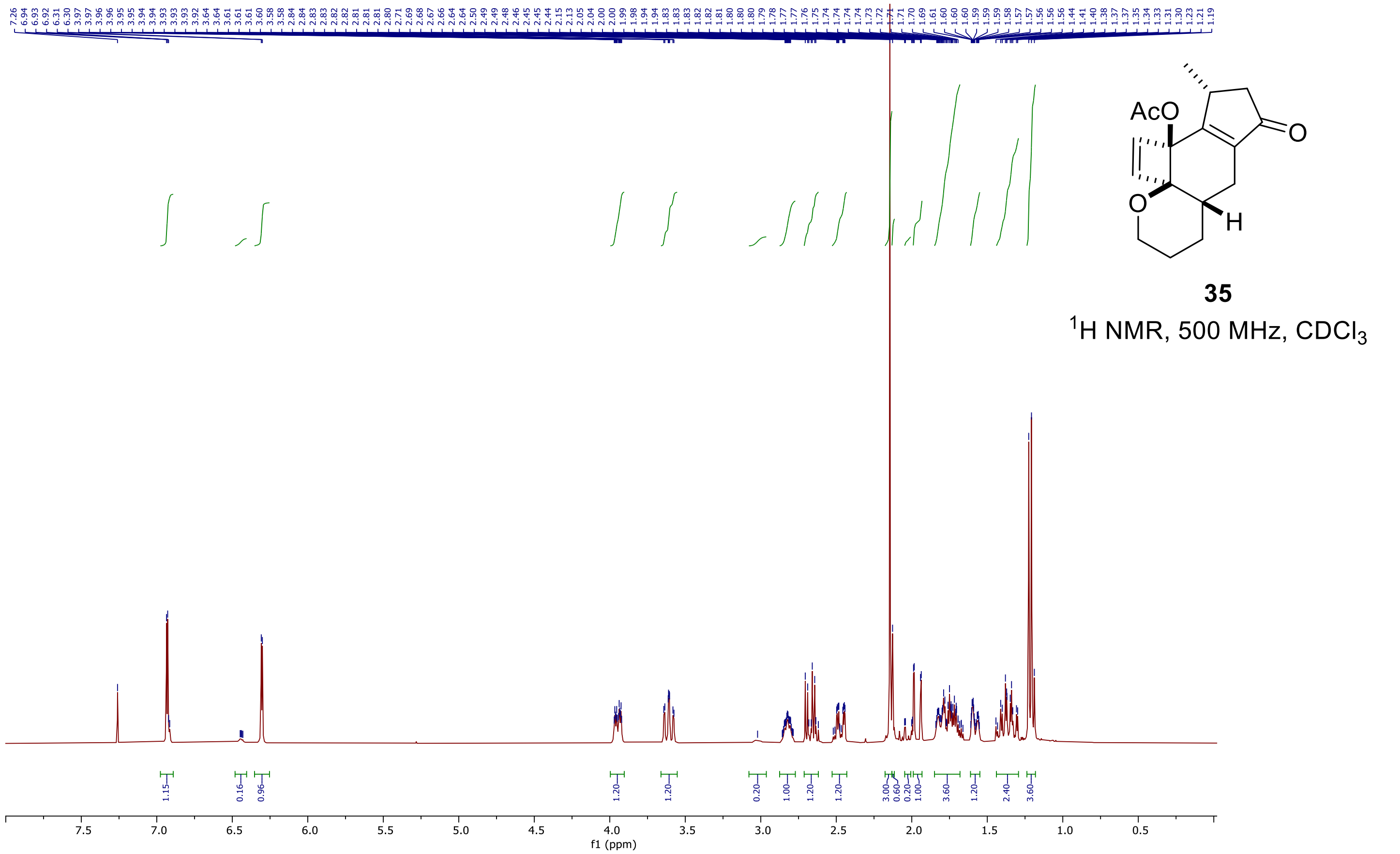

35

^{1}H NMR, 500 MHz, $CDCl_3$

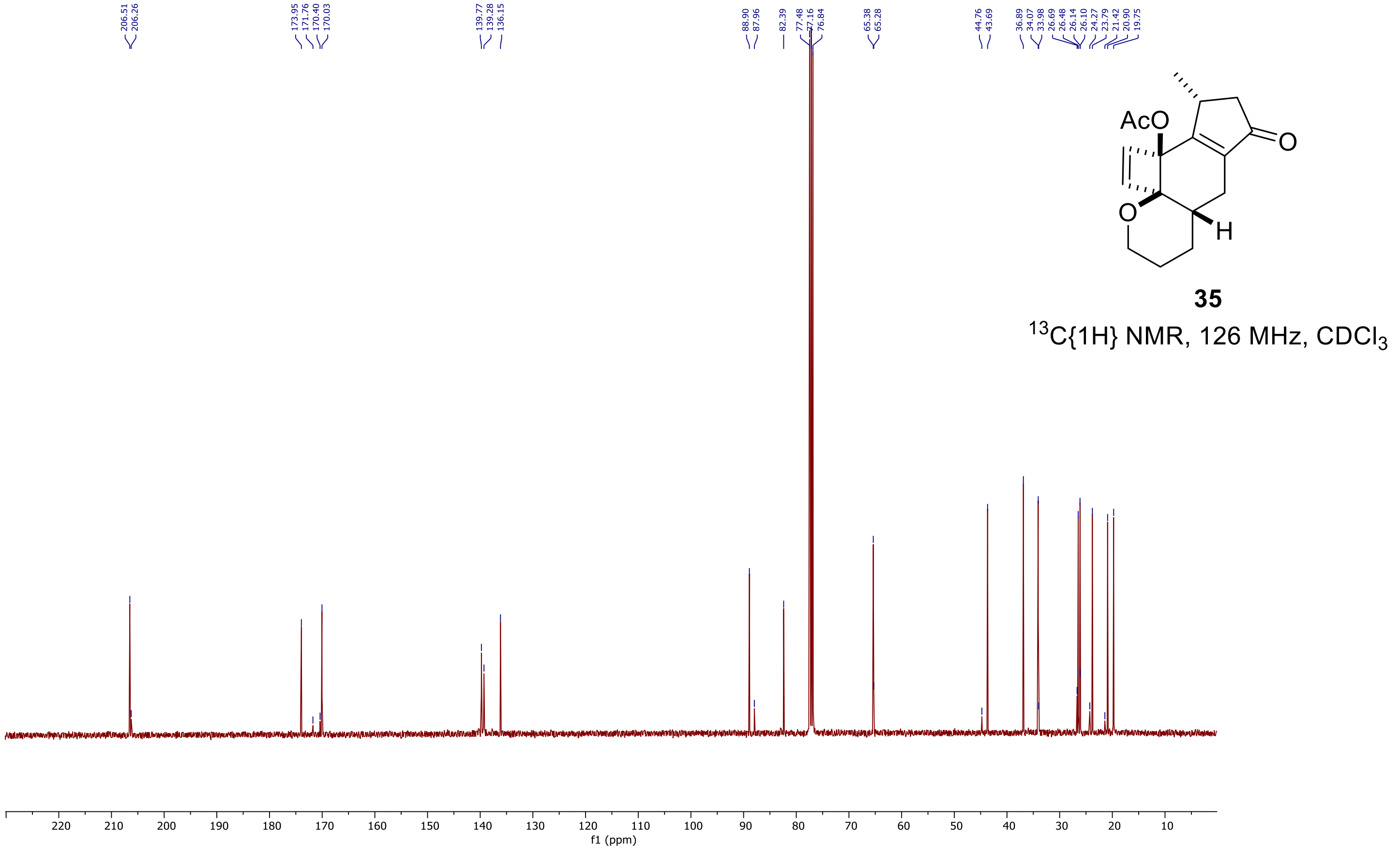

35

^{13}C{1H} NMR, 126 MHz, $CDCl_3$

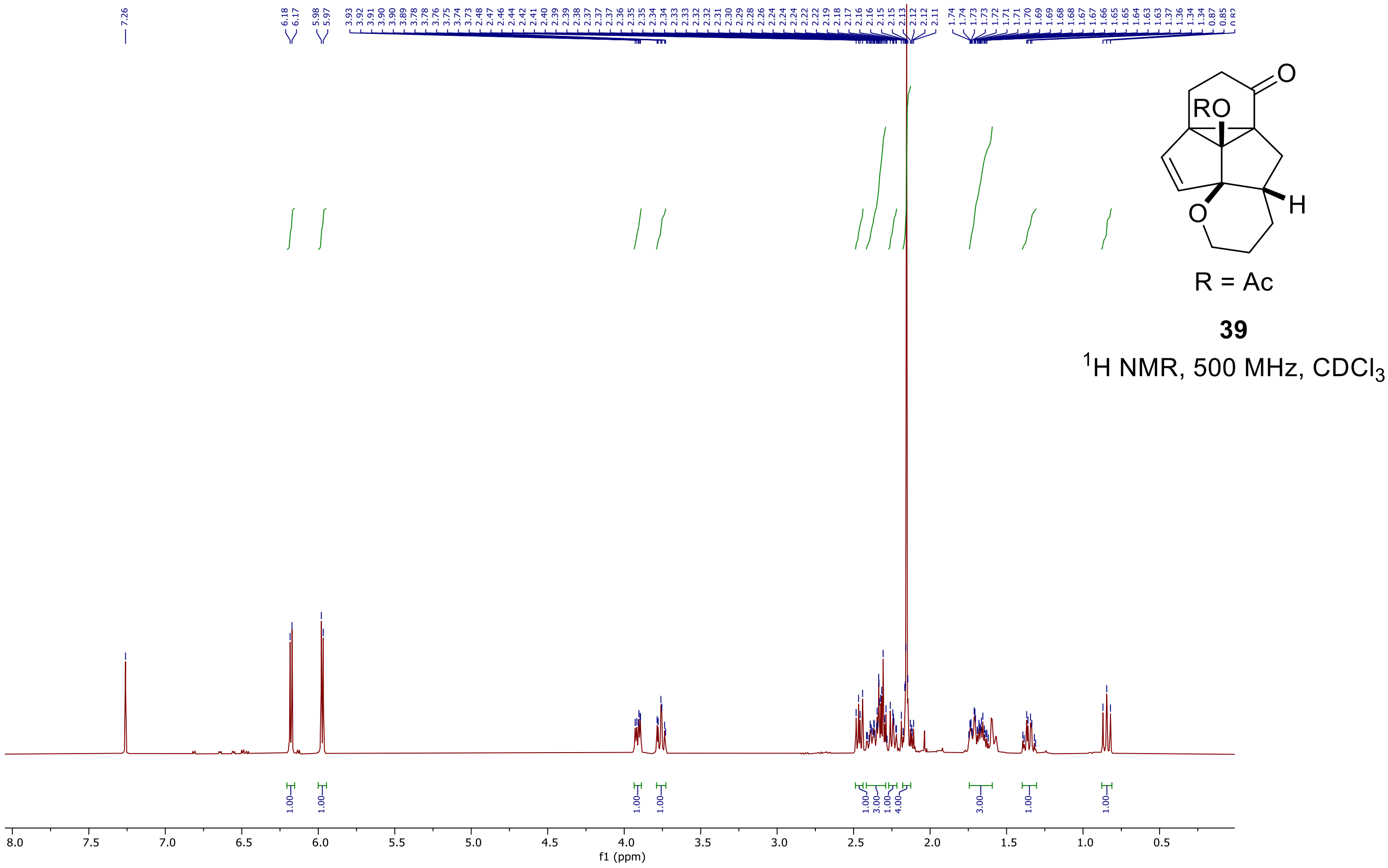

39

^{1}H NMR, 500 MHz, $CDCl_3$

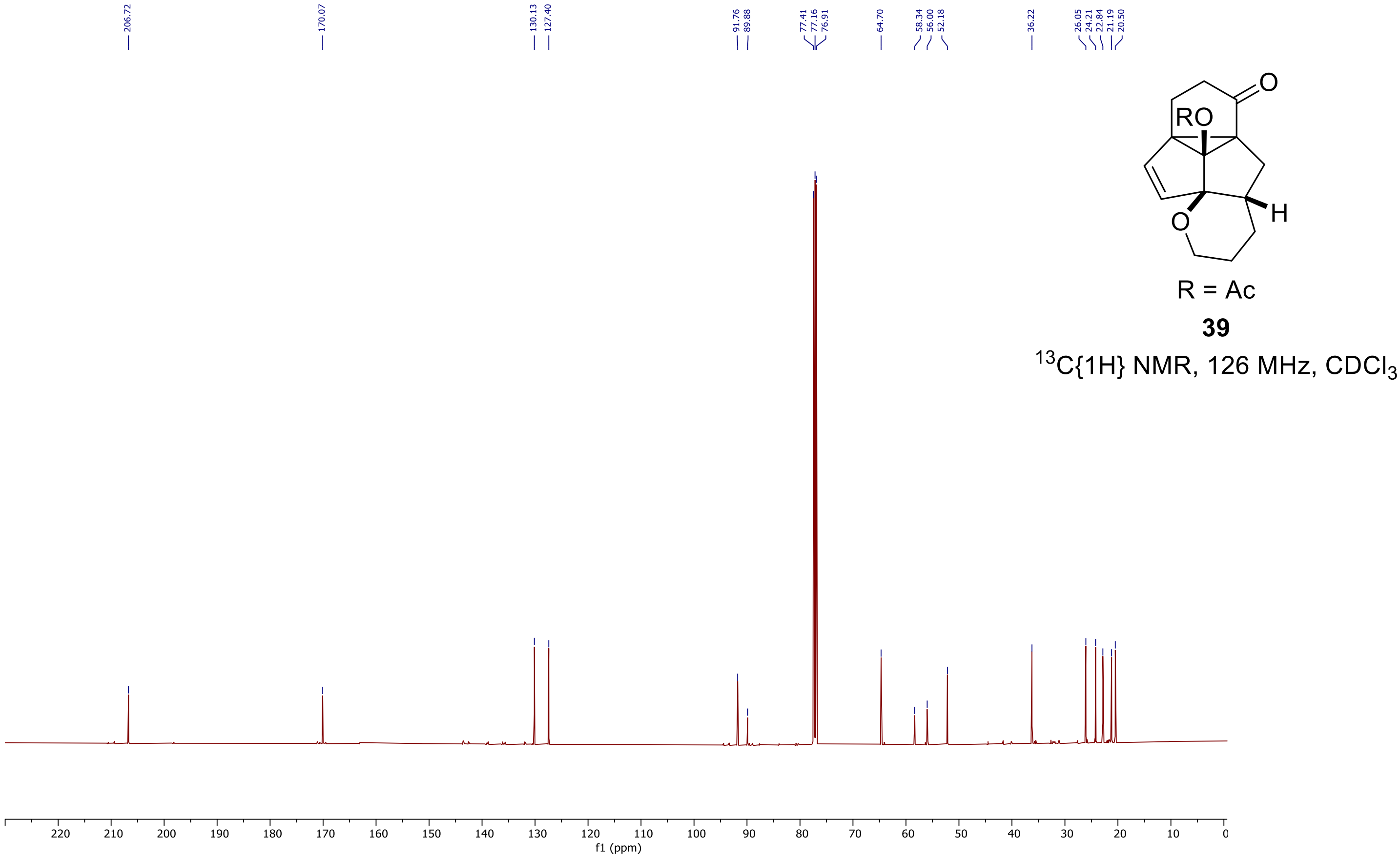

39

^{13}C{1H} NMR, 126 MHz, $CDCl_3$

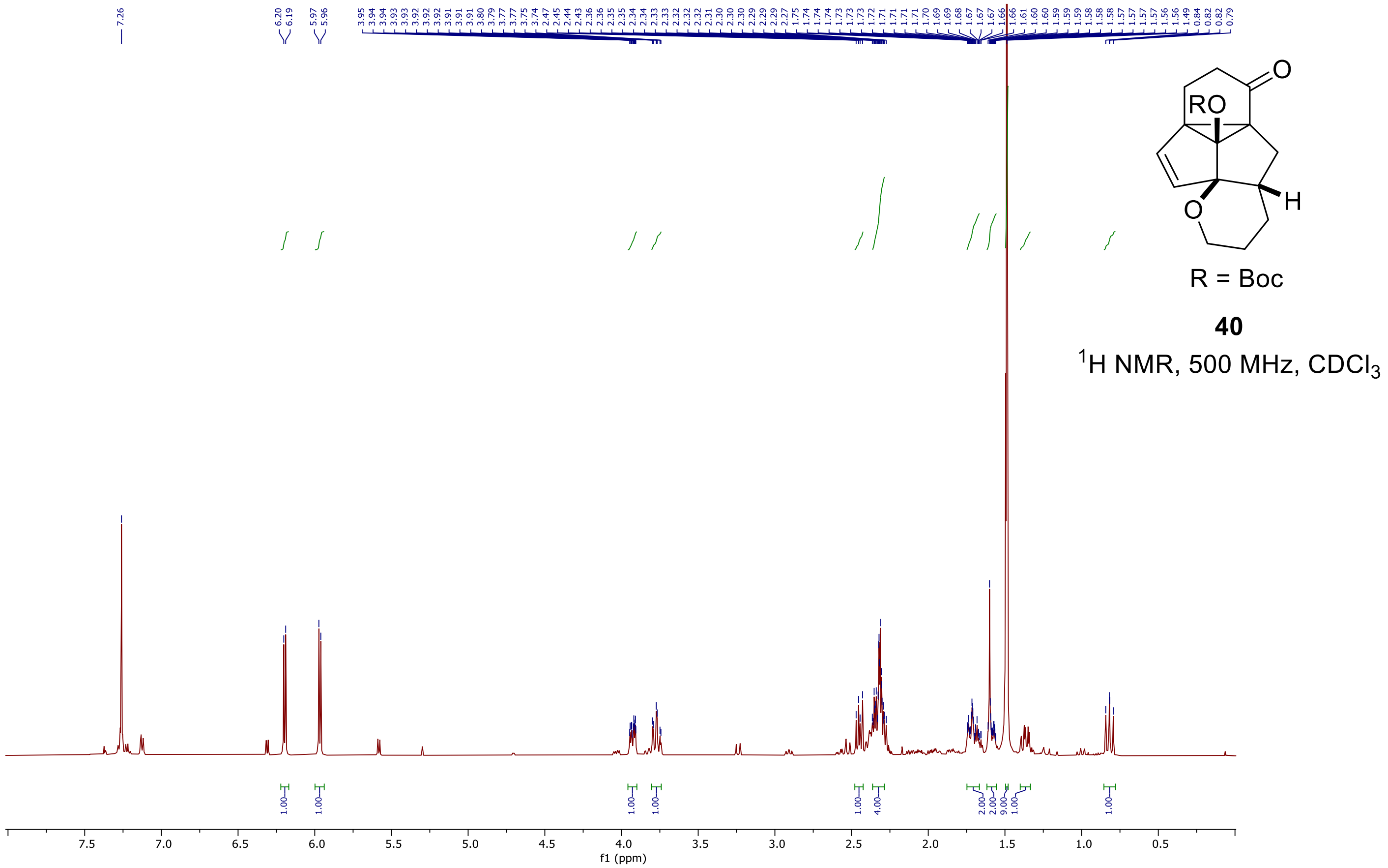
7.26
6.20 6.19
5.97 5.96
3.95 3.94 3.93 3.93 3.92 3.92 3.92 3.91 3.91 3.91 3.80 3.79 3.77 3.77 3.75 3.74 2.47 2.45 2.44 2.43 2.36 2.36 2.35 2.35 2.34 2.34 2.33 2.33 2.32 2.32 2.32 2.31 2.30 2.30 2.30 2.29 2.29 2.27 1.75 1.74 1.74 1.73 1.73 1.73 1.72 1.71 1.71 1.71 1.70 1.69 1.69 1.68 1.67 1.67 1.67 1.66 1.66 1.61 1.60 1.60 1.59 1.59 1.59 1.58 1.58 1.58 1.57 1.57 1.57 1.57 1.56 1.56 1.49 0.84 0.82 0.79
1.00 1.00 1.00 1.00 1.00 4.00 2.00 2.00 9.00 1.00 1.00
RO
O
H
O
R = Boc
40
1H NMR, 500 MHz, CDCl3
f1 (ppm)

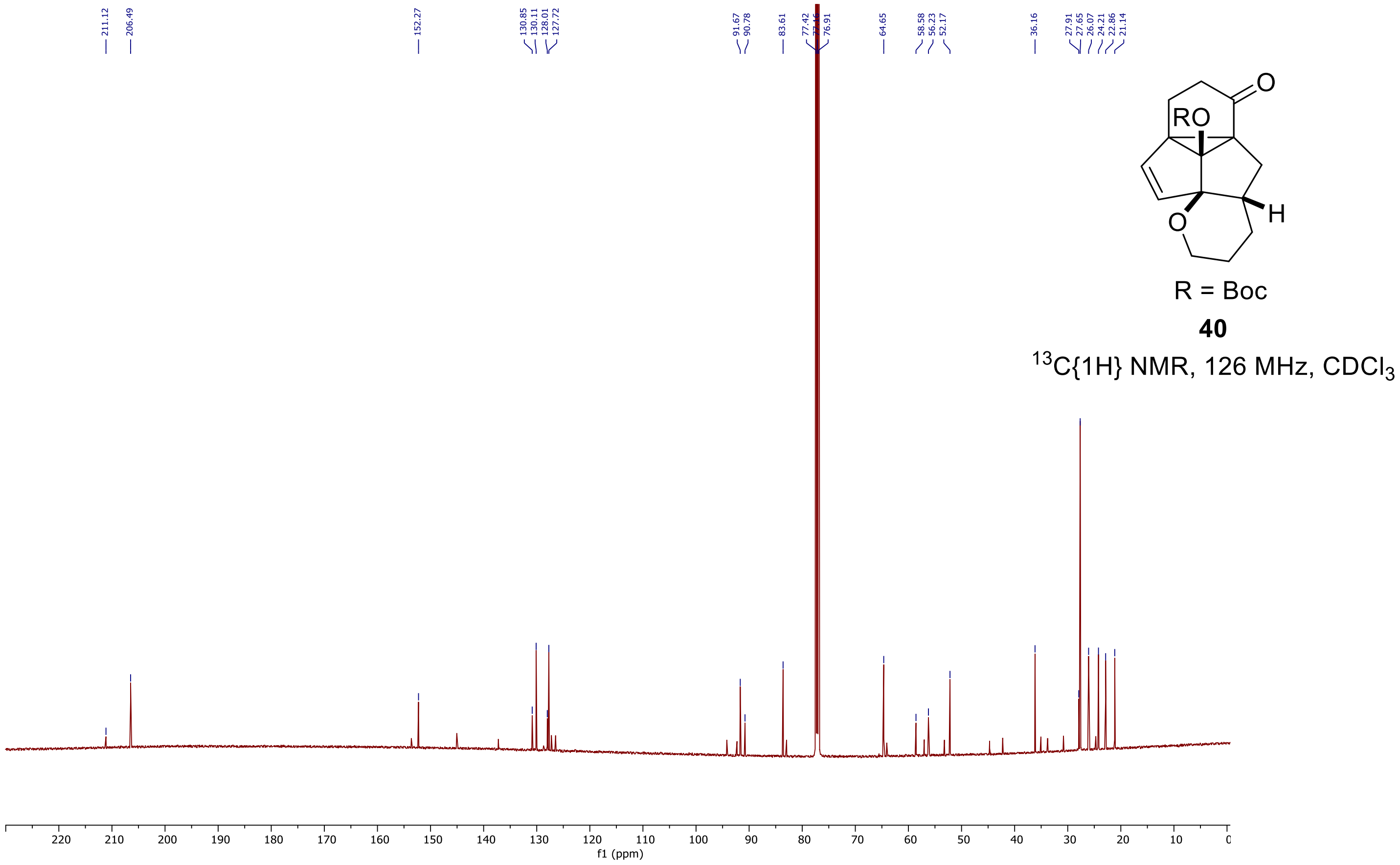
211.12
206.49
152.27
130.85 130.11 128.01 127.72
91.67 90.78
83.61
77.42 76.91
64.65
58.58 56.23 52.17
36.16
27.91 27.65 26.07 24.21 22.86 21.14
RO
O
H
O
R = Boc
40
13C{1H} NMR, 126 MHz, CDCl3
f1 (ppm)

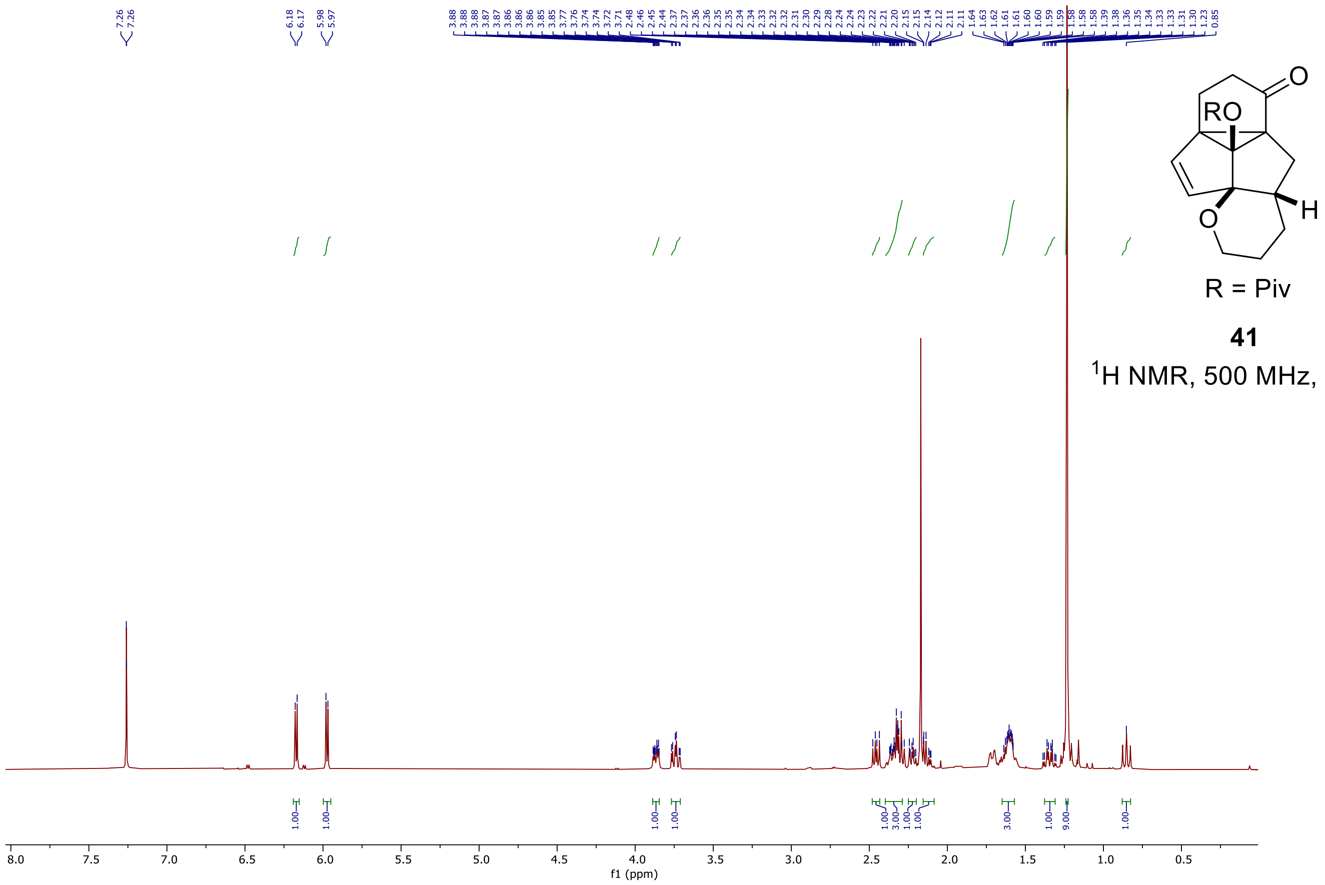

R = Piv

41

^{1}H NMR, 500 MHz, $CDCl_3$

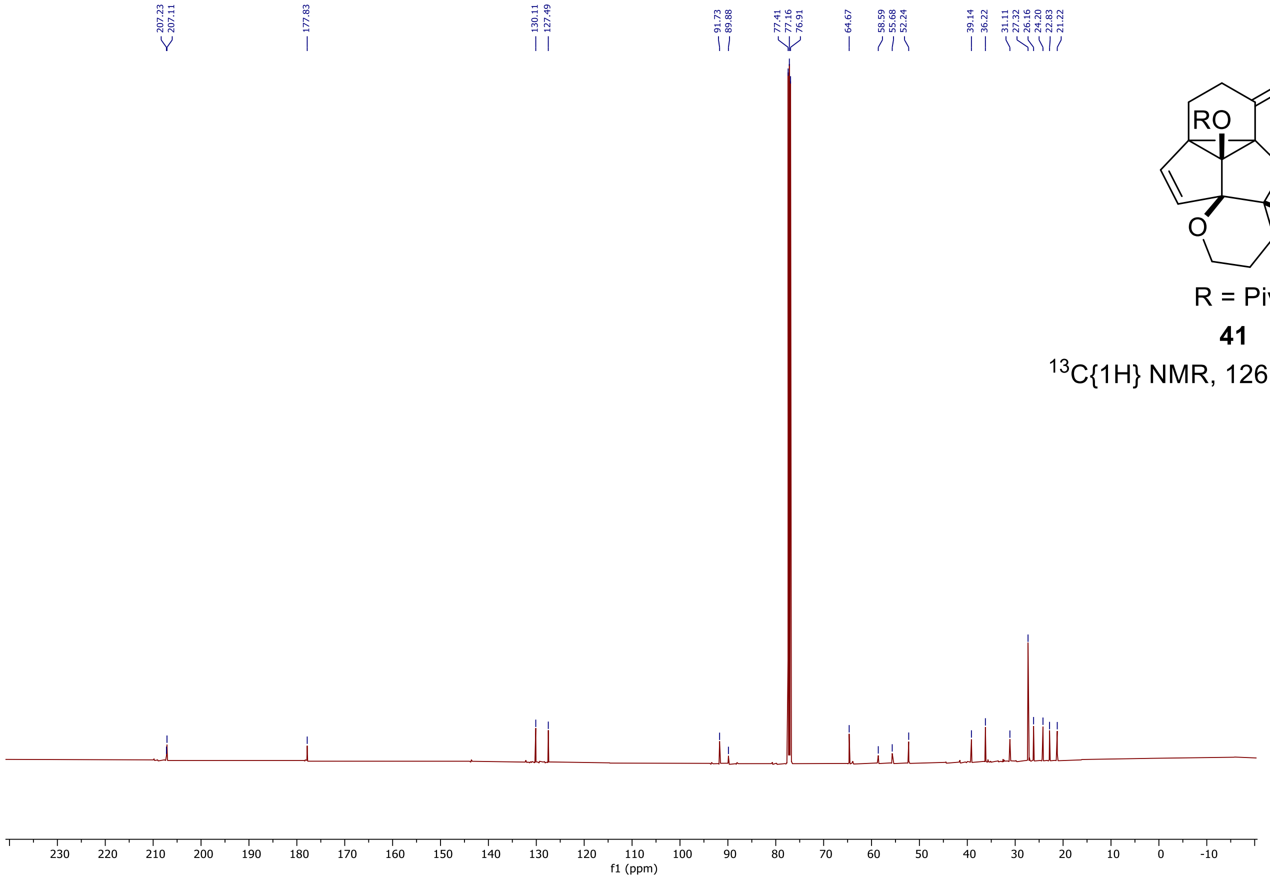

R = Piv

41

^{13}C{1H} NMR, 126 MHz, $CDCl_3$

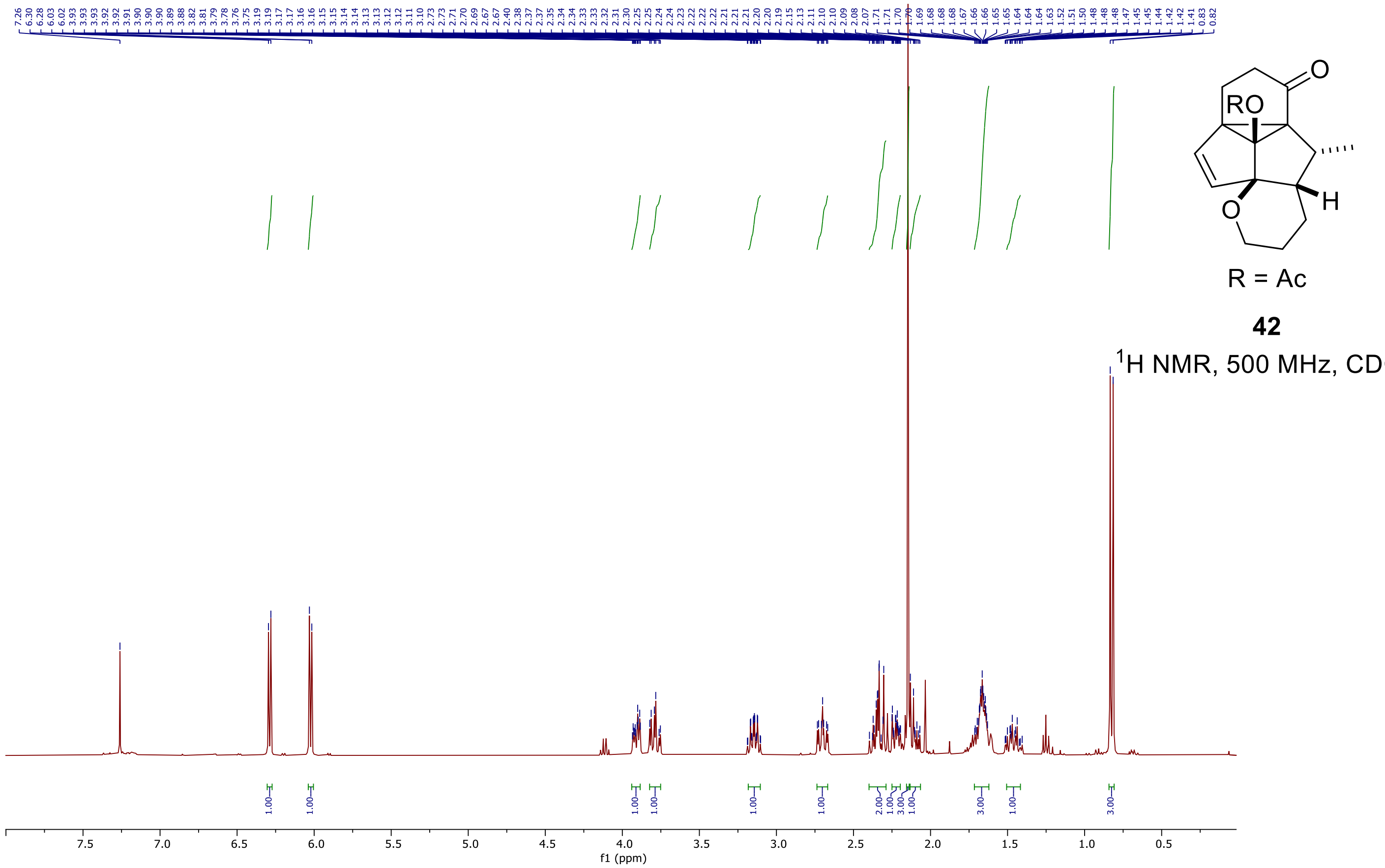

R = Ac

42

^{1}H NMR, 500 MHz, $CDCl_3$

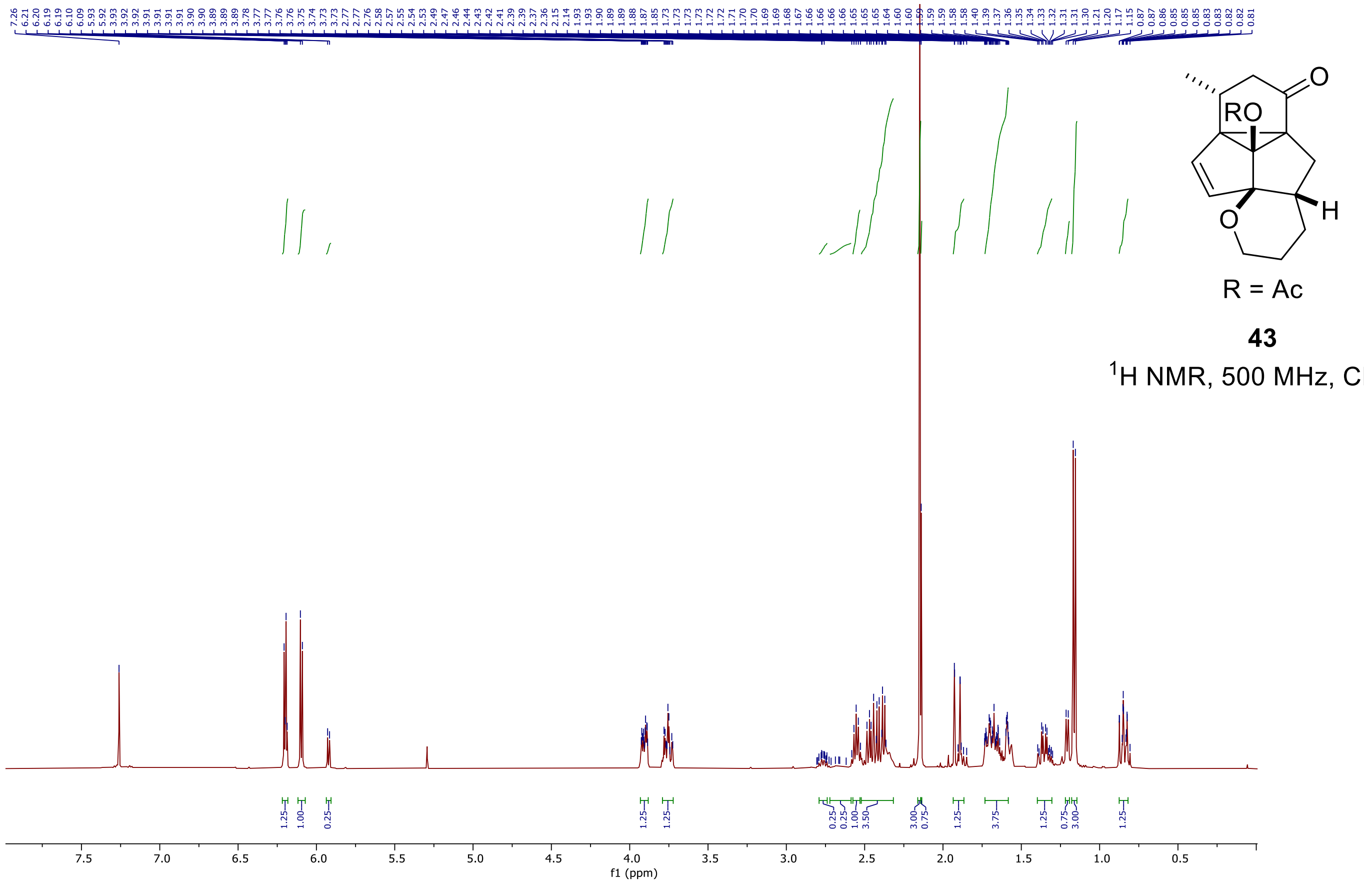

R = Ac

43

^{1}H NMR, 500 MHz, $CDCl_3$

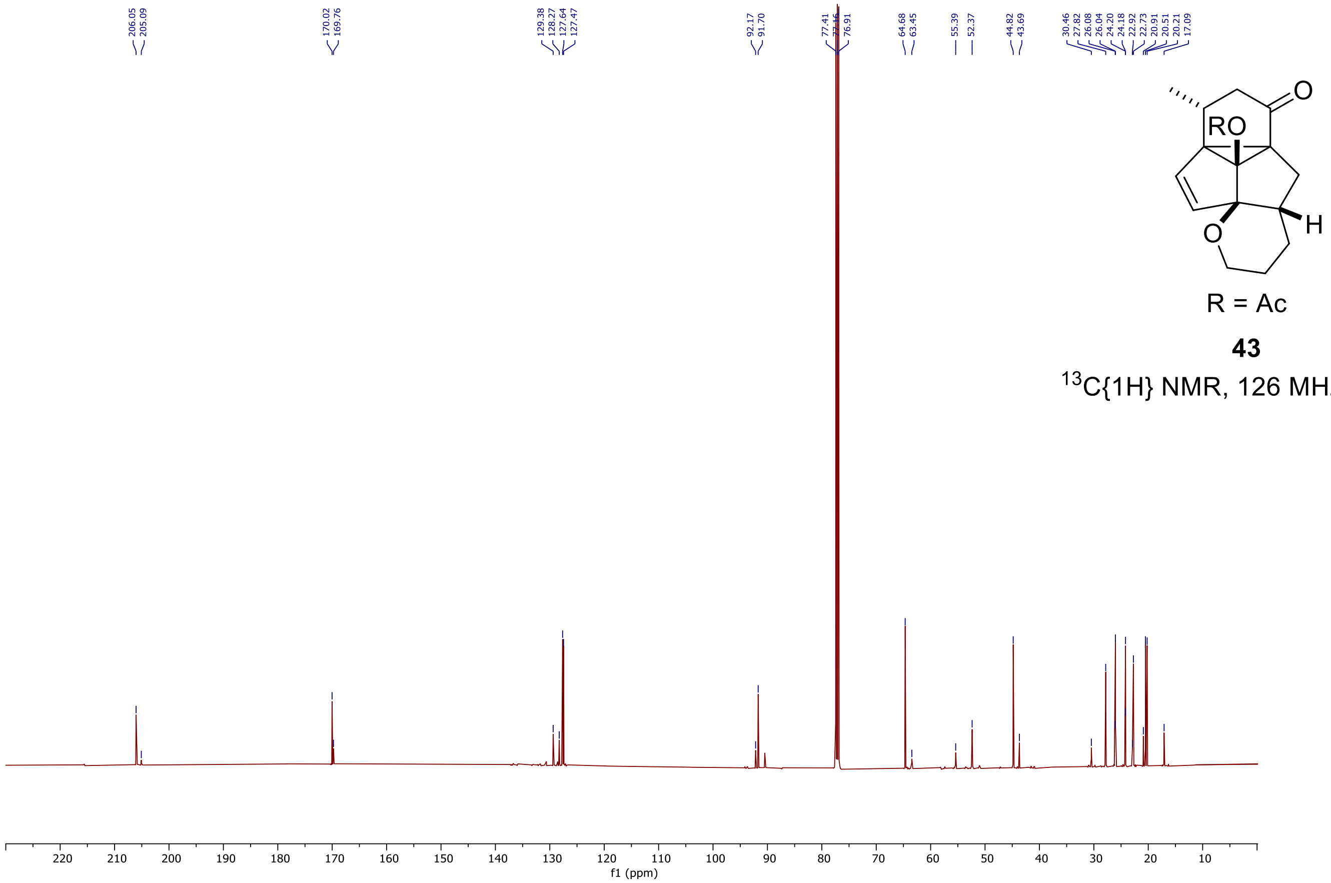

R = Ac

43

$^{13}C\{1H\}$ NMR, 126 MHz, $CDCl_3$

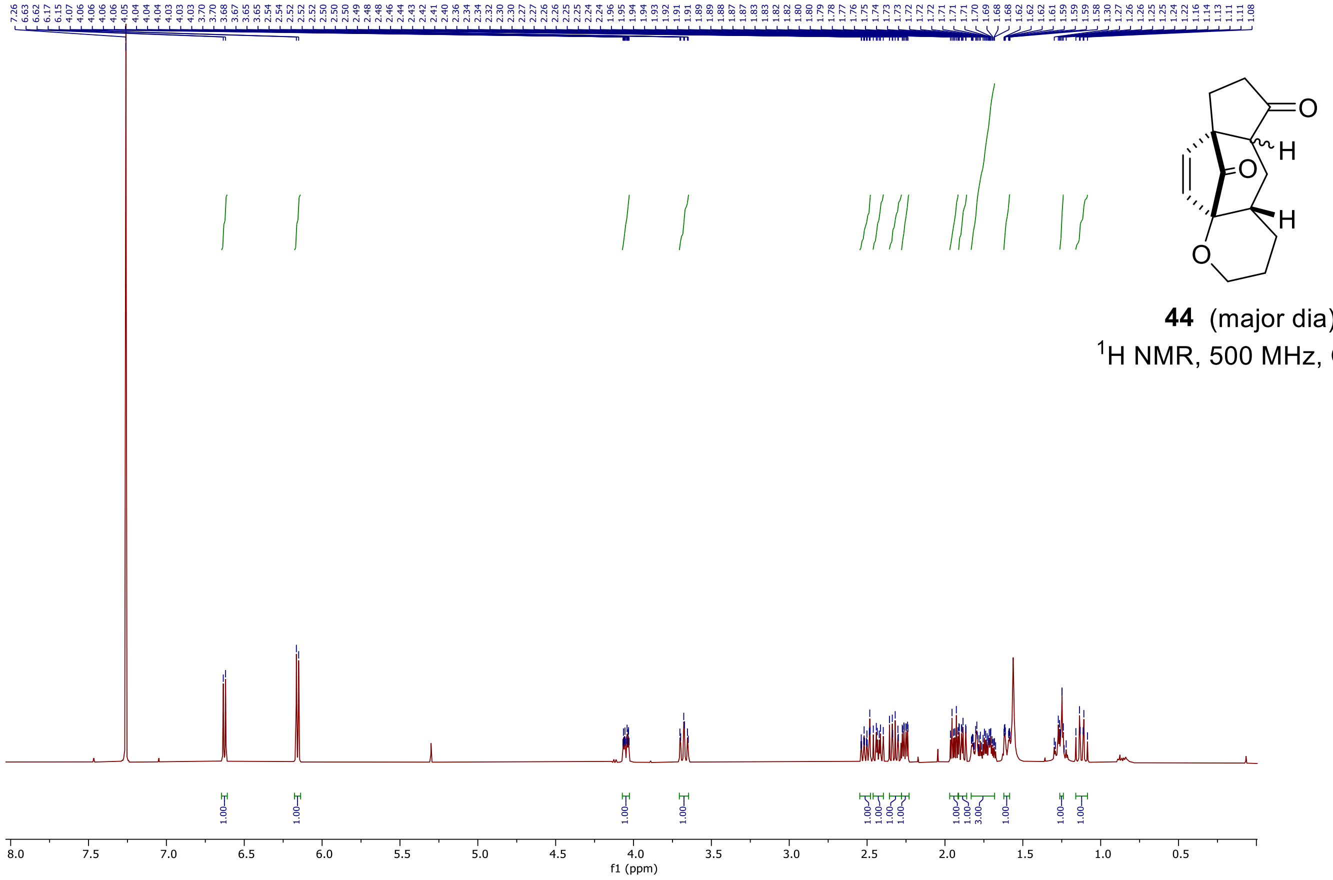

44 (major dia)

^{1}H NMR, 500 MHz, $CDCl_3$

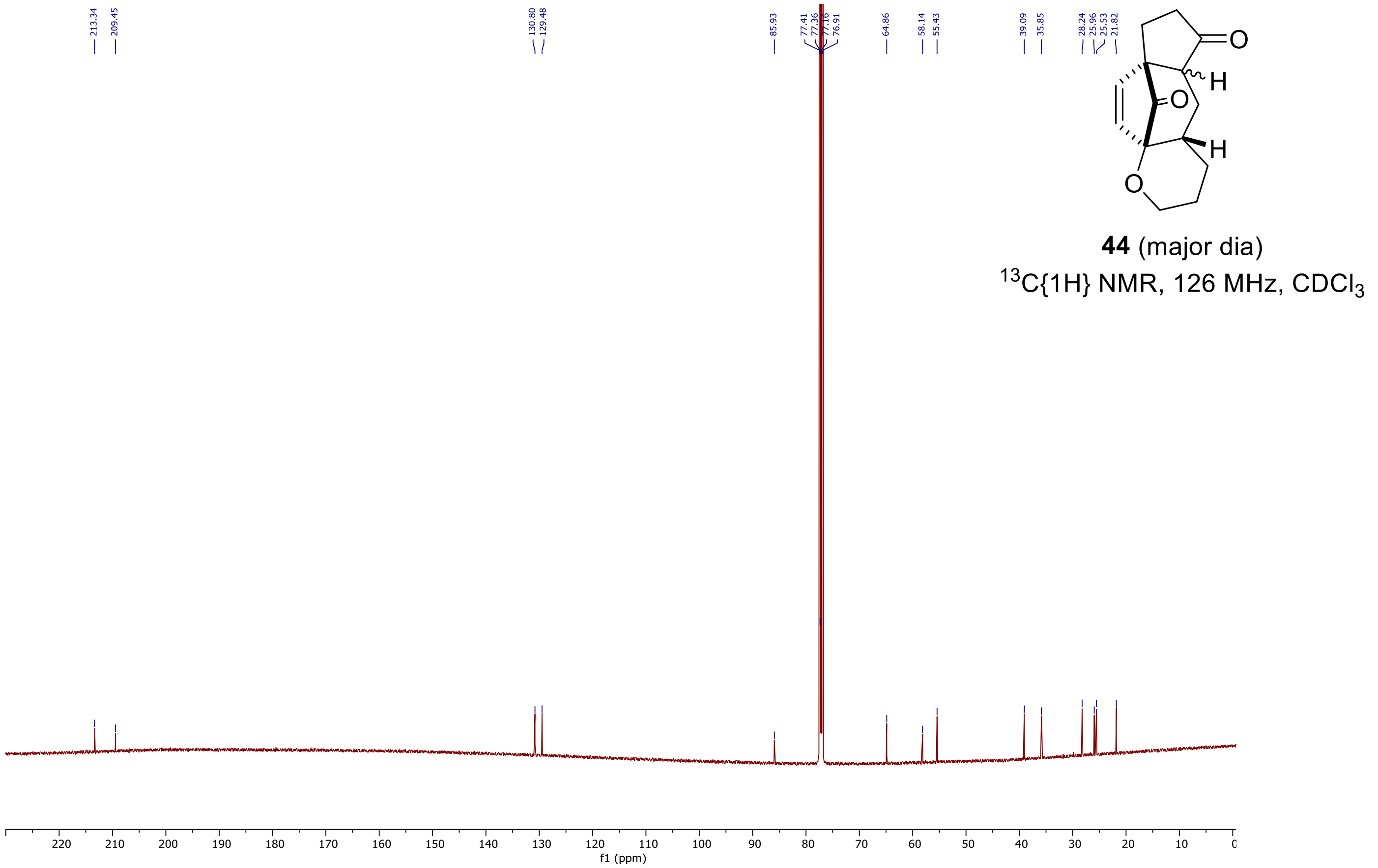

44 (major dia)

$^{13}C\{1H\}$ NMR, 126 MHz, $CDCl_3$

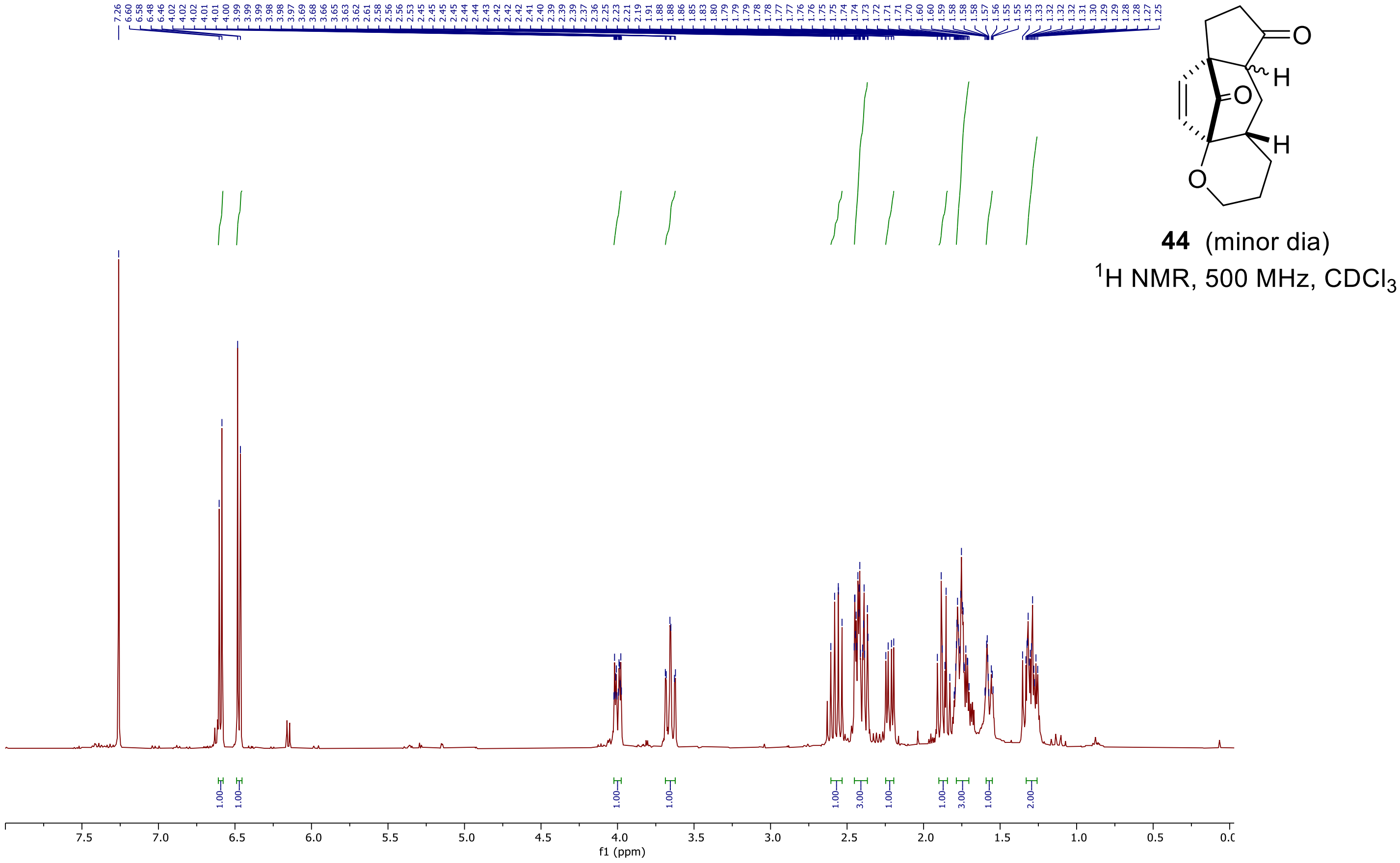

44 (minor dia)

^{1}H NMR, 500 MHz, $CDCl_3$

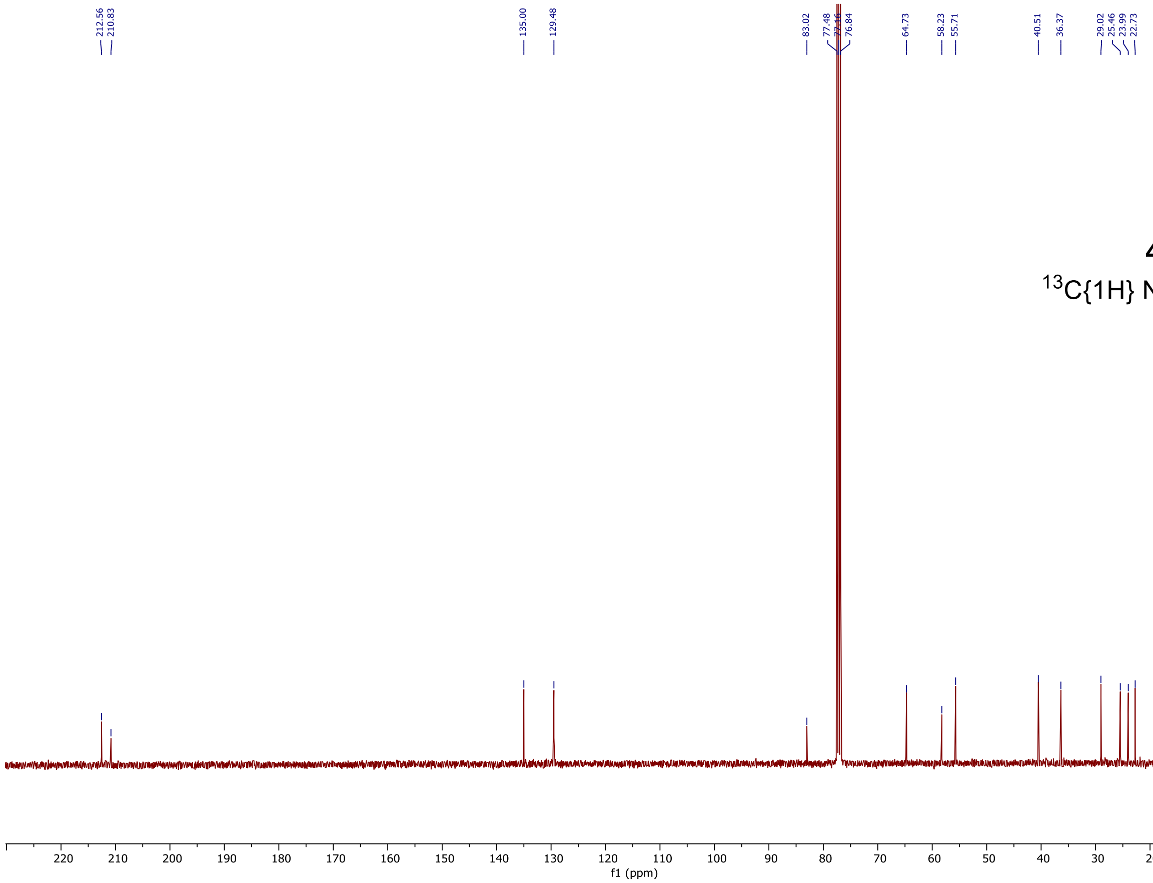

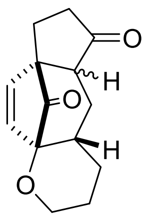

44 (minor dia)

$^{13}C\{1H\}$ NMR, 126 MHz, $CDCl_3$
